# Comparative genomic analysis of eutherian adiponectin genes

**DOI:** 10.1016/j.heliyon.2018.e00647

**Published:** 2018-06-06

**Authors:** Marko Premzl

**Affiliations:** The Australian National University Alumni, 4 Kninski trg Sq., Zagreb, Croatia

**Keywords:** Genetics

## Abstract

The present study proposed updated and standardized classification and nomenclature of eutherian adiponectin genes implicated in regulation of systemic metabolism and inflammation and activation of classical complement pathway. The revisions of comprehensive adiponectin gene data sets used eutherian comparative genomic analysis protocol and public reference genomic sequence assemblies. Among 438 potential coding sequences, the tests of reliability of eutherian public genomic sequences annotated most comprehensive curated third-party data gene data set of eutherian adiponectin genes that included 211 complete coding sequences. There were 18 major gene clusters of eutherian adiponectin genes described, one of which included evidence of differential gene expansions. For example, the present analysis initially described human *ADIF2* and *ADIR* genes. Finally, the tests of protein molecular evolution using relative synonymous codon usage statistics confirmed protein primary structure similarities between eutherian adiponectins and tumor necrosis factor ligands.

## 1 Introduction

The comprehensive eutherian adiponectin gene data sets were implicated in major physiological processes, including regulation of systemic metabolism and inflammation (Seldin et al., 2014; Luo and Liu, 2016b) and activation of classical complement pathway (Kishore et al., 2004a,b). The eutherian adiponectin homologues including adipokine adiponectin ACRP30, C1q classical complement subcomponent A, B and C chains and C1q/tumor necrosis factor-α-related proteins were described as one homologue subgroup that shared protein primary structure similarities with cerebellins, collagens, emilins, multimerins and otolins (Sellar et al., 1991; Scherer et al., 1995; Shapiro and Scherer, 1998; Arlaud et al., 2002; Bodmer et al., 2002; Gaboriaud et al., 2003; Kishore et al., 2004a,b; Wong et al. 2004, 2008; Seldin et al., 2014; Luo and Liu, 2016a,b). First, the adipokine adiponectin ACRP30 (Scherer et al., 1995; Shapiro and Scherer, 1998) regulated insulin sensitivity in multiple tissues, as well as energy homeostasis and thermogenesis (Luo and Liu, 2016b). In addition, the ACRP30 was described as obesity molecular marker having regulatory effect on innate immunity (Luo and Liu, 2016a). Second, the C1q classical complement subcomponent included A, B and C chains (Sellar et al., 1991) that associated into quaternary structures resembling tulips (Kishore et al., 2004a,b). Such C1q protein structures provided molecular receptors to multiple ligands triggering activation of classical complement pathway (Arlaud et al., 2002; Gaboriaud et al., 2003; Kishore et al., 2004a,b). Finally, there were C1q/tumor necrosis factor-α-related proteins CTRPs having protein primary structure features that were similar to those of ACRP30 and C1q A-C1q C (Wong et al. 2004, 2008). The most recently described CTRPs were implicated in both metabolic and non-metabolic functions, as reviewed by Seldin et al. (2014). Therefore, the similarities of protein amino acid sequence features among eutherian ACRP30, C1q A-C1q C and CTRP homologue data sets were well established (Scherer et al., 1995; Kishore et al., 2004a,b; Seldin et al., 2014). Of note, the public eutherian reference genomic sequence data sets greatly advanced biological and medical sciences (Murphy et al., 2001; Wilson and Reeder, 2005; Blakesley et al., 2004; Margulies et al., 2005; Lindblad-Toh et al., 2011; O'Leary et al., 2013). For example, the initial sequencing and analysis of human genome made attempts to update and revise human gene data sets and uncover potential new drugs, drug targets and molecular markers in medical diagnostics (International Human Genome Sequencing Consortium, 2001). Yet, future updates and revisions of eutherian reference gene data sets were expected, due to the incompleteness of eutherian reference genomic sequence assemblies (International Human Genome Sequencing Consortium, 2001; Harrow et al., 2012) and potential genomic sequence errors (International Human Genome Sequencing Consortium, 2004; Mouse Genome Sequencing Consortium, 2009). Specifically, the potential genomic sequence errors included analytical and bioinformatical errors (erroneous gene annotations, genomic sequence misassemblies) and Sanger DNA sequencing method errors (artefactual nucleotide deletions, insertions and substitutions). For example, the so-called lexicographical bias was described in some genomic sequence assembly programs (Gajer et al., 2004) and reference genomic sequence assemblies including lower genomic sequence redundancies were more likely to include potential genomic sequence errors (Hubisz et al., 2011; Prosdocimi et al., 2012; Denton et al., 2014). The eutherian comparative genomic analysis protocol was established as guidance in protection against potential genomic sequence errors in public eutherian reference genomic sequence data sets (Premzl, 2015, 2016, 2017). The protocol published new test of reliability of public eutherian genomic sequences using genomic sequence redundancies and new protein molecular evolution test using relative synonymous codon usage statistics. The protocol was applicable in revisions of 10 major eutherian gene data sets including 1293 complete coding sequences deposited in European Nucleotide Archive. Thus, using eutherian comparative genomic analysis protocol and free available eutherian reference genomic sequence data sets, the present study made attempts to update and revise comprehensive eutherian adiponectin *ADI* gene data sets.

## 2 Materials and methods

The eutherian comparative genomic analysis protocol RRID:SCR_014401 including gene annotations, phylogenetic analysis and protein molecular evolution analysis was published on Nature Protocol Exchange (https://doi.org/10.1038/protex.2018.028).

### 2.1 Gene annotations

First, there were gene identifications in public genomic sequences, analyses of gene features, tests of reliability of eutherian public genomic sequences and multiple pairwise genomic sequence alignments included in eutherian *ADI* gene annotations. In analyses of *ADI* nucleotide and protein sequences, the sequence alignment editor BioEdit 7.0.5.3 was used (http://www.mbio.ncsu.edu/BioEdit/bioedit.html). The gene identifications of potential *ADI* coding sequences used public eutherian genomic sequence assemblies (Benson et al., 2018) and BLAST programs (Altschul et al., 1997) made available by National Center for Biotechnology Information (NCBI) (https://blast.ncbi.nlm.nih.gov/Blast.cgi) (NCBI Resource Coordinators, 2018) and Ensembl genome browser (http://www.ensembl.org/index.html) (Zerbino et al., 2018). The direct evidence of gene annotations deposited in NCBI's nr, est_human, est_mouse and est_others databases were used in analyses of *ADI* gene features (https://www.ncbi.nlm.nih.gov). The tests of reliability of eutherian public genomic sequences used potential *ADI* coding sequences. Using NCBI's BLAST programs and genomic sequence reads deposited in NCBI's Trace Archive (https://www.ncbi.nlm.nih.gov/Traces/trace.cgi), the first test steps analysed nucleotide sequence coverages of each potential *ADI* coding sequence. The potential *ADI* coding sequences were annotated as complete *ADI* coding sequences if consensus read nucleotide sequence coverages were available for every nucleotide of each potential *ADI* coding sequence. Only the complete *ADI* coding sequences were used in analyses. Alternatively, the potential *ADI* coding sequences were described as putative *ADI* coding sequences and not used in analyses. The complete *ADI* coding sequences were also deposited in European Nucleotide Archive as curated eutherian third-party data gene data set (http://www.ebi.ac.uk/ena/about/tpa-policy) (Gibson et al., 2016; Karsch-Mizrachi et al., 2018; Silvester et al., 2018). In revised *ADI* gene classification and nomenclature, the guidelines of human (http://www.genenames.org/about/guidelines) and mouse (http://www.informatics.jax.org/mgihome/nomen/gene.shtml) gene nomenclatures were used. The multiple pairwise genomic sequence alignments of *ADI* genes used mVISTA's AVID program using default settings (http://genome.lbl.gov/vista/index.shtml) (Dubchak and Ryaboy, 2006). In pairwise alignments, the cut-offs of detection of common genomic sequence regions in alignments with base sequences (*Homo sapiens*) were: 95% per 100 bp (*Pan troglodytes*, *Gorilla gorilla*), 90% per 100 bp (*Pongo abelii*, *Nomascus leucogenys*), 85% per 100 bp (*Macaca mulatta*, *Papio hamadryas*), 80% per 100 bp (*Callithrix jacchus*), 75% per 100 bp (*Tarsius syrichta*, *Microcebus murinus*, *Otolemur garnettii*), 65% per 100 bp (rodents) or 70% per 100 bp in other pairwise alignments (Murphy et al., 2001; Wilson and Reeder, 2005; Blakesley et al., 2004; Margulies et al., 2005; Lindblad-Toh et al., 2011; O'Leary et al., 2013; Premzl, 2015, 2016, 2017). One exception was pairwise genomic sequence alignment between mouse (base sequence) and brown rat *Adil* genes having empirically determined cut-off 85% per 100 bp. In human base sequences, the RepeatMasker program version open-4.0.5 was used in detections and maskings of transposable elements using default settings, except that simple repeats and low complexity elements were not masked (sensitive mode, cross_match version 1.080812, RepBase Update 20140131 and RM database version 20140131) (http://www.repeatmasker.org/). Using ClustalW included in BioEdit 7.0.5.3, the common predicted *ADI* promoter genomic sequence regions were aligned at nucleotide sequence level and then manually corrected. The pairwise nucleotide sequence identities of *ADI* promoters were calculated using BioEdit 7.0.5.3 and used in statistical analyses that included calculations of average pairwise identities (*ā*) and their average absolute deviations (*ā*_ad_), largest pairwise identities (*a*_max_) and smallest pairwise identities (*a*_min_) using Microsoft Office Excel common statistical functions.

### 2.2 Phylogenetic analysis

Second, there were protein and nucleotide sequence alignments, calculations of phylogenetic trees and calculations of pairwise nucleotide sequence identities included in phylogenetic analysis. The complete *ADI* coding sequences were translated using BioEdit 7.0.5.3, and aligned at amino acid level using ClustalW included in BioEdit 7.0.5.3. The *ADI* nucleotide sequence alignments were prepared accordingly, after manual corrections of protein amino acid sequence alignments. The MEGA 6.06 program was used in *ADI* phylogenetic tree calculations (http://www.megasoftware.net) (Tamura et al., 2013), using minimum evolution method that was applicable in analysis of distant and very distant eutherian homologues (default settings, except gaps/missing data treatment = pairwise deletion). The pairwise nucleotide sequence identities of *ADI* nucleotide sequence alignments were calculated using BioEdit 7.0.5.3. Their statistical analyses included calculations of average pairwise identities (*ā*) and their average absolute deviations (*ā*_ad_), largest pairwise identities (*a*_max_) and smallest pairwise identities (*a*_min_) using Microsoft Office Excel common statistical functions.

### 2.3 Protein molecular evolution analysis

Third, the protein molecular evolution analysis included tests of protein molecular evolution that integrated patterns of nucleotide sequence similarities with protein primary structures. The complete *ADI* nucleotide sequence alignments including 211 eutherian homologues were used in tests of protein molecular evolution. In calculations of *ADI* codon usage statistics, the MEGA 6.06 program was used. The ratios between observed and expected amino acid codon counts determined relative synonymous codon usage statistics (*R*). The not preferable amino acid codons including *R ≤* 0.7 included 25 codons: TTT (0,52), TTA (0,19), TTG (0,46), CTT (0,65), CTA (0,31), ATA (0,28), GTT (0,38), GTA (0,27), TCA (0,62), TCG (0,56), CCG (0,68), ACG (0,66), GCA (0,63), GCG (0,66), TAT (0,57), CAT (0,62), CAA (0,42), AAT (0,7), AAA (0,68), GAT (0,64), GAA (0,64), TGT (0,67), CGT (0,44), AGT (0,64) and GGT (0,53). The reference human ADIA protein sequence amino acid sites were then described as invariant amino acid sites (invariant alignment positions), forward amino acid sites (variant alignment positions not including amino acid codons with *R ≤* 0.7) or compensatory amino acid sites (variant alignment positions including amino acid codons with *R ≤* 0.7) using protein and nucleotide sequence alignments. The SignalP 4.1 server was used in predictions of N-terminal signal peptide cleavage sites in ADI protein primary structures using default settings (http://www.cbs.dtu.dk/services/SignalP/), as well as ADI protein sequence alignments.

## 3 Results and discussion

### 3.1 Gene annotations

**Fig. 1:**
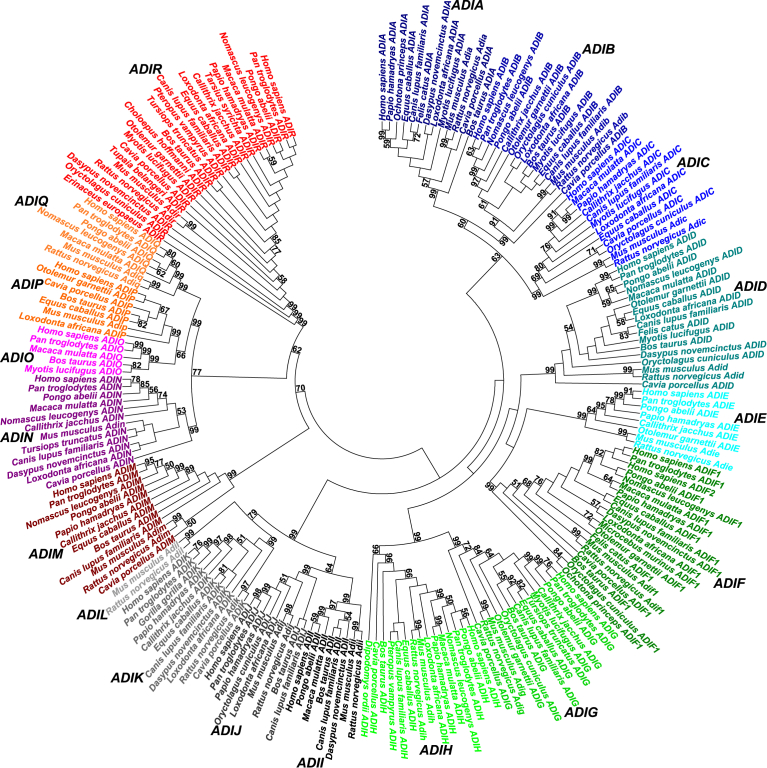
Minimum evolution phylogenetic tree of eutherian adiponectin genes. The tree was calculated using maximum composite likelihood method. The bootstrap estimates higher than 50% were shown after 1000 replicates. The major gene clusters of eutherian adiponectin genes *ADIA*-*ADIR* were indicated.

Among 438 potential coding sequences, the tests of reliability of eutherian public genomic sequences annotated most comprehensive gene data set of eutherian *ADI* genes that included 211 complete coding sequences (Fig. 1) (Supplementary data file 1). The curated third-party data gene data set of eutherian *ADI* genes was deposited in European Nucleotide Archive under accession numbers LT962964–LT963174 (https://www.ebi.ac.uk/ena/data/view/LT962964-LT963174). The present analysis described 18 major gene clusters of eutherian *ADI* genes *ADIA*-*ADIR* (Supplementary data file 2). First, the major gene cluster *ADIA* included 13 *C1q B* genes (panel A of Supplementary data file 2 – part 1). There were 15 *C1q A* genes included in major gene cluster *ADIB* (panel B of Supplementary data file 2 – part 1). For example, the common predicted Anthropoidea *ADIB* promoter genomic sequence region was annotated, including average pairwise nucleotide sequence identity *ā* = 0.923 (*a*_max_ = 0.982, *a*_min_ = 0.849, *ā*_ad_ = 0.058) (panel A of Supplementary data file 3). The major gene cluster *ADIC* included 12 *C1q C* genes (panel C of Supplementary data file 2 – part 1). The common predicted Anthropoidea *ADIC* promoter genomic sequence region included *ā* = 0.904 (*a*_max_ = 0.979, *a*_min_ = 0.862, *ā*_ad_ = 0.039) (panel B of Supplementary data file 3). Whereas there were 17 *ACRP30* genes included in major gene cluster *ADID* (panel D of Supplementary data file 2 – part 2), major gene cluster *ADIE* included 8 *CTRP5* genes (panel E of Supplementary data file 2 – part 2). For example, the common predicted Anthropoidea *ADIE* promoter genomic sequence region included *ā* = 0.946 (*a*_max_ = 0.994, *a*_min_ = 0.899, *ā*_ad_ = 0.028) (panel C of Supplementary data file 3). The major gene cluster *ADIF* included 20 *CTRP9* genes (panel F of Supplementary data file 2 – part 2). The common predicted Hominidae *ADIF* promoter genomic sequence region included *ā* = 0.945 (*a*_max_ = 0.976, *a*_min_ = 0.928, *ā*_ad_ = 0.011) (panel D of Supplementary data file 3). Only the major gene cluster *ADIF* included evidence of differential gene expansions. Specifically, there were human *ADIF1* and *ADIF2* paralogues annotated, in contrast to analyses of Wong et al. (2008) and Seldin et al. (2014) including only 1 human *CTRP9* gene (*ADIF1*). For example, the direct evidence of *ADIF1* and *ADIF2* gene annotations included selected human transcripts BC040438.1 (*ADIF1*) and BC137004.1 and BC137006.1 (*ADIF2*), as well as other human transcripts and ESTs (data not shown). In addition, the indirect evidence of *ADIF1* and *ADIF2* gene annotations included pairwise nucleotide sequence identities *a* = 0.99 between human *ADIF1* and *ADIF2* complete coding sequences (panel F of Supplementary data file 2 – part 2) and *a* = 0.93 between human *ADIF1* and *ADIF2* predicted promoter genomic sequence regions (panel D of Supplementary data file 3). The major gene cluster *ADIG* included 14 *CTRP7* genes (panel G of Supplementary data file 2 – part 3). For example, the common predicted Anthropoidea *ADIG* promoter genomic sequence region included *ā* = 0.941 (*a*_max_ = 0.988, *a*_min_ = 0.903, *ā*_ad_ = 0.037) (panel E of Supplementary data file 3). The major gene cluster *ADIH* included 15 *CTRP2* genes (panel H of Supplementary data file 2 – part 3). The common predicted Catarrhini *ADIH* promoter genomic sequence region included *ā* = 0.967 (*a*_max_ = 0.992, *a*_min_ = 0.95, *ā*_ad_ = 0.01) (*Papio hamadryas* was not included) (panel F of Supplementary data file 3). The major gene cluster *ADII* included 8 *CTRP10* genes (panel I of Supplementary data file 2 – part 3). The common predicted Catarrhini *ADII* promoter genomic sequence region included *ā* = 0.967 (*a*_max_ = 0.977, *a*_min_ = 0.958, *ā*_ad_ = 0.006) (panel G of Supplementary data file 3). The major gene cluster *ADIJ* included 9 *CTRP13* genes (panel J of Supplementary data file 2 – part 4). The common predicted Catarrhini *ADIJ* promoter genomic sequence region included *ā* = 0.984 (*a*_max_ = 0.994, *a*_min_ = 0.979, *ā*_ad_ = 0.006) (panel H of Supplementary data file 3). The major gene cluster *ADIK* included 11 *CTRP11* genes (panel K of Supplementary data file 2 – part 4). The common predicted Anthropoidea *ADIK* promoter genomic sequence region included *ā* = 0.928 (*a*_max_ = 0.993, *a*_min_ = 0.869, *ā*_ad_ = 0.044) (panel I of Supplementary data file 3). Whereas there were 2 rodent *CTRP14* genes included in major gene cluster *ADIL* (panel L of Supplementary data file 2 – part 4), major gene cluster *ADIM* included 12 *CTRP3* genes (panel M of Supplementary data file 2 – part 4). For example, the common predicted Anthropoidea *ADIM* promoter genomic sequence region included *ā* = 0.936 (*a*_max_ = 0.993, *a*_min_ = 0.855, *ā*_ad_ = 0.042) (panel J of Supplementary data file 3). Whereas there were 12 *CTRP4* genes included in major gene cluster *ADIN* (panel N of Supplementary data file 2 – part 5), major gene cluster *ADIO* included 5 *CTRP8* genes (panel O of Supplementary data file 2 – part 5). The common predicted Catarrhini *ADIO* promoter genomic sequence region included *ā* = 0.918 (*a*_max_ = 0.979, *a*_min_ = 0.877, *ā*_ad_ = 0.041) (panel K of Supplementary data file 3). Whereas there were 7 *CTRP1* genes included in major gene cluster *ADIP* (panel P of Supplementary data file 2 – part 5), major gene cluster *ADIQ* included 7 *CTRP6* genes (panel Q of Supplementary data file 2 – part 5). The common predicted Catarrhini *ADIQ* promoter genomic sequence region included *ā* = 0.969 (*a*_max_ = 0.992, *a*_min_ = 0.942, *ā*_ad_ = 0.018) (panel L of Supplementary data file 3). Finally, the present study initially described major gene cluster *ADIR* including 24 genes (panel R of Supplementary data file 2 – part 6). For example, the direct evidence of *ADIR* gene annotations included selected human transcripts BC007520.1, BC066295.1 and BC111007.1 and other transcripts and ESTs (data not shown). The indirect evidence of *ADIR* gene annotations included 24 eutherian complete coding sequences (panel R of Supplementary data file 2 – part 6), as well as common predicted primate *ADIR* promoter genomic sequence region including *ā* = 0.967 (*a*_max_ = 1, *a*_min_ = 0.932, *ā*_ad_ = 0.021) (panel M of Supplementary data file 3). There were 1–6 translated exons included in eutherian *ADI* genes. For example, whereas the human *ADIR* gene included 1 translated exon along 477 bp (panel R of Supplementary data file 2 – part 6), there were 6 translated exons in human *ADIM* gene along 22876 bp (panel M of Supplementary data file 2 – part 4). Within eutherian major gene clusters, respectively, the *ADI* translated exon numbers were constant. Of note, the *ADI* gene number estimates in each species were subject to future updates and refinements due to incompleteness of genomic sequence assemblies and potential genomic sequence errors (International Human Genome Sequencing Consortium 2001, 2004; Harrow et al., 2012) (Supplementary data file 1). Yet, the present major gene cluster *ADIA*-*ADIR* descriptions indicated that 18 was minimal expected number of *ADI* genes per eutherian species.

### 3.2 Phylogenetic analysis

The minimal evolution phylogenetic tree calculations distributed 18 major gene clusters of eutherian *ADI* genes into 5 groups (Fig. 1). The first group included major gene clusters *ADIA* (*C1q B*), *ADIB* (*C1q A*) and *ADIC* (*C1q C*). The grouping of major gene clusters *ADIA*-*ADIC* separate to other *ADI* genes agreed with Bodmer et al. (2002) and Seldin et al. (2014) but disagreed with Kishore et al. (2004a). The second group included major gene clusters *ADID* (*ACRP30*), *ADIE* (*CTRP5*), *ADIF* (*CTRP9*), *ADIG* (*CTRP7*) and *ADIH* (*CTRP2*). For example, the clustering of major gene clusters *ADIG* and *ADIH* was in agreement with Wong et al. (2008). Whereas the third group included major gene clusters *ADII* (*CTRP10*), *ADIJ* (*CTRP13*), *ADIK* (*CTRP11*) and *ADIL* (*CTRP14*), fourth group included single major gene cluster *ADIM* (*CTRP3*). Finally, the fifth group included major gene clusters *ADIN* (*CTRP4*), *ADIO* (*CTRP8*), *ADIP* (*CTRP1*), *ADIQ* (*CTRP6*) and *ADIR*. For example, the clustering of major gene clusters *ADIO*-*ADIQ* disagreed with Wong et al. (2008). Such phylogenetic distribution of 18 major gene clusters of eutherian *ADI* genes was confirmed by calculations of pairwise nucleotide sequence identity patterns (Supplementary data file 4). First, the complete *ADI* nucleotide sequence alignments including 211 eutherian homologues included average pairwise nucleotide sequence identity *ā* = 0.289 (*a*_max_ = 1, *a*_min_ = 0.107, *ā*_ad_ = 0.11). Among 18 eutherian *ADI* major gene clusters respectively, there were nucleotide sequence identity patterns of very close eutherian orthologues (*ADIL*), close eutherian orthologues (*ADIE*, *ADIJ*, *ADIK*, *ADIN* and *ADIR*), typical eutherian orthologues (*ADIA*-*ADID*, *ADIG*-*ADII*, *ADIM*, *ADIO*-*ADIQ*) and very close eutherian orthologues and paralogues (*ADIF*). In comparisons between eutherian *ADI* major gene clusters, there were nucleotide sequence identity patterns of very close eutherian homologues in comparisons between major gene clusters *ADIG* and *ADIH* agreeing with Wong et al. (2008), in comparisons between major gene clusters *ADII*-*ADIL* and in comparisons between major gene clusters *ADIO* and *ADIQ* disagreeing with Wong et al. (2008). In other comparisons between major gene clusters, there were nucleotide sequence identity patterns of close, typical, distant and very distant eutherian homologues. For example, there were nucleotide sequence identity patterns of close eutherian homologues in comparisons between major gene clusters *ADIA*-*ADIC*. The minimal evolution phylogenetic tree calculations and calculations of pairwise nucleotide sequence identity patterns confirmed present descriptions of major gene clusters *ADIA*-*ADIR* within one gene data set of eutherian homologues, and justified exclusion of cerebellins, collagens, emilins, multimerins and otolins from present analysis (Sellar et al., 1991; Scherer et al., 1995; Shapiro and Scherer, 1998; Arlaud et al., 2002; Bodmer et al., 2002; Gaboriaud et al., 2003; Kishore et al., 2004a,b; Wong et al. 2004, 2008; Seldin et al., 2014; Luo and Liu, 2016a,b).

### 3.3 Protein molecular evolution analysis

**Fig. 2:**
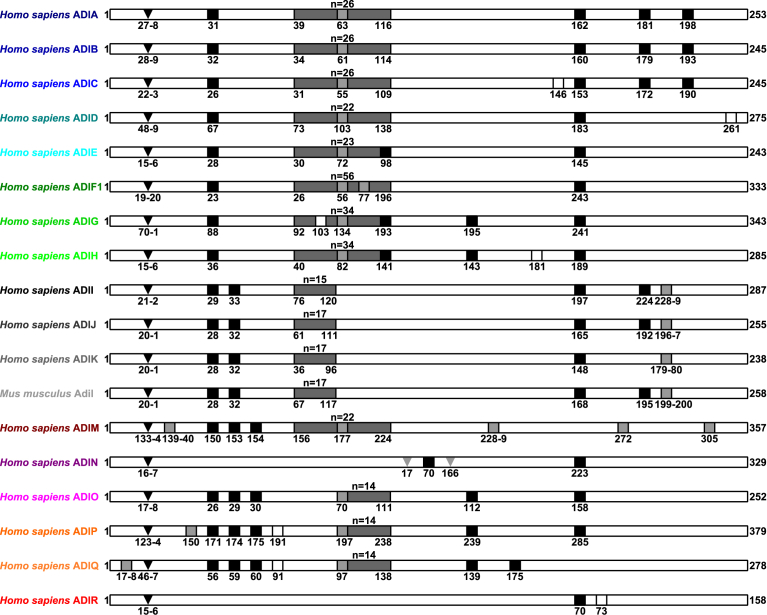
Major landmarks in eutherian adiponectin protein sequence alignments. The black squares labelled common cysteine amino acid residues. The grey squares labelled common exon-intron splice site amino acid sites. The white squares labelled common N-glycosylation sites. The dark grey rectangles displayed low complexity regions having numbers of tripeptide repeats (G-x(2)) shown above each rectangle (n). Whereas the black triangles labelled predicted N-terminal signal peptide cleavage sites, grey triangles labelled N-terminal and C-terminal amino acid sites of first ADIN C-terminal region. The numbers indicated numbers of amino acid residues.

The major landmarks in ADI protein sequence alignments included several protein primary structure features common to 18 eutherian major protein clusters ADIA-ADIR (Fig. 2) (Supplementary data file 5). First, the major protein clusters ADIA-ADIR, respectively, included 1–5 common cysteine amino acid residues. Second, the major protein clusters ADIA-ADIR, respectively, included 0–1 common N-glycosylation sites. Third, whereas the major protein clusters ADIA-ADIR, respectively, included common predicted N-terminal signal peptides, there were 0–5 exon-intron splice sites common to major protein clusters ADIA-ADIR, respectively. Finally, the major protein clusters ADIA-ADIM and ADIO-ADIQ, respectively, included common low complexity regions including imperfect tandem simple tripeptide repeats (G-x(2))_n_. For example, the human ADIs included 14–56 tripeptides (_n_), except that ADIN and ADIR did not include such low complexity regions. Whereas the eutherian ADINs included 2 common C-terminal regions, only 1 C-terminal region was common to eutherian ADIRs. Next, using complete protein and nucleotide sequence alignments (Supplementary data file 5), the tests of protein molecular evolution integrated patterns of *ADI* nucleotide sequence similarities with ADI protein primary structures. For example, among 253 reference human ADIA amino acid residues, there were 33 invariant amino acid sites and 1 forward amino acid site (Supplementary data file 6). First, the invariant amino acid sites included 3 common cysteine amino acid residues C31, C162 and, finally, C181 that was implicated in disulphide bonding (Gaboriaud et al., 2003). Second, there were 21 glycine amino acid residues in tripeptide repeats (G-x(2))_n=26_ included in invariant amino acid sites. Finally, the invariant amino acid sites included 8 amino acid residues common to 211 ADI protein primary structures (F124, F142, N148, F160, G166, Y168, F242 and G244). For example, the human ADIA invariant amino acid sites F124, F142, F160, G166, Y168, F242 and G244 corresponded to human tumor necrosis factor ligand TNLG1B invariant amino acid sites H98, W119, L139, G145, Y147, F246 and G247 (Premzl, 2016) that confirmed protein primary structure similarities between eutherian ADIs and tumor necrosis factor ligands (Shapiro and Scherer, 1998; Bodmer et al., 2002; Gaboriaud et al., 2003; Kishore et al., 2004a; Wong et al., 2008).

## 4 Conclusions

The present study attempted to update and revise comprehensive eutherian *ADI* gene data sets using eutherian comparative genomic analysis protocol and public eutherian reference genomic sequence data sets. First, among 438 potential coding sequences, the tests of reliability of public eutherian genomic sequences using genomic sequence redundancies annotated most comprehensive curated third-party data gene data set of eutherian *ADI* genes including 211 complete coding sequences. Second, the phylogenetic analysis of eutherian *ADI* genes descibed 18 major gene clusters *ADIA*-*ADIR*. For example, the major gene cluster *ADIF* included evidence of differential gene expansions, and human *ADIF2* and *ADIR* genes were initially described in present analysis. Third, the tests of protein molecular evolution using relative synonymous codon usage statistics confirmed protein primary structure similarities between eutherian ADIs and tumor necrosis factor ligands. Therefore, the present study proposed revised and standardized classification and nomenclature of eutherian *ADI* genes, as new framework of future analyses.

## Declarations

### Author contribution statement

Marko Premzl: Conceived and designed the experiments; Performed the experiments; Analyzed and interpreted the data; Contributed reagents, materials, analysis tools or data; Wrote the paper.

### Funding statement

This research did not receive any specific grant from funding agencies in the public, commercial, or not-for-profit sectors.

### Competing interest statement

The author declares no conflict of interest.

### Additional information

Data associated with this study has been deposited in the European Nucleotide Archive under the accession numbers LT962964–LT963174 (https://www.ebi.ac.uk/ena/data/view/LT962964-LT963174).

## Appendix A Supplementary data

Supplementary data file 1 Third-party data gene data set of eutherian adiponectin genes. Supplementary data file 1

Supplementary data file 2 Multiple pairwise genomic sequence alignments of eutherian adiponectin genes. The indigo rectangles displayed translated exons in base sequences (top). In each pairwise genomic sequence alignment, the genomic sequence regions including sequence identity levels above empirical cut-offs of detection of common genomic sequence regions were shown accordingly. The rectangles labelled common predicted promoter genomic sequence regions (P). Supplementary data file 2

Supplementary data file 3 Nucleotide sequence alignments of common predicted promoters of eutherian adiponectin genes. The numbers in brackets indicated positions of 3′-terminal nucleotides relative to translation start sites. The nucleotide positions were labelled according to their identity levels using white letters on black background (100% sequence identity level), white letters on dark grey background (≥85% sequence identity level) or black letters on grey background (≥70% sequence identity level). Supplementary data file 3

Supplementary data file 4 Pairwise nucleotide sequence identity patterns of eutherian adiponectin genes. Supplementary data file 4

Supplementary data file 5 Protein sequence alignments of eutherian adiponectins. The amino acid positions were labelled according to their identity levels using white letters on black background (100% sequence identity level), white letters on dark grey background (≥75% sequence identity level) or black letters on grey background (≥50% sequence identity level). In reference human ADIA protein amino acid sequence (top), the 33 invariant amino acid sites were shown using white letters on violet backgrounds and 1 forward amino acid site was shown using white letter on red background. The stop codons were indicated by &s. Supplementary data file 5

Supplementary data file 6 Reference human ADIA amino acid sequence. The 33 invariant amino acid sites were shown using white letters on violet backgrounds and 1 forward amino acid site was shown using white letter on red background. The rectangle displayed low complexity region including 26 tripeptide repeats (G-x(2)). The arrows indicated 6 invariant amino acid sites F124, F142, F160, G166, Y168, F242 and G244 corresponding to invariant amino acid sites H98, W119, L139, G145, Y147, F246 and G247 in human TNLG1B (Premzl, 2016). The secondary structure elements A, A′, B′, B, C, D, E, F, G and H (Gaboriaud et al., 2003) were labelled below reference protein amino acid sequence using grey letters. The black triangle showed predicted N-terminal signal peptide cleavage site. Supplementary data file 6
