# Supplementary material for: Comparative genomic analysis of eutherian adiponectin genes
Source: Heliyon. 2018 Jun 6;4(6):e00647. doi: 10.1016/j.heliyon.2018.e00647 (PMC6040601; doi:10.1016/j.heliyon.2018.e00647)
Supplement: Supplementary data file 1 — Third-party data gene data set of eutherian adiponectin genes. [file mmc1.pdf]

Supplementary data file 1: Third-party data gene data set of eutherian adiponectin genes.

Superordinal clade	Order	Species [a]	Genome assembly	Gene number	Gene [b]	Genomic coordinates [c]	GenBank ID
Euarchontoglires	Primates	Human	GCF_000001405.33	18	*ADIA* (*C1q B*)	exon 1: ch.1: 22659457-22659643 bp<br>exon 2: ch.1: 22660812-22661386 bp	LT962964
					ADIB (*C1q A*)	exon 1: ch.1: 22644024-22644204 bp<br>exon 2: ch.1: 22647227-22647783 bp	LT962965
					ADIC (*C1q C*)	exon 1: ch.1: 22637617-22637779 bp<br>exon 2: ch.1: 22638833-22639407 bp	LT962966
					ADID (*ACRP30*)	exon 1: ch.3: 186852966-186853272 bp<br>exon 2: ch.3: 186854184-186854704 bp	LT962967
					ADIE (*CTRP5*)	exon 1: ch.11: 119340184-119340397 bp<br>exon 2: ch.11: 119339331-119339848 bp	LT962968
					ADIF1 (*CTRP9*)	exon 1: ch.13: 24316004-24316169 bp<br>exon 2: ch.13: 24318818-24318880 bp<br>exon 3: ch.13: 24320996-24321768 bp	LT962969
					ADIF2 (-)	exon 1: ch.13: 23896821-23896986 bp<br>exon 2: ch.13: 23894139-23894201 bp<br>exon 3: ch.13: 23891289-23892061 bp	LT962970
					ADIG (*CTRP7*)	exon 1: ch.4: 15435582-15435981 bp<br>exon 2: ch.4: 15442168-15442799 bp	LT962971
					ADIH (*CTRP2*)	exon 1: ch.5: 160354768-160355011 bp<br>exon 2: ch.5: 160349168-160349781 bp	LT962972
					ADII (*CTRP10*)	exon 1: ch.2: 119157586-119158269 bp<br>exon 2: ch.2: 119156802-119156981 bp	LT962973
					ADIJ (*CTRP13*)	exon 1: ch.10: 16520478-16521065 bp<br>exon 2: ch.10: 16514528-16514707 bp	LT962974
					ADIK (*CTRP11*)	exon 1: ch.12: 49335941-49336477 bp<br>exon 2: ch.12: 49333054-49333233 bp	LT962975
					ADIM (*CTRP3*)	exon 1: ch.5: 34043042-34043458 bp<br>exon 2: ch.5: 34035647-34035758 bp<br>exon 3: ch.5: 34033304-34033458 bp<br>exon 4: ch.5: 34028754-34028883 bp	LT962976

						exon 5: ch.5: 34023909-34024008 bp<br>exon 6: ch.5: 34020583-34020742 bp	
					ADIN (*CTRP4*)	exon 1: ch.11: 47589821-47590810 bp	LT962977
					ADIO (*CTRP8*)	exon 1: ch.16: 1094715-1094922 bp<br>exon 2: ch.16: 1093501-1094051 bp	LT962978
					ADIP (*CTRP1*)	exon 1: ch.17: 79043675-79044123 bp<br>exon 2: ch.17: 79046555-79046694 bp<br>exon 3: ch.17: 79047538-79048088 bp	LT962979
					ADIQ (*CTRP6*)	exon 1: ch.22: 37188163-37188213 bp<br>exon 2: ch.22: 37185218-37185455 bp<br>exon 3: ch.22: 37182188-37182735 bp	LT962980
					ADIR (-)	exon 1: ch.12: 30709902-30710378 bp	LT962981
					putative (*ADIL*, *CTRP14*)	exon 1: ch.17: 44967452-44968048 bp<br>exon 2: ch.17: 44960188-44960367 bp	-
		Common chimpanzee	GCF_000001515.7	13	*ADIB*	exon 1: ch.1: 21772362-21772542 bp<br>exon 2: ch.1: 21775562-21776118 bp	LT962982
					ADID	exon 1: ch.3: 190982487-190982793 bp<br>exon 2: ch.3: 190983705-190984225 bp	LT962983
					ADIE	exon 1: ch.11: 119813163-119813376 bp<br>exon 2: ch.11: 119812310-119812827 bp	LT962984
					ADIF1	exon 1: ch.13: 8955489-8955654 bp<br>exon 2: ch.13: 8958282-8958344 bp<br>exon 3: ch.13: 8960472-8961244 bp	LT962985
					ADIG	exon 1: ch.4: 15736574-15736973 bp<br>exon 2: ch.4: 15743168-15743799 bp	LT962986
					ADIH	exon 1: ch.5: 160693085-160693328 bp<br>exon 2: ch.5: 160687486-160688099 bp	LT962987
					ADIJ	exon 1: ch.10: 17316408-17316995 bp<br>exon 2: ch.10: 17310445-17310624 bp	LT962988
					ADIK	exon 1: ch.12: 40573933-40574469 bp<br>exon 2: ch.12: 40577184-40577363 bp	LT962989

					ADIM	exon 1: ch.5: 80235740-80236156 bp<br>exon 2: ch.5: 80243445-80243556 bp<br>exon 3: ch.5: 80245745-80245899 bp<br>exon 4: ch.5: 80250324-80250453 bp<br>exon 5: ch.5: 80255220-80255319 bp<br>exon 6: ch.5: 80258478-80258637 bp	LT962990
					ADIN	exon 1: ch.11: 47860102-47861091 bp	LT962991
					ADIO	exon 1: ch.16: 1245369-1245576 bp<br>exon 2: ch.16: 1244155-1244705 bp	LT962992
					ADIQ	exon 1: ch.22: 23725942-23725992 bp<br>exon 2: ch.22: 23722995-23723232 bp<br>exon 3: ch.22: 23719843-23720390 bp	LT962993
					ADIR	exon 1: ch.12: 60015352-60015828 bp	LT962994
					putative	exon 1: ch.1: 21765958-21766120 bp<br>exon 2: ch.1: 21767172-21767746 bp	-
					putative	exon 1: ch.1: 21787755-21787941 bp<br>exon 2: ch.1: 21789113-21789687 bp	-
					putative	exon 1: ch.2B: 5587185-5587868 bp<br>exon 2: ch.2B: 5586402-5586581 bp	-
					putative	exon 1: ch.17: 12574055-12574651 bp<br>exon 2: ch.17: 12581431-12581610 bp	-
		Western gorilla	GCA_000151905.3	1	*ADIK*	exon 1: CABD030084013.1: 8531-9067 bp<br>exon 2: CABD030084013.1: 11783-11962 bp	LT962995
					putative	exon 1: CABD030001583.1: 39926-40088 bp<br>exon 2: CABD030001583.1: 41139-41713 bp	-
					putative	exon 1: CABD030001583.1: 46349-46529 bp<br>exon 2: CABD030001583.1: 49526-50082 bp	-
					putative	exon 1: CABD030001583.1: 61821-62007 bp<br>exon 2: CABD030001583.1: 63178-63752 bp	-
					putative	exon 1: CABD030015151.1: 57897-58580 bp<br>exon 2: CABD030015151.1: 57114-57293 bp	-
					putative	exon 1: CABD030026103.1: 16174-16480 bp<br>exon 2: CABD030026103.1: 17392-17912 bp	-

					putative	exon 1: CABD030027288.1: 127453-127852 bp<br>exon 2: CABD030027288.1: 134051-134682 bp	-
					putative	exon 1: CABD030035817.1: 17527-18074 bp<br>exon 2: CABD030035817.1: 14947-15086 bp<br>exon 3: CABD030035817.1: 13573-14123 bp	-
					putative	exon 1: CABD030042015.1: 30378-30621 bp<br>exon 2: CABD030042015.1: 24812-25425 bp	-
					putative	exon 1: CABD030078649.1: 12848-13837 bp	-
					putative	exon 1: CABD030081849.1: 20238-20451 bp<br>exon 2: CABD030081849.1: 19385-19902 bp	-
					putative	exon 1: CABD030084837.1: 24454-24930 bp	-
					putative	exon 1: CABD030098317.1: 58520-58727 bp<br>exon 2: CABD030098317.1: 57306-57856 bp	-
					putative	exon 1: CABD030121029.1: 43310-43360 bp<br>exon 2: CABD030121029.1: 40356-40593 bp<br>exon 3: CABD030121029.1: 37335-37882 bp	-
		Sumatran orangutan	GCF_000001545.4	11	*ADIB*	exon 1: ch.1: 207782328-207782508 bp<br>exon 2: ch.1: 207778743-207779299 bp	LT962996
					ADID	exon 1: ch.3: 190641141-190641354 bp<br>exon 2: ch.3: 190642265-190642785 bp	LT962997
					ADIE	exon 1: ch.11: 116290230-116290443 bp<br>exon 2: ch.11: 116289377-116289894 bp	LT962998
					ADIF1	exon 1: ch.13: 23553687-23553852 bp<br>exon 2: ch.13: 23556497-23556559 bp<br>exon 3: ch.13: 23558700-23559472 bp	LT962999
					ADIG	exon 1: ch.4: 15166236-15166635 bp<br>exon 2: ch.4: 15173067-15173698 bp	LT963000
					ADIH	exon 1: ch.5: 162717888-162718131 bp<br>exon 2: ch.5: 162712486-162713099 bp	LT963001
					ADII	exon 1: ch.2B: 15380502-15381185 bp<br>exon 2: ch.2B: 15381788-15381967 bp	LT963002
					ADIM	exon 1: ch.5: 35242435-35242719 bp<br>exon 2: ch.5: 35232785-35232896 bp	LT963003

						exon 3: ch.5: 35230446-35230600 bp<br>exon 4: ch.5: 35225909-35226038 bp<br>exon 5: ch.5: 35220921-35221020 bp<br>exon 6: ch.5: 35217588-35217747 bp	
					ADIN	exon 1: ch.11: 21021966-21022955 bp	LT963004
					ADIQ	exon 1: ch.22: 32313362-32313412 bp<br>exon 2: ch.22: 32310411-32310648 bp<br>exon 3: ch.22: 32307210-32307757 bp	LT963005
					ADIR	exon 1: ch.12: 31907282-31907758 bp	LT963006
					putative	exon 1: ch.16: 1102224-1103148 bp<br>exon 2: ch.16: 1101031-1101581 bp	-
		Northern white-cheeked gibbon	GCF_000146795.2	8	*ADIB*	exon 1: ch.24: 21796341-21796524 bp<br>exon 2: ch.24: 21799709-21800265 bp	LT963007
					ADID	exon 1: ch.11: 111720014-111720317 bp<br>exon 2: ch.11: 111721229-111721749 bp	LT963008
					ADIF1	exon 1: ch.5: 60558180-60558345 bp<br>exon 2: ch.5: 60555445-60555507 bp<br>exon 3: ch.5: 60552553-60553325 bp	LT963009
					ADIH	exon 1: ch.2: 20593931-20594174 bp<br>exon 2: ch.2: 20599427-20600040 bp	LT963010
					ADIM	exon 1: ch.6: 33076412-33076828 bp<br>exon 2: ch.6: 33069020-33069131 bp<br>exon 3: ch.6: 33066678-33066832 bp<br>exon 4: ch.6: 33061877-33062006 bp<br>exon 5: ch.6: 33056725-33056824 bp<br>exon 6: ch.6: 33053403-33053562 bp	LT963011
					ADIN	exon 1: ch.15: 108458389-108459363 bp	LT963012
					ADIQ	exon 1: ch.7B: 13381831-13381881 bp<br>exon 2: ch.7B: 13384588-13384825 bp<br>exon 3: ch.7B: 13387298-13387845 bp	LT963013
					ADIR	exon 1: ch.23: 4617721-4618197 bp	LT963014
					putative	exon 1: ch.14: 89479387-89479541 bp<br>exon 2: ch.14: 89476831-89476970 bp<br>exon 3: ch.14: 89475455-89476005 bp	-

					putative	exon 1: ch.20: 71280459-71280882 bp<br>exon 2: ch.20: 71273347-71273978 bp	-
					putative	exon 1: ch.24: 21789844-21790006 bp<br>exon 2: ch.24: 21791063-21791637 bp	-
					putative	exon 1: ch.24: 21817006-21817192 bp<br>exon 2: ch.24: 21818362-21818936 bp	-
		Rhesus monkey	GCF_000772875.2	9	*ADIC*	exon 1: ch.1: 21411876-21412038 bp<br>exon 2: ch.1: 21413106-21413680 bp	LT963015
					ADID	exon 1: ch.2: 192771933-192772233 bp<br>exon 2: ch.2: 192770491-192771011 bp	LT963016
					ADIF1	exon 1: ch.17: 4377479-4377644 bp<br>exon 2: ch.17: 4380301-4380363 bp<br>exon 3: ch.17: 4382540-4383312 bp	LT963017
					ADIH	exon 1: ch.6: 158551770-158552013 bp<br>exon 2: ch.6: 158546175-158546788 bp	LT963018
					ADII	exon 1: ch.12: 12036169-12036852 bp<br>exon 2: ch.12: 12037460-12037639 bp	LT963019
					ADIN	exon 1: ch.14: 18420010-18421005 bp	LT963020
					ADIO	exon 1: ch.20: 1063026-1063218 bp<br>exon 2: ch.20: 1061811-1062361 bp	LT963021
					ADIQ	exon 1: ch.10: 79165028-79165078 bp<br>exon 2: ch.10: 79162086-79162323 bp<br>exon 3: ch.10: 79158652-79159199 bp	LT963022
					ADIR	exon 1: ch.11: 31945609-31946085 bp	LT963023
					putative	exon 1: ch.1: 21418312-21418492 bp<br>exon 2: ch.1: 21421493-21422049 bp	-
					putative	exon 1: ch.1: 21433673-21433859 bp<br>exon 2: ch.1: 21435028-21435602 bp	-
					putative	exon 1: ch.6: 34732760-34733176 bp<br>exon 2: ch.6: 34725310-34725421 bp<br>exon 3: ch.6: 34722933-34723087 bp<br>exon 4: ch.6: 34718289-34718418 bp<br>exon 5: ch.6: 34713691-34713790 bp<br>exon 6: ch.6: 34710380-34710539 bp	-

					putative	exon 1: ch.9: 16622406-16622993 bp<br>exon 2: ch.9: 16616524-16616703 bp	-
					putative	exon 1: ch.14: 10846732-10847268 bp<br>exon 2: ch.14: 10849989-10850168 bp	-
					putative	exon 1: ch.14: 112205602-112205815 bp<br>exon 2: ch.14: 112204748-112205265 bp	-
					putative	exon 1: ch.16: 50853508-50854104 bp<br>exon 2: ch.16: 50846957-50847136 bp	-
					putative	exon 1: ch.16: 72859355-72859509 bp<br>exon 2: ch.16: 72861937-72862076 bp<br>exon 3: ch.16: 72862900-72863450 bp	-
		Hamadryas baboon	Pham_1.0 (Ensembl)	9	*ADIA*	exon 1: Contig536268_Contig398796: 8240-8426 bp<br>exon 2: Contig536268_Contig398796: 9595-10169 bp	LT963024
					ADIC	exon 1: Contig394130_Contig753207: 133271-133433 bp<br>exon 2: Contig394130_Contig753207: 134501-135075 bp	LT963025
					ADIE	exon 1: Contig513649_Contig12970_Contig382286_Contig272241: 132156-132369 bp<br>exon 2: Contig513649_Contig12970_Contig382286_Contig272241: 132723-133240 bp	LT963026
					ADIF1	exon 1: Contig282615_Contig452042: 47553-47718 bp<br>exon 2: Contig282615_Contig452042: 44856-44918 bp<br>exon 3: Contig282615_Contig452042: 41949-42721 bp	LT963027
					ADIH	exon 1: Contig266382_Contig640290: 46580-46823 bp<br>exon 2: Contig266382_Contig640290: 41217-41830 bp	LT963028
					ADIJ	exon 1: Contig815475_Contig578418: 118938-119525 bp<br>exon 2: Contig815475_Contig578418: 125216-125395 bp	LT963029
					ADIK	exon 1: Contig70043_Contig741826: 60424-60960 bp<br>exon 2: Contig70043_Contig741826: 63652-63831 bp	LT963030
					ADIM	exon 1: Contig439862_Contig429107: 41563-41979 bp<br>exon 2: Contig439862_Contig429107: 49414-49525 bp<br>exon 3: Contig439862_Contig429107: 51745-51899 bp<br>exon 4: Contig439862_Contig429107: 56425-56554 bp<br>exon 5: Contig439862_Contig429107: 61053-61152 bp	LT963031

						exon 6: Contig439862_Contig429107: 64326-64485 bp	
					ADIR	exon 1: Contig808859_Contig420161: 98519-98995 bp	LT963032
					putative	exon 1: Contig394130_Contig753207: 139612-139867 bp<br>exon 2: Contig394130_Contig753207: 143160-143716 bp	-
					putative	exon 1: Contig427210_Contig769011: 45124-46113 bp	-
					putative	exon 1: Contig657972_Contig785207: 14946-15542 bp<br>exon 2: Contig657972_Contig785207: 21512-21691 bp	-
					putative	exon 1: Contig669505_Contig782055: 169281-169932 bp<br>exon 2: Contig669505_Contig782055: 170595-171145 bp	-
					putative	exon 1: Contig777748_Contig790083_Contig605821_Contig280386: 274530-274684 bp<br>exon 2: Contig777748_Contig790083_Contig605821_Contig280386: 277103-277242 bp<br>exon 3: Contig777748_Contig790083_Contig605821_Contig280386: 278064-278614 bp	-
		Common marmoset	GCF_000004665.1	8	*ADIB*	exon 1: ch.7: 56647309-56647486 bp<br>exon 2: ch.7: 56650470-56651026 bp	LT963033
					ADIC	exon 1: ch.7: 56640690-56640852 bp<br>exon 2: ch.7: 56641897-56642471 bp	LT963034
					ADIE	exon 1: ch.11: 16997166-16997379 bp<br>exon 2: ch.11: 16996310-16996827 bp	LT963035
					ADIG	exon 1: ch.3: 175939054-175939411 bp<br>exon 2: ch.3: 175932572-175933203 bp	LT963036
					ADIK	exon 1: ch.9: 38108276-38108812 bp<br>exon 2: ch.9: 38105430-38105609 bp	LT963037
					ADIM	exon 1: ch.2: 169246864-169247250 bp<br>exon 2: ch.2: 169254773-169254884 bp<br>exon 3: ch.2: 169257106-169257260 bp<br>exon 4: ch.2: 169262036-169262165 bp<br>exon 5: ch.2: 169266628-169266727 bp<br>exon 6: ch.2: 169269522-169269681 bp	LT963038

					ADIN	exon 1: ch.11: 110881268-110882257 bp	LT963039
					ADIR	exon 1: ch.9: 36538417-36538893 bp	LT963040
					putative	exon 1: ch.5: 128296377-128296531 bp<br>exon 2: ch.5: 128299441-128299577 bp<br>exon 3: ch.5: 128300382-128300932 bp	-
					putative	exon 1: ch.15: 8450959-8451172 bp<br>exon 2: ch.15: 8452099-8452619 bp	-
		Philippine tarsier	GCF_000164805.1	1	*ADIR*	exon 1: NW_007252902.1: 544718-545194 bp	LT963041
					putative	exon 1: NW_007028781.1: 146132-146161 bp<br>exon 2: NW_007028781.1: 143407-143665 bp<br>exon 3: NW_007028781.1: 140290-140837 bp	-
					putative	exon 1: NW_007067417.1: 274571-274722 bp<br>exon 2: NW_007067417.1: 277687-277826 bp<br>exon 3: NW_007067417.1: 278766-279316 bp	-
					putative	exon 1: NW_007246429.1: 267605-267839 bp<br>exon 2: NW_007246429.1: 268753-269273 bp	-
					putative	exon 1: NW_007247197.1: 43313-43496 bp<br>exon 2: NW_007247197.1: 41547-42121 bp	-
					putative	exon 1: NW_007247197.1: 53181-53361 bp<br>exon 2: NW_007247197.1: 51097-51653 bp	-
					putative	exon 1: NW_007247197.1: 58942-59158 bp<br>exon 2: NW_007247197.1: 57401-57975 bp	-
					putative	exon 1: NW_007248573.1: 330875-331040 bp<br>exon 2: NW_007248573.1: 328262-328324 bp<br>exon 3: NW_007248573.1: 325006-325778 bp	-
					putative	exon 1: NW_007248778.1: 359418-359702 bp<br>exon 2: NW_007248778.1: 343656-343767 bp<br>exon 3: NW_007248778.1: 340866-341020 bp<br>exon 4: NW_007248778.1: 336501-336630 bp<br>exon 5: NW_007248778.1: 328685-328784 bp<br>exon 6: NW_007248778.1: 324323-324482 bp	-
					putative	exon 1: NW_007253531.1: 215568-215811 bp<br>exon 2: NW_007253531.1: 209888-210501 bp	-
					putative	exon 1: NW_007253579.1: 75274-75619 bp	-

						exon 2: NW_007253579.1: 67225-67856 bp	
		Gray mouse lemur	GCF_000165445.1	1	*ADIF1*	exon 1: NW_012199119.1: 191118-191283 bp<br>exon 2: NW_012199119.1: 190335-190397 bp<br>exon 3: NW_012199119.1: 188273-189042 bp	LT963042
					putative	exon 1: NW_012195041.1: 7101258-7101794 bp<br>exon 2: NW_012195041.1: 7098619-7098798 bp	-
					putative	exon 1: NW_012195453.1: 492680-493660 bp	-
					putative	exon 1: NW_012195531.1: 2036797-2037273 bp	-
					putative	exon 1: NW_012195927.1: 178407-179090 bp<br>exon 2: NW_012195927.1: 177667-177846 bp	-
					putative	exon 1: NW_012196186.1: 93768-93948 bp<br>exon 2: NW_012196186.1: 92121-92695 bp	-
					putative	exon 1: NW_012196186.1: 117769-118279 bp<br>exon 2: NW_012196186.1: 116155-116729 bp	-
					putative	exon 1: NW_012196587.1: 1732194-1732407 bp<br>exon 2: NW_012196587.1: 1732747-1733264 bp	-
					putative	exon 1: NW_012196877.1: 1481-2164 bp<br>exon 2: NW_012196877.1: 741-920 bp	-
					putative	exon 1: NW_012198142.1: 532753-532996 bp<br>exon 2: NW_012198142.1: 536402-537015 bp	-
					putative	exon 1: NW_012198186.1: 1459850-1460446 bp<br>exon 2: NW_012198186.1: 1453877-1454056 bp	-
					putative	exon 1: NW_012198519.1: 1497971-1498211 bp<br>exon 2: NW_012198519.1: 1496512-1497032 bp	-
					putative	exon 1: NW_012199241.1: 9434739-9435045 bp<br>exon 2: NW_012199241.1: 9428365-9428996 bp	-
					putative	exon 1: NW_012200463.1: 110485-110692 bp<br>exon 2: NW_012200463.1: 109379-109929 bp	-
					putative	exon 1: NW_012202819.1: 309422-310009 bp<br>exon 2: NW_012202819.1: 303525-303704 bp	-
		Northern greater galago	GCF_000181295.1	7	*ADIB*	exon 1: NW_003852404.1: 2852377-2852551 bp<br>exon 2: NW_003852404.1: 2848833-2849389 bp	LT963043

					ADID	exon 1: NW_003852455.1: 9801346-9801559 bp<br>exon 2: NW_003852455.1: 9799916-9800436 bp	LT963044
					ADIE	exon 1: NW_003852503.1: 5336053-5336266 bp<br>exon 2: NW_003852503.1: 5336640-5337157 bp	LT963045
					ADIF1	exon 1: NW_003852501.1: 6605131-6605296 bp<br>exon 2: NW_003852501.1: 6608416-6608478 bp<br>exon 3: NW_003852501.1: 6610069-6610838 bp	LT963046
					ADIG	exon 1: NW_003852623.1: 1761915-1762152 bp<br>exon 2: NW_003852623.1: 1766108-1766739 bp	LT963047
					ADIP	exon 1: NW_003852413.1: 3418595-3418749 bp<br>exon 2: NW_003852413.1: 3416408-3416547 bp<br>exon 3: NW_003852413.1: 3415190-3415740 bp	LT963048
					ADIR	exon 1: NW_003852452.1: 9661612-9662088 bp	LT963049
					putative	exon 1: NW_003852396.1: 72742913-72743893 bp	-
					putative	exon 1: NW_003852398.1: 16246700-16246943 bp<br>exon 2: NW_003852398.1: 16252510-16253123 bp	-
					putative	exon 1: NW_003852404.1: 2839034-2839214 bp<br>exon 2: NW_003852404.1: 2837410-2837984 bp	-
					putative	exon 1: NW_003852404.1: 2857843-2858059 bp<br>exon 2: NW_003852404.1: 2856195-2856769 bp	-
					putative	exon 1: NW_003852413.1: 19527487-19528083 bp<br>exon 2: NW_003852413.1: 19534101-19534280 bp	-
					putative	exon 1: NW_003852414.1: 16482751-16483338 bp<br>exon 2: NW_003852414.1: 16488972-16489151 bp	-
					putative	exon 1: NW_003852439.1: 7285081-7285291 bp<br>exon 2: NW_003852439.1: 7283910-7284460 bp	-
					putative	exon 1: NW_003852454.1: 4649543-4650229 bp<br>exon 2: NW_003852454.1: 4648787-4648966 bp	-
					putative	exon 1: NW_003852458.1: 2984699-2984782 bp<br>exon 2: NW_003852458.1: 2978204-2978315 bp<br>exon 3: NW_003852458.1: 2975497-2975651 bp<br>exon 4: NW_003852458.1: 2971780-2971909 bp<br>exon 5: NW_003852458.1: 2961621-2961720 bp<br>exon 6: NW_003852458.1: 2958934-2959093 bp	-

					putative	exon 1: NW_003852465.1: 9578676-9579212 bp<br>exon 2: NW_003852465.1: 9576391-9576570 bp	-
	Scadentia	Northern treeshrew	tupBel1 (Ensembl)	1	*ADIR*	exon 1: GeneScaffold_1329: 27569-28045 bp	LT963050
					putative	exon 1: GeneScaffold_2431: 128907-129150 bp<br>exon 2: GeneScaffold_2431: 124347-124960 bp	-
					putative	exon 1: GeneScaffold_3260: 457931-458215 bp<br>exon 2: GeneScaffold_3260: 449254-449365 bp<br>exon 3: GeneScaffold_3260: 445938-446092 bp<br>exon 4: GeneScaffold_3260: 442363-442492 bp<br>exon 5: GeneScaffold_3260: 433105-433204 bp<br>exon 6: GeneScaffold_3260: 431338-431497 bp	-
					putative	exon 1: GeneScaffold_4967: 169424-169589 bp<br>exon 2: GeneScaffold_4967: 172701-172763 bp<br>exon 3: GeneScaffold_4967: 174610-175379 bp	-
	Rodentia	Mouse	GCF_000001635.24	16	*Adia*	exon 1: ch.4: 136882127-136882394 bp<br>exon 2: ch.4: 136880289-136880869 bp	LT963051
					Adib	exon 1: ch.4: 136892379-136892652 bp<br>exon 2: ch.4: 136890043-136890599 bp	LT963052
					Adic	exon 1: ch.4: 136897675-136897837 bp<br>exon 2: ch.4: 136896154-136896728 bp	LT963053
					Adid	exon 1: ch.16: 23155192-23155447 bp<br>exon 2: ch.16: 23157074-23157594 bp	LT963054
					Adie	exon 1: ch.9: 44107758-44107971 bp<br>exon 2: ch.9: 44108295-44108812 bp	LT963055
					Adif1	exon 1: ch.14: 60772210-60772462 bp<br>exon 2: ch.14: 60777025-60777087 bp<br>exon 3: ch.14: 60779252-60780024 bp	LT963056
					Adig	exon 1: ch.5: 43608935-43609298 bp<br>exon 2: ch.5: 43615620-43616251 bp	LT963057
					Adih	exon 1: ch.11: 43485809-43486073 bp<br>exon 2: ch.11: 43490724-43491337 bp	LT963058
					Adii	exon 1: ch.1: 120341118-120341801 bp<br>exon 2: ch.1: 120342395-120342574 bp	LT963059
					Adij	exon 1: ch.2: 13010260-13010847 bp	LT963060

						exon 2: ch.2: 13004265-13004444 bp	
					Adil	exon 1: ch.11: 102945865-102946461 bp<br>exon 2: ch.11: 102939733-102939912 bp	LT963061
					Adim	exon 1: ch.15: 10952454-10952537 bp<br>exon 2: ch.15: 10957957-10958068 bp<br>exon 3: ch.15: 10960628-10960782 bp<br>exon 4: ch.15: 10971981-10972110 bp<br>exon 5: ch.15: 10975591-10975690 bp<br>exon 6: ch.15: 10978516-10978675 bp	LT963062
					Adin	exon 1: ch.2: 90889385-90890365 bp	LT963063
					Adip	exon 1: ch.11: 118443696-118443850 bp<br>exon 2: ch.11: 118446501-118446640 bp<br>exon 3: ch.11: 118447801-118448351 bp	LT963064
					Adiq	exon 1: ch.15: 78529518-78529523 bp<br>exon 2: ch.15: 78527228-78527468 bp<br>exon 3: ch.15: 78524851-78525398 bp	LT963065
					Adir	exon 1: ch.6: 148842830-148843306 bp	LT963066
					putative	exon 1: ch.15: 99087192-99087728 bp<br>exon 2: ch.15: 99084754-99084933 bp	-
		Brown rat	GCF_000001895.5	15	*Adia*	exon 1: ch.5: 155248434-155248614 bp<br>exon 2: ch.5: 155246590-155247170 bp	LT963067
					Adib	exon 1: ch.5: 155257872-155258121 bp<br>exon 2: ch.5: 155255291-155255847 bp	LT963068
					Adic	exon 1: ch.5: 155262993-155263191 bp<br>exon 2: ch.5: 155261481-155262055 bp	LT963069
					Adid	exon 1: ch.11: 81333897-81334110 bp<br>exon 2: ch.11: 81331512-81332032 bp	LT963070
					Adie	exon 1: ch.8: 48444243-48444456 bp<br>exon 2: ch.8: 48444748-48445265 bp	LT963071
					Adif1	exon 1: ch.15: 41074274-41074487 bp<br>exon 2: ch.15: 41077983-41078045 bp<br>exon 3: ch.15: 41079993-41080765 bp	LT963072
					Adig	exon 1: ch.14: 72017893-72018259 bp<br>exon 2: ch.14: 72011915-72012546 bp	LT963073

					Adih	exon 1: ch.10: 29080125-29080500 bp<br>exon 2: ch.10: 29083229-29083842 bp	LT963074
					Adii	exon 1: ch.13: 36378276-36378959 bp<br>exon 2: ch.13: 36379543-36379722 bp	LT963075
					Adij	exon 1: ch.17: 80320094-80320681 bp<br>exon 2: ch.17: 80314186-80314365 bp	LT963076
					Adik	exon 1: ch.7: 140770111-140770647 bp<br>exon 2: ch.7: 140767737-140767916 bp	LT963077
					Adil	exon 1: ch.10: 91046581-91047177 bp<br>exon 2: ch.10: 91040464-91040643 bp	LT963078
					Adim	exon 1: ch.2: 60920101-60920406 bp<br>exon 2: ch.2: 60926818-60926929 bp<br>exon 3: ch.2: 60929393-60929547 bp<br>exon 4: ch.2: 60933456-60933585 bp<br>exon 5: ch.2: 60938601-60938700 bp<br>exon 6: ch.2: 60941447-60941606 bp	LT963079
					Adiq	exon 1: ch.7: 119752599-119752604 bp<br>exon 2: ch.7: 119749929-119750166 bp<br>exon 3: ch.7: 119747606-119748153 bp	LT963080
					Adir	exon 1: ch.4: 183305813-183306289 bp	LT963081
					putative	exon 1: ch.10: 107471079-107471233 bp<br>exon 2: ch.10: 107473859-107473998 bp<br>exon 3: ch.10: 107475069-107475619 bp	-
		Ord's kangaroo rat	GCF_000151885.1	1	*ADIH*	exon 1: NW_012267328.1: 6009981-6010224 bp<br>exon 2: NW_012267328.1: 6010640-6011253 bp	LT963082
					putative	exon 1: NW_012267217.1: 30954311-30954476 bp<br>exon 2: NW_012267217.1: 30952553-30952615 bp<br>exon 3: NW_012267217.1: 30949920-30950692 bp	-
					putative	exon 1: NW_012267222.1: 14964043-14964391 bp<br>exon 2: NW_012267222.1: 14961939-14962513 bp	-
					putative	exon 1: NW_012267222.1: 14972738-14972918 bp<br>exon 2: NW_012267222.1: 14970069-14970625 bp	-
					putative	exon 1: NW_012267222.1: 14978021-14978297 bp<br>exon 2: NW_012267222.1: 14976568-14977151 bp	-

					putative	exon 1: NW_012267223.1: 1593430-1593782 bp<br>exon 2: NW_012267223.1: 1591217-1591356 bp<br>exon 3: NW_012267223.1: 1590051-1590601 bp	-
					putative	exon 1: NW_012267224.1: 3157575-3157845 bp<br>exon 2: NW_012267224.1: 3163338-3163969 bp	-
					putative	exon 1: NW_012267240.1: 14784648-14785235 bp<br>exon 2: NW_012267240.1: 14790940-14791119 bp	-
					putative	exon 1: NW_012267258.1: 2790384-2790920 bp<br>exon 2: NW_012267258.1: 2793528-2793707 bp	-
					putative	exon 1: NW_012267383.1: 1312731-1313207 bp	-
		Domesticated guinea pig	GCF_000151735.1	12	*ADIA*	exon 1: NT_176394.1: 12690814-12690994 bp<br>exon 2: NT_176394.1: 12689187-12689761 bp	LT963083
					ADIB	exon 1: NT_176394.1: 12700328-12700649 bp<br>exon 2: NT_176394.1: 12697351-12697922 bp	LT963084
					ADIC	exon 1: NT_176394.1: 12705093-12705252 bp<br>exon 2: NT_176394.1: 12703578-12704152 bp	LT963085
					ADID	exon 1: NT_176412.1: 51777678-51778017 bp<br>exon 2: NT_176412.1: 51779495-51780024 bp	LT963086
					ADIF1	exon 1: NT_176413.1: 26701563-26701728 bp<br>exon 2: NT_176413.1: 26697826-26697888 bp<br>exon 3: NT_176413.1: 26695165-26695937 bp	LT963087
					ADIG	exon 1: NT_176334.1: 6876849-6877086 bp<br>exon 2: NT_176334.1: 6882361-6882992 bp	LT963088
					ADIH	exon 1: NT_176402.1: 7226151-7226394 bp<br>exon 2: NT_176402.1: 7220740-7221353 bp	LT963089
					ADIK	exon 1: NT_176410.1: 50268360-50268896 bp<br>exon 2: NT_176410.1: 50271195-50271374 bp	LT963090
					ADIM	exon 1: NT_176390.1: 21558336-21558617 bp<br>exon 2: NT_176390.1: 21564226-21564340 bp<br>exon 3: NT_176390.1: 21567256-21567410 bp<br>exon 4: NT_176390.1: 21569649-21569778 bp<br>exon 5: NT_176390.1: 21571834-21571933 bp<br>exon 6: NT_176390.1: 21575288-21575447 bp	LT963091
					ADIN	exon 1: NT_176347.1: 6807750-6808751 bp	LT963092

					ADIP	exon 1: NT_176343.1: 3959007-3959161 bp<br>exon 2: NT_176343.1: 3961576-3961715 bp<br>exon 3: NT_176343.1: 3962396-3962946 bp	LT963093
					ADIR	exon 1: NT_176365.1: 5113941-5114417 bp	LT963094
					putative	exon 1: NT_176400.1: 28777680-28778100 bp<br>exon 2: NT_176400.1: 28778527-28779044 bp	-
					putative	exon 1: NT_176416.1: 59889501-59890184 bp<br>exon 2: NT_176416.1: 59888723-59888902 bp	-
		Thirteen-lined ground squirrel	GCF_000236235.1	0	putative	exon 1: NW_004936469.1: 51198722-51199405 bp<br>exon 2: NW_004936469.1: 51199994-51200173 bp	-
					putative	exon 1: NW_004936474.1: 8013686-8013866 bp<br>exon 2: NW_004936474.1: 8016202-8016758 bp	-
					putative	exon 1: NW_004936474.1: 8022398-8022740 bp<br>exon 2: NW_004936474.1: 8023718-8024292 bp	-
					putative	exon 1: NW_004936477.1: 12445404-12445881 bp<br>exon 2: NW_004936477.1: 12438780-12439411 bp	-
					putative	exon 1: NW_004936501.1: 53175-53418 bp<br>exon 2: NW_004936501.1: 53841-54391 bp	-
					putative	exon 1: NW_004936512.1: 7090076-7090612 bp<br>exon 2: NW_004936512.1: 7087720-7087899 bp	-
					putative	exon 1: NW_004936515.1: 3223370-3223613 bp<br>exon 2: NW_004936515.1: 3227197-3227810 bp	-
					putative	exon 1: NW_004936518.1: 7039733-7039816 bp<br>exon 2: NW_004936518.1: 7045402-7045513 bp<br>exon 3: NW_004936518.1: 7047933-7048087 bp<br>exon 4: NW_004936518.1: 7050753-7050882 bp<br>exon 5: NW_004936518.1: 7055362-7055461 bp<br>exon 6: NW_004936518.1: 7058393-7058552 bp	-
					putative	exon 1: NW_004936520.1: 10657374-10657961 bp<br>exon 2: NW_004936520.1: 10663722-10663901 bp	-
					putative	exon 1: NW_004936542.1: 4270882-4271236 bp<br>exon 2: NW_004936542.1: 4270015-4270532 bp	-
					putative	exon 1: NW_004936562.1: 1662011-1662997 bp	-

					putative	exon 1: NW_004936578.1: 3171330-3171564 bp<br>exon 2: NW_004936578.1: 3169929-3170449 bp	-
					putative	exon 1: NW_004936607.1: 4995106-4995582 bp	-
					putative	exon 1: NW_004936688.1: 1580665-1580830 bp<br>exon 2: NW_004936688.1: 1582861-1582923 bp<br>exon 3: NW_004936688.1: 1584266-1585038 bp	-
	Lagomorpha	European rabbit	GCF_000003625.3	7	*ADIB*	exon 1: ch.13: 130487628-130487967 bp<br>exon 2: ch.13: 130485282-130485838 bp	LT963095
					ADIC	exon 1: ch.13: 130492669-130492954 bp<br>exon 2: ch.13: 130491485-130492059 bp	LT963096
					ADID	exon 1: ch.14: 82543231-82543474 bp<br>exon 2: ch.14: 82544298-82544818 bp	LT963097
					ADIF1	exon 1: ch.8: 40855395-40855560 bp<br>exon 2: ch.8: 40858585-40858647 bp<br>exon 3: ch.8: 40860190-40860959 bp	LT963098
					ADIG	exon 1: ch.2: 5919966-5920203 bp<br>exon 2: ch.2: 5926436-5927067 bp	LT963099
					ADIJ	exon 1: ch.16: 44773805-44774392 bp<br>exon 2: ch.16: 44779984-44780163 bp	LT963100
					ADIR	exon 1: ch.8: 9902521-9902997 bp	LT963101
					putative	exon 1: ch.4: 33580669-33581205 bp<br>exon 2: ch.4: 33578449-33578628 bp	-
					putative	exon 1: ch.11: 55758541-55758624 bp<br>exon 2: ch.11: 55750957-55751068 bp<br>exon 3: ch.11: 55748026-55748180 bp<br>exon 4: ch.11: 55743482-55743611 bp<br>exon 5: ch.11: 55738305-55738404 bp<br>exon 6: ch.11: 55735353-55735512 bp	-
					putative	exon 1: ch.13: 130477287-130477467 bp<br>exon 2: ch.13: 130475617-130476185 bp	-
					putative	exon 1: ch.19: 45347659-45348186 bp<br>exon 2: ch.19: 45353597-45353776 bp	-
					putative	exon 1: NW_003159449.1: 796200-796413 bp<br>exon 2: NW_003159449.1: 795385-795902 bp	-

		American pika	GCF_000292845.1	2	*ADIA*	exon 1: NW_004535487.1: 5396612-5396792 bp<br>exon 2: NW_004535487.1: 5394995-5395569 bp	LT963102
					ADIF1	exon 1: NW_004535437.1: 69713749-69713914 bp<br>exon 2: NW_004535437.1: 69720285-69720347 bp<br>exon 3: NW_004535437.1: 69721585-69722354 bp	LT963103
					putative	exon 1: NW_004535438.1: 2952559-2952802 bp<br>exon 2: NW_004535438.1: 2953585-2954105 bp	-
					putative	exon 1: NW_004535439.1: 23772643-23773230 bp<br>exon 2: NW_004535439.1: 23778885-23779064 bp	-
					putative	exon 1: NW_004535440.1: 47275841-47276117 bp<br>exon 2: NW_004535440.1: 47269552-47270183 bp	-
					putative	exon 1: NW_004535452.1: 13450528-13450611 bp<br>exon 2: NW_004535452.1: 13444112-13444223 bp<br>exon 3: NW_004535452.1: 13441373-13441527 bp<br>exon 4: NW_004535452.1: 13435103-13435232 bp<br>exon 5: NW_004535452.1: 13430630-13430729 bp<br>exon 6: NW_004535452.1: 13429796-13429955 bp	-
					putative	exon 1: NW_004535456.1: 26366725-26367070 bp<br>exon 2: NW_004535456.1: 26365874-26366391 bp	-
					putative	exon 1: NW_004535457.1: 5398293-5399381 bp	-
					putative	exon 1: NW_004535463.1: 14705860-14706244 bp<br>exon 2: NW_004535463.1: 14708985-14709598 bp	-
					putative	exon 1: NW_004535473.1: 5849688-5850371 bp<br>exon 2: NW_004535473.1: 5850944-5851123 bp	-
					putative	exon 1: NW_004535487.1: 5404303-5404543 bp<br>exon 2: NW_004535487.1: 5403060-5403616 bp	-
					putative	exon 1: NW_004535487.1: 5409074-5409392 bp<br>exon 2: NW_004535487.1: 5407721-5408295 bp	-
					putative	exon 1: NW_004535492.1: 2199029-2199351 bp<br>exon 2: NW_004535492.1: 2197761-2197897 bp<br>exon 3: NW_004535492.1: 2196714-2197264 bp	-
					putative	exon 1: NW_004535524.1: 2138962-2139558 bp<br>exon 2: NW_004535524.1: 2144720-2144899 bp	-
					putative	exon 1: NW_004535573.1: 1634599-1635129 bp	-

						exon 2: NW_004535573.1: 1637137-1637316 bp	
					putative	exon 1: NW_004535579.1: 745647-746123 bp	-
Laurasiatheria	Cetacea	Bottlenose dolphin	GCF_000151865.1	3	*ADIG*	exon 1: NW_004200275.1: 42944-43337 bp<br>exon 2: NW_004200275.1: 36646-37277 bp	LT963104
					ADIN	exon 1: NW_004198200.1: 31355-32329 bp	LT963105
					ADIR	exon 1: NW_004199138.1: 41569-42045 bp	LT963106
					putative	exon 1: NW_004198978.1: 86422-86629 bp<br>exon 2: NW_004198978.1: 84770-85344 bp	-
					putative	exon 1: NW_004202650.1: 80603-80768 bp<br>exon 2: NW_004202650.1: 76047-76109 bp<br>exon 3: NW_004202650.1: 73819-74591 bp	-
					putative	exon 1: NW_004203481.1: 100982-101316 bp<br>exon 2: NW_004203481.1: 103624-103763 bp<br>exon 3: NW_004203481.1: 104340-104890 bp	-
					putative	exon 1: NW_004205038.1: 98957-99040 bp<br>exon 2: NW_004205038.1: 92379-92490 bp<br>exon 3: NW_004205038.1: 90132-90286 bp<br>exon 4: NW_004205038.1: 84587-84716 bp<br>exon 5: NW_004205038.1: 76692-76791 bp<br>exon 6: NW_004205038.1: 74710-74869 bp	-
					putative	exon 1: NW_004218567.1: 32386-32593 bp<br>exon 2: NW_004218567.1: 30950-31470 bp	-
					putative	exon 1: NW_004224891.1: 24831-25074 bp<br>exon 2: NW_004224891.1: 20414-21027 bp	-
	Artiodactyla	Domestic cattle	GCF_000003205.7	12	*ADIA*	exon 1: ch.2: 131195396-131195564 bp<br>exon 2: ch.2: 131193598-131194172 bp	LT963107
					ADIB	exon 1: ch.2: 131212102-131212276 bp<br>exon 2: ch.2: 131208518-131209074 bp	LT963108
					ADID	exon 1: ch.1: 81296851-81297049 bp<br>exon 2: ch.1: 81295430-81295953 bp	LT963109
					ADIF1	exon 1: ch.12: 34643982-34644147 bp<br>exon 2: ch.12: 34645029-34645091 bp<br>exon 3: ch.12: 34646475-34647247 bp	LT963110

					ADIG	exon 1: ch.6: 115838168-115838405 bp<br>exon 2: ch.6: 115845022-115845653 bp	LT963111
					ADIH	exon 1: ch.7: 74382726-74383020 bp<br>exon 2: ch.7: 74379560-74380173 bp	LT963112
					ADII	exon 1: ch.2: 71533535-71534218 bp<br>exon 2: ch.2: 71532772-71532951 bp	LT963113
					ADIJ	exon 1: ch.13: 31385835-31386422 bp<br>exon 2: ch.13: 31379854-31380033 bp	LT963114
					ADIM	exon 1: ch.20: 39892816-39892899 bp<br>exon 2: ch.20: 39902210-39902321 bp<br>exon 3: ch.20: 39904660-39904814 bp<br>exon 4: ch.20: 39910391-39910520 bp<br>exon 5: ch.20: 39921017-39921116 bp<br>exon 6: ch.20: 39922962-39923121 bp	LT963115
					ADIO	exon 1: ch.25: 868538-868742 bp<br>exon 2: ch.25: 867383-867933 bp	LT963116
					ADIP	exon 1: ch.19: 54206849-54207072 bp<br>exon 2: ch.19: 54203236-54203375 bp<br>exon 3: ch.19: 54202112-54202662 bp	LT963117
					ADIR	exon 1: ch.5: 79471366-79471842 bp	LT963118
					putative	exon 1: ch.2: 131218610-131218874 bp<br>exon 2: ch.2: 131217013-131217584 bp	-
					putative	exon 1: ch.15: 30626859-30627072 bp<br>exon 2: ch.15: 30625998-30626515 bp	-
					putative	exon 1: ch.15: 78970223-78971194 bp	-
					putative	exon 1: ch.19: 45484164-45484760 bp<br>exon 2: ch.19: 45478136-45478315 bp	-
		Wild boar	GCF_000003025.5	0	putative	exon 1: ch.2: 16202335-16203297 bp	-
					putative	exon 1: ch.5: 15717616-15718152 bp<br>exon 2: ch.5: 15715418-15715597 bp	-
					putative	exon 1: ch.5: 46881786-46882262 bp	-
					putative	exon 1: ch.6: 74610905-74611118 bp<br>exon 2: ch.6: 74612209-74612783 bp	-

					putative	exon 1: ch.6: 74616876-74617056 bp<br>exon 2: ch.6: 74620391-74620947 bp	-
					putative	exon 1: ch.9: 51583667-51584021 bp<br>exon 2: ch.9: 51582794-51583311 bp	-
					putative	exon 1: ch.11: 2150998-2151163 bp<br>exon 2: ch.11: 2148956-2149018 bp<br>exon 3: ch.11: 2146137-2146909 bp	-
					putative	exon 1: ch.15: 28312626-28313309 bp<br>exon 2: ch.15: 28311838-28312017 bp	-
					putative	exon 1: ch.16: 68554324-68554567 bp<br>exon 2: ch.16: 68557187-68557800 bp	-
		Vicugna	GCF_000164845.1	0	putative	exon 1: NW_005882704.1: 15886219-15886426 bp<br>exon 2: NW_005882704.1: 15887495-15888069 bp	-
					putative	exon 1: NW_005882704.1: 15892001-15892178 bp<br>exon 2: NW_005882704.1: 15895192-15895748 bp	-
					putative	exon 1: NW_005882704.1: 15903231-15903411 bp<br>exon 2: NW_005882704.1: 15904587-15905161 bp	-
					putative	exon 1: NW_005882717.1: 13720789-13720937 bp<br>exon 2: NW_005882717.1: 13723420-13723559 bp<br>exon 3: NW_005882717.1: 13724073-13724623 bp	-
					putative	exon 1: NW_005882720.1: 13567005-13567481 bp	-
					putative	exon 1: NW_005882727.1: 9014209-9014419 bp<br>exon 2: NW_005882727.1: 9013080-9013600 bp	-
					putative	exon 1: NW_005882745.1: 8499404-8499785 bp<br>exon 2: NW_005882745.1: 8498070-8498620 bp	-
					putative	exon 1: NW_005882845.1: 4068269-4068512 bp<br>exon 2: NW_005882845.1: 4063055-4063668 bp	-
					putative	exon 1: NW_005882946.1: 328626-328791 bp<br>exon 2: NW_005882946.1: 332054-332116 bp<br>exon 3: NW_005882946.1: 333406-334178 bp	-
	Perissodactyla	Horse	GCF_000002305.2	11	*ADIA*	exon 1: ch.2: 32051999-32052182 bp<br>exon 2: ch.2: 32050237-32050811 bp	LT963119
					ADIB	exon 1: ch.2: 32064219-32064453 bp	LT963120

						exon 2: ch.2: 32060661-32061217 bp	
					ADIC	exon 1: ch.2: 32070056-32070317 bp<br>exon 2: ch.2: 32068429-32068997 bp	LT963121
					ADID	exon 1: ch.19: 24845819-24846203 bp<br>exon 2: ch.19: 24847074-24847594 bp	LT963122
					ADIF1	exon 1: ch.17: 4547911-4548076 bp<br>exon 2: ch.17: 4543115-4543177 bp<br>exon 3: ch.17: 4540763-4541535 bp	LT963123
					ADIG	exon 1: ch.3: 107936926-107937286 bp<br>exon 2: ch.3: 107930536-107931167 bp	LT963124
					ADIH	exon 1: ch.14: 19372017-19372260 bp<br>exon 2: ch.14: 19375774-19376387 bp	LT963125
					ADIK	exon 1: ch.6: 67077159-67077695 bp<br>exon 2: ch.6: 67074797-67074976 bp	LT963126
					ADIM	exon 1: ch.21: 30605922-30606005 bp<br>exon 2: ch.21: 30611805-30611916 bp<br>exon 3: ch.21: 30614230-30614384 bp<br>exon 4: ch.21: 30617387-30617516 bp<br>exon 5: ch.21: 30627461-30627560 bp<br>exon 6: ch.21: 30630141-30630300 bp	LT963127
					ADIP	exon 1: ch.11: 3730832-3730980 bp<br>exon 2: ch.11: 3728042-3728181 bp<br>exon 3: ch.11: 3726870-3727420 bp	LT963128
					ADIR	exon 1: ch.6: 55400208-55400684 bp	LT963129
	Carnivora	Domestic dog	GCF_000002285.3	13	*ADIA*	exon 1: ch.2: 76652329-76652506 bp<br>exon 2: ch.2: 76650586-76651160 bp	LT963130
					ADIB	exon 1: ch.2: 76664003-76664183 bp<br>exon 2: ch.2: 76660318-76660874 bp	LT963131
					ADIC	exon 1: ch.2: 76669924-76670143 bp<br>exon 2: ch.2: 76668299-76668873 bp	LT963132
					ADID	exon 1: ch.34: 19406584-19406824 bp<br>exon 2: ch.34: 19407910-19408430 bp	LT963133
					ADIF1	exon 1: ch.25: 14740828-14740993 bp<br>exon 2: ch.25: 14745563-14745625 bp	LT963134

						exon 3: ch.25: 14746980-14747752 bp	
					ADIG	exon 1: ch.3: 64898323-64898683 bp<br>exon 2: ch.3: 64891589-64892220 bp	LT963135
					ADIH	exon 1: ch.4: 50367919-50368162 bp<br>exon 2: ch.4: 50371634-50372247 bp	LT963136
					ADII	exon 1: ch.19: 30863214-30863897 bp<br>exon 2: ch.19: 30864537-30864716 bp	LT963137
					ADIJ	exon 1: ch.2: 20301087-20301674 bp<br>exon 2: ch.2: 20307424-20307603 bp	LT963138
					ADIK	exon 1: ch.27: 5278360-5278896 bp<br>exon 2: ch.27: 5280948-5281127 bp	LT963139
					ADIM	exon 1: ch.4: 73787646-73787912 bp<br>exon 2: ch.4: 73793904-73794015 bp<br>exon 3: ch.4: 73796890-73797044 bp<br>exon 4: ch.4: 73800936-73801065 bp<br>exon 5: ch.4: 73805546-73805645 bp<br>exon 6: ch.4: 73808765-73808924 bp	LT963140
					ADIN	exon 1: ch.18: 42064308-42065282 bp	LT963141
					ADIR	exon 1: ch.27: 17560035-17560511 bp	LT963142
		Domestic cat	GCF_000181335.2	3	*ADIA*	exon 1: ch.C1: 16710786-16710975 bp<br>exon 2: ch.C1: 16712128-16712702 bp	LT963143
					ADID	exon 1: ch.C2: 80902008-80902221 bp<br>exon 2: ch.C2: 80900615-80901135 bp	LT963144
					ADIF1	exon 1: ch.A1: 4456976-4457141 bp<br>exon 2: ch.A1: 4453827-4453889 bp<br>exon 3: ch.A1: 4451336-4452105 bp	LT963145
					putative	exon 1: ch.A1: 188430545-188430788 bp<br>exon 2: ch.A1: 188434730-188435343 bp	-
					putative	exon 1: ch.A1: 212701142-212701225 bp<br>exon 2: ch.A1: 212707336-212707447 bp<br>exon 3: ch.A1: 212709705-212709859 bp<br>exon 4: ch.A1: 212714598-212714727 bp<br>exon 5: ch.A1: 212720016-212720115 bp<br>exon 6: ch.A1: 212723602-212723761 bp	-

					putative	exon 1: ch.B1: 194971722-194972022 bp<br>exon 2: ch.B1: 194964128-194964759 bp	-
					putative	exon 1: ch.B4: 63536459-63536935 bp	-
					putative	exon 1: ch.C1: 16693469-16693631 bp<br>exon 2: ch.C1: 16694911-16695485 bp	-
					putative	exon 1: ch.E3: 40286464-40286983 bp<br>exon 2: ch.E3: 40287472-40288022 bp	-
	Chiroptera	Little brown myotis	GCF_000147115.1	7	*ADIA*	exon 1: NW_005871176.1: 2671063-2671243 bp<br>exon 2: NW_005871176.1: 2672355-2672929 bp	LT963146
					ADIB	exon 1: NW_005871176.1: 2658536-2658716 bp<br>exon 2: NW_005871176.1: 2661684-2662240 bp	LT963147
					ADIC	exon 1: NW_005871176.1: 2652908-2653070 bp<br>exon 2: NW_005871176.1: 2654072-2654646 bp	LT963148
					ADID	exon 1: NW_005871062.1: 7889513-7889753 bp<br>exon 2: NW_005871062.1: 7888083-7888603 bp	LT963149
					ADIG	exon 1: NW_005871415.1: 219436-219673 bp<br>exon 2: NW_005871415.1: 229303-229934 bp	LT963150
					ADIO	exon 1: NW_005871670.1: 97380-97584 bp<br>exon 2: NW_005871670.1: 98264-98814 bp	LT963151
					ADIR	exon 1: NW_005871078.1: 7195740-7196216 bp	LT963152
					putative	exon 1: NW_005871104.1: 143002-143392 bp<br>exon 2: NW_005871104.1: 144443-145056 bp	-
					putative	exon 1: NW_005871139.1: 3184646-3185182 bp<br>exon 2: NW_005871139.1: 3182620-3182799 bp	-
					putative	exon 1: NW_005871146.1: 1895211-1895376 bp<br>exon 2: NW_005871146.1: 1910315-1910377 bp<br>exon 3: NW_005871146.1: 1912199-1912968 bp	-
		Large flying fox	GCF_000151845.1	2	*ADIH*	exon 1: NW_011888811.1: 7322704-7322947 bp<br>exon 2: NW_011888811.1: 7325947-7326560 bp	LT963153
					ADIR	exon 1: NW_011888863.1: 5602986-5603462 bp	LT963154
					putative	exon 1: NW_011888786.1: 1013624-1013861 bp<br>exon 2: NW_011888786.1: 1019748-1020379 bp	-

					putative	exon 1: NW_011888795.1: 7494478-7494817 bp<br>exon 2: NW_011888795.1: 7493002-7493522 bp	-
					putative	exon 1: NW_011888817.1: 2415897-2416059 bp<br>exon 2: NW_011888817.1: 2417077-2417651 bp	-
					putative	exon 1: NW_011888817.1: 2423049-2423229 bp<br>exon 2: NW_011888817.1: 2426313-2426869 bp	-
					putative	exon 1: NW_011888817.1: 2436547-2436727 bp<br>exon 2: NW_011888817.1: 2437897-2438471 bp	-
					putative	exon 1: NW_011888852.1: 320818-320977 bp<br>exon 2: NW_011888852.1: 325474-325536 bp<br>exon 3: NW_011888852.1: 326855-327627 bp	-
					putative	exon 1: NW_011888921.1: 5060132-5060668 bp<br>exon 2: NW_011888921.1: 5057863-5058042 bp	-
					putative	exon 1: NW_011889084.1: 1503120-1503321 bp<br>exon 2: NW_011889084.1: 1501814-1502364 bp	-
					putative	exon 1: NW_011889087.1: 1528145-1528741 bp<br>exon 2: NW_011889087.1: 1534180-1534359 bp	-
					putative	exon 1: NW_011889414.1: 833886-834240 bp<br>exon 2: NW_011889414.1: 833000-833517 bp	-
	Eulipotyphla	West European hedgehog	GCF_000296755.1	1	*ADIR*	exon 1: NW_006805039.1: 274613-275011 bp	LT963155
					putative	exon 1: NW_006803936.1: 1313928-1314515 bp<br>exon 2: NW_006803936.1: 1308290-1308469 bp	-
					putative	exon 1: NW_006804004.1: 2448290-2448527 bp<br>exon 2: NW_006804004.1: 2442024-2442655 bp	-
					putative	exon 1: NW_006804020.1: 1404214-1405218 bp	-
					putative	exon 1: NW_006804031.1: 315465-315678 bp<br>exon 2: NW_006804031.1: 311707-312227 bp	-
					putative	exon 1: NW_006804130.1: 1981468-1981681 bp<br>exon 2: NW_006804130.1: 1981982-1982499 bp	-
					putative	exon 1: NW_006804176.1: 1127616-1128299 bp<br>exon 2: NW_006804176.1: 1126867-1127046 bp	-
					putative	exon 1: NW_006804308.1: 1619011-1619547 bp<br>exon 2: NW_006804308.1: 1622813-1622992 bp	-

					putative	exon 1: NW_006804357.1: 1710873-1711116 bp<br>exon 2: NW_006804357.1: 1716370-1716983 bp	-
					putative	exon 1: NW_006804553.1: 446668-446882 bp<br>exon 2: NW_006804553.1: 449238-449377 bp<br>exon 3: NW_006804553.1: 450071-450621 bp	-
					putative	exon 1: NW_006804716.1: 590526-590688 bp<br>exon 2: NW_006804716.1: 591668-592242 bp	-
					putative	exon 1: NW_006804716.1: 595877-596051 bp<br>exon 2: NW_006804716.1: 597870-598426 bp	-
					putative	exon 1: NW_006804768.1: 532289-532885 bp<br>exon 2: NW_006804768.1: 539649-539828 bp	-
		Common shrew	GCF_000181275.1	0	putative	exon 1: NW_004545859.1: 56157-56693 bp<br>exon 2: NW_004545859.1: 54187-54366 bp	-
					putative	exon 1: NW_004545863.1: 29488559-29488772 bp<br>exon 2: NW_004545863.1: 29486631-29487151 bp	-
					putative	exon 1: NW_004545864.1: 28089156-28089318 bp<br>exon 2: NW_004545864.1: 28090293-28090873 bp	-
					putative	exon 1: NW_004545864.1: 28095465-28095645 bp<br>exon 2: NW_004545864.1: 28096255-28096811 bp	-
					putative	exon 1: NW_004545864.1: 28105188-28105449 bp<br>exon 2: NW_004545864.1: 28106918-28107492 bp	-
					putative	exon 1: NW_004545868.1: 3815977-3816145 bp<br>exon 2: NW_004545868.1: 3819823-3819885 bp<br>exon 3: NW_004545868.1: 3824044-3824813 bp	-
					putative	exon 1: NW_004545869.1: 7100732-7101074 bp<br>exon 2: NW_004545869.1: 7099942-7100459 bp	-
					putative	exon 1: NW_004545870.1: 6085126-6085713 bp<br>exon 2: NW_004545870.1: 6079156-6079335 bp	-
					putative	exon 1: NW_004545871.1: 7579956-7580039 bp<br>exon 2: NW_004545871.1: 7588108-7588219 bp<br>exon 3: NW_004545871.1: 7590704-7590858 bp<br>exon 4: NW_004545871.1: 7593267-7593396 bp<br>exon 5: NW_004545871.1: 7596134-7596233 bp<br>exon 6: NW_004545871.1: 7597951-7598110 bp	-

					putative	exon 1: NW_004545988.1: 3578498-3578735 bp<br>exon 2: NW_004545988.1: 3581327-3581958 bp	-
					putative	exon 1: NW_004546032.1: 1350094-1350690 bp<br>exon 2: NW_004546032.1: 1343982-1344161 bp	-
Xenarthra	Xenarthra	Nine-banded armadillo	GCF_000208655.1	7	*ADIA*	exon 1: NW_004482847.1: 247585-247756 bp<br>exon 2: NW_004482847.1: 245835-246409 bp	LT963156
					ADID	exon 1: NW_004486358.1: 1068884-1069094 bp<br>exon 2: NW_004486358.1: 1067488-1068008 bp	LT963157
					ADIF1	exon 1: NW_004469786.1: 1151898-1152066 bp<br>exon 2: NW_004469786.1: 1147941-1148003 bp<br>exon 3: NW_004469786.1: 1144778-1145547 bp	LT963158
					ADII	exon 1: NW_004483128.1: 246587-247270 bp<br>exon 2: NW_004483128.1: 245794-245973 bp	LT963159
					ADIK	exon 1: NW_004458728.1: 593374-593910 bp<br>exon 2: NW_004458728.1: 590838-591017 bp	LT963160
					ADIN	exon 1: NW_004478827.1: 1694955-1695929 bp	LT963161
					ADIR	exon 1: NW_004492584.1: 719820-720296 bp	LT963162
		Hoffmann's two-toed sloth	GCA_000164785.2	1	*ADIR*	exon 1: KN179874.1: 99200-99676 bp	LT963163
					putative	exon 1: KN181662.1: 167524-167842 bp<br>exon 2: KN181662.1: 168735-169255 bp	-
					putative	exon 1: KN183950.1: 95837-96373 bp<br>exon 2: KN183950.1: 93384-93563 bp	-
					putative	exon 1: KN184977.1: 300680-300860 bp<br>exon 2: KN184977.1: 298980-299554 bp	-
					putative	exon 1: KN184977.1: 313080-313254 bp<br>exon 2: KN184977.1: 309188-309744 bp	-
					putative	exon 1: KN184977.1: 318715-318877 bp<br>exon 2: KN184977.1: 317303-317874 bp	-
					putative	exon 1: KN186210.1: 230703-230946 bp<br>exon 2: KN186210.1: 235594-236207 bp	-
					putative	exon 1: KN190228.1: 421139-421415 bp<br>exon 2: KN190228.1: 426559-426621 bp<br>exon 3: KN190228.1: 429175-429944 bp	-

					putative	exon 1: KN193614.1: 312498-312735 bp<br>exon 2: KN193614.1: 318056-318687 bp	-
					putative	exon 1: KN194842.1: 1230542-1230891 bp<br>exon 2: KN194842.1: 1228018-1228160 bp<br>exon 3: KN194842.1: 1226753-1227303 bp	-
Afrotheria	Tenrecidae	Lesser hedgehog tenrec	GCF_000313985.1	0	putative	exon 1: NW_004558702.1: 52641539-52641782 bp<br>exon 2: NW_004558702.1: 52637469-52638082 bp	-
					putative	exon 1: NW_004558704.1: 56713230-56713313 bp<br>exon 2: NW_004558704.1: 56721682-56721793 bp<br>exon 3: NW_004558704.1: 56724393-56724547 bp<br>exon 4: NW_004558704.1: 56731511-56731640 bp<br>exon 5: NW_004558704.1: 56734613-56734712 bp<br>exon 6: NW_004558704.1: 56738361-56738520 bp	-
					putative	exon 1: NW_004558707.1: 74452597-74453073 bp	-
					putative	exon 1: NW_004558721.1: 22574997-22575177 bp<br>exon 2: NW_004558721.1: 22572461-22573035 bp	-
					putative	exon 1: NW_004558721.1: 22587002-22587263 bp<br>exon 2: NW_004558721.1: 22583328-22583884 bp	-
					putative	exon 1: NW_004558721.1: 22590804-22590966 bp<br>exon 2: NW_004558721.1: 22589239-22589813 bp	-
					putative	exon 1: NW_004558729.1: 5947615-5947852 bp<br>exon 2: NW_004558729.1: 5956979-5957610 bp	-
					putative	exon 1: NW_004558776.1: 5327060-5327267 bp<br>exon 2: NW_004558776.1: 5320318-5320841 bp	-
	Proboscidea	African bush elephant	GCF_000001905.1	11	*ADIA*	exon 1: NW_003573454.1: 698193-698373 bp<br>exon 2: NW_003573454.1: 699585-700159 bp	LT963164
					ADIB	exon 1: NW_003573454.1: 684473-684647 bp<br>exon 2: NW_003573454.1: 688519-689075 bp	LT963165
					ADIC	exon 1: NW_003573454.1: 677901-678108 bp<br>exon 2: NW_003573454.1: 679128-679702 bp	LT963166
					ADID	exon 1: NW_003573445.1: 7326031-7326244 bp<br>exon 2: NW_003573445.1: 7327130-7327653 bp	LT963167
					ADIF1	exon 1: NW_003573449.1: 9289856-9290021 bp<br>exon 2: NW_003573449.1: 9283939-9284001 bp	LT963168

						exon 3: NW_003573449.1: 9279123-9279895 bp	
					ADIH	exon 1: NW_003573421.1: 81637309-81637552 bp<br>exon 2: NW_003573421.1: 81631533-81632146 bp	LT963169
					ADIJ	exon 1: NW_003573435.1: 28440462-28441049 bp<br>exon 2: NW_003573435.1: 28447086-28447265 bp	LT963170
					ADIK	exon 1: NW_003573422.1: 35671260-35671796 bp<br>exon 2: NW_003573422.1: 35668732-35668911 bp	LT963171
					ADIN	exon 1: NW_003573441.1: 1943871-1944842 bp	LT963172
					ADIP	exon 1: NW_003573469.1: 3887002-3887312 bp<br>exon 2: NW_003573469.1: 3885225-3885364 bp<br>exon 3: NW_003573469.1: 3883054-3883604 bp	LT963173
					ADIR	exon 1: NW_003573422.1: 20568280-20568756 bp	LT963174
					putative	exon 1: NW_003573427.1: 37993392-37993475 bp<br>exon 2: NW_003573427.1: 37985720-37985831 bp<br>exon 3: NW_003573427.1: 37983707-37983861 bp<br>exon 4: NW_003573427.1: 37976139-37976268 bp<br>exon 5: NW_003573427.1: 37973571-37973670 bp<br>exon 6: NW_003573427.1: 37968766-37968925 bp	-
					putative	exon 1: NW_003573438.1: 13936475-13936712 bp<br>exon 2: NW_003573438.1: 13945126-13945757 bp	-
					putative	exon 1: NW_003573466.1: 1876400-1877113 bp<br>exon 2: NW_003573466.1: 1875616-1875795 bp	-
	Hyracoidea	Rock hyrax	GCA_000152225.2	0	putative	exon 1: KN676296.1: 815373-815583 bp<br>exon 2: KN676296.1: 813964-814514 bp	-
					putative	exon 1: KN676324.1: 621359-621649 bp<br>exon 2: KN676324.1: 630251-630362 bp<br>exon 3: KN676324.1: 632928-633082 bp<br>exon 4: KN676324.1: 639629-639758 bp<br>exon 5: KN676324.1: 642614-642713 bp<br>exon 6: KN676324.1: 645818-645977 bp	-
					putative	exon 1: KN676395.1: 2436577-2437113 bp<br>exon 2: KN676395.1: 2439365-2439544 bp	-
					putative	exon 1: KN676607.1: 806920-808203 bp	-
					putative	exon 1: KN676681.1: 751590-752066 bp	-

					putative	exon 1: KN676701.1: 232488-232725 bp<br>exon 2: KN676701.1: 240568-241199 bp	-
					putative	exon 1: KN677080.1: 243449-244036 bp<br>exon 2: KN677080.1: 237108-237287 bp	-
					putative	exon 1: KN677106.1: 137832-138045 bp<br>exon 2: KN677106.1: 138931-139451 bp	-
					putative	exon 1: KN677358.1: 173433-173598 bp<br>exon 2: KN677358.1: 181877-181939 bp<br>exon 3: KN677358.1: 186081-186853 bp	-
					putative	exon 1: KN677980.1: 374425-374605 bp<br>exon 2: KN677980.1: 372631-373205 bp	-
					putative	exon 1: KN677980.1: 387518-387692 bp<br>exon 2: KN677980.1: 383575-384131 bp	-
					putative	exon 1: KN677980.1: 394145-394307 bp<br>exon 2: KN677980.1: 392555-393129 bp	-
					putative	exon 1: KN678277.1: 241471-241789 bp<br>exon 2: KN678277.1: 242131-242648 bp	-
					putative	exon 1: KN678862.1: 173997-174178 bp<br>exon 2: KN678862.1: 175462-175601 bp<br>exon 3: KN678862.1: 177688-178238 bp	-
					putative	exon 1: KN681188.1: 64066-64749 bp<br>exon 2: KN681188.1: 65372-65551 bp	-
					putative	exon 1: KN681263.1: 59335-59578 bp<br>exon 2: KN681263.1: 66432-67045 bp	-

[a], the common names were cited from Wilson and Reeder (2005); [b], the human *ADI* gene abbreviations provided in parentheses were cited from Sellar et al. (1991), Kishore et al. (2004), Seldin et al. (2014) and present analysis; [c], translated exons; ch., chromosome.
