# Supplementary material for: Comparative genomic analysis of eutherian adiponectin genes
Source: Heliyon. 2018 Jun 6;4(6):e00647. doi: 10.1016/j.heliyon.2018.e00647 (PMC6040601; doi:10.1016/j.heliyon.2018.e00647)
Supplement: Supplementary data file 2 — Multiple pairwise genomic sequence alignments of eutherian adiponectin genes. The indigo rectangles displayed translated exons in base sequences (top). In each pairwise genomic sequence alignment, the genomic sequence regions including sequence identity levels above empirical cut-offs of detection of common genomic sequence regions were shown accordingly. The rectangles labelled common predicted promoter genomic sequence regions (P). [file mmc2.zip › hly_647_Supplementary data file 2 - part 3.pdf]

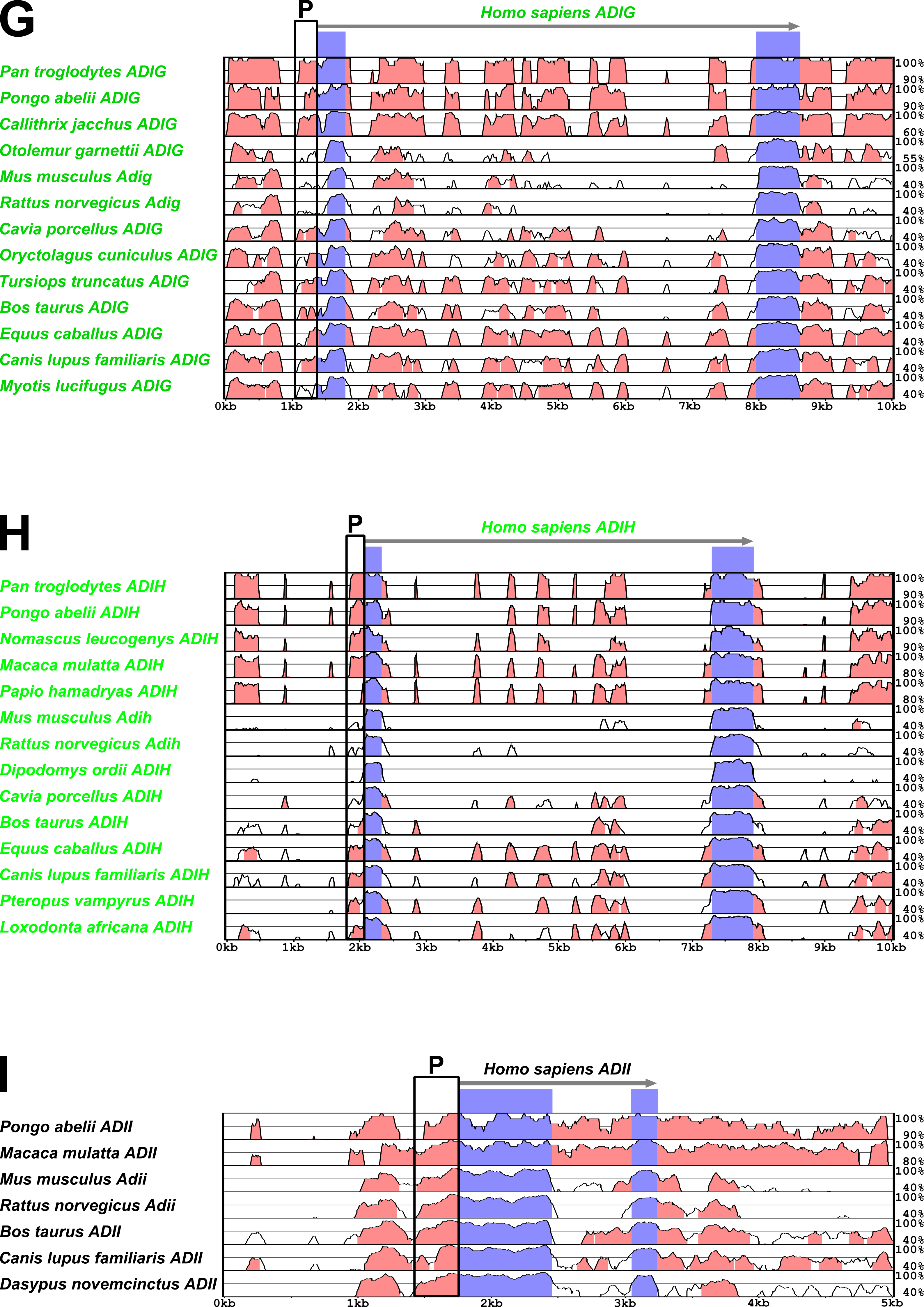
G
P
Homo sapiens ADIG
Pan troglodytes ADIG
Pongo abelii ADIG
Callithrix jacchus ADIG
Otolemur garnettii ADIG
Mus musculus Adig
Rattus norvegicus Adig
Cavia porcellus ADIG
Oryctolagus cuniculus ADIG
Tursiops truncatus ADIG
Bos taurus ADIG
Equus caballus ADIG
Canis lupus familiaris ADIG
Myotis lucifugus ADIG
100%
90%
60%
55%
40%
0kb
1kb
2kb
3kb
4kb
5kb
6kb
7kb
8kb
9kb
10kb
H
P
Homo sapiens ADIH
Pan troglodytes ADIH
Pongo abelii ADIH
Nomascus leucogenys ADIH
Macaca mulatta ADIH
Papio hamadryas ADIH
Mus musculus Adih
Rattus norvegicus Adih
Dipodomys ordii ADIH
Cavia porcellus ADIH
Bos taurus ADIH
Equus caballus ADIH
Canis lupus familiaris ADIH
Pteropus vampyrus ADIH
Loxodonta africana ADIH
100%
90%
80%
40%
0kb
1kb
2kb
3kb
4kb
5kb
6kb
7kb
8kb
9kb
10kb
I
P
Homo sapiens ADII
Pongo abelii ADII
Macaca mulatta ADII
Mus musculus Adii
Rattus norvegicus Adii
Bos taurus ADII
Canis lupus familiaris ADII
Dasypus novemcinctus ADII
100%
90%
80%
40%
0kb
1kb
2kb
3kb
4kb
5kb
