# Supplementary material for: Comparative genomic analysis of eutherian adiponectin genes
Source: Heliyon. 2018 Jun 6;4(6):e00647. doi: 10.1016/j.heliyon.2018.e00647 (PMC6040601; doi:10.1016/j.heliyon.2018.e00647)
Supplement: Supplementary data file 2 — Multiple pairwise genomic sequence alignments of eutherian adiponectin genes. The indigo rectangles displayed translated exons in base sequences (top). In each pairwise genomic sequence alignment, the genomic sequence regions including sequence identity levels above empirical cut-offs of detection of common genomic sequence regions were shown accordingly. The rectangles labelled common predicted promoter genomic sequence regions (P). [file mmc2.zip › hly_647_Supplementary data file 2 - part 4.pdf]

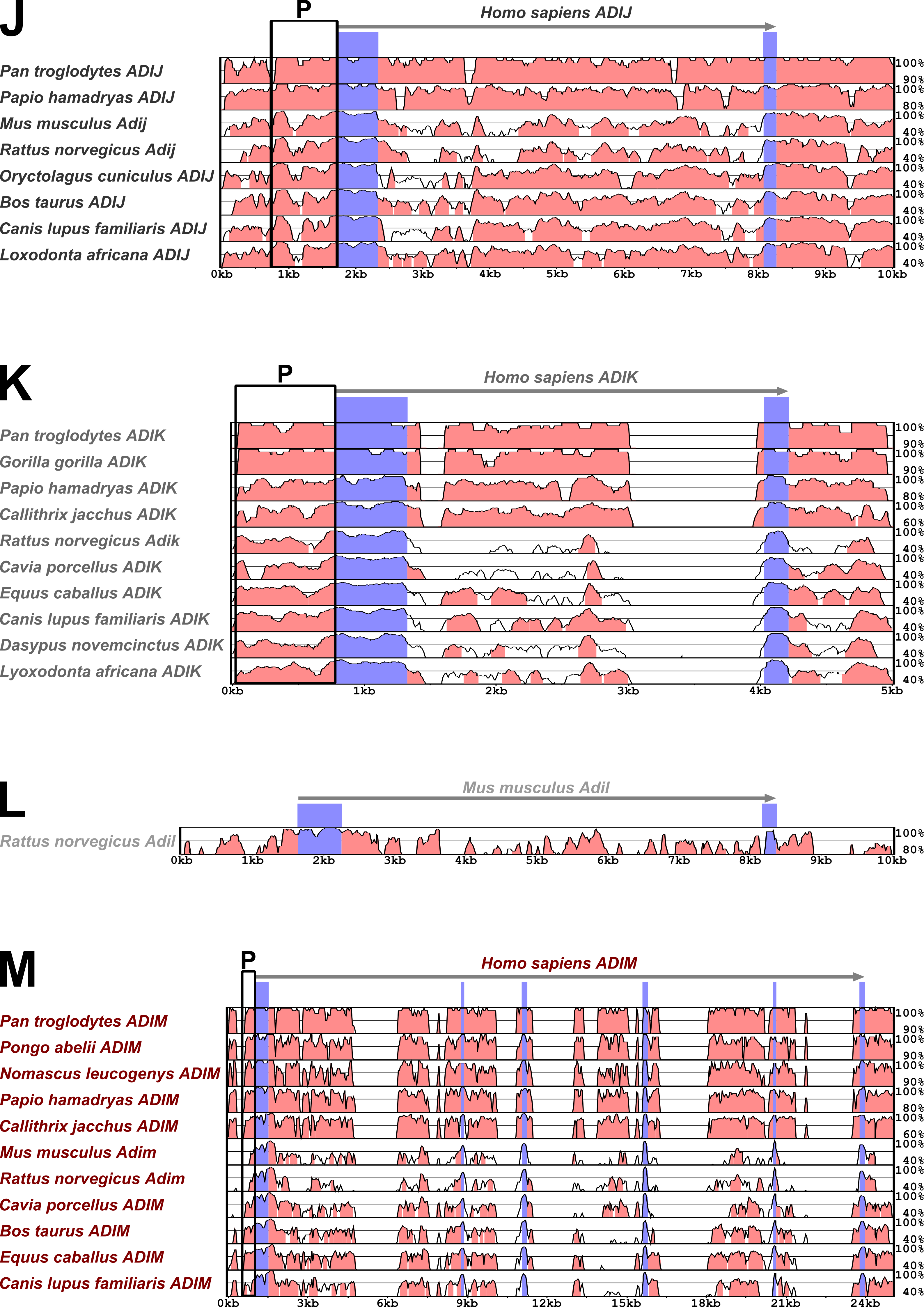
J
P
Homo sapiens ADIJ
Pan troglodytes ADIJ
Papio hamadryas ADIJ
Mus musculus Adij
Rattus norvegicus Adij
Oryctolagus cuniculus ADIJ
Bos taurus ADIJ
Canis lupus familiaris ADIJ
Loxodonta africana ADIJ
100%
90%
80%
40%
0kb 1kb 2kb 3kb 4kb 5kb 6kb 7kb 8kb 9kb 10kb
K
P
Homo sapiens ADIK
Pan troglodytes ADIK
Gorilla gorilla ADIK
Papio hamadryas ADIK
Callithrix jacchus ADIK
Rattus norvegicus Adik
Cavia porcellus ADIK
Equus caballus ADIK
Canis lupus familiaris ADIK
Dasypus novemcinctus ADIK
Lyoxodonta africana ADIK
100%
90%
80%
60%
40%
0kb 1kb 2kb 3kb 4kb 5kb
L
Mus musculus Adil
Rattus norvegicus Adil
100%
80%
0kb 1kb 2kb 3kb 4kb 5kb 6kb 7kb 8kb 9kb 10kb
M
P
Homo sapiens ADIM
Pan troglodytes ADIM
Pongo abelii ADIM
Nomascus leucogenys ADIM
Papio hamadryas ADIM
Callithrix jacchus ADIM
Mus musculus Adim
Rattus norvegicus Adim
Cavia porcellus ADIM
Bos taurus ADIM
Equus caballus ADIM
Canis lupus familiaris ADIM
100%
90%
80%
60%
40%
0kb 3kb 6kb 9kb 12kb 15kb 18kb 21kb 24kb
