# Supplementary material for: Comparative genomic analysis of eutherian adiponectin genes
Source: Heliyon. 2018 Jun 6;4(6):e00647. doi: 10.1016/j.heliyon.2018.e00647 (PMC6040601; doi:10.1016/j.heliyon.2018.e00647)
Supplement: Supplementary data file 2 — Multiple pairwise genomic sequence alignments of eutherian adiponectin genes. The indigo rectangles displayed translated exons in base sequences (top). In each pairwise genomic sequence alignment, the genomic sequence regions including sequence identity levels above empirical cut-offs of detection of common genomic sequence regions were shown accordingly. The rectangles labelled common predicted promoter genomic sequence regions (P). [file mmc2.zip › hly_647_Supplementary data file 2 - part 5.pdf]

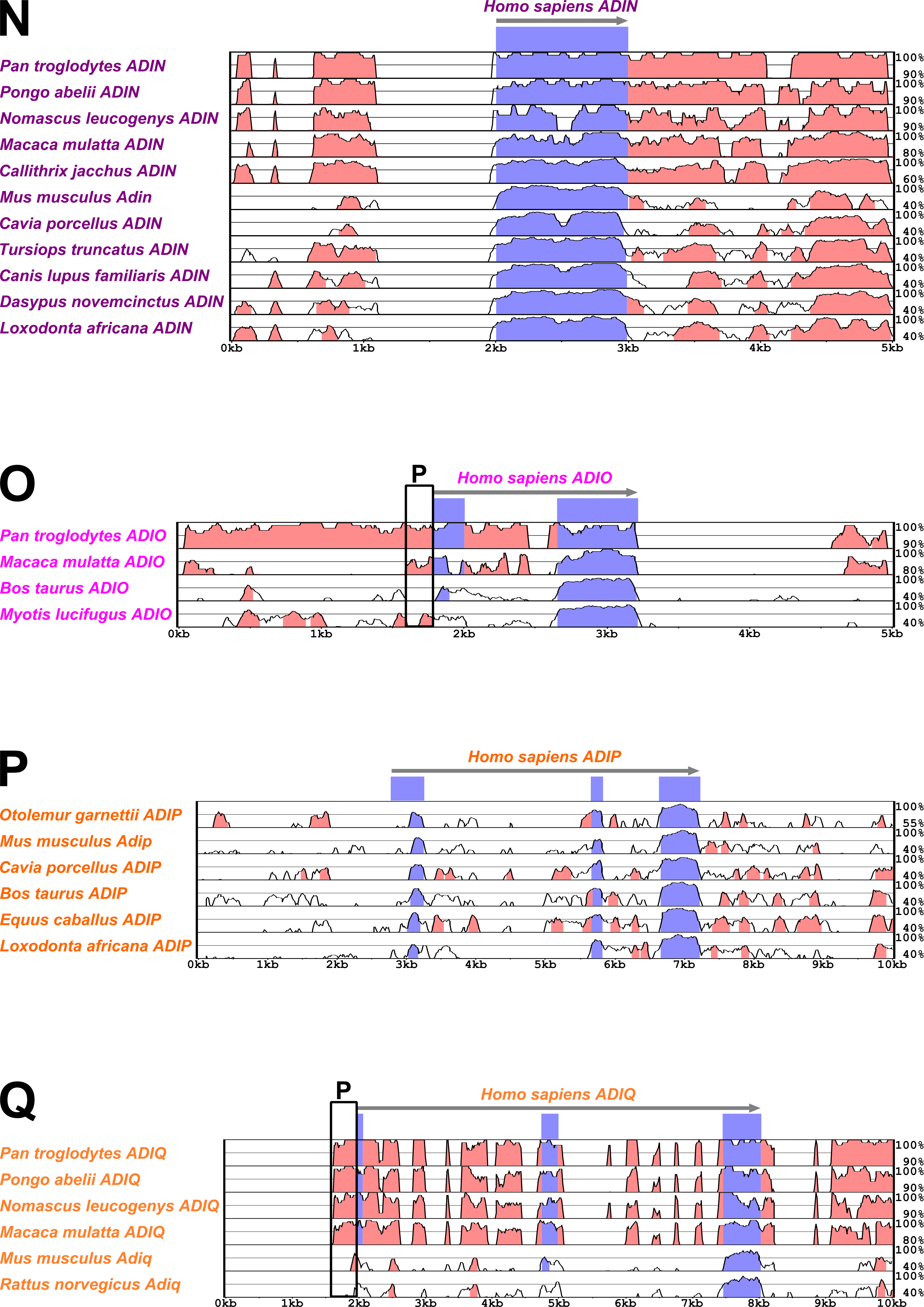
N
Homo sapiens ADIN
Pan troglodytes ADIN
Pongo abelii ADIN
Nomascus leucogenys ADIN
Macaca mulatta ADIN
Callithrix jacchus ADIN
Mus musculus Adin
Cavia porcellus ADIN
Tursiops truncatus ADIN
Canis lupus familiaris ADIN
Dasypus novemcinctus ADIN
Loxodonta africana ADIN
100%
90%
80%
60%
40%
0kb
1kb
2kb
3kb
4kb
5kb
O
P
Homo sapiens ADIO
Pan troglodytes ADIO
Macaca mulatta ADIO
Bos taurus ADIO
Myotis lucifugus ADIO
P
Homo sapiens ADIP
Otolemur garnettii ADIP
Mus musculus Adip
Cavia porcellus ADIP
Bos taurus ADIP
Equus caballus ADIP
Loxodonta africana ADIP
55%
6kb
7kb
8kb
9kb
10kb
Q
Homo sapiens ADIQ
Pan troglodytes ADIQ
Pongo abelii ADIQ
Nomascus leucogenys ADIQ
Macaca mulatta ADIQ
Mus musculus Adiq
Rattus norvegicus Adiq
