# Supplementary material for: Comparative genomic analysis of eutherian adiponectin genes
Source: Heliyon. 2018 Jun 6;4(6):e00647. doi: 10.1016/j.heliyon.2018.e00647 (PMC6040601; doi:10.1016/j.heliyon.2018.e00647)
Supplement: Supplementary data file 2 — Multiple pairwise genomic sequence alignments of eutherian adiponectin genes. The indigo rectangles displayed translated exons in base sequences (top). In each pairwise genomic sequence alignment, the genomic sequence regions including sequence identity levels above empirical cut-offs of detection of common genomic sequence regions were shown accordingly. The rectangles labelled common predicted promoter genomic sequence regions (P). [file mmc2.zip › hly_647_Supplementary data file 2 - part 6.pdf]

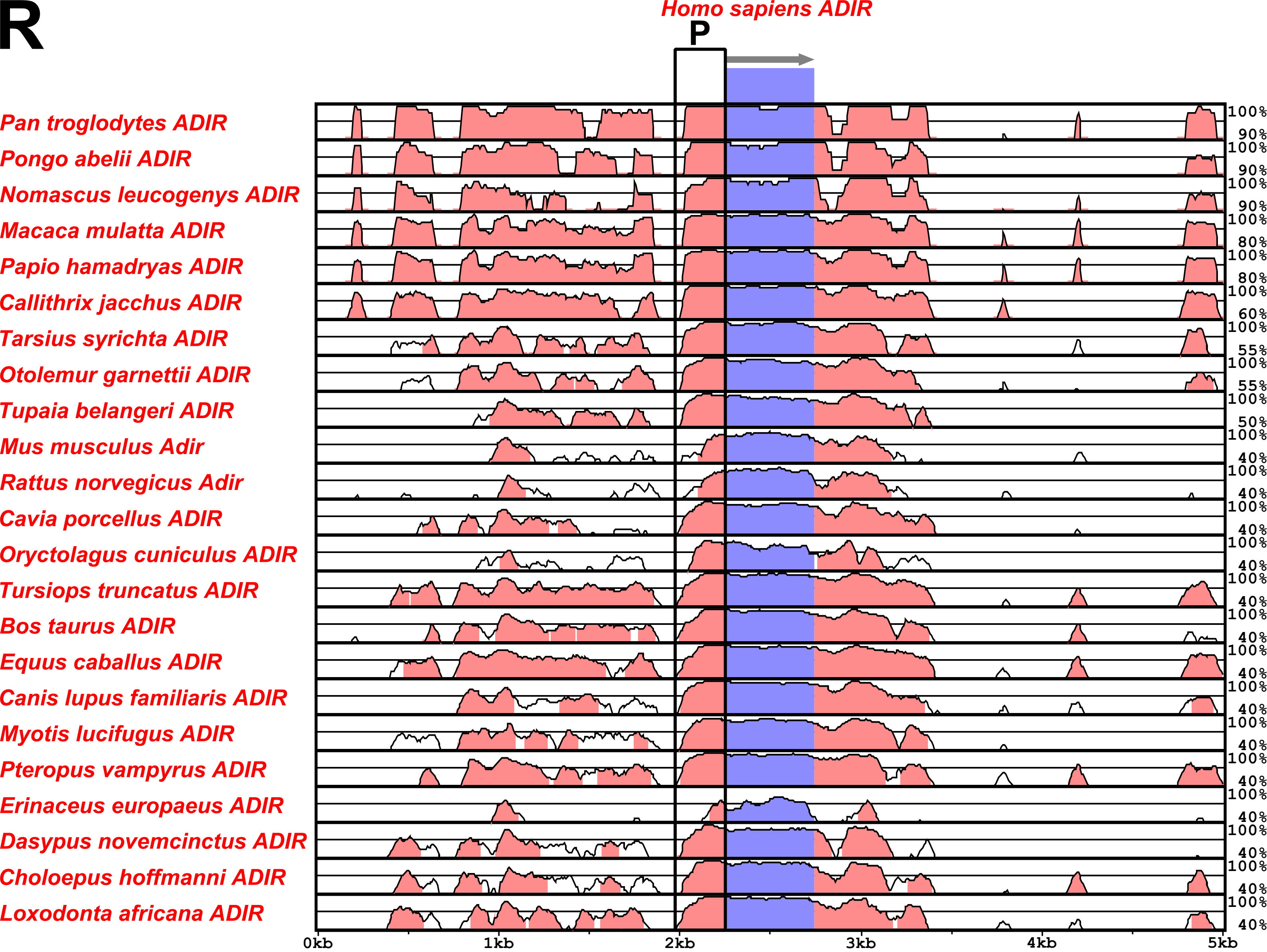
R
Homo sapiens ADIR
P
Pan troglodytes ADIR
Pongo abelii ADIR
Nomascus leucogenys ADIR
Macaca mulatta ADIR
Papio hamadryas ADIR
Callithrix jacchus ADIR
Tarsius syrichta ADIR
Otolemur garnettii ADIR
Tupaia belangeri ADIR
Mus musculus Adir
Rattus norvegicus Adir
Cavia porcellus ADIR
Oryctolagus cuniculus ADIR
Tursiops truncatus ADIR
Bos taurus ADIR
Equus caballus ADIR
Canis lupus familiaris ADIR
Myotis lucifugus ADIR
Pteropus vampyrus ADIR
Erinaceus europaeus ADIR
Dasypus novemcinctus ADIR
Choloepus hoffmanni ADIR
Loxodonta africana ADIR
0kb
1kb
2kb
3kb
4kb
5kb
100%
90%
80%
60%
55%
50%
40%
