# Supplementary figures and images for: Comparative genomic analysis of eutherian adiponectin genes
Source: Heliyon. 2018 Jun 6;4(6):e00647. doi: 10.1016/j.heliyon.2018.e00647 (PMC6040601; doi:10.1016/j.heliyon.2018.e00647)

**A**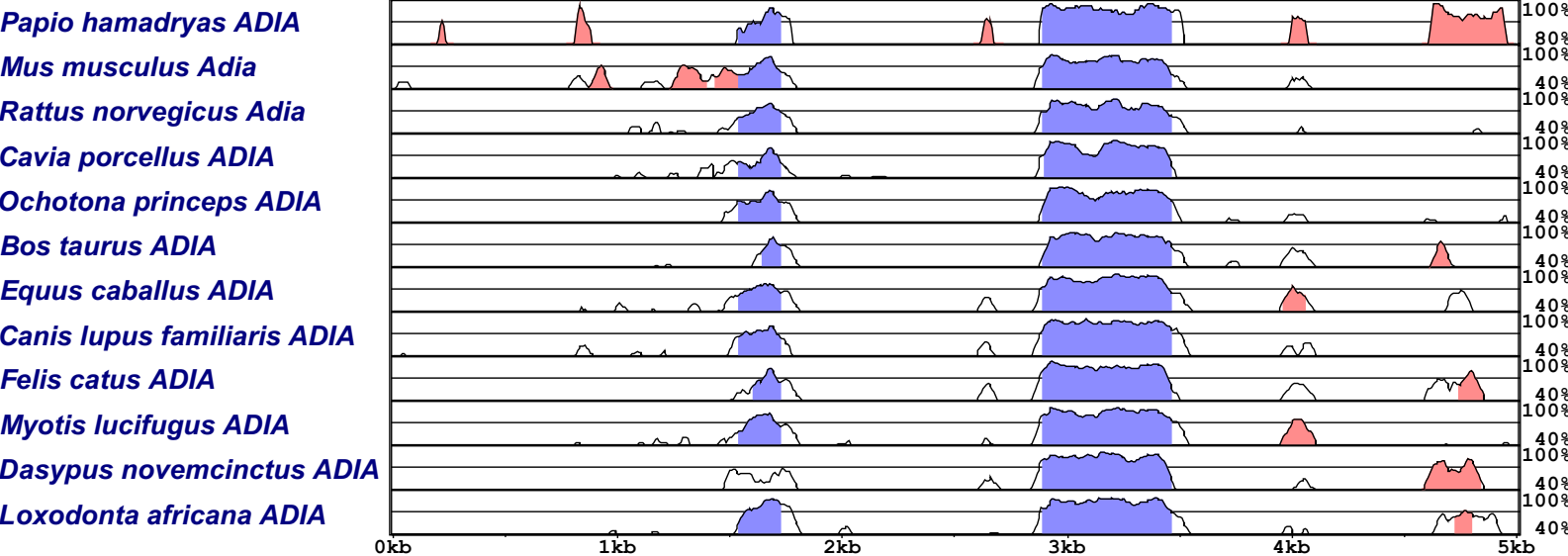**B**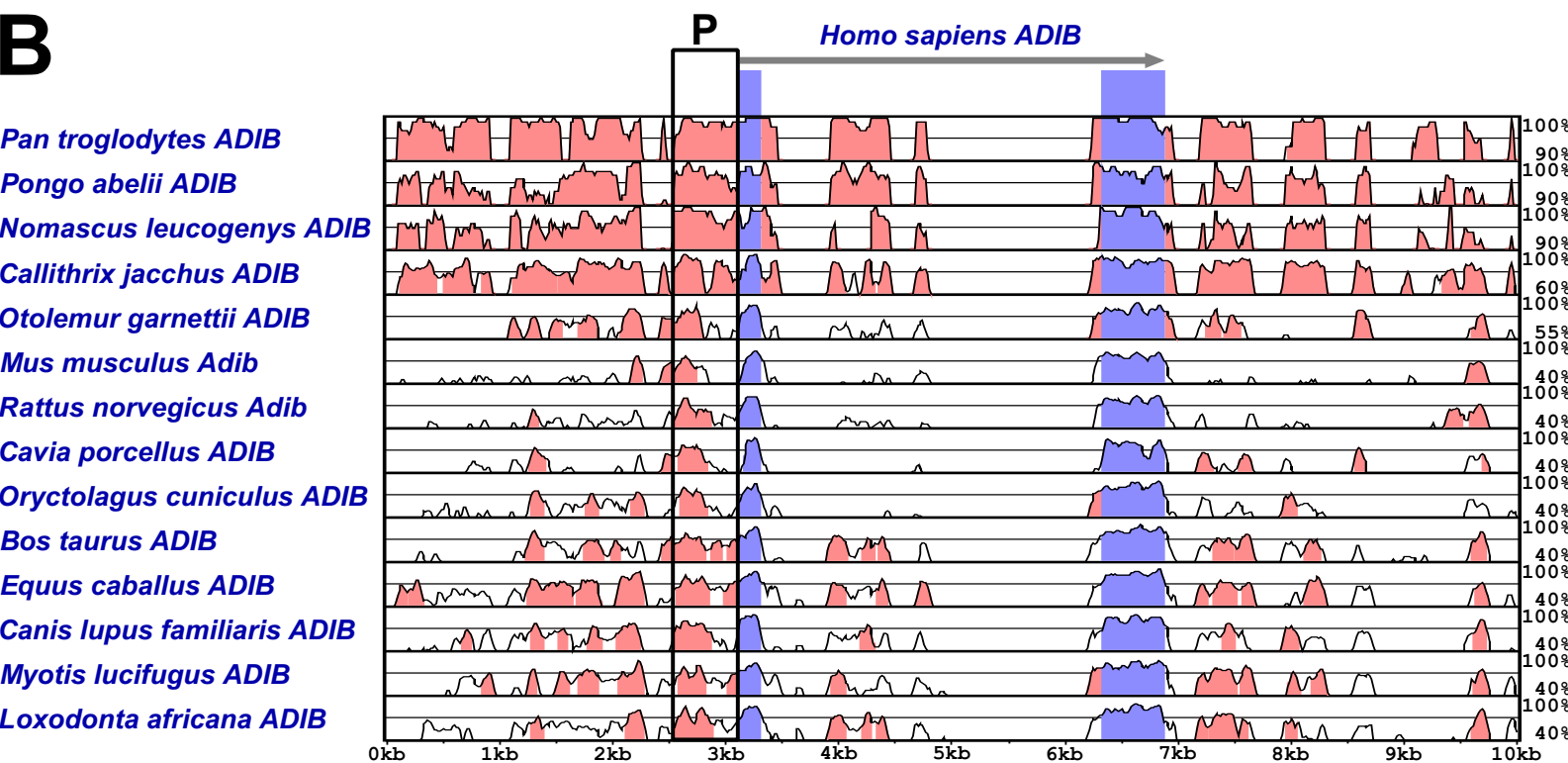**C**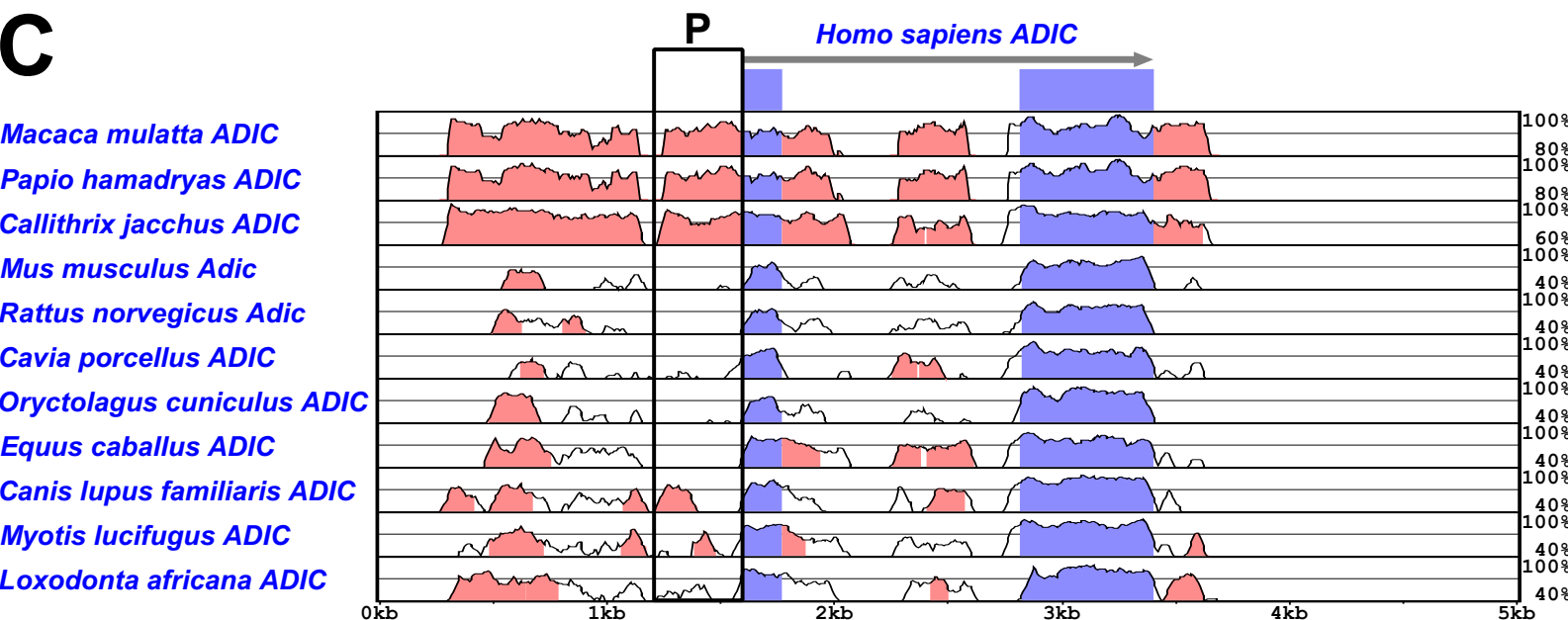

Supplement: Supplementary data file 2 — Multiple pairwise genomic sequence alignments of eutherian adiponectin genes. The indigo rectangles displayed translated exons in base sequences (top). In each pairwise genomic sequence alignment, the genomic sequence regions including sequence identity levels above empirical cut-offs of detection of common genomic sequence regions were shown accordingly. The rectangles labelled common predicted promoter genomic sequence regions (P). [file mmc2.zip › hly_647_Supplementary data file 2 - part 1.pdf]

**D**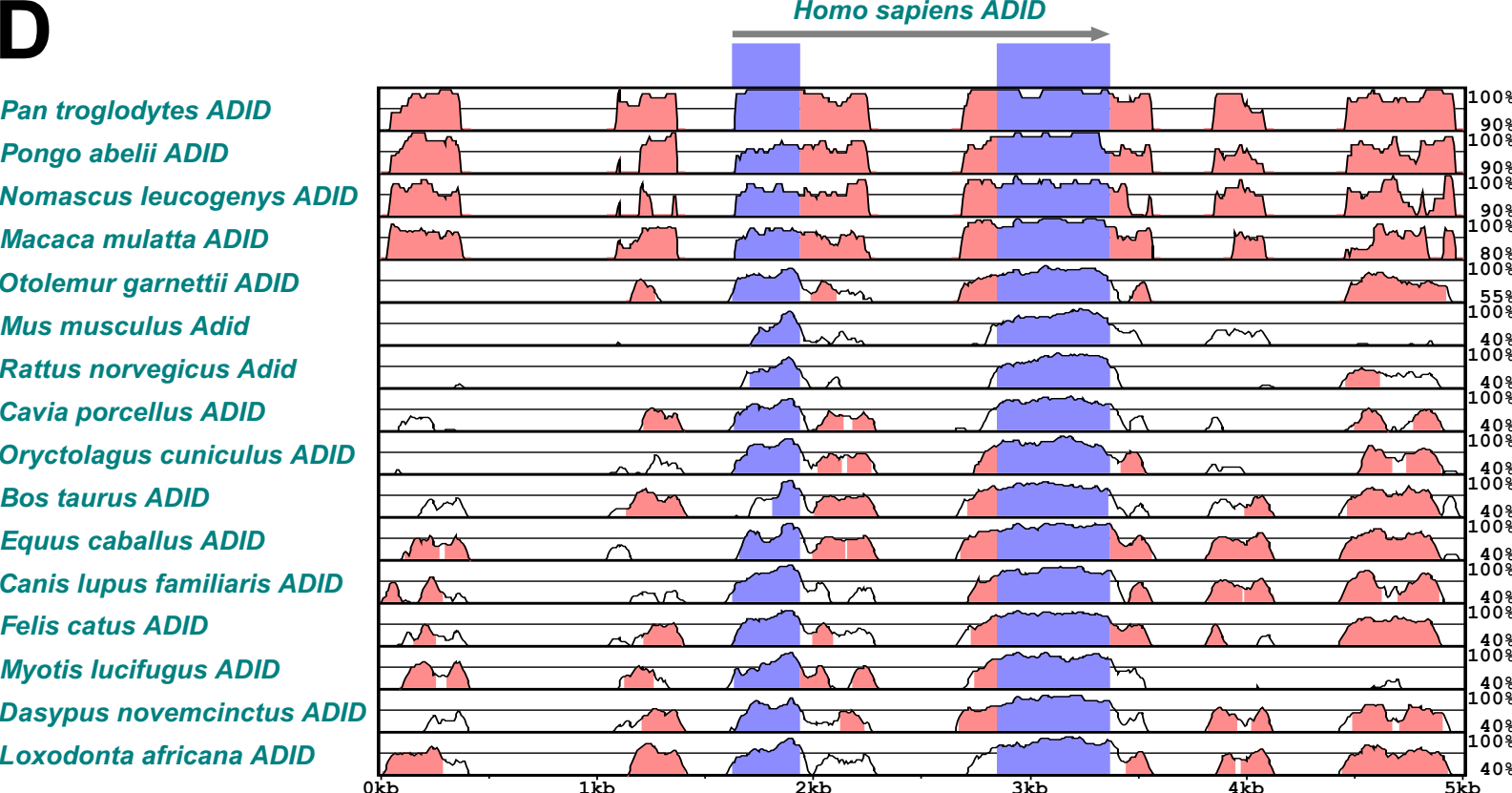**E**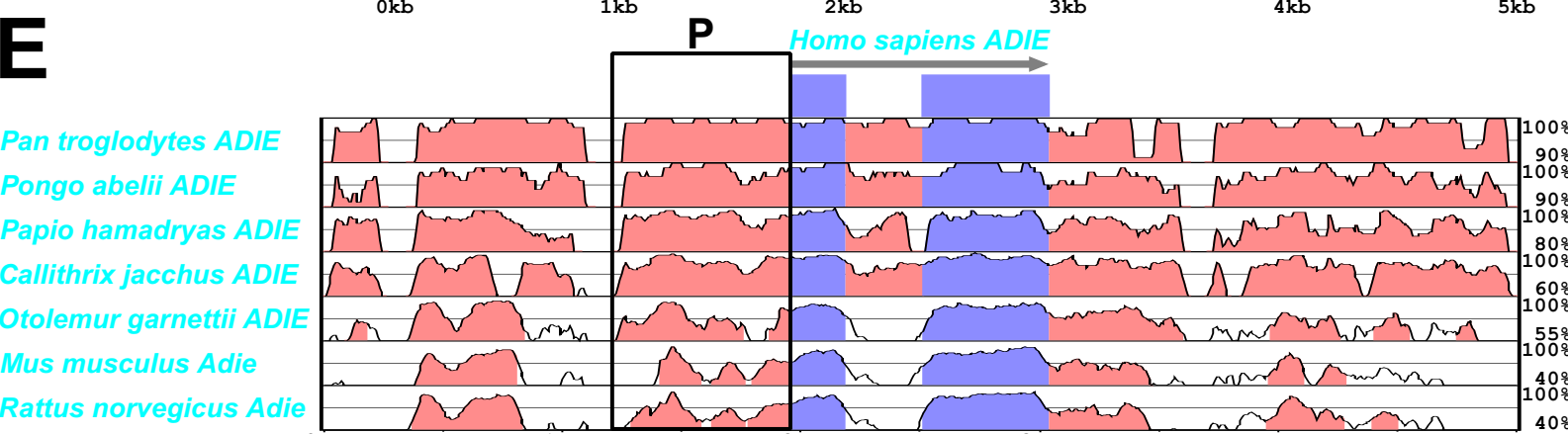**F**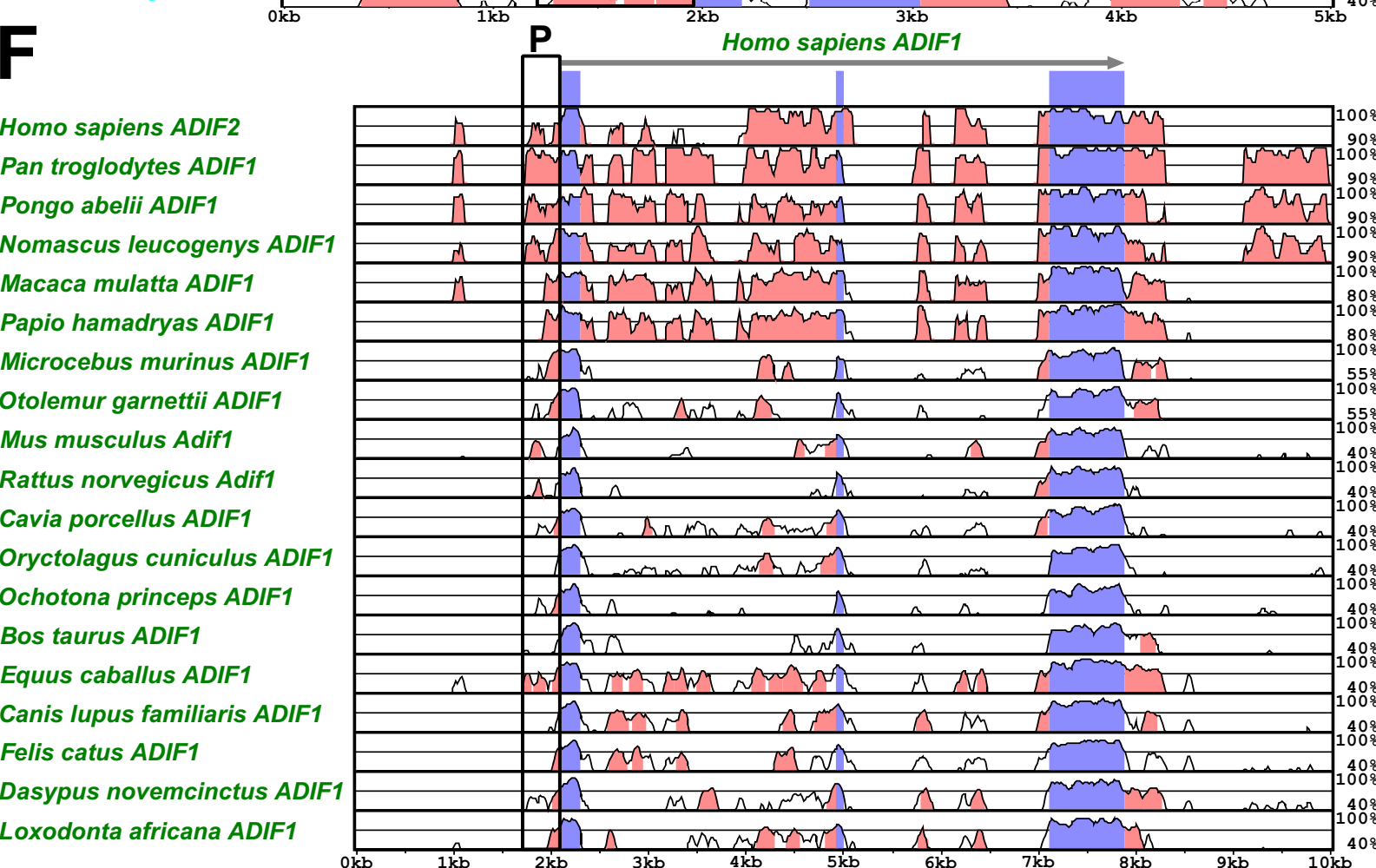

Supplement: Supplementary data file 2 — Multiple pairwise genomic sequence alignments of eutherian adiponectin genes. The indigo rectangles displayed translated exons in base sequences (top). In each pairwise genomic sequence alignment, the genomic sequence regions including sequence identity levels above empirical cut-offs of detection of common genomic sequence regions were shown accordingly. The rectangles labelled common predicted promoter genomic sequence regions (P). [file mmc2.zip › hly_647_Supplementary data file 2 - part 2.pdf]
