# Supplementary material for: Comparative genomic analysis of eutherian adiponectin genes
Source: Heliyon. 2018 Jun 6;4(6):e00647. doi: 10.1016/j.heliyon.2018.e00647 (PMC6040601; doi:10.1016/j.heliyon.2018.e00647)
Supplement: Supplementary data file 3 — Nucleotide sequence alignments of common predicted promoters of eutherian adiponectin genes. The numbers in brackets indicated positions of 3′-terminal nucleotides relative to translation start sites. The nucleotide positions were labelled according to their identity levels using white letters on black background (100% sequence identity level), white letters on dark grey background (≥85% sequence identity level) or black letters on grey background (≥70% sequence identity level). [file mmc3.pdf]

A

```
                                         10        20        30        40        50        60
Homo sapiens ADIB           1  CACACACACACACACCCTCAGCCAGATGCTGGGGCAGGGGACAGTTCCTCATTTCGGACACTGAC   65
Pan troglodytes ADIB        1  CACACACACACACGCCCTCAGCCAGATGCTGGGGCAGGGGACAGTTCCTCATTTTGGACACTGAA   65
Pongo abelii ADIB           1  CACACACACACACGCCCTCAGCCAGATGCTGGGGCAGGGGACAGTTCCTCATTTCGGACACTGAC   65
Nomascus leucogenys ADIB    1  CACACACACACACACCCTCAGCCAGATGCTGGGGCAGGGGACAGTTCCTCATTTCGGACACTGAC   65
Callithrix jacchus ADIB     1  CACACACACACATGCCCTCAGCCAGATGCTGGGGCAATGGACATTTCCTCATTTCAGACACTCAT   65

                                    70        80        90       100       110       120       130
Homo sapiens ADIB          66  TGCCGGTGGGGGA-TGTGGAT-GGGGTATTGCTGTGGTCAGTCTGGCTCAGCACCCAAAAGAAA  128
Pan troglodytes ADIB       66  TGCCGGTAGGGGG-TGTGGAT-GGGGTATTGCTGTGGTCAGTCTGGCTCAGCACCCAAAAGAAA  128
Pongo abelii ADIB          66  TGCCGGTGGGGGA-TGTGGACAGGGGTATTGCTGTGGTCAGTCTGGCTCTGCACCCAAAAGAAA  129
Nomascus leucogenys ADIB   66  TGCCGGTGGGGGA-TGTGGAC-GGGGTATTGCTGTGGTCAGTCTGGCTCAGCACCCAAAAGAAA  128
Callithrix jacchus ADIB    66  GACTGCCGGTGGGATTGTGGACGGGGGTGTTGCTGTGGTCAGTCTGGCTCAGCACCCAAAAGAAA  130

                                        140       150       160       170       180       190
Homo sapiens ADIB         129  GTGTCAAGTCAGGGAAAATTCTGGGAAAGGGGATGTGGGATTGGGGGAGGGCACGAGAAGGGGAG  193
Pan troglodytes ADIB      129  GTGTCAAGTCAGGGAAAATTCTGGGAAAGGGGATGTGGGATTGGGGGAGGGCACGAGAAGGGGAG  193
Pongo abelii ADIB         130  GTGTCAAGTCAGGGAAAATTCTGGGAAAGGGGATGTGGGATTGGGGGAGGGCACGAGAAGGGGAG  194
Nomascus leucogenys ADIB  129  GTGTCAAGTCAGGGAAAATTCTGGGAAAGGGGATGTGGGATTGGGGGAGGGCACGAGAAGGGGAG  193
Callithrix jacchus ADIB   131  GTGTCAAGTCAGGGAAAATTCTGGGAAAGGGGATGTGGGATTGG-GGAGGGCATGAGAAGGGGAG  194

                                   200       210       220       230       240       250       260
Homo sapiens ADIB         194  GGGGCCCAGCCCTGCTTTGGGCAATCCTTGCTCTGACCACTCAGACACCGTGTCCTCTTGCCTGG  258
Pan troglodytes ADIB      194  GGGGCCCAGCCCTGCTTTGGGCAATCCTTGCTCTGACCACTCGGACACCGTGTCCTCTTGCCTGG  258
Pongo abelii ADIB         195  GGGGCCCAGCCCTGCTTTGGGCAATCCTTGCTCTGACCATTCTGACACCGTGTCCTCTTGCCTGG  259
Nomascus leucogenys ADIB  194  GGGGCCCAGCCCTGCTTTGGGCAATCCTTGCTCTGACCACTCTGACACCGTGTCCTCTTGCCTGG  258
Callithrix jacchus ADIB   195  AGGGCCCAGCCCTGCTCTGGGCAATCCTTGCTCTGACCACTCTGACACCGTATCCTCTTGCCTGG  259

                                        270       280       290       300       310       320
Homo sapiens ADIB         259  GAGAGGGGAAGCAGATCTGAGGACATCTCTGTG-CCAGGCCAGAAACCGCCCACCTGCAGGTGAG  322
Pan troglodytes ADIB      259  GAGAGGGGAAGCAGATCTGAGGACATCTCTGTG-CCAGGCCAGAAACCGCCCACCCGCAGGTGAG  322
Pongo abelii ADIB         260  GAGAGGGGAAGCAGATCTGAGGACATCTCTGTG-CCAGGCCAGAAACCGCCCACCCGCAGGTGAG  323
Nomascus leucogenys ADIB  259  GAGAGGGGAAGCAGATCTGAGGACATCTCTGTG-CCAGGCCAGAAACCGCCCACCCGCAGGTGAG  322
Callithrix jacchus ADIB   260  GAGAGGGGAAGCAGATCTGAGGACATCTCTGTGACCAGGCTAGAAGCTGCACACCTGCAGGTGA-  323

                                   330       340       350       360       370       380       390
Homo sapiens ADIB         323  GCCCGGACCCCTGCCCAGGTATGGGCAGATGGCCGTTTCCTCCACCTTCCGCCACCTTCCTGAAA  387
Pan troglodytes ADIB      323  GCCCGGACCCCTGCCCAGGTATGGGCACATGGCCGTTTCCTCCACCTTCCGCCACCTTCCTGAAA  387
Pongo abelii ADIB         324  GCCCGGACCCCTGCCCAGGTATGGGCAGATGGCCATTTCCTCCATCTTCTGCCACCTTCCTGAAA  388
Nomascus leucogenys ADIB  323  GCCCGGACCCCTGCCCAGGTATGGGCAGATGGCCGTTTCCTCCACCTTCCGCCACCTTCCCGAAA  387
Callithrix jacchus ADIB   324  ----------------------GGGCAGATGGCCATTCCCCCCACCTTCCACCACCTTCCTGAAA  366

                                        400       410       420       430       440       450
Homo sapiens ADIB         388  TGTGGTGCCAGGGGCAGCTTGGTGTGTGAGGGGAAGGAGGGTTCCCACTC-CTGCTGGGCCCCGG  451
Pan troglodytes ADIB      388  TGTGGTGCCAGGGGCAGCTTGGTGTGTGAGGGGAAGGAGGGTTCCCACTC-CTGATGGGCCCCGG  451
Pongo abelii ADIB         389  TGTGGTGCCAGGGGCAGCTTGGTGTGTGAGGGGAAGGAGGGTTCCCACTC-CTGCTGGGCCCCAG  452
Nomascus leucogenys ADIB  388  TGTGGTGCCAGGGGCAGCTTGGTGTGTGAGGGGTAGGAGGGTTCCCACTC-CTGCTCGGCCCCTG  451
Callithrix jacchus ADIB   367  CGTGGTGCCAGGG-CAGCTTGCTGTGTGAGGGGAAGGAGGGCTCCCACTCCCTGCTGGGCCCCGG  430

                                   460       470       480       490       500       510       520
Homo sapiens ADIB         452  GGCCTGGGCTGAAGAAAAGGCCCCACCATCCATCCATGGTGAGGCTCCTGGGGGAGGAAGGAAGG  516
Pan troglodytes ADIB      452  GGCCTGGGCTGAAGAAAAGGCCCCACCATCCATCCATGGTGAGGCTCCTGGGGGAGGCAGGAAGG  516
Pongo abelii ADIB         453  GGCCTGGGCTGAAGAAGAGGTCCCACCATCCATCCATGGTGAGGCTCCTGGGGGAGGCAGGAAGG  517
Nomascus leucogenys ADIB  452  GGCCTGGGCTGAAGAAGAGGCCCCACCATCCATCCATGGTGAGGCTCCTGGGGGAGGCAGGAAGG  516
Callithrix jacchus ADIB   431  GACCTGGGCTTAAGAAGAGGTGTCACCATCCATCCATGGTGAGGCTCCCGGGGGAGGCAGGGAGG  495

                                        530       540       550       560       570       580
Homo sapiens ADIB         517  CGAGCTCCCGAGGGCTGAGGAGGACGGGCCCTGGGGAATGAGAGGGT--------TGGGGGCAGG  573
Pan troglodytes ADIB      517  CGAGCTCCCGAGGGCTGAGGAGGACGGGCCCCGGGGAATGAGAGGGT--------TGGGGGCAGG  573
Pongo abelii ADIB         518  CGAGCTCCCGAGGGCTGAGGAGGACGGGCCCTGGGGAATGACAGGGT--------TCGGGACAGG  574
Nomascus leucogenys ADIB  517  CGAGCTC-TGAGGGCTGAGGAGGACGGGCCCTGGGGAATGAGAGGGT--------TGGGGGCAGG  572
Callithrix jacchus ADIB   496  CAGGATCCCGAGAGCTGGGGAGGACGAGCCCTGGGGAATGAGAGGGTTGGCAGGGTGGGGGAAGG  560
```

```
                                590        600        610        620        630
Homo sapiens ADIB          574 TGAGGGGCTGGCGGGGACAGCTCAGCTCTCTCCCTCCCAGTTCCTTCTCCGGG 626 (-1 bp)
Pan troglodytes ADIB       574 TGAGGGGCTGGCGGGGACAGCTCAGCTCTCTCCCTCCCAGTTCCTTCTCCGGG 626 (-1 bp)
Pongo abelii ADIB          575 CAAGGGGCTGGCAGGGACAGCTCAGCTCTCTCCCTCCCAGTTCCTTCTCCGGG 627 (-1 bp)
Nomascus leucogenys ADIB   573 TGAGGGGCTGGCGGGGACAGCTCAGCTCTCTCCCTCCCAGTGCCTTCTCCGGG 625 (-1 bp)
Callithrix jacchus ADIB    561 TGAGAGGCTG--GGGGCCAGATCAGCTCTCTCCCTCCCAGCTCCTTCTCCGGG 611 (-1 bp)
```

B

```
                                   10        20        30        40        50        60
Homo sapiens ADIC         1 GATTACCTGTGGGAGTGTATGAATGTGTGTGTCCGTGCAAGTGAGGGACAGGGTCTATTTGGGTA  65
Macaca mulatta ADIC       1 GATCACCTGTGGGAGTGTATGAATG-GTGTGTCCATGTGAGTGAGGGACAGAGTCTATTTGGGTA  64
Papio hamadryas ADIC      1 GATCACCTGTGGGAGTGTATGAATGTGTGTGTCCGTGTGAGTGAGGGACAGAGTCTATTTGGGTA  65
Callithrix jacchus ADIC   1 GATCACCTGTG-GAGTGTATGAATGTGTGTGTCGGTGCGAGTGAGGGACAGAGTCTATTTGTGTA  64

                               70        80        90       100       110       120       130
Homo sapiens ADIC        66 TCAGTTGTGTGTCTAGGG-GGGTATAGTGGATTTCTGAGTTTGCCTACTGTGTTTTGTAGGTGCG 129
Macaca mulatta ADIC      65 TCAGTTGTGTGTCTAGGG-GGGTTTAGTGGATTTCCAAGTTTGCCTACTGTGTTTTGTAGGTACA 128
Papio hamadryas ADIC     66 TCAGTTGTGTGTCTAGGGGGGGTTTAGTGGATTTCCGAGTTTGCCTACTGTGTTTTGTAGGTGCA 130
Callithrix jacchus ADIC  65 TCAGTTGTGTGTCTAGGG-GTGTGTAGTGGATTTCTGAGTTTCCATATCGTGTTTTGTAGGTGTG 128

                                  140       150       160       170       180       190
Homo sapiens ADIC       130 TGGATGAGAG-CTGTGTTTGT--GTGAGTGTGTGAAGATGTGGGTGTGCTCTGTTGCATGTGTGG 191
Macaca mulatta ADIC     129 TGTATGAGAGACTGTGTTTGT--GTGAATGTGTGATGATGTGGGTGTGCTTGGTTACATGTGTGG 191
Papio hamadryas ADIC    131 TGTGTGAGAGACTGTGTTTGT--GTGAATGTGTGATGATGTGGGTGTGCTTGGTTACATGTGTGG 193
Callithrix jacchus ADIC 129 TGGGTGAGAGGCTGTGTTTGTTTATGAGTGTGTGATGAGGTGAGTGTGCTTGGTTGCATGTG--- 190

                             200       210       220       230       240       250       260
Homo sapiens ADIC       192 ATGTGTGTGAGTTTGTGGTTCTGTGTATATGCGTGGGGTC-CTGGGCTGGATTGAGAGTGGACA 255
Macaca mulatta ADIC     192 ATGTGTGTGAGTTTGTGGTTCTGTGTATATGTGTGGGGTCCCTGGCGCTGGATTGAGAGTGGACA 256
Papio hamadryas ADIC    194 ATGTGTGTGAGTTTGTGGTTCTGTGTATATGTGTGGGGTCCCTGGCACTGGATTGAGAGTGGACA 258
Callithrix jacchus ADIC 191 ---TGTGTAAGTTTGTGGTTCTTTGTATATGTGTGGGGTCCCTGGAGCTTGATTGAGAGTGGACG 252

                                 270       280       290       300       310       320
Homo sapiens ADIC       256 TTGAGAGCCCCAGAGGGTGCATGTGCACTTGGGGAGGACTGTGCATATATCATTGTGTGCATGGG 320
Macaca mulatta ADIC     257 TTGAGAGCCCCGGAGGGTGCATGTGCACTGGGGGAGGTCTGTGCATATATCATTGTGTGCATGGG 321
Papio hamadryas ADIC    259 TTGAGAGCCCCGGAGGGTGCATGTGCACTGGGGGAGGGCTGTGCATATATCATTGTGTGCATGGG 323
Callithrix jacchus ADIC 253 CTGAGAGCCCTGGAGGGTGCGTGTGCATTGGGAGAGGGGTGTGCATACATCATGGTGTGCATGGG 317

                             330       340       350       360       370       380       390
Homo sapiens ADIC       321 ACTCAAGGGTGGGAGCTGGGTGTGAG--TGTGATGTCCAACCTGCCCAGGCCCTCCCGTGTCTCC 383
Macaca mulatta ADIC     322 ACTCAAGGGTGTGAGCTGGGTGTGAG--TGTGATGTCCAGCCTGTCCAGGCCCTCCCGTGTCTCC 384
Papio hamadryas ADIC    324 ACTCAAGGGTGTGAGCTGGGTGTGAG--TGTGATGTCCAGCCTGTCCAGGCCCTCCCGTGTCTCC 386
Callithrix jacchus ADIC 318 ACCCAAGGGTGTGAGCTGGGTGTGAGTATGTGATGTCCAGCCTGCCCAGGCTCTTCCGTGTCTCC 382

                               400
Homo sapiens ADIC       384 ACAGAGGCATC 394 (-1 bp)
Macaca mulatta ADIC     385 ACAGGGACAAC 395 (-1 bp)
Papio hamadryas ADIC    387 ACAGGGACAAC 397 (-1 bp)
Callithrix jacchus ADIC 383 ACAGGGGCATC 393 (-1 bp)
```

C

```
                              10        20        30        40        50        60
Homo sapiens ADIE          1 GTCTGCCTCTAGAACTCTCCACCCTCATCTCTCTGCGTATTTCTCCCTGAAATGGGGTCTGGTCC  65
Pan troglodytes ADIE       1 GTCTGCCTCTAGAACTCTCCACCCTCATCTCTCTGCGTATTTCTCCCTGAAATGGGGTCTGGTCC  65
Pongo abelii ADIE          1 GTCTGCCTCCAGAACTCTCCACCCTCATCTCTCTGCGTATTTCTCCCTGAAATGGAGTCTGGTCC  65
Papio hamadryas ADIE       1 GTCTGCCTCTAGAACTCTCCACCCTCATCTCCCTACGTATTTCTCCCTGAAATGGGGTCTGGTCC  65
Callithrix jacchus ADIE    1 GTCTGCCTCTAGAATTCTCCACCCTCATCTCTCTGCTTATTTCTCTCTGGAAGGGGGTCTGATCT  65
```

```
                          70        80        90       100       110       120       130
Homo sapiens ADIE         66 TTGGTCTCTGCCACTGCCCTGCCTCTCCTCTGGCC-CTGGGAACAGGAGGTGCCCTGTGTGTCCG 129
Pan troglodytes ADIE      66 TTGGTCTCTGCCACTGCCCCGCCTCTCCTCTGGCC-CTGGGAACAGGAGGTGCCCTGTGTGTCCG 129
Pongo abelii ADIE         66 TTGGTCTCTGCCACTGCCCCGCCTCTCCTCTGGCC-CTGGGAACAGGAGGTGCCCTGTGTGTCCG 129
Papio hamadryas ADIE      66 TTGGTCTCTGCCACTGCCCCGCCTCTCCTCCGGCC-CTGGGAGCAGGAGGTGCCCTGTGTGTCCG 129
Callithrix jacchus ADIE   66 TTGGTCTCTGCCACTGTCCAGCCTCTCCTCTGGCCCCTGGGAACCAGAGGTGCCCTATGTGTCTG 130
```

```
                                 140       150       160       170       180       190
Homo sapiens ADIE        130 TCTCTCGAAGTTCTGCCTCTCTGTGCCCAGCTCAAGTCTCTCTCCCCCTCCTTTCTCCCCCTAAA 194
Pan troglodytes ADIE     130 TCTCTCGAAGTTCTGCCTCTCTGTGCCCAGCTCAAGTCTCTCTCCCCCTCCTTTCTCCCCCTAAA 194
Pongo abelii ADIE        130 TCTCTCGAAGTTCTGCCTCTCTGTGCGCTGCTCAAGTCTCTCTCCCCCTCCTTTCTCCCCCCAAA 194
Papio hamadryas ADIE     130 TCTCTCGAAGCTCTGCCTCTCTGTGCCCAGCTCAAGTCTTTCTACCCCTCCTTTCTCCCCCTAAA 194
Callithrix jacchus ADIE  131 TCTCTCGAGGTTCTGCCTCTCTGTGCCCGGCTCAAGTCTCTCTCCCCCTCCTTTCTCCCCCCAAA 195
```

```
                            200       210       220       230       240       250       260
Homo sapiens ADIE        195 CTTTGGCCGGCCGCCGGGCGACACCACGAGTTATTTCCCAGCTATTTCCCGGTCCGGGAGCTCTT 259
Pan troglodytes ADIE     195 CTTTGGCCGGCCGCCGGGCGACACCACGAGTTATTTCCCAGCTATTTCCCGGTCCGGGAGCTCTT 259
Pongo abelii ADIE        195 CTTTGGCCGGCCGCCGGGCGACACCACGAGTTATTTCCCAGCTATTTCCCGGTCCGGGAGCTCTT 259
Papio hamadryas ADIE     195 CTTTGGCCGGCCGCCGGGCGACACCACGAGTTATTTCCCAGCTATTTCCCGGTCCGGGAGCTCTT 259
Callithrix jacchus ADIE  196 CTTTGGCCGGCCGCCGGGCGACACCACGAGTTATTTCCCAGCTATTTCCCGGTCCGGGAGCTCCT 260
```

```
                                 270       280       290       300       310       320
Homo sapiens ADIE        260 GGCCCCTGAACAACTGGTTTCCTCTTGGAGTCTGGGAGGAGGAAAGCGGAGCCGGCAGGGAGCGA 324
Pan troglodytes ADIE     260 GGCCCCTGAACAACTGGTTTCCTCTTGGAGGCTGGGAGGAGGAAAGCGGAGCCGGCAGGGAGCGA 324
Pongo abelii ADIE        260 GGCCCCTGAACAACTGGTTTCCTCTTGGAGTCTGGGAGGAGGAAAGCGGAGCCGGCAGGGAGCGA 324
Papio hamadryas ADIE     260 GGCCCCCGAACAACTGGTTTCCTCTTGGAGTCTGGGAGGAGAAGAGCGGAGCCGGCAGGCAGCGA 324
Callithrix jacchus ADIE  261 GGCCCCTGAACAACTGGTTTCCTCTTGGAGTCTGGGAGGAGGAGAGCAGAGCCGGCAGGGAGCGA 325
```

```
                            330       340       350       360       370       380       390
Homo sapiens ADIE        325 ACCAGGACTGGGGTGACGGCAGGGCAGGGGGCGCCTGGCCGGGGAGAAGCGCGGGGGCTGGAGCA 389
Pan troglodytes ADIE     325 ACCAGGACTGGGGTGACGGCAGGGCAGGGGGCGCCTGGCCGGGGAGAAGCGCGGGGGCTGGAGCA 389
Pongo abelii ADIE        325 ACCAGGATTGGGGTGACGGCAGGGCAGGGGGCGCCTGGCCGGGGAGAAGCGCGGGGGCTGGAGCA 389
Papio hamadryas ADIE     325 ACCAGGACTGGGGTGACGGCAGGGCAGGGGGCGCCTCGCCGGGGAGAGGCGCGGGGGCTGGAGCA 389
Callithrix jacchus ADIE  326 ACCAGGACCGGAGTGACCTCGGGGAAGGGGGCGCCTGGCCGGGGAGAAGCGCGGGGGCGGGGGCA 390
```

```
                                 400       410       420       430       440       450
Homo sapiens ADIE        390 CCACCAACTGGAGGGTCCGGAGTAGCGAGCGCC-CCGAAGGAGGCCATCGGGGAGCCGGGAGGGG 453
Pan troglodytes ADIE     390 CCACCAACTGGAGGGTCCGGAGTAGCGAGCGCC-CCGAAGGAGGCCATCGGGGAGCCGGGACGGG 453
Pongo abelii ADIE        390 CCACCAACTGGAGGGTCCGGAGTAGCGAGCGCC-CCGAAGGAGGCCATCGGGGAGCCGGGAGGGG 453
Papio hamadryas ADIE     390 CCACCGACTGGAGGGTCCGGAGTAGCGAGCGCC-CCGAAGGAGGCCATCAGGGAGCCGGGAGGCG 453
Callithrix jacchus ADIE  391 CCGCCTACTGGAGGGTCCGGAGTAGGTAGCGCCCCCGAAGGAGGCCATTGGGGAGCCAGGAGG-G 454
```

```
                            460       470       480       490       500       510       520
Homo sapiens ADIE        454 GGGTGAGTCTGGCGGAGGAGGGGAAAGGGGCTGGGGAGGGGACTGTGGGAAAGGCTGGCGCGGGC 518
Pan troglodytes ADIE     454 GGGTGAGTCTGGCGGAGGAGGGGAAAGGGGCTGGGGAGGGGACTGTGGGAAAGGCTGGCGCGGGC 518
Pongo abelii ADIE        454 GGGTGAGTCTGGCGGAGGAGGGGAAAGGGGCTGGGGAGGGGACTGTGGGAAAGGCTGGCGCGGGC 518
Papio hamadryas ADIE     454 GGGTGAGTCTGGCGGAGGAGGGGAAAGGGGCTGGGGAGGGGACTGTGGGAAAGGCTGGCGCGGGC 518
Callithrix jacchus ADIE  455 GGGTGAGTCTGGCGGAGGAGGGCAAAGGGGCTGGGGAGGAGACAGTGGGAAAGGCTGGCGCAGGC 519
```

```
                                 530       540       550       560       570       580
Homo sapiens ADIE        519 CGGCGGTGGAGGGAGGGCTGGCCGCTCCCACCCTCTCGCGCCTGCCTGTGCTCTCCGGATGCTC 583
Pan troglodytes ADIE     519 CGGCGGTGGAGGGAGGGCTGGCCGCTCCCACCCTCTCTCGCCTGCCTGTGCTCTCCGGATGCTC 583
Pongo abelii ADIE        519 CAGCGGTGGAGGGAGCGCTGGCCGCTCCCACCCTCTCTCGCCTGCCTGCGCTCTCCGGGTGCTC 583
Papio hamadryas ADIE     519 CGGCGGTGGAGGGAGGGCTGGCCGCTCCTACCCGCTCTCGCCTGCCTGTGCTCTTCGGGTGTTC 583
Callithrix jacchus ADIE  520 CAGCGGTGGAGGGAAAACTGACCGCTCCTACCCGCTCTCGCCTGCCTG-GCTCACCGGGTGCTC 583
```

```
                                  590       600       610       620       630       640       650
Homo sapiens ADIE          584 GGCCCTGCGAGCGCTCAGCCTCAGGGGCACGGCAGTGGGGTGGGGACCGACTGGGCCGCGCCGGG 648
Pan troglodytes ADIE       584 GGCCCTGCGAGCGCTCAGCCTCAGGGGCACGGCAGTGGGGTGGGGACCGACTGGGCCGCGCCGGG 648
Pongo abelii ADIE          584 GGCCCTGCGAGCGCTCAGCCTCAGGGGCACGGCAGTGGGGTGGGGACCGACTGGGCAGCGCCGGG 648
Papio hamadryas ADIE       584 GGCCCTGTGGGCGCTCAGCCTCAGGGGCACAGCAGTGGGGTGGGGACCGACTGGGCAGCGCCGGG 648
Callithrix jacchus ADIE    584 GGGTCCGCGGGCACTCAGCCTCAGGGGAACGGCAGTGGGGTGGGGACCAACTGGGCCGCGCCGGG 648

                                       660       670       680       690       700       710
Homo sapiens ADIE          649 GTGGGGAGAGAGGCAGGTCCCAGGATTCTGGGTCCCGACTCCAGTCCTGCCTGGTCCACAGACTG 713
Pan troglodytes ADIE       649 GTGGGGAGAGAGGCAGGTCCCAGGATTCTGGGTCCCGACTCCAGTCCTGCCTGGTCCACAGACTG 713
Pongo abelii ADIE          649 GTGGGGAGCGAGGCAGGTCCCAGGATTCTGGGTCCCAACTCCAGTCCTGCCTGGTCCACAGACTG 713
Papio hamadryas ADIE       649 GTGGGGATCGAGGTAGGTCCCAGGATTCTGGGTCCCGACTCCAGTCCTGCCTGGCCCACAGACTG 713
Callithrix jacchus ADIE    649 GTGGGGAGCGAGGCAGGTCCCAGGATTCTGGGTCCCGACTCCAGTCCTGGCTGGCCCACAGACTG 713

                                  720       730       740       750
Homo sapiens ADIE          714 CGAGAGGACCCCGGCGTCCGGGCTCCCGGTGCCAGCGCT 752 (-1 bp)
Pan troglodytes ADIE       714 CGAGAGGACCCCGGCGTCCGGGCTCCCGGTGCCAGCGCT 752 (-1 bp)
Pongo abelii ADIE          714 CGAGAGGACCCCGGCGTCCGGGCTCCCGGTGCCAGCGCT 752 (-1 bp)
Papio hamadryas ADIE       714 CGAGAGGACCCCGGCGTTCGGGCTCCCGGTGCCAGCGCT 752 (-1 bp)
Callithrix jacchus ADIE    714 CCAGAGGACCCCGGCGTCCGGGCTCCCGGTGCCAGCGCC 752 (-1 bp)
```

D

```
                                        10        20        30        40        50        60
Homo sapiens ADIF1              1 TATCGTGATAAAATTTGATCTGTACGACAGTCCTGTGAGATAAGTAAGCATTGATCTCTGGTTCA  65
Homo sapiens ADIF2              1 TATTGTGATAAAATTTGATTTTACAATAGTCCTGTGAGATAAGTAAGCATTGGTCTGTGGTTAA  65
Pan troglodytes ADIF1           1 TATTGTGATAAAATTTGATCTGTACGACAGTCCTGTGAGATAAGTAAGCATTGATCTCTGGTTCA  65
Pongo abelii ADIF1              1 TATTGTGATAAAATTTGATCTTTACAACAGTCCTGTGAGATAAGTAAGCATTGATCTCTGGTTCA  65
Nomascus leucogenys ADIF1       1 TATTGTGATAAAATTTGATGTTTACAACAGTCCTGTGAGATAAGTAAGCATTGATCTCTGGTTCA  65

                                     70        80        90       100       110       120       130
Homo sapiens ADIF1             66 GAAAAGTGATTTCTTCAAAGTGAAATGATGAACGGAAGAGTATCCTGACCTTAAACTTGGCTTTT 130
Homo sapiens ADIF2             66 GAGAAGTGATTTCTTCAAAGTGAAATGATGAACGGAAGAGTATCCTGACCTTAAACTTGGCTTTT 130
Pan troglodytes ADIF1          66 GAAAAGTGATTTCTTCAAAGTGAAATGATGAACGGAAGAGTATCCTGACCTTAAACTTCGCTTTT 130
Pongo abelii ADIF1             66 GAAAAGTGATTTCTTCAAAGTGAACTGATGAATGGAAGAGTATCCTGACCTTAAACTTGGCTTTT 130
Nomascus leucogenys ADIF1      66 GAAAACTGATTTCTTCAAAGTGAAATGATGAATGGAAGAGTATCCCGACCTTAAACTTGGCTTTT 130

                                         140       150       160       170       180       190
Homo sapiens ADIF1            131 -TAAATTGCCCGTTCACCTTGAAGAGTGGTTCCAATCACAAGTG--TTACCTTCCAAGGACAGTG 192
Homo sapiens ADIF2            131 TTTTATTGCCCGTTCAGCTTGAAGAGTGGTTCCAATCACAAGTG--TTACCTTCCAAGGACAATG 193
Pan troglodytes ADIF1         131 -TTAATTGCCCGTTCACCTTGAAGAGTGGTTCCAATCACAAGTG--TTACCTTCCAAGGACAATG 192
Pongo abelii ADIF1            131 -TTTATTGCCCGTTCACCTTGAAGAGTGGTTCCAATCACAAGTGTGTTACCTTCCAAGGACAATG 194
Nomascus leucogenys ADIF1     131 -TTTATTGCCCGTTCACCTTGAAGAGTGGTTCCAATCACAAGCG--TTACCTTCCAAGGACAATG 192

                                     200       210       220       230       240       250       260
Homo sapiens ADIF1            193 TCATTCTGGTTATAATCAGCTTGCTCCCCTGCCCTGAGCCTTCTGTCCAAGTTTGTGCAGGTGTG 257
Homo sapiens ADIF2            194 TCATCCTGGTAATAACCAGCTTGCTCCCCTGCCCTGAGTCTTCTG-CCAAGTCTGTCCAGGTGTG 257
Pan troglodytes ADIF1         193 TCATTCTGGTTATAATCAGCTTGCTCCCCTGCCCTGAGCCTTCTGTCCAAGTCTGTGCAGGTGTG 257
Pongo abelii ADIF1            195 TCATTCTAGTTATAATCAGCTTGCTCCCCTGTCCTGAGCCTTCTGTCCAAGTCTGTCCAGGTGTG 259
Nomascus leucogenys ADIF1     193 CTATCCTGGTTATAACCAGCTTGCTCTCCTGCCCTGAGCCTTCTGTCCGAGTCTGTCCAGGTGTG 257

                                         270       280       290       300       310       320
Homo sapiens ADIF1            258 GGTCTGGGGCAGGGGACAGCTCATCCATTTCCCACTGCACGGAACAGAGGCTGTGTCCAGGGCCC 322
Homo sapiens ADIF2            258 GGTCTGGGGCAGGGGACAGCTCATCCATTTCCCACTGCACAGAACGGAGGCTGTGTCCAGGGCCC 322
Pan troglodytes ADIF1         258 GGTCTGGGGCAGGGGACAGCTCACCCATTTCCCACTGCACGGAAGAGAGGCTGTGTCCAGGGCCC 322
Pongo abelii ADIF1            260 GGTCTGGGGCAGGGGACAGCTCACCCATTTCCCACTGCACGAAACAGAGGCTGTGTCCAAGGCGC 324
Nomascus leucogenys ADIF1     258 GGCCTGGGACAGGGGACAGCTCATCCATTTCCCACTGCACGGAACAGAGGCTGTGTCCAGGGCCC 322

                                     330       340       350       360       370       380       390
Homo sapiens ADIF1            323 CAGCAACAAAGATTTCTCCTTTGGGTTTCTGTTTCCCTCTTTCAGTTCAGAGTCTGTCATCTGAA 387
Homo sapiens ADIF2            323 CAGCAACAAAGGTTTCTCCTCTGGGTTTCTGTTTCCCTCTTTCAGTTCAG--TCTGTCATCTGAA 385
Pan troglodytes ADIF1         323 CAGCAACAAAGGTTTCTCCTCTGGGTTTCTGTTTCCCTCTTTCAGTTCAGAGTCTGTCATCTGAA 387
Pongo abelii ADIF1            325 CAGCAACAAAGGTTTCTCCTTTGGGTTTCTGTTTCCCTCTTTCAGTTCAGAGTCTGTCATCTGAA 389
Nomascus leucogenys ADIF1     323 CAGCAACAAAGGTTTCTCCTTTGGGTTTCTGTTTCCCTCTTTCAGTTCAGAGTCTGTCATCTGAA 387

Homo sapiens ADIF1            388 CC 389 (-1 bp)
Homo sapiens ADIF2            386 CC 387 (-1 bp)
Pan troglodytes ADIF1         388 CC 389 (-1 bp)
Pongo abelii ADIF1            390 CC 391 (-1 bp)
Nomascus leucogenys ADIF1     388 CC 389 (-1 bp)
```

E

```
                                   10        20        30        40        50        60
Homo sapiens ADIG          1 AATGTAAATCTCAAGGTGGTAAAAATAATGTTGATGTTTATCTTTTTCATTTCCAGGTCTAAATT  65
Pan troglodytes ADIG       1 AATGTAAATCTCAAGGTGGTAAAAATAATGTTGATGTTTATCTTTTTCATTTCCAGGTCTAAATT  65
Pongo abelii ADIG          1 AATGTAAATCTCAAGGTGGTAAAAATAATGTTGATGTTTATCTTTTTCATTTCCAGGTCTAAATT  65
Callithrix jacchus ADIG    1 AATGCAAATCTCAAGGTGGTAAAAATAATATTGATGTTTGTCTTTTTCAATTCCATGTCTAAATT  65

                                 70        80        90       100       110       120       130
Homo sapiens ADIG         66 TTTTTCTTTAAAAAATGCACTATGTTTGTAAAAAGAAAATGCCATTTTTAAATCCATCATTGATT 130
Pan troglodytes ADIG      66 TTTTTCTTTAAAAAATGCACTACATTTGTAAAAAGAAAATGCCATTTTTAAATCCATCATTGATT 130
Pongo abelii ADIG         66 TTTTTCTTTAAAAAATGCACTACTTTTGCAAAAAGAAAATGCCATTTTTAAGTCCATCATTGATT 130
Callithrix jacchus ADIG   66 TTATTCTTTGAAAAATGTGCTACTTTTGCAA-AAGAAAATGCCATTTTTAAATCCATCATTTATT 129

                                    140       150       160       170       180       190
Homo sapiens ADIG        131 TCTGAAGAATAAAT-----GCAGTGATTTATTTTGAAATGTTTTTAACCAATTCTTCATCTGCAC 190
Pan troglodytes ADIG     131 TCTGAAGAATAAAT-----GCAGTGATTTATTTTGAAATGTTTTTAACCAATTCTTCATCCGCAC 190
Pongo abelii ADIG        131 TCTGAAGAATAAAT-----GCAGTGATTTATTTTGAAATGATTTTAACCAATTCTTCATCTGCAC 190
Callithrix jacchus ADIG  130 TCTGAAGAATAAATACAATGCAGTGGTTTATTTTGAAATGTTTGTAACCAATTCTTCATCTGCAC 194

                               200       210       220       230       240       250
Homo sapiens ADIG        191 CAACTCCCGTGTAGGGCACTGAGGATTCTATATCAATAATGTGTTGATAATTTTCAATGGAATT 254
Pan troglodytes ADIG     191 CAACTCCCGTGTAGGGCACTGAGGATTCTATATCAATAATGTGTTGATAATTTTCAATGGAATT 254
Pongo abelii ADIG        191 CAACTCCCGTGTAGGGCACTGAGGATTCTATGTCAATAATGTGTTAATAATTTTCAATGGAATT 254
Callithrix jacchus ADIG  195 CAACTCCCGTGTAGGGCACTGCAGATTCTATATCAATAATGTGTTAATAATTTTCACTGGAATT 258

Homo sapiens ADIG        (-1 bp)
Pan troglodytes ADIG     (-1 bp)
Pongo abelii ADIG        (-1 bp)
Callithrix jacchus ADIG  (-23 bp)
```

F

```
                                     10        20        30        40        50        60
Homo sapiens ADIH            1 GATGATGAATGCCTCACCCCCGAGACCCCTTCGAAGTGTATTCTGGGTAATGACTCCACTCACCC  65
Pan troglodytes ADIH         1 GTTGATGAATGCCTCAACCCCGAGACCCCTTCGAAGTGTATTCTGGGTAATGACTCCACTCACCC  65
Pongo abelii ADIH            1 GATGATGAATGCCTCACCCCCGAGACCCCTTCAAAGTGTATTCTGGGTAATGACTCCACTCACCC  65
Nomascus leucogenys ADIH     1 GATGATGAGTGCCTCACCCCCGAGACCCCTTCAAAGTGTATTCTGGGTAATGACTCCACTCACCC  65
Macaca mulatta ADIH          1 GATGATGAGTGCCTCACCCCCGAGACCCCTTCAAAGTGTATTCTGGGTAATGACTCCACTCACCC  65

                                  70        80        90       100       110       120       130
Homo sapiens ADIH           66 AAATATGTCCTCAAGATATGACGACTCTGTCCACATCACATCTACTTGTATGTGATGCAGGAAAG 130
Pan troglodytes ADIH        66 AAATATGTCCTCAAGATATGACGACTCTGTCCACATCACATCTACTTGTATGTGATGCAGGAAAG 130
Pongo abelii ADIH           66 AAATACGTCCTCAAGATATGATGACTCTGTCCACATCACATCTACTTGTATGTGATGCAGGAAAG 130
Nomascus leucogenys ADIH    66 AAATACGTCCTCAAGATATGACAACTCTGTCCACATCACATCTACTTGTATGTGATGCAGGAAAG 130
Macaca mulatta ADIH         66 AAATACTTCCTCAAGATATGACGACTCTGTCCACATCACATCTACTTGAATGTGATGCAGGAAAG 130

                                     140       150       160       170       180       190
Homo sapiens ADIH          131 AGCAAGAGACAGAAATCCAACTGAGTGTATCCCAGCTGCCTCCTCCTGTGCATCCCCTCTGACCA 195
Pan troglodytes ADIH       131 AGCAAGAGACAGAAATCCAACTGAGTGTATCCCAGCTGCCTCCTCCTGTGCATCCCCTCTGACCA 195
Pongo abelii ADIH          131 AGCAAGAGACAGAAATCCAACTGAGTGTATCCCAGCTGCCTCCTCCTGTGCATCCCCTCTGACCA 195
Nomascus leucogenys ADIH   131 AGCAAGAAACAGCAATCCAACTGAGTGTATCCCAGCTGCCTCCTTCTGTGCATCCCCTCTGACCA 195
Macaca mulatta ADIH        131 AGCAAGAGACAGAAATCCAGTTGAGTGTATCCCAACTGCCTCCTCCTGTGCATCCCCTCTGACCA 195

                                200       210       220       230       240       250       260
Homo sapiens ADIH          196 GCCCCAGGTCAGCTTGGTGGCCACACTCACCTGTCACAGCTCTCTTGCCTCCCACAGGTAACCAC 260
Pan troglodytes ADIH       196 GCCCCAGGTCAGCTTGGTGGCCACACTCACCTGTCACAGCTCTCTTGCCTCCCACAGGTAACCAC 260
Pongo abelii ADIH          196 GCCCCAGGTCAGCTTGGTGGCCACACTCACCTGTCACAGCTCTCTTGCTTCCCACAGGTGACCAC 260
Nomascus leucogenys ADIH   196 GCCCCAGGTCAGCTTGGTGGCCACACTCACCTGTCACAGCTCTCTTGCCTCCCACAGGTGACCAC 260
Macaca mulatta ADIH        196 GACCCAGGCCAGCTTGGTGGCCACACTCACCTGTCACAGCTCTCTTGCCTCCCACAGGTGACCAC 260

Homo sapiens ADIH          261 C 261 (-1 bp)
Pan troglodytes ADIH       261 C 261 (-1 bp)
Pongo abelii ADIH          261 C 261 (-1 bp)
Nomascus leucogenys ADIH   261 C 261 (-1 bp)
Macaca mulatta ADIH        261 C 261 (-1 bp)
```

G

```
                                10        20        30        40        50        60
Homo sapiens ADII      1 TCGTCTTGTGTGCCCCACGTCATGCGCGCGGGCGTCCGCGGCTCTCCCGACCAGCCCCAGCGCGG  65
Pongo abelii ADII      1 TCGTCTTGTGTGCCCCACGTCATGCGCGCCGGCGTCCGCGGCTCTCCCGACCAGCCCCAGCGCGG  65
Macaca mulatta ADII    1 TTGTCTTGTGTGCCCCACGCCATGCGCGCCGGGGTCCGCGGCTCTTCCGAGCAGCCCCAGCGCGG  65

                             70        80        90       100       110       120       130
Homo sapiens ADII     66 TGGGCCCAGGCTCAGAGGTCCCGGCGCCTCTCGCTGAAGTAGTTGGGTAGCCGGGGCTGGGGGTC 130
Pongo abelii ADII     66 TGGGCCCAAGCGCAGAGGTCCCGGCGCCTCTCGCTGAAGTAGTTGGGTGGCCGGGCCGGGGGGTC 130
Macaca mulatta ADII   66 TGGGCCCAGGCGCCGAGGTCCCGGCGCCTCTCGCTGAAGTAGTTGGGTGGCCGGGGCGGGGGGTC 130

                                140       150       160       170       180       190
Homo sapiens ADII    131 GCCACGTCGGGGGCGCGGCCAGGACCCGCGGAGCCGGTCCCCGAGCGCGGGGAGCGGGGCCGCCC 195
Pongo abelii ADII    131 GCCACGTCGGGGGCGCGGCCAGGACCCGCGGAGCCGGCCCCCGAGCGCGGGGAGCGGGGCCGCCC 195
Macaca mulatta ADII  131 GCCACGTCGGGGGCGCGGCCAGGACCCGCGGAGCCGGCCCCCGAGCACGGGGAGCGGGGCCGCCC 195

                           200       210       220       230       240       250       260
Homo sapiens ADII    196 GCGCCGCCCCACCATTACCTCCCCGGGCGGCAAGGAGGAGCTGGTGGCGGTCGCCTCCCGGCTGT 260
Pongo abelii ADII    196 GCGCCGCCCCACCATTACCTCCCCGGGCGGCAAGGAGGAGCTGGTGGCGGTCGCCTCCCGGCTGT 260
Macaca mulatta ADII  196 GCGCCGCCCCACCATTACCTCCCCGGGCGGCAAGGAGGAGCTGGTGGCGGTCGCCTCCCGGCTGT 260

                                270       280       290       300       310
Homo sapiens ADII    261 GGCAGCGGCGGCGGCGTGCCTGCCTGGCGGCCGTCGGCGTACTCTTGGCC 310 (-1 bp)
Pongo abelii ADII    261 GGCAGCGGCGGCGGCGTGCCTGCCTGGCGGCCGTCGGCGTACTCTTGGCC 310 (-1 bp)
Macaca mulatta ADII  261 GGCAGCGGCGGCGGCGTGCCTGCCTGGCGGCCGTCGGCGTACTCCTGGCC 310 (-1 bp)
```

H

```
                                10        20        30        40        50        60
Homo sapiens ADIJ        1 TCCCACATTGTCAGAGTAATGCCAAACTCTCTCTGAGTGGGATGAGCAGAGCAGATGCTGCAATG  65
Pan troglodytes ADIJ     1 TCCCACATTGTCAGAGTAATGCCAAACTCTCTCTGAGTGGGATGAGCAGAGCAGATGCTGCAATG  65
Papio hamadryas ADIJ     1 TCCCACATTGTCAGAGTAATGCCAAACTCTCTCTGAGTGGGATGAGCAGAGCAGATGCTGCAATG  65

                             70        80        90       100       110       120       130
Homo sapiens ADIJ       66 AGATGCCAAAGCGGCTCCCCTTCCTCTGTGCCTTGGGTGCCTATAAATTGCTCCGGCGCGCGTTT 130
Pan troglodytes ADIJ    66 AGATGCCAAAGCGGCTCCCCTTCCTCTGTGCCTTGGGTGCCTATAAATTGCTCCGGCGCGCGTTT 130
Papio hamadryas ADIJ    66 AGATGCCAAAGCGGCTCCCCTTCCTCTGTGCCTTGGGTGCCTATAAATTGCTCCGGCGCGCGTTT 130

                                140       150       160       170       180       190
Homo sapiens ADIJ      131 GTCAGCCTCCTCTTCTCCTGGCAGGTGGTACCCAGGCAGAATTCTGCCTTCAGTCTCTCTCTCGC 195
Pan troglodytes ADIJ   131 GTCAGCCTCCTCTTCTCCTGGCAGGTGGTACCCAGGCAGAATTCTGCCTTCAGTCTCTCTCTCGC 195
Papio hamadryas ADIJ   131 GTCAGCCTCCTCTTCTCCTGGCAGGTGGTACCCAGGCAGAATTCTGCCTTCAGTCTCTCTCTCGC 195

                             200       210       220       230       240       250       260
Homo sapiens ADIJ      196 TCCGCTCCCGGCCGTGAGGCGCTCGCCGCTGCTCGCTCGCTCCTCCGCCCCAGCTCTGAGCCTCG 260
Pan troglodytes ADIJ   196 TCCGCTCCCGGCCGTGAGGCGCTCGCCGCTGCTCGCTCGCTCCTCCGCCCCAGCTCTGAGCCTCG 260
Papio hamadryas ADIJ   196 TCCGCTCCCGGCCGTGAGGCGCTCGCCGCTGCTCGCTCGCTCCTCCACCCCAGCCCCGAGCCTCG 260

                                270       280       290       300       310       320
Homo sapiens ADIJ      261 CCGTGCCGACCGTGCCCGCCGCCGCCGCCGCTGGGCGCACCCGGGGGACGCCCGGGCCCACGCGG 325
Pan troglodytes ADIJ   261 CCGTGCCGACCGTGCCCGCCGCCGCCGCCGCTGGGCGCACCCGGGGGACGCCCGGGCCCACGCGG 325
Papio hamadryas ADIJ   261 CCGAGCCTACAGTGCCCGCCGCCGCCGCCGCTGGGCGCACCCGGGGGACGCCCGGGCCCACGCGG 325

                             330       340       350       360       370       380       390
Homo sapiens ADIJ      326 GGCTTTGGGGTGCGCGTCTATTCGAGTTGTGGTGGTGGCAAAGGAGGAAGAGAGAAAGGAGGCAA 390
Pan troglodytes ADIJ   326 GGCTTTGGGGTGCGCGTCTATTCGAGTTGTGGTGGTGGCAAAGGAGGAAGAGAGAAAGGAGGCAA 390
Papio hamadryas ADIJ   326 GGCTTTAGGGTGCGCGTCTATTCGAGTTGTGGTGGTGGCAAAGGAGGAAGAGAGAAAGGAGGCAA 390

                                400       410       420       430       440       450
Homo sapiens ADIJ      391 T-AAAAA--AAAAAGGCAGCGGACGGGCGAACTGAGCGAGCGAAAGAAGAGGAGGAGGAGGCAGA 452
Pan troglodytes ADIJ   391 TTAAAAAAAAAAAAGGCAGCGGACGGGCGAACTGAGCGAGCGAAAGAAGAGGAGGAGGAGGCAGA 455
Papio hamadryas ADIJ   391 TTAAAAA--AAAAAGGCAGCGGACGGGCGAACTGAGCGAGCGAAAGAAGAGGAGGAGGAGGCAGA 453

                             460       470       480       490       500       510       520
Homo sapiens ADIJ      453 AAAAGGCAACTTCAGACGGAAAGTTGGTGCGAACAGGCGCAGTCTGCAAAAACGGAAAAGTCGAT 517
Pan troglodytes ADIJ   456 AAAAGGCAACTTCAGACGGAAAGTTGGTGCGAACTGGCGCAGTCTGCAAAAACGGAAAAGTCGAT 520
Papio hamadryas ADIJ   454 AAAAGGCAACTTCAGACGGAAAGTTGGTGCGAACTGGCGCAGTCTGCAAAAACGGAAAAGTCGAT 518

                                530       540       550       560       570       580
Homo sapiens ADIJ      518 CGCAGGCGGCGGCGGCATAAAAGTGTGAGCTGCCCGGGCGAGCTCAGGAGGCGGCGGCTGGCTCT 582
Pan troglodytes ADIJ   521 CGCAGGCGGCGGCGGCATAAAAGTGTGAGCTGCCCGGGCGAGCTCAGGAGGCGGCGGCTGGCTCT 585
Papio hamadryas ADIJ   519 CGCAGGCGGCGGCGGCATAAAAGTGTGAGCGGCCCGGGCGAGCGCAGGAGGCGGCGGCTGGCTCT 583

                             590       600       610       620       630       640       650
Homo sapiens ADIJ      583 GCCCTCCCGGTGGCCGCGCCGGCGCCGGCTGCAGCCACAGGTGCGAAGGAGCTCGCGGGGGGCGA 647
Pan troglodytes ADIJ   586 GCCCTCCCGGTGGCCGCGCCGGCGCCGGCTGCAGCCACAGGTGCGAAGGAGCTCGCGGGGGGCGA 650
Papio hamadryas ADIJ   584 GCCCTCCCGGTGGCCGCGCCGGAGCCCGCTGCAGCCACAGGTGCGAAGGAGCTCGCGGGAGGCGA 648

                                660       670       680       690       700       710
Homo sapiens ADIJ      648 GGGCGCCCCGCGCACCCCTCCCCCGGCCCCCACCCCGGGCTCGGGACTTCGGCTCAAGTCACTGG 712
Pan troglodytes ADIJ   651 GGGCGCCCCGCGCACCCCTCCCCCGGCCCCCACCCCGGGCTCGGGACTTCGGCTCAAGTCACTGG 715
Papio hamadryas ADIJ   649 GGGCGCCCCGCGCACCCCTCGCCCGACCCCCACCCCAGGCTCGGGACTTCGGCTCAAGTCACTGG 713

                             720       730       740       750       760       770       780
Homo sapiens ADIJ      713 GCGCCCGCGCTCCCTCCCCAGCCGCAGCCTCCGCGGGGGGAGCAGGAGTCGGCAGCAGCGGCCGG 777
Pan troglodytes ADIJ   716 GCGCCCGCGCTCCCTCCCCAGCCGCAGCCTCCGCGGGGGGAGCAGGAGTCGGCAGCAGCGGTCGG 780
Papio hamadryas ADIJ   714 GCGCCCGCGCTCCCTCCCCAGCCGCAGCCTCCGCGGGGGGAGCAGGAGTCGGCAGCAGCGGCCGG 778
```

```
                                       790       800       810       820       830       840
Homo sapiens ADIJ     778 CGCACGCACGCCCAGGGGAGTTGGGGTTCGCAGGGGGTTGTTTTCGGCTCTGAAGAGGTCCCCGC 842
Pan troglodytes ADIJ  781 CGCACGCACGCCCAGGGGAGTTGGGGTTCGCAGGGGGTTGTTTTCGGCTCTGAAGAGGTCCCCGC 845
Papio hamadryas ADIJ  779 CGCGCGCCCGCCCAGGGGAGTTGGGGTTCGCAGGGGGTTGTTTTCGGCTCTGAAGAGGTCCCCGC 843

                            850       860       870       880       890       900       910
Homo sapiens ADIJ     843 CCAACCTTCAAAATTCTGTCCAAAAGCAGACAAGAGGATCGCCCCGCGCTGAGCCGGCTGGTGGG 907
Pan troglodytes ADIJ  846 CCAACCTTCAAAATTCTGTCCAAAAGCAGACAAGAGGATCGCCCCGCGCTGAGCCGGCTGGTGGG 910
Papio hamadryas ADIJ  844 CCAACCTTCAAAATTCTGTCCAAAAGCAGACAAGAGGATCGCCCCGCGCTGAGCCGGCTGGTGGG 908

                                 920       930       940       950
Homo sapiens ADIJ     908 CAGCAGGAGGCGCCTGATCGCCGCCGGGGCGCTGGGGGTGGTG 950 (-1 bp)
Pan troglodytes ADIJ  911 CAGCAGGAGGCGCCTGATCGCCGCCGGGGCGCTGGGGGTGGTG 953 (-1 bp)
Papio hamadryas ADIJ  909 CAGCAGGAGGCGCCTGATCGCCGCCGGGGCGCTGGGGGTGGTG 951 (-1 bp)
```

I

```
                             10         20        30         40         50          60
Homo sapiens ADIK          1 CCCGCACTTCCCACTCTGGGCACCAGGGGTCGCTCCGCCTCTGGCCGCTTATAGTGGCTCCCGCC  65
Pan troglodytes ADIK       1 CCCGCACTTCCCACTCTGGGCACCAGGGGTCGCTCCGCCTCTGGCCGCTTATAGTGGCTCCCGCC  65
Gorilla gorilla ADIK       1 CCCGCACTTCCCACTCTGGGCACCAGGGGTCGCTCCGCCTCTGGCCGCTTATAGTGGCTCCCGCC  65
Papio hamadryas ADIK       1 CCCGCGCTTCCCACTCTGGGCACCAGGGGTCGCTCCGCCTCTGGCCGCTTATAGTGGCTCCGGCC  65
Callithrix jacchus ADIK    1 CCCGCGCTTCCCACTCTGGGCACCAGGGGTCGCTCTGCCTCCAGTCGCTTATAGTGGCTCTCGCC  65

                                70        80        90         100       110       120       130
Homo sapiens ADIK         66 GCGCGGCGCCTCATACCACCCG------CGCCCGGGAGGGAGGGGAGGAAGGTTAGGGAGGCGGA 124
Pan troglodytes ADIK      66 GCGCGGCGCCTCATACCACCCG------CGCCCGGGAGGGAGGGGAGGAAGGTTAGGGAGGCGGA 124
Gorilla gorilla ADIK      66 GCGCGGCGCCTCATACCACCCG------CGCCCGGGAGGGAGGGGAGGAAGGTTAGGGAGGCGGA 124
Papio hamadryas ADIK      66 GCTCGGCGCCTCACACCACCCGCATCGCCGCCCGGGAGGGAGGGGAGGAAGGTTAGGGACGCGGA 130
Callithrix jacchus ADIK   66 TCGCGGCGACTCACACTACCCGCATCGCCGCCCGGGAGGGAGGGGAGGAAGGTTAGGGACGCGGA 130

                                   140       150       160        170       180       190
Homo sapiens ADIK        125 GAGGGACCGCGCCCGCAGGAGAGAGGCGCGGGCCAGGGCTCTAGCAGGGACTGGGGCCGCGGCAG 189
Pan troglodytes ADIK     125 GAGGGACCGCGCCCGCAGGAGAGAGGCGCGGGCCAGGGCTCTAGCAGGGACTGGGGCCGCGGCAG 189
Gorilla gorilla ADIK     125 GAGGGACCGCGCCCGCAGGAGAGAGGCGCGGGCCAGGGCTCTAGCAGGGACTGGGGCCGCGGCAG 189
Papio hamadryas ADIK     131 GACCGACCGCGCTCGCAGGACAGAGGCGCGGGCCAGGGCTCTAGCGGGGGCTGGGGCCGCGGCAG 195
Callithrix jacchus ADIK  131 GA---------------GGAGAGAGGCACGGGCCAGGGCTCTAGCAGGGGCTGGGACCGCGGCAG 180

                              200       210       220       230        240       250       260
Homo sapiens ADIK        190 GGGTAGCAAGGTGAGTCGGTGCTTGCCAAGAGGCAGAGCGCAAAACCTACTAGGAGATCGCGCCC 254
Pan troglodytes ADIK     190 CGGTAGCAAGGTGAGTCGGTGCTTGCCAAGAGGCAGAGCGCAAAACCTACTAGGAGATCGCGCCC 254
Gorilla gorilla ADIK     190 GGGTAGCAAGGTGAGTCGGTGCTTGCCAAGAGGCAGAGCGCAAAACCTACTAGGAGATCACGCCC 254
Papio hamadryas ADIK     196 GGGTAGCAAGGTGAGTCGGTGCTTGCCAAGAGGCAGAGCGCAAAACCTACCAGGAGATCGCGCCC 260
Callithrix jacchus ADIK  181 GGGTAGCAAGGTGAGTCGGTGCTTGCCTGGAGGCAGAGCGCAAAACCTACGAGGAGATCGTGCAC 245

                                   270       280       290        300       310       320
Homo sapiens ADIK        255 G-GTGAGCAGCACCCGCAGCTCAGAGCCCGGGACGTCCGGAGCGCGGGGAGCAGTCCCCTCTCCA 318
Pan troglodytes ADIK     255 G-GTGAGCAGCACCCGCAGCTCAGAGCCCGGGACGTCCGGAGCGCGGGGAGCAGTCCCCTCTCCA 318
Gorilla gorilla ADIK     255 G-GTGAGCAGCACCCGCAGCTCAGAGCCCGGGACGTCCGGAGCGCGGGGAGCAGTCCCCTCTCCA 318
Papio hamadryas ADIK     261 G-GTGAGCAGCACCCACAGCTCAGACCCCGGGACGTCCGGAGCGCGGGGAGTAGTCCCCGCTCCA 324
Callithrix jacchus ADIK  246 GGGTGGACAGCACCCGCAGCTCAGAGCCTGGGACGTCCGGAGAGCGGGGAGTAGTCCCGGCTCCA 310

                              330       340       350        360       370       380       390
Homo sapiens ADIK        319 TCAGGGAGTGGTCTATCTGGGCAGTCTGGGACCCAGGCACCGCGCCATCCCTGAGAGAGCAGCAG 383
Pan troglodytes ADIK     319 TCAGGGAGTGGTCTATCTGGGCAGTCTGGGACCCAGGCACCGCACCATCCCTGAGAGAGCAGCGG 383
Gorilla gorilla ADIK     319 TCAGGGAGTGGTCTACCTGGGCAGTCTGGGACCCAGGCACCGCGCCATCCCTGAGAGAGCAGCGG 383
Papio hamadryas ADIK     325 GCCGGGAGCGGTCTATTTGAGCAGCCTGGGACCCAGGCACCGCGCCATCCCTGAGAAAGCAGCGG 389
Callithrix jacchus ADIK  311 GCCAAGAGCGGTTTGTCTGGGCAGCCTGGGACCCAGGCACCGC--CATCCCTGAGAAAGCGGCAG 373

                                   400       410       420       430        440       450
Homo sapiens ADIK        384 TCTGGAGAGCAGGCATCTCAGATCCCTAAGAAACCAGCCGTCCGAGAAGCCGCGGATCTCAGGTG 448
Pan troglodytes ADIK     384 TCTGGAGAGCAGGCATTTCAGATCCTTAAGAAACCAGCCGTCCGAGAAGCCGCGGATCTCAGGTG 448
Gorilla gorilla ADIK     384 TCTGGAGAGCAGGCATCTCAGATCCCTAAGAAACCAGCCGTCCGAGAAGCCGCGGATCTCAGGTG 448
Papio hamadryas ADIK     390 TCTGGAGAGCAGGCATCTCAGATCTGTAAGAAACCAGCCGTCTGAGAAGCCGCGGATCTCAGGTG 454
Callithrix jacchus ADIK  374 TCTGGAGAGCAGACATCTCAGATCTGTAAGAAACCAGTCGTCCGAGAAGCCGCGGATCTCAGGTG 438

                              460       470       480       490        500       510       520
Homo sapiens ADIK        449 CCCAGGATCGTTAGGACTGAACGGGAGGGTACTAGAGGACCACTGGCTCTGGACCGTCGGGAGCT 513
Pan troglodytes ADIK     449 CCCAGGATCGTTAGGACTGAACGGGAGGGTACTAGAGGACCACTGGCTCTGGACCGTCGGGAGCT 513
Gorilla gorilla ADIK     449 CCCAGGATCGTTAGGACTGAACGGGAGGGTACTAGAGGACCACTGGCTCTGGACCGTCGGGAGCT 513
Papio hamadryas ADIK     455 CCCAGGATCGTTAGGACTGAACGGGAGGGTACTAGAGGACCACGGGCTCTGGATCGTCAGGAGCT 519
Callithrix jacchus ADIK  439 CCCAGGATC-TTAGGACTGAATGGGAGGGTACTAGAGGACCAGGGATTCTGGACCGTCGGGAGCT 502

                                   530       540       550       560        570       580
Homo sapiens ADIK        514 G-CCCCTGACGTAACCCACGAGG-GGCCTCCCCTTGACGGACGGCTT-----GGGGAGCGGCACC 571
Pan troglodytes ADIK     514 G-CCCCTGACGTAACCCACGAGG-GGCCTCCCCTTGACGGACGGCTT-----GGGGAGCGGCACC 571
Gorilla gorilla ADIK     514 G-CCCCTGACGTAACCCACGAGG-GGCCTCCCCTTGACGGACGGCTT-----GGGGAGCGGCACC 571
Papio hamadryas ADIK     520 G-CCCCTGACGTAACCCACGAGG-GGCCTCCCCTTGACGGACGGCTT-----GGGGAGCGGCACC 577
Callithrix jacchus ADIK  503 ATCCCCTGACGTAGCCCACGGGGGGGCCTCCCCTCGACTGACGGCTCGGCTCGGGAAGCGGCATC 567
```

```
                                590       600       610       620       630       640       650
Homo sapiens ADIK         572 GCCGCGGCTGGAGCCCGCAGAGGCAGGGTAAGGGGAGCGGGGGGCAGCCGTCGGGGGAGTGCAGA 636
Pan troglodytes ADIK      572 GCCGCGGCTGGAGCCCGCAGAGGCAGGGTAAGGGGAGCGGGGGGCAGCCGTCGGGGGAGTGCAGA 636
Gorilla gorilla ADIK      572 GCCGCGGCT-GAGCCCGCAGAGGCAGGGTAAGGGGAGCGGGGGGCAGCCGTCGGGGGAGTGCAGA 635
Papio hamadryas ADIK      578 GCCGCCGCGGGGACCCGTAGAGGCAGGGTAAGGGGAGTGGGGGGCAGCCGTCGGGGGAGTGCAGA 642
Callithrix jacchus ADIK   568 GCAGCGGCGAGG--CCGCAGAGGTAGAGTAGGGGGAGCGGG-GGCAGCCGTCGGGGGAGTGCAGA 629

                                     660       670       680       690       700       710
Homo sapiens ADIK         637 CCCAGGCCCAAGGCGGGTCACCGCCTCCTG-GCCCGCGGAGAGCCCCG-GCCCCGGCAGCCATTG 699
Pan troglodytes ADIK      637 CCCAGGCCCAAGGCGGGTCACCGCCTCCTG-GCCCGCGGAGAGCCCCG-GCCCCGGCAGCCATTG 699
Gorilla gorilla ADIK      636 CCCAGGCCCAAGGCGGGTCACCGCCTCCTG-GCCCGCGGAGAGCCCCG-GCCCCGGCAGCCATTG 698
Papio hamadryas ADIK      643 CCCAGGCCCAAGGCGGGTCACCGCCTCCTGGGCCCGCGGAGAGTCCCGGGCCCCGGCAGCCATTG 707
Callithrix jacchus ADIK   630 CCCCTGCCCAAGGCGGGTCACCGCCTCTTGGGCCCGCGGAGAGCCCCGGGCCCCGGCAGCCATTG 694

                                720       730       740       750       760       770       780
Homo sapiens ADIK         700 CGCCCAAGAGTGAGGAAGATTTGCTGGCCCTGGCAGCGTCGCGGCTGAGCCGCCGCAAGAGGGTG 764
Pan troglodytes ADIK      700 CGCCCAAGAGTGAGGAAGATTTGCTGGCCCTGGCCGCGTCGCGGCTGAGCCGCCGCAAGAGGGTG 764
Gorilla gorilla ADIK      699 CGCCCAAGAGTGAGGAAGATTTGCTGGCCCTGGCCGCGTCGCGGCTGAGCCGCCGCAAGAGGGTG 763
Papio hamadryas ADIK      708 CGCCCAAGAGTGAAGAAGATTTGCTGGCCCTGGCCGCGTCGCGGCTGAGCCGCCGCAAGAGGGTG 772
Callithrix jacchus ADIK   695 CGCCCAAGAGTGAGGAAGATTTGCTGGCCCTGGCCGCGTCGCGGCTAAGCCGCCGCAAGAGGGTG 759

                                     790       800
Homo sapiens ADIK         765 GCGGGCGCGGCCGTCGGAGTGGCC 788 (-1 bp)
Pan troglodytes ADIK      765 GCGGGCGCGGCCGTCGGAGTGGCC 788 (-1 bp)
Gorilla gorilla ADIK      764 GCGGGCGCGGCCGTCGGAGTGGCC 787 (-1 bp)
Papio hamadryas ADIK      773 GCGGGTGCGGCCGTCGGGGTGGCC 796 (-1 bp)
Callithrix jacchus ADIK   760 GCGGGCGCGGCCGTCGGGGTGGCC 783 (-1 bp)
```

J

```
                                           10        20        30        40        50        60
Homo sapiens ADIM              1 CCGTTTCTATTTTAATCAAGAATATGTCAAAAGAAAGAGGAGCCAGAGACAGCCTTCTCTTAACA  65
Pan troglodytes ADIM           1 CCGTTTCTATTTTAATCAAGAATATGTCAAAAGAAAGAGGAGCCAGAGACAGCCTTCTCTTAACA  65
Pongo abelii ADIM              1 CCATTTCTATTTTAATCAAGCATATGTCAAAAGAAAGAGGAGCTGGAGACAGCCTTCTCTTAACA  65
Nomascus leucogenys ADIM       1 CCGTTTCTATTTTAATCAAGAATATGTCAAAAGAAAGAGGAGCCAGAGACAGCCTTCTCTTAACA  65
Papio hamadryas ADIM           1 CCGTTTCTATTTTAAATCAGAATATGTCAAAAGAAAGAGGAGCCAGAGACAGCCTTCTCTTAACA  65
Callithrix jacchus ADIM        1 CCATTTCTATTTTAATCAAGAATATGTCAAATGAAAGAGTAGCCAGAGATAGATAGC-CTTCTTT  64

                                      70        80        90       100       110       120       130
Homo sapiens ADIM             66 CAAAGCAGGGTGTTGTATCTTCATCTGACAATAAGTGACTTAGTTAACTTTGATTTAATTTTGGC 130
Pan troglodytes ADIM          66 CAAAGCAGGGTGTTGTATCTTCATCTGACAATAAGTGACTTGGTTAACTTTGATTTAATTTTGGC 130
Pongo abelii ADIM             66 CAAAGCACGGTGTTTTATCTTCATCTGACAATAAGTGACTTGGTTAACTTTGATTTAATTTTGGC 130
Nomascus leucogenys ADIM      66 CAAGGCAGGGTGTTGTATCTTCATCTGACAATAAGTGACTTGGTTAACTTTGATTTAATTTTGGC 130
Papio hamadryas ADIM          66 CAAAGCAGAGTGTTATATCTTCATCTGACAATAAGTGACTTGGTGAACTTTGATTTAATTTTGGC 130
Callithrix jacchus ADIM       65 TACAGCAGGGTGTTGTATCTTCATCTAACAGTAGCTGACTTGGTTAACTTTGATTTAATATTGGC 129

                                           140       150       160       170       180       190
Homo sapiens ADIM            131 TTTTGGGAAGTCATGGGGAAAAGAAGTAAAGCTGAGGGTGGCGAAATACCTTTGCTAAACCCAGA 195
Pan troglodytes ADIM         131 TTTTGGGAAGTCATGGGGAAAAGAAGTAAAGCTGAGGATGGCGAAATACCTTTGCTAAACCCAGA 195
Pongo abelii ADIM            131 TTTTGGGAAGTCATGGGGAAAAGAAGTAAAGCTGAGGATGGCGAAATACCTTTGCTAAACCCAGA 195
Nomascus leucogenys ADIM     131 TTTTGGAAAGTCATGGGGAAAAGAAGTAAAGCTGAGGATGGCGAAATACCTTTGCTAAACCCAGA 195
Papio hamadryas ADIM         131 TTTTGGGAAGTCATGGGGAAAAGAAGTAAAGCTGAGGATAGCGAAATACCTTTGCTAAACCCAGA 195
Callithrix jacchus ADIM      130 TTTTGGGAAGCCAGGGGAAAAAGAAGTAAAGCTGAAGATGGTGAAATATTGTTGCTATACCCAGA 194

                                      200       210       220       230       240       250       260
Homo sapiens ADIM            196 GACACTGCACCTGAATCCTGCTCAGTGTTTTGTAGGCAACCAACTGCCTTCTGCCACTGGTATCC 260
Pan troglodytes ADIM         196 GACACTGCACCTGAATCCTGCTCAGTGTTTTGTAGGCAACCAACTGCCTTCTGCCACTGGTATCC 260
Pongo abelii ADIM            196 GACACTGCACCTGAATCCTGCTCAGTGTTTTGTAGGCAACCAACTGCCTTCTGCCACTGGTATCT 260
Nomascus leucogenys ADIM     196 GACACTGCACCTGAATCCTGCTCAGTGTTTTGTAGGCAACCAACTGCCTTCTGCCACTGGTATCC 260
Papio hamadryas ADIM         196 GGCACTGCACCTGAATCTTGCTCAGTGTTTTGTAGGCAACTAACTGCCTTCTGCCACTGCCTTCT 260
Callithrix jacchus ADIM      195 AACACTGCACCTGATTCCCGCTCAGTGTTTCCTAGGAGACCAACCGCCTTCTGCCGCCGGTATCC 259

                                           270       280       290       300       310       320
Homo sapiens ADIM            261 GGAAGGGGAAATGAGTTACCTTAAGTGGGCCAAAGTGGGGAAATACTAACAGTACAGCTAGAAAC 325
Pan troglodytes ADIM         261 GGAAGGGGAAATGAGTTACCTTAAGTGGGCCAAAGTGGGGAAATACTAACAGTACAGCTAGAAAC 325
Pongo abelii ADIM            261 GGAAGGGGAAATGAGTTACCTTAAGTGGGCCAAAGTGGGGAAATACTAACAGTACAACTAGAAAG 325
Nomascus leucogenys ADIM     261 GGAAGGGGAAATGAGTTACCTTAAGTGGGCCAAAGTGGGGAAATACTAACAGTACAACTAGAAAG 325
Papio hamadryas ADIM         261 GGAAGGGGAAATGAGTTATCTTAAGTGGGCCAAAGTGGGGAAATACTAACAGTACAACTAGAAAG 325
Callithrix jacchus ADIM      260 GGAAGGGGAAATGAGTTACCTTAAGTGGGCCCAAGTGGGGAAGTATTAACTGTACAGCTAGATAG 324

                                      330       340       350       360       370       380       390
Homo sapiens ADIM            326 CAAGAGCTGTCGTGAAAGCCCTGAATGGGTTCCCACTGCTATTTCAGACAAAACTGATGCAGTTA 390
Pan troglodytes ADIM         326 CAAGAGCTGTCGTGAAAGCCCTGAATGGGTTCCCACTGCTATTTCAGACAAAACTGATGCAGTTA 390
Pongo abelii ADIM            326 CAAGAGCTGTCGTGAAAGCCCTGAATGGGTTCCCACTGCCATTTCAGACAAAACTGATGCAGTTA 390
Nomascus leucogenys ADIM     326 CAAGAGCTGTTGTGAAAGCCCTGAATGGGTTCCCACTGCCATTTCAGACAAAACTGATGCAGTTA 390
Papio hamadryas ADIM         326 CAAGAGCTGTCGTGAAAGCCCTGAATGGGTTCCCACTGCCATTTCAGACGAAACTGATGCAGTTA 390
Callithrix jacchus ADIM      325 CAAGAGCTGTCGTGAAAGCCCTGAATGGGTTTCCACTGCTATTTCAGACGAAACTCATGCAGTTA 389

                                           400       410       420       430       440       450
Homo sapiens ADIM            391 AGCCTGAATTTCCTGGCAAGCACAAGGGTAGATTTTTCCAAAAGGCTTTTTAGACTGCAAATACA 455
Pan troglodytes ADIM         391 AGCCTGAATTTCCTGGCAAGCGCAAGGGTAGATTTTTCCAAAAGGCTTTTTAGACTGCAAATACA 455
Pongo abelii ADIM            391 AGCCTGAATTTCCTGGCAAGCACAAGGGTAGATTTTTCCAAAAGGCTTTTTAGACTGCAAATACA 455
Nomascus leucogenys ADIM     391 AGCCTGACTTTCCTGGCAAGCGCAAGGGTAGATTTTTCCAAAAGGCTTTTTAGACTGCAAATACA 455
Papio hamadryas ADIM         391 AGCCTGAATTTCCTGGCAAGCGCAACGGTAGGTTTTTCCAAAAGGCTTTTTAGACTGCAAATACA 455
Callithrix jacchus ADIM      390 AGCCTGAATTTCCTGGCAAGCGCAAGAGTAGATTTTTCCAAAAGGCTTTTCAGACTGCAAATGCA 454

                                      460       470       480       490
Homo sapiens ADIM            456 CAGCCTTTCCATGTCTAATCACAAAAGCAAACTGCTGGACAC 497 (-1 bp)
Pan troglodytes ADIM         456 CAGCCTTTCCATGTCTAATCACAAAAGCAAACTGCTGGACAC 497 (-1 bp)
Pongo abelii ADIM            456 CAGCCTTTCCATGTCTCATCACAAAAGCAAACTGCTGGACAC 497 (-132 bp)
Nomascus leucogenys ADIM     456 CAGCCTTTCCATGTCTAATCACAAACGCAAACTGCTGGACAC 497 (-1 bp)
Papio hamadryas ADIM         456 CAGCCTTTCCATGTCTAATCACAAAAGCAAACCGCTGGACAC 497 (-1 bp)
Callithrix jacchus ADIM      455 CAGCCTTTTCATGTCTAATCACGAAAGCCAGCCGCTGGACAC 496 (-28 bp)
```

K

```
                                  10        20        30        40        50        60
Homo sapiens ADIO         1 AGAGTGTAGCAGCCCCTGAGCCTCGGCCTTGGCCTCACCCACTGGGCCAGGACCGTCCGTGCCCA   65
Pan troglodytes ADIO      1 AGAGTGTAGCAGCCCCTGAGCCTCGGCCTTGGCCTCACCCACTGGGCCAGGAGCGTCCGTGCCCA   65
Macaca mulatta ADIO       1 AGAGTGCGGCAGCCCCTGAGCCCCGGCCTTGGCCTCACCCACTGGGCCAGGACCGTCCACGTCCA   65

                               70        80        90       100       110       120       130
Homo sapiens ADIO        66 GGCAGAAGCTCCCGCCTCCACTGGCTGTATTCCCAACGGGGGCAGGG-----CCGGGCTGCCGTA 125
Pan troglodytes ADIO     66 GGCAGAAGCTACCGCCTCCACTGGCTGTATTCCCAACGGGGGCAGGG-----CCGGGCTGCTGTA 125
Macaca mulatta ADIO     66 GGCAGAAGCTCCCGCCTCCACCAGCTGTATTCCCAATAGGGGCAGGGTGGGGCTGGGCCGCCGTA 130

                              140       150       160       170       180       190
Homo sapiens ADIO       126 GGGTCTGGGGGCTCCAGCTGGGGCACCTCCTTCTCAGTCCCTCCCCTCTTTCCTCTCCCCCAGCC 190
Pan troglodytes ADIO    126 GGGTCTGGGGGCTCCAGCTGGGGCACCTCCTTCTCAGTCCCTCCCCTCTTTCCTCTCTCCCAGCC 190
Macaca mulatta ADIO     131 GGGTCTGGGGGCTCCAGCTGGGGCGCCTCCTTCTAAGTCCCTCCCCTCTTCCCTCTCCCCCAGCC 195

                             200
Homo sapiens ADIO       191 CTGGCCAAG 199 (-1 bp)
Pan troglodytes ADIO    191 CTGGCCAAG 199 (-1 bp)
Macaca mulatta ADIO     196 CTGGCCGAG 204 (-1 bp)
```

L

```
                                        10        20        30        40        50        60
Homo sapiens ADIQ           1 TAAGCACTTTAGCTCAGGGTGGACTCAGGGCTGCCTCCCCCTTCCTGAGCAACCTAGGGGCTCCT  65
Pan troglodytes ADIQ        1 TAAGCGCTTTAGCTCAGGGTGGACTCAGGGCTGCCTCCCCCTTCCTGAGCAACCTAGGGGCTCCT  65
Pongo abelii ADIQ           1 TAAGCACTTTAGCTCAGGGTGGACTCAGGGCTGCCTCCCCCTTCCTGAGCAACCTAGGGGCTCCT  65
Nomascus leucogenys ADIQ    1 TAAGCACTTTAGCTCAGGGTGGACTCAGGGCTGCCTCCCCCTTCCTGAGCAACCTAGGGGCTCCT  65
Macaca mulatta ADIQ         1 TGAGCACTTTAGCTCAGGGTGGACTCAGGGCTGCCTTCCCCTTCCTGAGCAACCTAGGGGCTCCT  65

                                   70        80        90       100       110       120       130
Homo sapiens ADIQ          66 GTGCCCCATGCCTGTGTCCATTTCCCAATCATCAGGGGGCCTCAGACTCCTGCCTGCTCTGACTT 130
Pan troglodytes ADIQ       66 GTGCCCCATTCCTGTGTCCATTTCCCAATCATCAGGGGGCCTCAGACTCCTGCCTGCTCTGACTT 130
Pongo abelii ADIQ          66 GTGCCCCATGCCTGTGTCCAGTTCCCAATCATCAGGGGGCCTCAGACTCCTGCCTGCTCTGACTT 130
Nomascus leucogenys ADIQ   66 GTGCCCCATCCCTGTGTCCATTTCCCAATCATGAGGGGGCCTCAGACTCCTGCCTGCTCTGACTT 130
Macaca mulatta ADIQ        66 CTGCCCCATCTGTGTGTCCATTTCCCAATCATCAGGGAGCCTCACATTCCTCCCTGCTCTGACTT 130

                                        140       150       160       170       180       190
Homo sapiens ADIQ         131 TCTAGGCCTTTGAATCAAGCAAGACCCCACTGGATCACCAAAGAGTTAAGCAAAGGAAATCCCTG 195
Pan troglodytes ADIQ      131 TCTAGGCCTTTCAATCAAACAAGACCCCACTGGATCACCAAAGAGTTAAGCAAAGGAAATCCCTG 195
Pongo abelii ADIQ         131 TCTAGGCCTTTGAATCAAACAAGACCCCACTGGATCACCAAAGAGTTAAGCAAAGGAAATCCCTG 195
Nomascus leucogenys ADIQ  131 TCTAGGCCTTTGAATCAGACAAGACCCCATTGGATCACCAAAGAGTTAAGCAAAGGAAATCCCTG 195
Macaca mulatta ADIQ       131 TCTAGGCCTTTGAATCAAACAAGACCCCACTGGATCACCAAAGAGTTAAGCAAAGGAAAGCTCTG 195

                                   200       210       220       230       240       250       260
Homo sapiens ADIQ         196 CCTCCCTTTATCTCCGCAGAACCCAGAGGCTGAAAGGGAGGGAGCAGGGAGGAGGGGAAGTGGGA 260
Pan troglodytes ADIQ      196 CCTCCCTTTATCTCCGCAGAACCCAGAGGCTGAAAGGGAGGGAGCAGGGAGGAGGGGAAGTGGGA 260
Pongo abelii ADIQ         196 CCTCCCTTTATCTCCGCAGAACCCAGAGGCTGAAAGGGAGGGAGCAGGGAGGAGGGGAAGTGGGA 260
Nomascus leucogenys ADIQ  196 CCTCCCTTTATCTCCGCAGAACCCAGAGGCTGAAAGGGAGGGAGCAGGGAGGAGGGGAAGTGGGA 260
Macaca mulatta ADIQ       196 CCTCCCTTTATCTCCGCAGAACCCAGAGGCTGAAGGGGAGGGAGCAGGGAGGAGGGGAAGTGGGA 260

                                        270       280       290       300       310       320
Homo sapiens ADIQ         261 AGCGGCTCTGCCCTCTCCCTCCGCCCTCCCTCCGCCCTCTCTCCCTCCATCCCCTCCTCTCTCCC 325
Pan troglodytes ADIQ      261 AGCGGCTCTGCCCTCTCCCTCCGCCCTCCCTCCGCCCTCTCTCCCTCCATCCCCTCCTCTCTCCC 325
Pongo abelii ADIQ         261 AGCGGCTCTGCCCTCTCCCTCCGCCCTCCCTCCGCCCTCTCTCCCTCCATCCCCTCCTCTCTCCC 325
Nomascus leucogenys ADIQ  261 AGCGGCTCTGCCCTCTCCGTCCGCCCTCCCTCCGCCCTCTCTCCCTCCATCCCCTCCTCTTTCCC 325
Macaca mulatta ADIQ       261 AGCGGCTCTGCCCGCTCCCTCCGCCCTCCCTCCGCCCTCTCTCCCTCCATCCTCTCCTCTCTCCC 325

                                   330       340       350       360       370       380       390
Homo sapiens ADIQ         326 TCCTCTGCTGGGCCTGGGGGTCTGGGCCAGCAACAAGTTAGTATTGCAGACATGGGCCAAGGAGC 390
Pan troglodytes ADIQ      326 TCCTCTGCTGGGCCTGGGGGTCTGGGCCAGCAACAAGTTAGTATTGCAGACATGGGCCAAGGAGC 390
Pongo abelii ADIQ         326 TCCTCTGCTGGGCCTGGGGGTCTGGGCCAGCAACAAGTTAGTGTTGCAGACATGGGCCAAGGAGC 390
Nomascus leucogenys ADIQ  326 TCCTCTGCTGGGCCTGGGGGTCTGGGCCAGCAACAAGTTAGTATTGCAGACATGGGCCAAGGAGC 390
Macaca mulatta ADIQ       326 TCCTCTGCTGGGCCTGGGGGTCTGGGCCAGCAAC----TAGTATTGCAGACATGGGCCAAGGAGC 386

Homo sapiens ADIQ         391 CAGAGGCC 398 (-1 bp)
Pan troglodytes ADIQ      391 CAGAGGCC 398 (-1 bp)
Pongo abelii ADIQ         391 CAGAGGCC 398 (-1 bp)
Nomascus leucogenys ADIQ  391 CAGAGGCC 398 (-1 bp)
Macaca mulatta ADIQ       387 CAGAGGCC 394 (-1 bp)
```

M

```
                                              10        20        30        40        50        60
Homo sapiens ADIR              1 AGAGTA-TGTAAAAACCTATTACCAGAAAAATCTAGAAAAGTATAATCTAGCTATA---TATTGC  61
Pan troglodytes ADIR           1 AGAGTA-TGTAAAAACCTATTACCAGAAAAATCTAGAAAAGTATAATCTAGCTATA---TATTGC  61
Pongo abelii ADIR              1 AGAGTATTGTAAAAACCTAGTGCCAGAAAAATCTAGAAAAGTATAATCTAGCTATA---TATTGC  62
Nomascus leucogenys ADIR       1 AGGGTATTGTAAAAACCTATTGTCAGAAAAATCTAGAAAAGTATAATCTAGCTATA---TATTGC  62
Macaca mulatta ADIR            1 AGAGTATTGTAAAAACCTATTGCCAGAAAAATCTAGAAAAGTATAATCTAGCTATA---TATTGC  62
Papio hamadryas ADIR           1 AGAGTATTGTAAAAACCTATTGCCAGAAAAATCTAGAAAAGTATAATCTAGCTATA---TATTGC  62
Callithrix jacchus ADIR        1 AGAGTATTATAAAATCCTATTGCCAGAAAAATCTAGAAAAGTATAATCTAGCTATA---TATTGC  62
Tarsius syrichta ADIR          1 ATAGTATTATAAAAACCTATTACTAAAAAAATCTAGAAAAGTATAATCTAGCTATACTTTATTGC  65
Otolemur garnettii ADIR        1 ATAGTATTATAAAAGCCGATGGCCAGAAAAATCTAGAAAAGTATAATCCAGCTTTACGGTATTGC  65

                                       70        80        90       100       110       120       130
Homo sapiens ADIR             62 TTTTGTTTTGGAATTTACTATAAAATGTATCTTTGTCTG--TTTCAGTAGCCGACTAATGTTAAA 124
Pan troglodytes ADIR          62 TTTTGTTTTGGAATTTACTATAAAATGTATCTTTGTCTG--TTTCAGTAGCCGACTAATGTTAAA 124
Pongo abelii ADIR             63 TTTTGTTTTGGAATTTACTATAAAATGTATCTTTGTCTG--TTTCAGTAGCCGACTAATGTTAAA 125
Nomascus leucogenys ADIR      63 TTTTGTTTTGGAATTTACTATAAAATGTATCTTTGTCTA--TTTCAGTAGCCAACTAATGTTAAA 125
Macaca mulatta ADIR           63 TTTTGTTTTGGAATTTACTGTAAAATGTATCTTTGTCTG--TTTCAGTAGCCGACTAATGTTAAA 125
Papio hamadryas ADIR          63 TTTTGTTTTGGAATTTACTGTAAAATGTATCTTTGTCTG--TTTCAGTAGCCGACTAATGTTAAA 125
Callithrix jacchus ADIR       63 TTTTGTTTTGGAATTTACTATAAAATGTATCTTTGTCTG--TTTCAGTAGCCGACTAATGTTAAA 125
Tarsius syrichta ADIR         66 TTT-GTTATGGAATTTACTATAAAATGTATCTCTATCTG--TTTCAGTAGCCAACTAATGTTAAA 127
Otolemur garnettii ADIR       66 TTTTGTTTTGGAATTTACTATAAAATGTGTCTTTATCTGTGTTTCAGTAGCCGACTAATGTTAAA 130

                                              140       150       160       170       180       190
Homo sapiens ADIR            125 TATTGTGTTCAAAGCTAACTGCTTCATTATGAGAAACTCACTGTTGCTAATAAAACAGCAGGGTG 189
Pan troglodytes ADIR         125 TATTGTGTTCAAAGCTAACTGCTTCATTATGAGAAACTCACTGTTGCTAATAAAACAGCAGGGTG 189
Pongo abelii ADIR            126 TATTGTGTTCAAAGCTAACTGCTTCATTATGAGAAACTCACTGTTGCTAATAAAACAGCAGGGTG 190
Nomascus leucogenys ADIR     126 TATTGTGTTCAAAGCTAACTGCTTCATTATGAGAAACTCACTGTTGCTAATAAAACAGCAGGGTG 190
Macaca mulatta ADIR          126 TATTGTGTTCAAAGCTAACTGCTTCATTATGAGAAACTCACTGTTGCTAATAAAACAGCAGGGTG 190
Papio hamadryas ADIR         126 TATTGTGTTCAAAGCTAACTGCTTCATTATGAGAAACTCACTGTTGCTAATAAAACAGCAGGGTG 190
Callithrix jacchus ADIR      126 TATTGTGTTCAAAGCTAACTGCTTCATTATGAGAAACTCACTGTTGCTAATAAAACAGCAGGGTG 190
Tarsius syrichta ADIR        128 TATTGTGTTCAAAGCTAACTGCTTCATTATGAGAAACTCACTGTTGCTAATAAAACAGCAGGGTG 192
Otolemur garnettii ADIR      131 TATTGTGTTCAAAGCTAACTACTTCATTATGAGAAACTCACTGTTGCTAATAAAACAGCAGGGTG 195

                                   200       210       220       230       240       250       260
Homo sapiens ADIR            190 GAGTGATTCTTCTCAGGTGAGCAGCCCAGAAAGAGACAACGAAACCTTTAACAGTGGTGACTCTG 254
Pan troglodytes ADIR         190 GAGTGATTCTTCTCAGGTGAGCAGCCCAGAAAGAGACAACGAAACCTTTAACAGTGGTGACTCTG 254
Pongo abelii ADIR            191 GAGTGATTCTTCTCAGGTGAGCAGCCCAGAAAGAGACAACGAAACCTTTAACAGTGGTGACTCTG 255
Nomascus leucogenys ADIR     191 GAGTGATTCTTCTCAGGTGAGCAGCCCAGAAAGAGACAACGAAACCTTTAACAGTGGTGACTCTG 255
Macaca mulatta ADIR          191 GAGTGATTCTTCTCAGGTGAGCAGCCCAGAAAGAGACAACGAAACCTTTAACAGTGGTGACTCTG 255
Papio hamadryas ADIR         191 GAGTGATTCTTCTCAGGTGAGCAGCCCAGAAAGAGACAACGAAACCTTTAACAGTGGTGACTCTG 255
Callithrix jacchus ADIR      191 GAGTGATTCTTCTCAGGTGAGCAGCCCAGAAAGAGACAACGAAACCTTTAACAGTGGTGACTCTG 255
Tarsius syrichta ADIR        193 GAGTGATTCTTCTCAGGTGAGCAGCCCAGAAAGAGACAACGAAACCTTTAACAGTGGTGACTCTG 257
Otolemur garnettii ADIR      196 GAGTGATTCTTCTCAGGTGAGCAGCCCAGAAAGAGACAACGAAACCTTTAACAGTGGTGACTCTG 260

                                        270       280
Homo sapiens ADIR            255 GACAAGGAGACTCCCGTAGC 274 (-1 bp)
Pan troglodytes ADIR         255 GACAAGGAGACTCCCGTAGC 274 (-1 bp)
Pongo abelii ADIR            256 GACAAGGAGACTCCCGTAGC 275 (-1 bp)
Nomascus leucogenys ADIR     256 GACAAGGAGACTCCCGTAGC 275 (-1 bp)
Macaca mulatta ADIR          256 GACAAGGCGACTCCCGTAGC 275 (-1 bp)
Papio hamadryas ADIR         256 GACAAGGCGACTCCCGTAGC 275 (-1 bp)
Callithrix jacchus ADIR      256 GACAAGGAGACTCCCGTAGC 275 (-1 bp)
Tarsius syrichta ADIR        258 GACAAGGAGACTCCCGCAGC 277 (-1 bp)
Otolemur garnettii ADIR      261 GACAAGGAGACTCCCGTAGC 280 (-1 bp)
```
