# Supplementary material for: Comparative genomic analysis of eutherian adiponectin genes
Source: Heliyon. 2018 Jun 6;4(6):e00647. doi: 10.1016/j.heliyon.2018.e00647 (PMC6040601; doi:10.1016/j.heliyon.2018.e00647)
Supplement: Supplementary data file 4 — Pairwise nucleotide sequence identity patterns of eutherian adiponectin genes. [file mmc4.pdf]

Supplementary data file 4: Pairwise nucleotide sequence identity patterns of eutherian adiponectin genes.

	$\bar{a}$	a_{max}	a_{min}	$\bar{a}_{ad}$	$\bar{a}$	a_{max}	a_{min}	$\bar{a}_{ad}$	$\bar{a}$	a_{max}	a_{min}	$\bar{a}_{ad}$	$\bar{a}$	a_{max}	a_{min}	$\bar{a}_{ad}$	$\bar{a}$	a_{max}	a_{min}	$\bar{a}_{ad}$
Cluster	*ADIA*				*ADIB*				*ADIC*				*ADID*				*ADIE*			
ADIA	**0,786**	**0,956**	**0,645**	**0,042**																
ADIB	0,489	0,536	0,396	0,03	**0,775**	**0,993**	**0,633**	**0,077**												
ADIC	0,465	0,517	0,399	0,021	0,431	0,479	0,367	0,025	**0,759**	**0,991**	**0,635**	**0,055**								
ADID	0,345	0,38	0,295	0,019	0,349	0,389	0,296	0,019	0,32	0,355	0,274	0,015	**0,787**	**0,995**	**0,641**	**0,052**				
ADIE	0,338	0,356	0,299	0,012	0,325	0,353	0,277	0,015	0,307	0,337	0,27	0,011	0,369	0,404	0,309	0,019	**0,91**	**0,995**	**0,848**	**0,045**
ADIF	0,301	0,323	0,275	0,008	0,292	0,324	0,251	0,013	0,294	0,319	0,257	0,01	0,329	0,355	0,282	0,014	0,294	0,315	0,272	0,008
ADIG	0,322	0,361	0,288	0,02	0,306	0,353	0,271	0,018	0,316	0,358	0,279	0,017	0,356	0,406	0,317	0,017	0,344	0,387	0,313	0,026
ADIH	0,341	0,37	0,28	0,014	0,347	0,374	0,295	0,017	0,35	0,379	0,295	0,014	0,376	0,415	0,316	0,018	0,379	0,4	0,312	0,014
ADII	0,229	0,248	0,203	0,008	0,215	0,235	0,19	0,009	0,196	0,208	0,18	0,006	0,222	0,242	0,194	0,008	0,246	0,26	0,226	0,007
ADIJ	0,252	0,27	0,224	0,009	0,229	0,249	0,201	0,01	0,224	0,239	0,204	0,007	0,256	0,279	0,222	0,012	0,272	0,283	0,263	0,004
ADIK	0,243	0,268	0,197	0,012	0,231	0,25	0,194	0,011	0,217	0,239	0,195	0,008	0,247	0,268	0,21	0,011	0,278	0,292	0,262	0,006
ADIL	0,26	0,278	0,227	0,009	0,224	0,24	0,201	0,009	0,225	0,234	0,213	0,006	0,258	0,277	0,223	0,011	0,283	0,291	0,271	0,005
ADIM	0,257	0,322	0,211	0,026	0,256	0,317	0,221	0,023	0,248	0,307	0,212	0,023	0,267	0,332	0,228	0,024	0,227	0,281	0,199	0,023
ADIN	0,151	0,168	0,131	0,007	0,142	0,153	0,126	0,006	0,143	0,154	0,13	0,005	0,139	0,153	0,124	0,005	0,175	0,186	0,157	0,006
ADIO	0,265	0,29	0,226	0,011	0,25	0,27	0,213	0,012	0,261	0,285	0,234	0,01	0,235	0,261	0,197	0,011	0,252	0,268	0,226	0,008
ADIP	0,245	0,28	0,196	0,018	0,231	0,26	0,188	0,014	0,24	0,266	0,196	0,014	0,233	0,259	0,191	0,013	0,226	0,25	0,183	0,016
ADIQ	0,248	0,275	0,232	0,008	0,245	0,276	0,225	0,008	0,253	0,266	0,239	0,007	0,235	0,262	0,206	0,01	0,228	0,237	0,205	0,006
ADIR	0,194	0,239	0,162	0,01	0,171	0,209	0,142	0,01	0,184	0,228	0,158	0,009	0,203	0,229	0,171	0,009	0,218	0,243	0,208	0,004

Cluster	*ADIF*				*ADIG*				*ADIH*				*ADII*				*ADIJ*			
ADIF	**0,84**	**0,99**	**0,726**	**0,046**																
ADIG	0,309	0,347	0,277	0,016	**0,82**	**0,994**	**0,739**	**0,049**												
ADIH	0,323	0,355	0,281	0,01	0,528	0,597	0,483	0,035	**0,879**	**0,993**	**0,723**	**0,052**								
ADII	0,178	0,197	0,163	0,005	0,199	0,222	0,182	0,012	0,231	0,247	0,199	0,006	**0,898**	**0,98**	**0,856**	**0,028**				
ADIJ	0,198	0,21	0,179	0,004	0,228	0,259	0,209	0,015	0,259	0,273	0,222	0,007	0,623	0,647	0,609	0,007	**0,943**	**0,997**	**0,917**	**0,015**
ADIK	0,19	0,206	0,171	0,005	0,206	0,233	0,186	0,012	0,242	0,255	0,208	0,007	0,5	0,519	0,475	0,007	0,579	0,592	0,564	0,006
ADIL	0,196	0,206	0,178	0,004	0,216	0,238	0,198	0,013	0,261	0,272	0,229	0,006	0,594	0,602	0,588	0,004	0,69	0,698	0,68	0,004
ADIM	0,202	0,249	0,176	0,017	0,242	0,294	0,206	0,016	0,25	0,311	0,216	0,024	0,183	0,221	0,16	0,018	0,202	0,253	0,175	0,021
ADIN	0,116	0,131	0,107	0,005	0,135	0,155	0,121	0,007	0,152	0,165	0,127	0,004	0,154	0,162	0,148	0,003	0,161	0,168	0,15	0,003
ADIO	0,188	0,211	0,17	0,008	0,211	0,241	0,191	0,015	0,257	0,272	0,22	0,007	0,241	0,256	0,223	0,007	0,229	0,241	0,213	0,006
ADIP	0,174	0,202	0,139	0,01	0,221	0,249	0,18	0,013	0,243	0,274	0,193	0,015	0,195	0,22	0,163	0,013	0,191	0,212	0,158	0,013
ADIQ	0,186	0,205	0,174	0,006	0,211	0,235	0,196	0,007	0,234	0,259	0,216	0,007	0,234	0,243	0,224	0,004	0,226	0,238	0,214	0,004
ADIR	0,151	0,171	0,13	0,004	0,164	0,198	0,142	0,01	0,178	0,211	0,151	0,008	0,195	0,23	0,18	0,006	0,224	0,242	0,204	0,004

Cluster	*ADIK*				*ADIL*				*ADIM*				*ADIN*				*ADIO*			
ADIK	**0,915**	**0,998**	**0,86**	**0,025**																
ADIL	0,583	0,594	0,561	0,007	**0,978**	**-**	**-**	**-**												
ADIM	0,21	0,262	0,179	0,023	0,201	0,245	0,176	0,021	**0,761**	**0,999**	**0,621**	**0,093**								
ADIN	0,182	0,197	0,165	0,005	0,164	0,17	0,161	0,002	0,13	0,158	0,111	0,011	**0,908**	**0,991**	**0,828**	**0,027**				
ADIO	0,223	0,243	0,195	0,012	0,246	0,251	0,238	0,003	0,23	0,289	0,199	0,025	0,2	0,212	0,183	0,005	**0,853**	**0,981**	**0,797**	**0,056**
ADIP	0,193	0,215	0,155	0,013	0,206	0,224	0,169	0,014	0,234	0,282	0,2	0,021	0,181	0,2	0,15	0,01	0,475	0,536	0,377	0,04
ADIQ	0,217	0,225	0,203	0,004	0,239	0,244	0,234	0,002	0,244	0,296	0,215	0,018	0,184	0,194	0,177	0,003	0,537	0,559	0,517	0,009
ADIR	0,251	0,281	0,233	0,005	0,226	0,244	0,219	0,004	0,186	0,236	0,156	0,021	0,225	0,269	0,21	0,007	0,206	0,25	0,193	0,009

Cluster	*ADIP*				*ADIQ*				*ADIR*			
ADIP	**0,741**	**0,865**	**0,608**	**0,073**								
ADIQ	0,471	0,511	0,383	0,027	**0,84**	**0,99**	**0,696**	**0,131**				
ADIR	0,199	0,241	0,156	0,019	0,209	0,238	0,201	0,005	**0,909**	**1**	**0,624**	**0,059**

$\bar{a}$, average pairwise identity; $\bar{a}_{ad}$, average absolute deviation for $\bar{a}$; a_{max}, largest pairwise identity; a_{min}, smallest pairwise identity; bold, calculation within major cluster.
