# Supplementary material for: Comparative genomic analysis of eutherian adiponectin genes
Source: Heliyon. 2018 Jun 6;4(6):e00647. doi: 10.1016/j.heliyon.2018.e00647 (PMC6040601; doi:10.1016/j.heliyon.2018.e00647)
Supplement: Supplementary data file 5 — Protein sequence alignments of eutherian adiponectins. The amino acid positions were labelled according to their identity levels using white letters on black background (100% sequence identity level), white letters on dark grey background (≥75% sequence identity level) or black letters on grey background (≥50% sequence identity level). In reference human ADIA protein amino acid sequence (top), the 33 invariant amino acid sites were shown using white letters on violet backgrounds and 1 forward amino acid site was shown using white letter on red background. The stop codons were indicated by &s. [file mmc5.pdf]

Homo sapiens ADIA	-	--	-
		10 20 30 40 50 60	
Homo sapiens ADIA	-	--	-
Papio hamadryas ADIA	-	--	-
Mus musculus Adia	-	--	-
Rattus norvegicus Adia	-	--	-
Cavia porcellus ADIA	-	--	-
Ochotona princeps ADIA	-	--	-
Bos taurus ADIA	-	--	-
Equus caballus ADIA	-	--	-
Canis lupus familiaris ADIA	-	--	-
Felis catus ADIA	-	--	-
Myotis lucifugus ADIA	-	--	-
Dasypus novemcinctus ADIA	-	--	-
Loxodonta africana ADIA	-	--	-
Homo sapiens ADIB	-	--	-
Pan troglodytes ADIB	-	--	-
Pongo abelii ADIB	-	--	-
Nomascus leucogenys ADIB	-	--	-
Callithrix jacchus ADIB	-	--	-
Otolemur garnettii ADIB	-	--	-
Mus musculus Adib	-	--	-
Rattus norvegicus Adib	-	--	-
Cavia porcellus ADIB	-	--	-
Oryctolagus cuniculus ADIB	-	--	-
Bos taurus ADIB	-	--	-
Equus caballus ADIB	-	--	-
Canis lupus familiaris ADIB	-	--	-
Myotis lucifugus ADIB	-	--	-
Loxodonta africana ADIB	-	--	-
Homo sapiens ADIC	-	--	-
Macaca mulatta ADIC	-	--	-
Papio hamadryas ADIC	-	--	-
Callithrix jacchus ADIC	-	--	-
Mus musculus Adic	-	--	-
Rattus norvegicus Adic	-	--	-
Cavia porcellus ADIC	-	--	-
Oryctolagus cuniculus ADIC	-	--	-
Equus caballus ADIC	-	--	-
Canis lupus familiaris ADIC	-	--	-
Myotis lucifugus ADIC	-	--	-
Loxodonta africana ADIC	-	--	-
Homo sapiens ADID	-	--	-
Pan troglodytes ADID	-	--	-
Pongo abelii ADID	-	--	-
Nomascus leucogenys ADID	-	--	-
Macaca mulatta ADID	-	--	-
Otolemur garnettii ADID	-	--	-
Mus musculus Adid	-	--	-
Rattus norvegicus Adid	-	--	-
Cavia porcellus ADID	-	--	-
Oryctolagus cuniculus ADID	-	--	-
Bos taurus ADID	-	--	-
Equus caballus ADID	1	--MG	2
Canis lupus familiaris ADID	-	--	-
Felis catus ADID	-	--	-
Myotis lucifugus ADID	-	--	-
Dasypus novemcinctus ADID	-	--	-
Loxodonta africana ADID	-	--	-
Homo sapiens ADIE	-	--	-
Pan troglodytes ADIE	-	--	-
Pongo abelii ADIE	-	--	-
Papio hamadryas ADIE	-	--	-
Callithrix jacchus ADIE	-	--	-
Otolemur garnettii ADIE	-	--	-
Mus musculus Adie	-	--	-
Rattus norvegicus Adie	-	--	-
Homo sapiens ADIF1	-	--	-
Homo sapiens ADIF2	-	--	-
Pan troglodytes ADIF1	-	--	-
Pongo abelii ADIF1	-	--	-
Nomascus leucogenys ADIF1	-	--	-
Macaca mulatta ADIF1	-	--	-
Papio hamadryas ADIF1	-	--	-

Microcebus murinus ADIF1	-	--	-
Otolemur garnettii ADIF1	-	--	-
Mus musculus Adif1	-	--	-
Rattus norvegicus Adif1	-	--	-
Cavia porcellus ADIF1	-	--	-
Oryctolagus cuniculus ADIF1	-	--	-
Ochotona princeps ADIF1	-	--	-
Bos taurus ADIF1	-	--	-
Equus caballus ADIF1	-	--	-
Canis lupus familiaris ADIF1	-	--	-
Felis catus ADIF1	-	--	-
Dasypus novemcinctus ADIF1	-	--	-
Loxodonta africana ADIF1	-	--	-
Homo sapiens ADIG	-	--	-
Pan troglodytes ADIG	-	--	-
Pongo abelii ADIG	-	--	-
Callithrix jacchus ADIG	-	--	-
Otolemur garnettii ADIG	-	--	-
Mus musculus Adig	-	--	-
Rattus norvegicus Adig	-	--	-
Cavia porcellus ADIG	-	--	-
Oryctolagus cuniculus ADIG	-	--	-
Tursiops truncatus ADIG	-	--	-
Bos taurus ADIG	-	--	-
Equus caballus ADIG	-	--	-
Canis lupus familiaris ADIG	-	--	-
Myotis lucifugus ADIG	-	--	-
Homo sapiens ADIH	-	--	-
Pan troglodytes ADIH	-	--	-
Pongo abelii ADIH	-	--	-
Nomascus leucogenys ADIH	-	--	-
Macaca mulatta ADIH	-	--	-
Papio hamadryas ADIH	-	--	-
Mus musculus Adih	-	--	-
Rattus norvegicus Adih	-	--	-
Dipodomys ordii ADIH	-	--	-
Cavia porcellus ADIH	-	--	-
Bos taurus ADIH	-	--	-
Equus caballus ADIH	-	--	-
Canis lupus familiaris ADIH	-	--	-
Pteropus vampyrus ADIH	-	--	-
Loxodonta africana ADIH	-	--	-
Homo sapiens ADII	-	--	-
Pongo abelii ADII	-	--	-
Macaca mulatta ADII	-	--	-
Mus musculus Adii	-	--	-
Rattus norvegicus Adii	-	--	-
Bos taurus ADII	-	--	-
Canis lupus familiaris ADII	-	--	-
Dasypus novemcinctus ADII	-	--	-
Homo sapiens ADIJ	-	--	-
Pan troglodytes ADIJ	-	--	-
Papio hamadryas ADIJ	-	--	-
Mus musculus Adij	-	--	-
Rattus norvegicus Adij	-	--	-
Oryctolagus cuniculus ADIJ	-	--	-
Bos taurus ADIJ	-	--	-
Canis lupus familiaris ADIJ	-	--	-
Loxodonta africana ADIJ	-	--	-
Homo sapiens ADIK	-	--	-
Pan troglodytes ADIK	-	--	-
Gorilla gorilla ADIK	-	--	-
Papio hamadryas ADIK	-	--	-
Callithrix jacchus ADIK	-	--	-
Rattus norvegicus Adik	-	--	-
Cavia porcellus ADIK	-	--	-
Equus caballus ADIK	-	--	-
Canis lupus familiaris ADIK	-	--	-
Dasypus novemcinctus ADIK	-	--	-
Loxodonta africana ADIK	-	--	-
Mus musculus Adil	-	--	-
Rattus norvegicus Adil	-	--	-
Homo sapiens ADIM	1	MLSVPSWLCMFSSHFNRNVPRCDIYEFRQLGTVHTNRSCLWCGKMMNGGCSAWGGGLLSL	60
Pan troglodytes ADIM	1	MLSVPSWLCMFSSHFNRNVPRCDIYEFRQLGTVHTNRSCLWCGKMMNGGCSAWGGGLLSL	60

```
Pongo abelii ADIM               1 --------------------------------------------MMNGGCSAWGGGLLSL  16
Nomascus leucogenys ADIM        1 MLSVPSWLCMFSSHFNRNVPRCDIYEFRQLGTVHTNRSCLWCGKMMNGGCSAWGGGLLSL  60
Papio hamadryas ADIM            1 MLSVPSWLCMFSSHFNRNVPRCDIYEFRQLGTVHTNRSCLWCGKMMNGGCSAWGGGLLSL  60
Callithrix jacchus ADIM         1 ---------MFSSHFNRNVPRCDTYESRQLESVHTDRSCLWCGKVMNGGCSARGGGLLSL  51
Mus musculus Adim               - ------------------------------------------------------------   -
Rattus norvegicus Adim          1 ---------------------------------------------MGAGLSGVGGSCCHS  15
Cavia porcellus ADIM            1 ---------------------------------------------MGAVLPG-EEGCCHS  14
Bos taurus ADIM                 - ------------------------------------------------------------   -
Equus caballus ADIM             - ------------------------------------------------------------   -
Canis lupus familiaris ADIM     1 ---------------------------------------------MNGGRPAWGGGLLPS  15
Homo sapiens ADIN               - ------------------------------------------------------------   -
Pan troglodytes ADIN            - ------------------------------------------------------------   -
Pongo abelii ADIN               - ------------------------------------------------------------   -
Nomascus leucogenys ADIN        - ------------------------------------------------------------   -
Macaca mulatta ADIN             - ------------------------------------------------------------   -
Callithrix jacchus ADIN         - ------------------------------------------------------------   -
Mus musculus Adin               - ------------------------------------------------------------   -
Cavia porcellus ADIN            - ------------------------------------------------------------   -
Tursiops truncatus ADIN         - ------------------------------------------------------------   -
Canis lupus familiaris ADIN     - ------------------------------------------------------------   -
Dasypus novemcinctus ADIN       - ------------------------------------------------------------   -
Loxodonta africana ADIN         - ------------------------------------------------------------   -
Homo sapiens ADIO               - ------------------------------------------------------------   -
Pan troglodytes ADIO            - ------------------------------------------------------------   -
Macaca mulatta ADIO             - ------------------------------------------------------------   -
Bos taurus ADIO                 - ------------------------------------------------------------   -
Myotis lucifugus ADIO           - ------------------------------------------------------------   -
Homo sapiens ADIP               1 ----------------------MCVECVHVWGLHVCECVCMCVCARECVVSACMHAWVCE  38
Otolemur garnettii ADIP         - ------------------------------------------------------------   -
Mus musculus Adip               - ------------------------------------------------------------   -
Cavia porcellus ADIP            - ------------------------------------------------------------   -
Bos taurus ADIP                 - ------------------------------------------------------------   -
Equus caballus ADIP             - ------------------------------------------------------------   -
Loxodonta africana ADIP         - ------------------------------------------------------------   -
Homo sapiens ADIQ               - ------------------------------------------------------------   -
Pan troglodytes ADIQ            - ------------------------------------------------------------   -
Pongo abelii ADIQ               - ------------------------------------------------------------   -
Nomascus leucogenys ADIQ        - ------------------------------------------------------------   -
Macaca mulatta ADIQ             - ------------------------------------------------------------   -
Mus musculus Adiq               - ------------------------------------------------------------   -
Rattus norvegicus Adiq          - ------------------------------------------------------------   -
Homo sapiens ADIR               - ------------------------------------------------------------   -
Pan troglodytes ADIR            - ------------------------------------------------------------   -
Pongo abelii ADIR               - ------------------------------------------------------------   -
Nomascus leucogenys ADIR        - ------------------------------------------------------------   -
Macaca mulatta ADIR             - ------------------------------------------------------------   -
Papio hamadryas ADIR            - ------------------------------------------------------------   -
Callithrix jacchus ADIR         - ------------------------------------------------------------   -
Tarsius syrichta ADIR           - ------------------------------------------------------------   -
Otolemur garnettii ADIR         - ------------------------------------------------------------   -
Tupaia belangeri ADIR           - ------------------------------------------------------------   -
Mus musculus Adir               - ------------------------------------------------------------   -
Rattus norvegicus Adir          - ------------------------------------------------------------   -
Cavia porcellus ADIR            - ------------------------------------------------------------   -
Oryctolagus cuniculus ADIR      - ------------------------------------------------------------   -
Tursiops truncatus ADIR         - ------------------------------------------------------------   -
Bos taurus ADIR                 - ------------------------------------------------------------   -
Equus caballus ADIR             - ------------------------------------------------------------   -
Canis lupus familiaris ADIR     - ------------------------------------------------------------   -
Myotis lucifugus ADIR           - ------------------------------------------------------------   -
Pteropus vampyrus ADIR          - ------------------------------------------------------------   -
Erinaceus europaeus ADIR        - ------------------------------------------------------------   -
Dasypus novemcinctus ADIR       - ------------------------------------------------------------   -
Choloepus hoffmanni ADIR        - ------------------------------------------------------------   -
Loxodonta africana ADIR         - ------------------------------------------------------------   -
```

```
Homo sapiens ADIA              1 ----------------------------------------------------------MM   2
                                         70        80        90       100       110       120
Homo sapiens ADIA              1 ----------------------------------------------------------MM   2
Papio hamadryas ADIA           1 ----------------------------------------------------------MM   2
Mus musculus Adia              1 -------------------------------MDGLMTDGSYHGSNPPCVLTPQEAPEAEL  29
Rattus norvegicus Adia         - ------------------------------------------------------------   -
Cavia porcellus ADIA           - ------------------------------------------------------------   -
Ochotona princeps ADIA         1 ----------------------------------------------------------MA   2
Bos taurus ADIA                - ------------------------------------------------------------   -
Equus caballus ADIA            - ------------------------------------------------------------   -
Canis lupus familiaris ADIA    - ------------------------------------------------------------   -
Felis catus ADIA               - ------------------------------------------------------------   -
Myotis lucifugus ADIA          - ------------------------------------------------------------   -
Dasypus novemcinctus ADIA      - ------------------------------------------------------------   -
Loxodonta africana ADIA        - ------------------------------------------------------------   -
Homo sapiens ADIB              - ------------------------------------------------------------   -
Pan troglodytes ADIB           - ------------------------------------------------------------   -
Pongo abelii ADIB              - ------------------------------------------------------------   -
Nomascus leucogenys ADIB       - ------------------------------------------------------------   -
Callithrix jacchus ADIB        - ------------------------------------------------------------   -
Otolemur garnettii ADIB        - ------------------------------------------------------------   -
Mus musculus Adib              1 ------------------------------MKGYGRGRGNEEREGEFSTSSSISPPPLLR  30
Rattus norvegicus Adib         1 -------------------------------------MGNEEREGELSTSSSISPPFLLR  23
Cavia porcellus ADIB           1 ---------MWQGPPITLVGAQAAGGGVQGLCGARPGAVGRKEGRDWRLTPHSASSFLPK  51
Oryctolagus cuniculus ADIB     1 -------MPASEAWHQGRLGWRVPRGLGWGPWGGSEDEQGTRGARPGGLSPCLPYHSLPS  53
Bos taurus ADIB                - ------------------------------------------------------------   -
Equus caballus ADIB            1 ------------------------------------------MRGWVVSPALSSSTSFPR  18
Canis lupus familiaris ADIB    - ------------------------------------------------------------   -
Myotis lucifugus ADIB          - ------------------------------------------------------------   -
Loxodonta africana ADIB        - ------------------------------------------------------------   -
Homo sapiens ADIC              - ------------------------------------------------------------   -
Macaca mulatta ADIC            - ------------------------------------------------------------   -
Papio hamadryas ADIC           - ------------------------------------------------------------   -
Callithrix jacchus ADIC        - ------------------------------------------------------------   -
Mus musculus Adic              - ------------------------------------------------------------   -
Rattus norvegicus Adic         1 ------------------------------------------------MPGVPVCLCRNI  12
Cavia porcellus ADIC           - ------------------------------------------------------------   -
Oryctolagus cuniculus ADIC     1 ------------------MRLCPVHACLRVRLRFWACVRGRGGGEPERRPSSHLCLHRHH  42
Equus caballus ADIC            1 ---------------------------MSSSGGIQVRKLGVHERVMSACRGPAPCLHSGI  33
Canis lupus familiaris ADIC    1 -----------------------------------------MGVCACVRSGPDPCLRSGI  19
Myotis lucifugus ADIC          - ------------------------------------------------------------   -
Loxodonta africana ADIC        1 ---------------------------------------------MSSLPSPTLCLHSAI  15
Homo sapiens ADID              1 -----------------------------MDGVLCRSQLGVCVGSVSPWLTVHMWIPGLR  31
Pan troglodytes ADID           1 -----------------------------MDGVLCRSQLGVCVGSVSPWLTVHMWIPGLR  31
Pongo abelii ADID              - ------------------------------------------------------------   -
Nomascus leucogenys ADID       1 -----------------------------MESCVGPNWV-CCVGSVSPWLTVHMWIPGLR  30
Macaca mulatta ADID            1 -----------------------------MESCVGISWV-CCVGSVSPWLTVHMWIPGLR  30
Otolemur garnettii ADID        - ------------------------------------------------------------   -
Mus musculus Adid              1 -------------------------------------------------MTIHLSISGLR  11
Rattus norvegicus Adid         - ------------------------------------------------------------   -
Cavia porcellus ADID           1 ------------------MESSFMSVPCTAGLSLGGSCLGCSERSASPWLTAHSHIPGLR  42
Oryctolagus cuniculus ADID     1 --------------------------------------------------MGHVCIPGLR  10
Bos taurus ADID                - ------------------------------------------------------------   -
Equus caballus ADID            3 QCVSGCLTRSRKDYVCVCACAYMCVQVCVCMYMYVCVWVGCAVWGLLSWLTVQIWIPGLR  62
Canis lupus familiaris ADID    1 ---------------------------------------------------MQIWVPGLR   9
Felis catus ADID               - ------------------------------------------------------------   -
Myotis lucifugus ADID          1 ---------------------------------------------------MAGLISGLR   9
Dasypus novemcinctus ADID      - ------------------------------------------------------------   -
Loxodonta africana ADID        - ------------------------------------------------------------   -
Homo sapiens ADIE              - ------------------------------------------------------------   -
Pan troglodytes ADIE           - ------------------------------------------------------------   -
Pongo abelii ADIE              - ------------------------------------------------------------   -
Papio hamadryas ADIE           - ------------------------------------------------------------   -
Callithrix jacchus ADIE        - ------------------------------------------------------------   -
Otolemur garnettii ADIE        - ------------------------------------------------------------   -
Mus musculus Adie              - ------------------------------------------------------------   -
Rattus norvegicus Adie         - ------------------------------------------------------------   -
Homo sapiens ADIF1             - ------------------------------------------------------------   -
Homo sapiens ADIF2             - ------------------------------------------------------------   -
Pan troglodytes ADIF1          - ------------------------------------------------------------   -
Pongo abelii ADIF1             - ------------------------------------------------------------   -
Nomascus leucogenys ADIF1      - ------------------------------------------------------------   -
Macaca mulatta ADIF1           - ------------------------------------------------------------   -
Papio hamadryas ADIF1          - ------------------------------------------------------------   -
```

```
Microcebus murinus ADIF1        - ------------------------------------------------------------   -
Otolemur garnettii ADIF1        - ------------------------------------------------------------   -
Mus musculus Adif1              1 -------------------------------MPSNCTCTQGLYILQFLFSLFQLRPCGLT  29
Rattus norvegicus Adif1         1 ------------------------------------------MPLPVSVSLFQSRPCRLT  18
Cavia porcellus ADIF1           - ------------------------------------------------------------   -
Oryctolagus cuniculus ADIF1     - ------------------------------------------------------------   -
Ochotona princeps ADIF1         - ------------------------------------------------------------   -
Bos taurus ADIF1                - ------------------------------------------------------------   -
Equus caballus ADIF1            - ------------------------------------------------------------   -
Canis lupus familiaris ADIF1    - ------------------------------------------------------------   -
Felis catus ADIF1               - ------------------------------------------------------------   -
Dasypus novemcinctus ADIF1      - ------------------------------------------------------------   -
Loxodonta africana ADIF1        - ------------------------------------------------------------   -
Homo sapiens ADIG               1 -----MKVNSVLHVWKDEHWSDLLQMHILLFKLIQSCQTTPLP-TQKVHLRLCVSLPEPK  54
Pan troglodytes ADIG            1 -----MKVNRVLHVWKDEHWSDLLQMHILMFKLIQSCQTTPLP-TQKVHLRLCVSLPEPK  54
Pongo abelii ADIG               1 -----MKVNRVLHVWKDEHWSDLLQMHILMFKLIQSCQTTPLP-TQKAHLRPCVSLPEPK  54
Callithrix jacchus ADIG         1 -------------------MCGKINTELLVFRLIQSCQTTPLL-TQKVHLCPCVSLPEPK  40
Otolemur garnettii ADIG         - ------------------------------------------------------------   -
Mus musculus Adig               1 ----------------MK-KANARGDCTHAHSSAHPNSIMPKPFPGE-STDVFTSLLEPK  42
Rattus norvegicus Adig          1 ----------------MGGKANAGGQLSTAHSSAHPNSIAPKPFPGE-YTDVCTSLLEPK  43
Cavia porcellus ADIG            - ------------------------------------------------------------   -
Oryctolagus cuniculus ADIG      - ------------------------------------------------------------   -
Tursiops truncatus ADIG         1 --------MELLGQTGYLDTHGEVSPGVNFCKCPFSCSVMPKTPPKRVHLCPCVSLPEPK  52
Bos taurus ADIG                 - ------------------------------------------------------------   -
Equus caballus ADIG             1 -------------------MHGKVSTGVNYCKCPFLCSIMPKPPPERVHLCPCVSLPEPK  41
Canis lupus familiaris ADIG     1 -------------------MHGEVSTGVSGCKRLFSCSFMPKLSPERVHLCPCVSFPEPK  41
Myotis lucifugus ADIG           - ------------------------------------------------------------   -
Homo sapiens ADIH               - ------------------------------------------------------------   -
Pan troglodytes ADIH            - ------------------------------------------------------------   -
Pongo abelii ADIH               - ------------------------------------------------------------   -
Nomascus leucogenys ADIH        - ------------------------------------------------------------   -
Macaca mulatta ADIH             - ------------------------------------------------------------   -
Papio hamadryas ADIH            - ------------------------------------------------------------   -
Mus musculus Adih               1 -----------------------------------------------------MLLQVTT   7
Rattus norvegicus Adih          1 ----------------MQLHPCLLLLLLIPLIFPPMPYWSVVCQHHVHSYLLFCHPQVTT  44
Dipodomys ordii ADIH            - ------------------------------------------------------------   -
Cavia porcellus ADIH            - ------------------------------------------------------------   -
Bos taurus ADIH                 1 -------------------------------------------MARLTYSQLSCLPQVAT  17
Equus caballus ADIH             - ------------------------------------------------------------   -
Canis lupus familiaris ADIH     - ------------------------------------------------------------   -
Pteropus vampyrus ADIH          - ------------------------------------------------------------   -
Loxodonta africana ADIH         - ------------------------------------------------------------   -
Homo sapiens ADII               - ------------------------------------------------------------   -
Pongo abelii ADII               - ------------------------------------------------------------   -
Macaca mulatta ADII             - ------------------------------------------------------------   -
Mus musculus Adii               - ------------------------------------------------------------   -
Rattus norvegicus Adii          - ------------------------------------------------------------   -
Bos taurus ADII                 - ------------------------------------------------------------   -
Canis lupus familiaris ADII     - ------------------------------------------------------------   -
Dasypus novemcinctus ADII       - ------------------------------------------------------------   -
Homo sapiens ADIJ               - ------------------------------------------------------------   -
Pan troglodytes ADIJ            - ------------------------------------------------------------   -
Papio hamadryas ADIJ            - ------------------------------------------------------------   -
Mus musculus Adij               - ------------------------------------------------------------   -
Rattus norvegicus Adij          - ------------------------------------------------------------   -
Oryctolagus cuniculus ADIJ      - ------------------------------------------------------------   -
Bos taurus ADIJ                 - ------------------------------------------------------------   -
Canis lupus familiaris ADIJ     - ------------------------------------------------------------   -
Loxodonta africana ADIJ         - ------------------------------------------------------------   -
Homo sapiens ADIK               - ------------------------------------------------------------   -
Pan troglodytes ADIK            - ------------------------------------------------------------   -
Gorilla gorilla ADIK            - ------------------------------------------------------------   -
Papio hamadryas ADIK            - ------------------------------------------------------------   -
Callithrix jacchus ADIK         - ------------------------------------------------------------   -
Rattus norvegicus Adik          - ------------------------------------------------------------   -
Cavia porcellus ADIK            - ------------------------------------------------------------   -
Equus caballus ADIK             - ------------------------------------------------------------   -
Canis lupus familiaris ADIK     - ------------------------------------------------------------   -
Dasypus novemcinctus ADIK       - ------------------------------------------------------------   -
Loxodonta africana ADIK         - ------------------------------------------------------------   -
Mus musculus Adil               - ------------------------------------------------------------   -
Rattus norvegicus Adil          - ------------------------------------------------------------   -
Homo sapiens ADIM              61 WHAYSLIYYTPKYKTTPP---LQLSAPVLASAR---GDHAPGAL---LSSQGDSEALLRI 111
Pan troglodytes ADIM           61 WHAYSLIYYTPKYKTTPP---LQLSAPVLASAR---GDHAPGAL---LSSQGDSEALLRI 111
```

```
Pongo abelii ADIM                 17 WHSYSLIYYTPKYKTTPP---LQLSAPVLASAR---GDHAPGAL---LSSQGDSEALLRI  67
Nomascus leucogenys ADIM          61 WHAYSLIYYTPKYKTTPP---LQLSAPVLASAR---GDHAPGAL---LSSQGDSEALLRI 111
Papio hamadryas ADIM              61 WHAYSLIYYTPKYKTTPP---LQLSAPVLASAR---GDHAPGAL---LSSQGSSEALLRI 111
Callithrix jacchus ADIM           52 WHAYSLIYYTPNYKTKLRAASALSSSPGIHPRRPR-SWKEP---------QGGSEALLRI 101
Mus musculus Adim                  - ------------------------------------------------------------   -
Rattus norvegicus Adim            16 GMTAALFITHQKYKTTQPAA-ALISKPGICLRRLRCGCRVPGAYSPHQLPRGDSADLSRA  74
Cavia porcellus ADIM              15 GVSAALFITHQSIKPPSPAA-VLIFSPGICLRRSR-FLKPP------LSSQGGSVALLKI  66
Bos taurus ADIM                    - ------------------------------------------------------------   -
Equus caballus ADIM                - ------------------------------------------------------------   -
Canis lupus familiaris ADIM       16 GMPAALFITHQSIKPPSR---LQLASPALAFAREA-PPSSP----------GGSGALLRN  61
Homo sapiens ADIN                  - ------------------------------------------------------------   -
Pan troglodytes ADIN               - ------------------------------------------------------------   -
Pongo abelii ADIN                  - ------------------------------------------------------------   -
Nomascus leucogenys ADIN           - ------------------------------------------------------------   -
Macaca mulatta ADIN                - ------------------------------------------------------------   -
Callithrix jacchus ADIN            - ------------------------------------------------------------   -
Mus musculus Adin                  - ------------------------------------------------------------   -
Cavia porcellus ADIN               1 -------------------------------------------------MSPVLPVSTGA  11
Tursiops truncatus ADIN            - ------------------------------------------------------------   -
Canis lupus familiaris ADIN        - ------------------------------------------------------------   -
Dasypus novemcinctus ADIN          - ------------------------------------------------------------   -
Loxodonta africana ADIN            - ------------------------------------------------------------   -
Homo sapiens ADIO                  - ------------------------------------------------------------   -
Pan troglodytes ADIO               - ------------------------------------------------------------   -
Macaca mulatta ADIO                - ------------------------------------------------------------   -
Bos taurus ADIO                    - ------------------------------------------------------------   -
Myotis lucifugus ADIO              - ------------------------------------------------------------   -
Homo sapiens ADIP                 39 SDCPPRSCVTAAFPSGVRLGKHLRFPGCFSPQFSGCFSVQLLPNSVPSLCVSFPPGPGRK  98
Otolemur garnettii ADIP            - ------------------------------------------------------------   -
Mus musculus Adip                  - ------------------------------------------------------------   -
Cavia porcellus ADIP               - ------------------------------------------------------------   -
Bos taurus ADIP                    1 ----------------------------------MGLFSPWGSSSPHPPCVS-PAGLGGK  25
Equus caballus ADIP                - ------------------------------------------------------------   -
Loxodonta africana ADIP            1 -----------------MVIAGLGKRPSFLAASPRSPIPTRPLPHLSPCGVSFPPGHGRK  43
Homo sapiens ADIQ                  1 -----------------------------------------MQWLRVRESPGEATGHRVT  19
Pan troglodytes ADIQ               1 -----------------------------------------MQWLRVRESPGEATGHRVT  19
Pongo abelii ADIQ                  1 -----------------------------------------MQWLRVRESPGEATGHRVT  19
Nomascus leucogenys ADIQ           1 -----------------------------------------MQWLRVRESPGEATGHRVT  19
Macaca mulatta ADIQ                1 -----------------------------------------MQWLRVRDSPGEATGHRVT  19
Mus musculus Adiq                  1 --------------------------------------------------------MRVI   4
Rattus norvegicus Adiq             1 --------------------------------------------------------MRVI   4
Homo sapiens ADIR                  - ------------------------------------------------------------   -
Pan troglodytes ADIR               - ------------------------------------------------------------   -
Pongo abelii ADIR                  - ------------------------------------------------------------   -
Nomascus leucogenys ADIR           - ------------------------------------------------------------   -
Macaca mulatta ADIR                - ------------------------------------------------------------   -
Papio hamadryas ADIR               - ------------------------------------------------------------   -
Callithrix jacchus ADIR            - ------------------------------------------------------------   -
Tarsius syrichta ADIR              - ------------------------------------------------------------   -
Otolemur garnettii ADIR            - ------------------------------------------------------------   -
Tupaia belangeri ADIR              - ------------------------------------------------------------   -
Mus musculus Adir                  - ------------------------------------------------------------   -
Rattus norvegicus Adir             - ------------------------------------------------------------   -
Cavia porcellus ADIR               - ------------------------------------------------------------   -
Oryctolagus cuniculus ADIR         - ------------------------------------------------------------   -
Tursiops truncatus ADIR            - ------------------------------------------------------------   -
Bos taurus ADIR                    - ------------------------------------------------------------   -
Equus caballus ADIR                - ------------------------------------------------------------   -
Canis lupus familiaris ADIR        - ------------------------------------------------------------   -
Myotis lucifugus ADIR              - ------------------------------------------------------------   -
Pteropus vampyrus ADIR             - ------------------------------------------------------------   -
Erinaceus europaeus ADIR           - ------------------------------------------------------------   -
Dasypus novemcinctus ADIR          - ------------------------------------------------------------   -
Choloepus hoffmanni ADIR           - ------------------------------------------------------------   -
Loxodonta africana ADIR            - ------------------------------------------------------------   -
```

```
Homo sapiens ADIA                 3 MKIP-WGSIPV-LMLL-------LLL----GLIDISQAQ-L------------------  29
                                          130       140       150       160       170       180

Homo sapiens ADIA                 3 MKIP-WGSIPV-LMLL-------LLL----GLIDISQAQ-L------------------  29
Papio hamadryas ADIA              3 MKIL-WGSIPV-LMLL-------LLL----GLLDVSWAQ-G------------------  29
Mus musculus Adia                30 MKTQ-WGEVWT-HLLL-------LLL----GFLHVSWAQ-S------------------  56
Rattus norvegicus Adia            1 MKTQ-WSEILT-PLLL-------LLL----GLLHVSWAQ-S------------------  27
Cavia porcellus ADIA              1 MKIP-WGDVSV-LLLL-------LLL----PLLRVSRVQ-S------------------  27
Ochotona princeps ADIA            3 MKSP-RGSIPA---LL-------LLL----SLLDASQAQ-S------------------  27
Bos taurus ADIA                   1 MKTP-RGSVLV----L-------LLL----NLLRVSWAQ-S------------------  24
Equus caballus ADIA               1 MKTLTMGGILA-LLPL-------LLL----GPLDVSRAQ-S------------------  28
Canis lupus familiaris ADIA       1 MKTP-RGGILA-LLLP-------LLL----GLLEVSWAQ--------------------  26
Felis catus ADIA                  1 MKTV-RDGVLASLLLL-------LLLF--LFLLEVSWAQ-S------------------  30
Myotis lucifugus ADIA             1 MKTP-RGGLLA-LLLP-------LLL----GPLDVSQAQ-R------------------  27
Dasypus novemcinctus ADIA         1 MKTL-WGGVLP----L-------LLL----SLPDASCAQ-S------------------  24
Loxodonta africana ADIA           1 MKTL-WAGIPV-LLPL-------LLL----SLLGVSWAQ-I------------------  27
Homo sapiens ADIB                 1 MDVG-PSSLPHLGLKL-------LLL--LLLLPLRGQAN-T------------------  30
Pan troglodytes ADIB              1 MDMG-PSSLPHLGLKL-------LLL--LLLLPLRGQAN-T------------------  30
Pongo abelii ADIB                 1 MDMG-PSSLPHLGLKL-------LLP--LLLLPLRGQAN-T------------------  30
Nomascus leucogenys ADIB          1 MDVG-PSSLPHLGLKL-------LLLL-LLLLPLRGQAN-T------------------  31
Callithrix jacchus ADIB           1 MDVG-HGS--HFGLNL-------LLLL-LLLLPLRSQAN-T------------------  29
Otolemur garnettii ADIB           1 M-V--PNCRPHLQLNL-------LLL--LLVLPLGGQAS-T------------------  28
Mus musculus Adib                31 MVVG-PSCQPPCGLCL-------LLLF-LLALPLRSQAS-A------------------  61
Rattus norvegicus Adib           24 MVVG-TSCQPQHGLYL-------LLL--LLALPLRSQAN-A------------------  53
Cavia porcellus ADIB             52 MDVG-L----PLGLHL-------LLL--LLAVPLRGQTR-P------------------  77
Oryctolagus cuniculus ADIB       54 MDVG-LGSQSPLGLHL-------LLL--LLALPLGSQAS-A------------------  83
Bos taurus ADIB                   1 MGSG-A--WPLLVLNL-------LLL--LLARPLRGQTE-T------------------  28
Equus caballus ADIB              19 MDVG-SSSRLPLGLHL-------LLL--LLALPLGGQAS-T------------------  48
Canis lupus familiaris ADIB       1 MDTG-PSSWPHLGLNL-------LLL--LLALPLGGQAS-T------------------  30
Myotis lucifugus ADIB             1 MDRG-SSIWLPLGLNL-------LLL--LLALPLGGLAA-T------------------  30
Loxodonta africana ADIB           1 M--G-PGSQPPLSLNL-------LLL--LLTLPLRGQAS-T------------------  28
Homo sapiens ADIC                 1 MEGP-RG-WLVLCVLA-------ISL----AS-MV--TE-D------------------  24
Macaca mulatta ADIC              1 MEGP-QG-WLVVCVLA-------ISL----AS-IV--TQ-N------------------  24
Papio hamadryas ADIC              1 MEGP-QG-WLVVCVLA-------ISL----AS-IV--TQ-N------------------  24
Callithrix jacchus ADIC           1 MEGS-QG-WLVVCVLA-------ISL----AS-TV--TQ-D------------------  24
Mus musculus Adic                 1 METS-QG-WLVACVLT-------MTL----VW-TV--AE-D------------------  24
Rattus norvegicus Adic           13 METS-QG-WLVACVLA-------VTL----VW-TV--AE-D------------------  36
Cavia porcellus ADIC              1 M-TP-RL-WLLVCVLA-------VSL----SS-GV--AE-D------------------  23
Oryctolagus cuniculus ADIC       43 METS-T--WLLLCVLA-------TSL----SS-GV--TQ-G------------------  65
Equus caballus ADIC              34 MEAP-WG-WLMVGVLA-------VSL----AS-TG--TQ-D------------------  57
Canis lupus familiaris ADIC      20 MEAP-WG-WLALCVLA-------TSL----AS-AV--TQ-D------------------  43
Myotis lucifugus ADIC             1 MEAP-WG-WLVVGVLA-------MSL----AS-TV--TQ-N------------------  24
Loxodonta africana ADIC          16 MEAP-KG-WLVVCVLA-------VSL----AS-TV--TQ-D------------------  39
Homo sapiens ADID                32 M--L----LLG--AVL-------LLL----ALP--GHDQ-E--TTTQGP-G---------  57
Pan troglodytes ADID             32 M--L----LLG--AVL-------LLL----ALP--GHDQ-E--TTTQGP-G---------  57
Pongo abelii ADID                 1 M--L----LLG--AIL-------LLL----ALP--SHGQ-E--TTTQGP-R---------  26
Nomascus leucogenys ADID         31 M--L----LLG--AVL-------LLL----ALP--SHGQ-D--TTTQGP-G---------  56
Macaca mulatta ADID              31 M-------LLG--AVL-------LLL----ALP--SHGQ-D--TTTQGP-G---------  55
Otolemur garnettii ADID           1 M--L----LLQ--AVL-------LLL----ALP--SHGQ-D--TSTEGP-G---------  26
Mus musculus Adid                12 M--L----LLQ--ALL-------FLL----ILP--SHAE-DDVTTTEEL-A---------  39
Rattus norvegicus Adid            1 M--L----LLQ--ALL-------FLL----ILP--SHEG-I--TATEGP-G---------  26
Cavia porcellus ADID             43 M--L----VLR--AVL-------LLL----ALP--SCAQ-E--TSAAES-E---------  68
Oryctolagus cuniculus ADID       11 M--L----LLQ--AVL-------LLL----ALP--SHGQ-D--STTESP-G---------  36
Bos taurus ADID                   1 M--L----LQG--ALL-------LLL----ALP--SHGE-D--NM-EDP-----------  24
Equus caballus ADID              63 M--L----LLQ--AVL-------LLL----VLP--SPGE-V--TTTEE------------  86
Canis lupus familiaris ADID      10 M--L----LLR--AVL-------LLL----VLP--AHGQ-D--SVAEGP-G---------  35
Felis catus ADID                  1 M--L----LLR--AVL-------LLL----VLP--IRGQ-D--SETEGP-G---------  26
Myotis lucifugus ADID            10 M--L----LLQ--AVL-------LLL----VLP--SRGQ-D--TPTKEP-E---------  35
Dasypus novemcinctus ADID         1 M--L----LLQ--AVL-------LLL----ALP--SHGQ-DT-TTQEEP-G---------  27
Loxodonta africana ADID           1 M--L----LLR--AVL-------LLL----ALP--NNGE-N--LATEGP-G---------  26
Homo sapiens ADIE                 1 MR-P----LLV--LLL-------LGL----A-----AGS-PPL-----D-D---------  21
Pan troglodytes ADIE              1 MR-P----LLV--LLL-------LGL----A-----AGS-PPL-----D-D---------  21
Pongo abelii ADIE                 1 MR-P----LLV--LLL-------LGL----A-----AGS-PPL-----D-D---------  21
Papio hamadryas ADIE              1 MR-P----LLV--LLL-------LGL----A-----AGS-PPL-----D-D---------  21
Callithrix jacchus ADIE           1 MR-P----LLV--LLL-------LGL----A-----ASS-PPL-----E-D---------  21
Otolemur garnettii ADIE           1 MR-L----LLA--LLF-------LGL----A-----AGS-PPL-----D-D---------  21
Mus musculus Adie                 1 MR-P----LLA--LLL-------LGL----V-----SGS-PPL-----D-D---------  21
Rattus norvegicus Adie            1 MR-P----LLA--LLL-------LGL----A-----SGS-PPL-----D-D---------  21
Homo sapiens ADIF1                1 MR-I----WWL--LLA-------IEI-C--TGN--INSQ-D------------------  21
Homo sapiens ADIF2                1 MR-I----WWL--LLA-------IEI-C--TGN--INSQ-D------------------  21
Pan troglodytes ADIF1             1 MR-I----WWF--LLA-------IEI-C--TGN--INSQ-D------------------  21
Pongo abelii ADIF1                1 MR-I----WWF--LLA-------VEI-C--TGN--INSQ-D------------------  21
Nomascus leucogenys ADIF1         1 MR-I----WWL--LLA-------IEI-C--KGN--INSQ-D------------------  21
Macaca mulatta ADIF1              1 MR-I----WWF--LLA-------IGI-C--AGN--ISSQ-N------------------  21
Papio hamadryas ADIF1             1 MR-I----WWF--LLA-------IEI-C--TGN--ISSQ-N------------------  21
```

```
Microcebus murinus ADIF1          1 MK-I----CWL--LLA-------AGI-C--TGN--INAQ-D-------------------  21
Otolemur garnettii ADIF1          1 MK-I----WWL--LLA-------IGI-C--TGN--INAQ-D-------------------  21
Mus musculus Adif1               30 MR-I----WWL--LLV-------MGA-C--TRS--VFSQ-D-------------------  50
Rattus norvegicus Adif1          19 MR-I----WWL--LLV-------MDA-----GS--VFSQ-D-------------------  37
Cavia porcellus ADIF1             1 MR-T----WWL--LLA-------LGV-C--TGA--MSSQ-D-------------------  21
Oryctolagus cuniculus ADIF1       1 MR-I----WWL--LLV-------TGV-C--TGS--VSSQ-D-------------------  21
Ochotona princeps ADIF1           1 MR-I----WWL--LLV-------IGV-C--LGS--VSLQ-D-------------------  21
Bos taurus ADIF1                  1 MR-I----WWL--LLA-------SGV-C--TGM--VSSQ-D-------------------  21
Equus caballus ADIF1              1 MR-I----WWL--LLV-------TGI-C--TGK--VNSQ-D-------------------  21
Canis lupus familiaris ADIF1      1 MR-I----WLL--LLA-------AGI-C--MGN--VKSQ-D-------------------  21
Felis catus ADIF1                 1 MR-T----WLL--LLA-------GGI-C--MGN--VNAQ-D-------------------  21
Dasypus novemcinctus ADIF1        1 MRII----WWV--LLA-------IGI-C--MGS--INSQ-D-------------------  22
Loxodonta africana ADIF1          1 MR-F----WWL--LLA-------VGA-C--TGN--INSQ-D-------------------  21
Homo sapiens ADIG                55 M--F----VLL--YVT-S-----FAI-C--A-----SGQ-PR-GNQLKG-E---------  80
Pan troglodytes ADIG             55 M--F----VLL--YVT-S-----FAI-C--A-----SGQ-PR-GNQLKG-E---------  80
Pongo abelii ADIG                55 M--F----VLL--YVT-S-----FAI-C--A-----SGQ-PR-GNQLKG-E---------  80
Callithrix jacchus ADIG          41 M--F----VLL--YVT-S-----FAI-C--A-----SGQ-PR-GNQLKG-E---------  66
Otolemur garnettii ADIG           1 M--F----VLL--YVT-S-----FAI-C--A-----SGQ-PR-GNQLKG-E---------  26
Mus musculus Adig                43 M--I----VLL--YVT-S-----LAI-C--A-----SGQ-PR-ANQAKG-E---------  68
Rattus norvegicus Adig           44 M--I----VLL--YVT-S-----LAI-C--A-----SGQ-PR-GNQAKG-E---------  69
Cavia porcellus ADIG              1 M--F----VLL--YVT-S-----FAI-C--A-----SGQ-PR-GSQVKG-E---------  26
Oryctolagus cuniculus ADIG        1 M--F----VLL--YVT-S-----FAL-C--A-----SGQ-PR-GHQFKG-E---------  26
Tursiops truncatus ADIG          53 M--F----VLL--YVT-S-----FAI-C--A-----SGQ-PR-GNQFKG-D---------  78
Bos taurus ADIG                   1 M--F----VLL--YVT-S-----FAI-C--A-----SGQ-PR-SNQFKG-E---------  26
Equus caballus ADIG              42 M--F----VLL--YVT-S-----FAI-C--A-----SGQ-PR-GSQFKG-D---------  67
Canis lupus familiaris ADIG      42 M--F----ILL--YVT-S-----FAI-C--A-----SGQ-PR-GNQFKG-E---------  67
Myotis lucifugus ADIG             1 M--F----VLL--YVT-S-----FAI-C--V-----SGQ-PR-GNQFKG-E---------  26
Homo sapiens ADIH                 1 M--I----PWV--LLA-C-----ALP-C--A------AD-PLLGAFARR-D---------  26
Pan troglodytes ADIH              1 M--I----PWV--LLA-C-----ALP-C--A------AD-PLLGAFARR-D---------  26
Pongo abelii ADIH                 1 M--I----PWV--LLA-C-----ALP-C--A------AD-PLLGAFARR-D---------  26
Nomascus leucogenys ADIH          1 M--I----PWV--LLA-C-----ALP-C--A------AD-PLLGAFARR-D---------  26
Macaca mulatta ADIH               1 M--I----PWV--LLA-C-----ALP-C--A------AD-PLLGAFARR-D---------  26
Papio hamadryas ADIH              1 M--I----PWV--LLA-C-----ALP-C--A------AD-PLLGAFARR-D---------  26
Mus musculus Adih                 8 M--I----SWV--LLA-C-----ALP-C--A------AD-PMLGAFARR-D---------  33
Rattus norvegicus Adih           45 M--I----SWM--LLA-C-----ALP-C--A------AD-PMLGAFARR-D---------  70
Dipodomys ordii ADIH              1 M--I----PWV--LLA-C-----ALP-C--A------AD-PLLGPFAHR-D---------  26
Cavia porcellus ADIH              1 M--I----PWV--LLA-C-----ALP-C--A------AD-PLLAAYARR-D---------  26
Bos taurus ADIH                  18 M--I----PWV--LLA-C-----ALP-C--A------AD-PLLAAFARR-D---------  43
Equus caballus ADIH               1 M--I----PWV--LLA-C-----ALP-C--A------AD-PLLGAFARR-D---------  26
Canis lupus familiaris ADIH       1 M--I----PWV--LLA-C-----ALP-C--A------AD-PLLGAFARR-D---------  26
Pteropus vampyrus ADIH            1 M--I----PWV--LLA-C-----ALP-C--A------AD-PLLGAFAHR-D---------  26
Loxodonta africana ADIH           1 M--I----SWV--LLA-C-----ALP-C--A------TD-PLLGAFARR-D---------  26
Homo sapiens ADII                 1 M-------ALG--LLIA----VPLLL----QAAPRGAAHYEMM-----------------  26
Pongo abelii ADII                 1 M-------ALG--LLIA----VPLLL----QAAPRAAAHYEMM-----------------  26
Macaca mulatta ADII               1 M-------ALG--LLIA----VPLLL----QAAPPGAAHYEMM-----------------  26
Mus musculus Adii                 1 M-------ALG--LLIA----VPLLL----QAAPPGAAHYEML-----------------  26
Rattus norvegicus Adii            1 M-------ALG--LLIA----VPLLL----QAAPPGAAHYEML-----------------  26
Bos taurus ADII                   1 M-------ALG--LLIA----VPLLL----QAAPPGAAHYEMM-----------------  26
Canis lupus familiaris ADII       1 M-------ALG--LLIA----VPLLL----QAAPPGAAHYEMM-----------------  26
Dasypus novemcinctus ADII         1 M-------ALA--LLIA----VPLLL----QAAPPGAAHYEMM-----------------  26
Homo sapiens ADIJ                 1 M-------VLL--LVIL----IPVLV----SSA-GTSAHYEML-----------------  25
Pan troglodytes ADIJ              1 M-------VLL--LVIL----IPVLV----SSA-GTSAHYEML-----------------  25
Papio hamadryas ADIJ              1 M-------VLL--LVIL----IPVLV----SSA-GTSAHYEML-----------------  25
Mus musculus Adij                 1 M-------VLL--LVIL----IPVLV----SSA-GTSAHYEML-----------------  25
Rattus norvegicus Adij            1 M-------VLL--LVIL----IPVLV----SSA-GTSAHYEML-----------------  25
Oryctolagus cuniculus ADIJ        1 M-------VLL--LVIL----IPVLV----SSA-GTSAHYEML-----------------  25
Bos taurus ADIJ                   1 M-------VLL--LVIL----IPVLV----SSA-GTSAHYEML-----------------  25
Canis lupus familiaris ADIJ       1 M-------VLL--LVIL----IPVLV----SSA-GTSAHYEML-----------------  25
Loxodonta africana ADIJ           1 M-------VLL--LVIL----IPVLV----SSA-GTSAHYEML-----------------  25
Homo sapiens ADIK                 1 M-------VLL--LLVA----IPLLV----HSS-RGPAHYEML-----------------  25
Pan troglodytes ADIK              1 M-------VLL--LLVA----IPLLV----HSS-RGPAHYEML-----------------  25
Gorilla gorilla ADIK              1 M-------VLL--LLVA----IPLLV----HSS-RGPAHYEML-----------------  25
Papio hamadryas ADIK              1 M-------VLL--LLVA----IPLLV----HSS-RGPAHYEML-----------------  25
Callithrix jacchus ADIK           1 M-------VLL--LLVA----IPLLV----HSS-RGPAHYEML-----------------  25
Rattus norvegicus Adik            1 M-------VLL--LLVA----IPLLV----HSS-RGPTHYEML-----------------  25
Cavia porcellus ADIK              1 M-------VLL--LLVA----IPLLV----HSS-RGPAHYEML-----------------  25
Equus caballus ADIK               1 M-------VLL--LLVA----IPLLV----QSS-RGPAHYEML-----------------  25
Canis lupus familiaris ADIK       1 M-------VLL--LLVA----IPLLV----HSS-RGPAHYEML-----------------  25
Dasypus novemcinctus ADIK         1 M-------VLL--LLVA----IPLLV----HSS-RGPAHYEML-----------------  25
Loxodonta africana ADIK           1 M-------VLL--LLVA----IPLLV----HSS-REPAHYEML-----------------  25
Mus musculus Adil                 1 M-------LLV--LVVL----IPVLV----SSG-GPDGHYEML-----------------  25
Rattus norvegicus Adil            1 M-------LLV--LVVL----IPVLV----SSG-GPDGHYEML-----------------  25
Homo sapiens ADIM               112 M--L-WRQLIY--WQLL--A-LFFLPFC--------LCQDEYMESPQTG-G--------- 145
Pan troglodytes ADIM            112 M--L-WRQLVY--WQLL--A-LFFLPFC--------LCQDEYMESPQTG-G--------- 145
```

Pongo abelii ADIM	68	M--L-WRQLIY--WQLL--A-LFFLPFC--------LCQDEYMESPQTG-G---------	101
Nomascus leucogenys ADIM	112	M--L-WRQLVY--WQLL--A-LFFLPFC--------LCQDEYMESPQTG-G---------	145
Papio hamadryas ADIM	112	M--L-WRQLVY--WQLL--A-LFFLPFC--------LCQDEYMESPQTG-G---------	145
Callithrix jacchus ADIM	102	M--L-WRQLIY--WQLL--A-LFFLPFC--------LCQDEYMESPQTG-G---------	135
Mus musculus Adim	1	M--L-GRQRIW--WHLL--P-LLFLPFC--------LCQDEYMESPQAG-G---------	34
Rattus norvegicus Adim	75	M--L-RRQLVW--WHLL--A-LLFLPFC--------LCQDEYMESPQAG-G---------	108
Cavia porcellus ADIM	67	M--L-GSQLIC--WHLL--A-LFFLPFC--------LCQDDYMEPPQTGGG---------	101
Bos taurus ADIM	1	M--L-GRQLVY--WHLL--A-LLFLPFC--------LCQDEYMESPQTG-G---------	34
Equus caballus ADIM	1	M--L-RRQLVY--WHLL--A-LLFLPFC--------LCQDEYMESPQTG-G---------	34
Canis lupus familiaris ADIM	62	M--L-GRQLFY--WHLL--A-LLFLPFC--------LCQDEYMESPQTG-G---------	95
Homo sapiens ADIN	1	M-LP----LLL--GLLG-----PAA--C--------WA----------------------	16
Pan troglodytes ADIN	1	M-LP----LLL--GLLG-----PAA--C--------WA----------------------	16
Pongo abelii ADIN	1	M-LP----LLL--VLLG-----PAA--C--------WA----------------------	16
Nomascus leucogenys ADIN	1	M-LP----LLL--GLLG-----PAA--C--------WA----------------------	16
Macaca mulatta ADIN	1	M-LP----LLL--GLLG-----PAA--C--------WA----------------------	16
Callithrix jacchus ADIN	1	M-LP----LLL--GLLG-----PVG--C--------WA----------------------	16
Mus musculus Adin	1	M-LL----LLL--GFLG-----PAA--C--------WA----------------------	16
Cavia porcellus ADIN	12	M-LL----LLF--GLLA-----PAT--C--------WA----------------------	27
Tursiops truncatus ADIN	1	M-LP----LLL--GLLG-----PAA--C--------WA----------------------	16
Canis lupus familiaris ADIN	1	M-LL----LLL--GLLG-----PAA--C--------WA----------------------	16
Dasypus novemcinctus ADIN	1	M-LT----LLL--GLLG-----PVA--C--------WA----------------------	16
Loxodonta africana ADIN	1	M-LP----ILL--GLLG-----PAA--C--------WA----------------------	16
Homo sapiens ADIO	1	M--A----A----PALL--L-LALLL------P-V-GAW-PGL-----------------	21
Pan troglodytes ADIO	1	M--A----A----PALL--L-LALLL------P-A-GAW-PGL-----------------	21
Macaca mulatta ADIO	1	M--A----A----PTLL--L-LALLL------P-A-GAW-PGL-----------------	21
Bos taurus ADIO	1	M--V----A----PALL--LLLA--L------P-A-GAW-RGL-----------------	20
Myotis lucifugus ADIO	1	M--A----A----PGLL--LLLA--L------P-A-GAW-PRL-----------------	20
Homo sapiens ADIP	99	MGSR----GQG--LLLAYCLLLAFA--S--G-L-V-LSRVPHVQGEQQEWE---------	136
Otolemur garnettii ADIP	1	MRSP----GWG--RVLACCLLLAVA--C--G-P-A-LSLVPRIQHEEQEQE---------	38
Mus musculus Adip	1	MGSC----AQG--FMLGCCLLLAIT--W--G-P-I-LSLVPRVQEEQQEWE---------	38
Cavia porcellus ADIP	1	MGSR----GQR--LVLGCCLLLAFA--W--G-L-V-LSHVPRGQREQQEQE---------	38
Bos taurus ADIP	26	MGSR----GLR--LALACCLLLAFA--C--G-L-V-LGRVPHGQQEQQEQE---------	63
Equus caballus ADIP	1	MGSR----GLG--LVLACCLLLTFA--C--G-P-V-LGRVPPGQQEQQEQE---------	38
Loxodonta africana ADIP	44	MGAL----ALG--LQLGCCLVLIFA--C--A-P-V-LGRTPRGQQNQQEHEATPGDSKAE	90
Homo sapiens ADIQ	20	MVTA----ALG--PVWA--ALLLFLLMC--EIPMV-ELTFDRA-----------------	51
Pan troglodytes ADIQ	20	MGTA----ALG--PLWA--ALLLFLLMC--EIPMV-ELTFDRA-----------------	51
Pongo abelii ADIQ	20	MGTA----ALG--PLWA--ALLLFLLMC--EIPTV-ELTFDRA-----------------	51
Nomascus leucogenys ADIQ	20	MGTA----TLG--PLWA--VLLLFILMC--EIPTV-ELTFDRA-----------------	51
Macaca mulatta ADIQ	20	MGTA----ALG--PLWA--VLLLFLLMC--EVPKV-ELTFDRA-----------------	51
Mus musculus Adiq	5	MGIA----SLG--FLWA--VFLLPLVF---GVPTE-ETTFGES-----------------	35
Rattus norvegicus Adiq	5	MGTA----SLG--SIWA--VFLLPLVF---GVPTE-EPTFGES-----------------	35
Homo sapiens ADIR	1	M-TP----VDV--PVTN-----PAA-----------TI----------------------	15
Pan troglodytes ADIR	1	M-TP----VDV--PVTN-----PAA-----------TI----------------------	15
Pongo abelii ADIR	1	M-TP----VDV--PVTN-----PAA-----------TI----------------------	15
Nomascus leucogenys ADIR	1	M-TP----VDV--PVTN-----PAA-----------TI----------------------	15
Macaca mulatta ADIR	1	M-TP----VDV--PVTN-----PAA-----------TI----------------------	15
Papio hamadryas ADIR	1	M-TP----VDV--PVTN-----PAA-----------TI----------------------	15
Callithrix jacchus ADIR	1	M-TP----VDV--PVSN-----PAA-----------TI----------------------	15
Tarsius syrichta ADIR	1	M-TP----VDV--PVTN-----PAA-----------TI----------------------	15
Otolemur garnettii ADIR	1	M-TP----VDV--PVTN-----PAA-----------TV----------------------	15
Tupaia belangeri ADIR	1	M-TP----VDV--PVTN-----PAA-----------TI----------------------	15
Mus musculus Adir	1	M-TP----VDV--PVTS-----PAA-----------AI----------------------	15
Rattus norvegicus Adir	1	M-TP----VDV--PVTS-----PAA-----------AI----------------------	15
Cavia porcellus ADIR	1	M-TP----VDV--PVTN-----PAA-----------TL----------------------	15
Oryctolagus cuniculus ADIR	1	M-TP----VDV--PVTN-----PAA-----------TI----------------------	15
Tursiops truncatus ADIR	1	M-TP----VDV--PVTN-----PAA-----------TI----------------------	15
Bos taurus ADIR	1	M-TP----VDV--PVTN-----PAA-----------TI----------------------	15
Equus caballus ADIR	1	M-TP----VDV--PVTN-----PAA-----------TI----------------------	15
Canis lupus familiaris ADIR	1	M-TP----VDV--PVTN-----PAA-----------TI----------------------	15
Myotis lucifugus ADIR	1	M-TP----VDV--PVTN-----PAA-----------TI----------------------	15
Pteropus vampyrus ADIR	1	M-TP----VDV--PVTS-----PAA-----------TI----------------------	15
Erinaceus europaeus ADIR	-	--	-
Dasypus novemcinctus ADIR	1	M-TP----VDV--PVTS-----PAT-----------TI----------------------	15
Choloepus hoffmanni ADIR	1	M-TP----VDV--PVTN-----PAA-----------TI----------------------	15
Loxodonta africana ADIR	1	M-TP----VDV--PVTN-----PAA-----------TI----------------------	15

```
Homo sapiens ADIA               30 ---------------------------------SC------------------------- 31
                                           190       200       210       220       230       240
Homo sapiens ADIA               30 ---------------------------------SC------------------------- 31
Papio hamadryas ADIA            30 ---------------------------------SC------------------------- 31
Mus musculus Adia               57 ---------------------------------SC------------------------- 58
Rattus norvegicus Adia          28 ---------------------------------SC------------------------- 29
Cavia porcellus ADIA            28 ---------------------------------SC------------------------- 29
Ochotona princeps ADIA          28 ---------------------------------SC------------------------- 29
Bos taurus ADIA                 25 ---------------------------------NC------------------------- 26
Equus caballus ADIA             29 ---------------------------------SC------------------------- 30
Canis lupus familiaris ADIA     27 ---------------------------------SC------------------------- 28
Felis catus ADIA                31 ---------------------------------SC------------------------- 32
Myotis lucifugus ADIA           28 ---------------------------------DC------------------------- 29
Dasypus novemcinctus ADIA       25 ---------------------------------SC------------------------- 26
Loxodonta africana ADIA         28 ---------------------------------SC------------------------- 29
Homo sapiens ADIB               31 ---------------------------------GC------------------------- 32
Pan troglodytes ADIB            31 ---------------------------------GC------------------------- 32
Pongo abelii ADIB               31 ---------------------------------GC------------------------- 32
Nomascus leucogenys ADIB        32 ---------------------------------GC------------------------- 33
Callithrix jacchus ADIB         30 ---------------------------------GC------------------------- 31
Otolemur garnettii ADIB         29 ---------------------------------GC------------------------- 30
Mus musculus Adib               62 ---------------------------------GC------------------------- 63
Rattus norvegicus Adib          54 ---------------------------------GC------------------------- 55
Cavia porcellus ADIB            78 ---------------------------------GC------------------------- 79
Oryctolagus cuniculus ADIB      84 ---------------------------------DC------------------------- 85
Bos taurus ADIB                 29 ---------------------------------YC------------------------- 30
Equus caballus ADIB             49 ---------------------------------DC------------------------- 50
Canis lupus familiaris ADIB     31 ---------------------------------GC------------------------- 32
Myotis lucifugus ADIB           31 ---------------------------------EC------------------------- 32
Loxodonta africana ADIB         29 ---------------------------------SC------------------------- 30
Homo sapiens ADIC               25 ---------------------------------LC------------------------- 26
Macaca mulatta ADIC             25 ---------------------------------VC------------------------- 26
Papio hamadryas ADIC            25 ---------------------------------VC------------------------- 26
Callithrix jacchus ADIC         25 ---------------------------------VC------------------------- 26
Mus musculus Adic               25 ---------------------------------VC------------------------- 26
Rattus norvegicus Adic          37 ---------------------------------VC------------------------- 38
Cavia porcellus ADIC            24 ---------------------------------VC------------------------- 25
Oryctolagus cuniculus ADIC      66 ---------------------------------VC------------------------- 67
Equus caballus ADIC             58 ---------------------------------VC------------------------- 59
Canis lupus familiaris ADIC     44 ---------------------------------VC------------------------- 45
Myotis lucifugus ADIC           25 ---------------------------------VC------------------------- 26
Loxodonta africana ADIC         40 ---------------------------------TC------------------------- 41
Homo sapiens ADID               58 -------------------------VLLPLPKGAC------------------------- 67
Pan troglodytes ADID            58 -------------------------VLLPLPKGAC------------------------- 67
Pongo abelii ADID               27 -------------------------VLLPLPKGAC------------------------- 36
Nomascus leucogenys ADID        57 -------------------------VLLPLPKGAC------------------------- 66
Macaca mulatta ADID             56 -------------------------VLLPLPKGAC------------------------- 65
Otolemur garnettii ADID         27 -------------------------VGLPLPKGAC------------------------- 36
Mus musculus Adid               40 ------------------------PALVPPPKGTC------------------------- 50
Rattus norvegicus Adid          27 -------------------------ALVPPPKETC------------------------- 36
Cavia porcellus ADID            69 -------------------------VPLLLPKGAC------------------------- 78
Oryctolagus cuniculus ADID      37 -------------------------VLIPAPKGAC------------------------- 46
Bos taurus ADID                 25 ----------------------------PLPKGAC------------------------- 31
Equus caballus ADID             87 ----------------------------TLPKEGC------------------------- 93
Canis lupus familiaris ADID     36 -------------------------VLLPLPKGAC------------------------- 45
Felis catus ADID                27 -------------------------VVVPLPKGAC------------------------- 36
Myotis lucifugus ADID           36 -------------------------VQLPLPKGVC------------------------- 45
Dasypus novemcinctus ADID       28 -------------------------VLLLPPKGAC------------------------- 37
Loxodonta africana ADID         27 -------------------------VLLSPPKERC------------------------- 36
Homo sapiens ADIE               22 --------------------------NKI-PSL-C------------------------- 28
Pan troglodytes ADIE            22 --------------------------NKI-PSL-C------------------------- 28
Pongo abelii ADIE               22 --------------------------NKI-PSL-C------------------------- 28
Papio hamadryas ADIE            22 --------------------------NKI-PSL-C------------------------- 28
Callithrix jacchus ADIE         22 --------------------------NKI-PSL-C------------------------- 28
Otolemur garnettii ADIE         22 --------------------------NKI-PSL-C------------------------- 28
Mus musculus Adie               22 --------------------------NKI-PSL-C------------------------- 28
Rattus norvegicus Adie          22 --------------------------NKI-PSL-C------------------------- 28
Homo sapiens ADIF1              22 ---------------------------------TC------------------------- 23
Homo sapiens ADIF2              22 ---------------------------------TC------------------------- 23
Pan troglodytes ADIF1           22 ---------------------------------TC------------------------- 23
Pongo abelii ADIF1              22 ---------------------------------TC------------------------- 23
Nomascus leucogenys ADIF1       22 ---------------------------------TC------------------------- 23
Macaca mulatta ADIF1            22 ---------------------------------TC------------------------- 23
Papio hamadryas ADIF1           22 ---------------------------------TC------------------------- 23
```

```
Microcebus murinus ADIF1        22 ---------------------------------TC-------------------------  23
Otolemur garnettii ADIF1        22 ---------------------------------TC-------------------------  23
Mus musculus Adif1              51 ---------------------------------TC-------------------------  52
Rattus norvegicus Adif1         38 ---------------------------------TC-------------------------  39
Cavia porcellus ADIF1           22 ---------------------------------TC-------------------------  23
Oryctolagus cuniculus ADIF1     22 ---------------------------------TC-------------------------  23
Ochotona princeps ADIF1         22 ---------------------------------TC-------------------------  23
Bos taurus ADIF1                22 ---------------------------------TC-------------------------  23
Equus caballus ADIF1            22 ---------------------------------TC-------------------------  23
Canis lupus familiaris ADIF1    22 ---------------------------------TC-------------------------  23
Felis catus ADIF1               22 ---------------------------------TC-------------------------  23
Dasypus novemcinctus ADIF1      23 ---------------------------------TC-------------------------  24
Loxodonta africana ADIF1        22 ---------------------------------MC-------------------------  23
Homo sapiens ADIG               81 ---------------------------NYSPRYIC-------------------------  88
Pan troglodytes ADIG            81 ---------------------------NYSPRYIC-------------------------  88
Pongo abelii ADIG               81 ---------------------------NYSPRYIC-------------------------  88
Callithrix jacchus ADIG         67 ---------------------------NYSPRYIC-------------------------  74
Otolemur garnettii ADIG         27 ---------------------------NYSPRYIC-------------------------  34
Mus musculus Adig               69 ---------------------------SYSPRYIC-------------------------  76
Rattus norvegicus Adig          70 ---------------------------SYSPRYIC-------------------------  77
Cavia porcellus ADIG            27 ---------------------------SYSPRYIC-------------------------  34
Oryctolagus cuniculus ADIG      27 ---------------------------SYTPRYIC-------------------------  34
Tursiops truncatus ADIG         79 ---------------------------SYSPRYIC-------------------------  86
Bos taurus ADIG                 27 ---------------------------SYSPRYIC-------------------------  34
Equus caballus ADIG             68 ---------------------------SYSPRYIC-------------------------  75
Canis lupus familiaris ADIG     68 ---------------------------SYSPRYIC-------------------------  75
Myotis lucifugus ADIG           27 ---------------------------SYAPRYIC-------------------------  34
Homo sapiens ADIH               27 -------------------------FRKGSPQLVC-------------------------  36
Pan troglodytes ADIH            27 -------------------------FRKGSPQLIC-------------------------  36
Pongo abelii ADIH               27 -------------------------FRKSSPQLVC-------------------------  36
Nomascus leucogenys ADIH        27 -------------------------FRKGSPQLVC-------------------------  36
Macaca mulatta ADIH             27 -------------------------FRKGSPQLVC-------------------------  36
Papio hamadryas ADIH            27 -------------------------FRKGSPQLVC-------------------------  36
Mus musculus Adih               34 -------------------------FQKGGPQLVC-------------------------  43
Rattus norvegicus Adih          71 -------------------------FQKGGPQLVC-------------------------  80
Dipodomys ordii ADIH            27 -------------------------FQKGAPQLIC-------------------------  36
Cavia porcellus ADIH            27 -------------------------FQKGSPQLVC-------------------------  36
Bos taurus ADIH                 44 -------------------------FQKGSPQLIC-------------------------  53
Equus caballus ADIH             27 -------------------------FQKGSPQLVC-------------------------  36
Canis lupus familiaris ADIH     27 -------------------------FQKGSPQLVC-------------------------  36
Pteropus vampyrus ADIH          27 -------------------------FQKGSSQLVC-------------------------  36
Loxodonta africana ADIH         27 -------------------------IKKGPPQLIC-------------------------  36
Homo sapiens ADII               27 --------------------------------GTCRMIC-DP-YTAA-PGGEPPGAKAQP  51
Pongo abelii ADII               27 --------------------------------GTCRMIC-DP-YTAA-PGGGPAGAKAQP  51
Macaca mulatta ADII             27 --------------------------------GTCRMIC-DP-YTAA-PGGGPAGAKAQP  51
Mus musculus Adii               27 --------------------------------GTCRMIC-DP-YSVA-PAGGPAGAKAPP  51
Rattus norvegicus Adii          27 --------------------------------GTCRMIC-DP-YTVS-PAGGPAGAKAPP  51
Bos taurus ADII                 27 --------------------------------GTCRMIC-DP-YTAA-PGAGPAGAKAPP  51
Canis lupus familiaris ADII     27 --------------------------------GTCRMIC-DP-YGAA-PAGGPAGAKAPP  51
Dasypus novemcinctus ADII       27 --------------------------------GTCRMIC-DP-YSAS-PGGGPAGAKAPP  51
Homo sapiens ADIJ               26 --------------------------------GTCRMVC-DP-YG---------GTKA--  40
Pan troglodytes ADIJ            26 --------------------------------GTCRMVC-DP-YG---------GTKA--  40
Papio hamadryas ADIJ            26 --------------------------------GTCRMVC-DP-YG---------GTKA--  40
Mus musculus Adij               26 --------------------------------GTCRMVC-DP-YG---------GTKA--  40
Rattus norvegicus Adij          26 --------------------------------GTCRMVC-DP-YG---------GTKA--  40
Oryctolagus cuniculus ADIJ      26 --------------------------------GTCRMVC-DP-YG---------GTKA--  40
Bos taurus ADIJ                 26 --------------------------------GTCRMVC-DP-YG---------GTKA--  40
Canis lupus familiaris ADIJ     26 --------------------------------GTCRMVC-DP-YG---------GTKA--  40
Loxodonta africana ADIJ         26 --------------------------------GTCRMVC-DP-YG---------GTKA--  40
Homo sapiens ADIK               26 --------------------------------GRCRMVC-DP-H----------------  35
Pan troglodytes ADIK            26 --------------------------------GRCRMVC-DP-H----------------  35
Gorilla gorilla ADIK            26 --------------------------------GRCRMVC-DP-H----------------  35
Papio hamadryas ADIK            26 --------------------------------GRCRMVC-DP-H----------------  35
Callithrix jacchus ADIK         26 --------------------------------GRCRMVC-DP-H----------------  35
Rattus norvegicus Adik          26 --------------------------------GRCRMVC-DP-H----------------  35
Cavia porcellus ADIK            26 --------------------------------GRCRMVC-DP-H----------------  35
Equus caballus ADIK             26 --------------------------------GRCRMVC-DP-H----------------  35
Canis lupus familiaris ADIK     26 --------------------------------GRCRMVC-DP-H----------------  35
Dasypus novemcinctus ADIK       26 --------------------------------GRCRMVC-DP-H----------------  35
Loxodonta africana ADIK         26 --------------------------------GRCRMVC-DP-H----------------  35
Mus musculus Adil               26 --------------------------------GTCRMVC-DP-YPARGPGA---GARSD-  47
Rattus norvegicus Adil          26 --------------------------------GTCRMVC-DP-YPARGPGA---GARSD-  47
Homo sapiens ADIM              146 -----------------------------LP-PDCSK-CC---H---------------- 155
Pan troglodytes ADIM           146 -----------------------------LP-PDCSK-CC---H---------------- 155
```

```
Pongo abelii ADIM                 102 -----------------------------LP-PDCSK-CC---H----------------  111
Nomascus leucogenys ADIM          146 -----------------------------LP-PDCSK-CC---H----------------  155
Papio hamadryas ADIM              146 -----------------------------LP-PDCSK-CC---H----------------  155
Callithrix jacchus ADIM           136 -----------------------------MA-PDCSK-CC---H----------------  145
Mus musculus Adim                  35 -----------------------------LP-PDCSK-CC---H----------------   44
Rattus norvegicus Adim            109 -----------------------------LP-PDCSK-CC---H----------------  118
Cavia porcellus ADIM              102 -----------------------------LP-PDCSK-CC---H----------------  111
Bos taurus ADIM                    35 -----------------------------LP-PDCSK-CC---H----------------   44
Equus caballus ADIM                35 -----------------------------LP-PDCSK-CC---H----------------   44
Canis lupus familiaris ADIM        96 -----------------------------LP-PDCSK-CC---H----------------  105
Homo sapiens ADIN                   - ------------------------------------------------------------    -
Pan troglodytes ADIN                - ------------------------------------------------------------    -
Pongo abelii ADIN                   - ------------------------------------------------------------    -
Nomascus leucogenys ADIN            - ------------------------------------------------------------    -
Macaca mulatta ADIN                 - ------------------------------------------------------------    -
Callithrix jacchus ADIN             - ------------------------------------------------------------    -
Mus musculus Adin                   - ------------------------------------------------------------    -
Cavia porcellus ADIN                - ------------------------------------------------------------    -
Tursiops truncatus ADIN             - ------------------------------------------------------------    -
Canis lupus familiaris ADIN         - ------------------------------------------------------------    -
Dasypus novemcinctus ADIN           - ------------------------------------------------------------    -
Loxodonta africana ADIN             - ------------------------------------------------------------    -
Homo sapiens ADIO                  22 ------------------------------PRRPCVH-CCRPAWP---PG--P-YARVS-   43
Pan troglodytes ADIO               22 ------------------------------PRRPCVH-CCRPAWP---PG--P-YARVS-   43
Macaca mulatta ADIO                22 ------------------------------PRRPCVH-CCHPAWP---PG--P-YARVS-   43
Bos taurus ADIO                    21 ----------------------------GLPRRPCVQ-CCHPAWPPAAPG--P-GAHVS-   47
Myotis lucifugus ADIO              21 ----------------------------GPPHRPCVH-CCHPAWPPAAPG--S-YTPEG-   47
Homo sapiens ADIP                 137 GTEELPSPPDHAERAEEQHEKYRPSQDQGLPASRCLR-CCDPGTS---MY--P-ATAV--  187
Otolemur garnettii ADIP            39 GTKEPPTPPDHTDRAEEKHEKYRPSQGEELPTSQCFR-CCDPAAP---LY--P-VAPV--   89
Mus musculus Adip                  39 ETEELPSPLDPVTRPEETREKYSPRQGEDLPTSRCYR-CCDPSTP---VY--Q-TIPP--   89
Cavia porcellus ADIP               39 GTQKPPPLMDHAERDEDKHEKYSTSLGEELPASQCFR-CCDPGTP---VY--Q-GIPV--   89
Bos taurus ADIP                    64 GTREPP--MDHAERDEEEHEKYGPRQDEEAPASRCLR-CCDPGTP---VY--Q-AIPV--  112
Equus caballus ADIP               39 GTREPP--LDPAERTEEKHEKYNPKQGEEPTASRCFR-CCDPGTP---VY--Q-AIPV--   87
Loxodonta africana ADIP            91 GTKEPPPPRGHPERAEEEHEKYSPRQAEEPPASRCFR-CCDPSAP---VY--Q-PIPV--  141
Homo sapiens ADIQ                  52 -------------------------VAS----G-CQR-CCDSEDP---LD--P--AHVS-   72
Pan troglodytes ADIQ               52 -------------------------VAS----G-CQR-CCDSEDP---LD--P--AHVS-   72
Pongo abelii ADIQ                  52 -------------------------VAS----G-CQR-CCDSEDP---LD--P--AHVS-   72
Nomascus leucogenys ADIQ           52 -------------------------VAS----S-CQR-CCDSEDP---LD--P--AHVS-   72
Macaca mulatta ADIQ                52 -------------------------VAS----G-CQR-CCDSEDP---LD--P--AHVS-   72
Mus musculus Adiq                  36 -------------------------VASHLPKG-CRR-CCDPEDL---MS--S--DDTV-   60
Rattus norvegicus Adiq             36 -------------------------VASHLPKN-CQR-CCDPEDP---LS--P--ADTV-   60
Homo sapiens ADIR                   - ------------------------------------------------------------    -
Pan troglodytes ADIR                - ------------------------------------------------------------    -
Pongo abelii ADIR                   - ------------------------------------------------------------    -
Nomascus leucogenys ADIR            - ------------------------------------------------------------    -
Macaca mulatta ADIR                 - ------------------------------------------------------------    -
Papio hamadryas ADIR                - ------------------------------------------------------------    -
Callithrix jacchus ADIR             - ------------------------------------------------------------    -
Tarsius syrichta ADIR               - ------------------------------------------------------------    -
Otolemur garnettii ADIR             - ------------------------------------------------------------    -
Tupaia belangeri ADIR               - ------------------------------------------------------------    -
Mus musculus Adir                   - ------------------------------------------------------------    -
Rattus norvegicus Adir              - ------------------------------------------------------------    -
Cavia porcellus ADIR                - ------------------------------------------------------------    -
Oryctolagus cuniculus ADIR          - ------------------------------------------------------------    -
Tursiops truncatus ADIR             - ------------------------------------------------------------    -
Bos taurus ADIR                     - ------------------------------------------------------------    -
Equus caballus ADIR                 - ------------------------------------------------------------    -
Canis lupus familiaris ADIR         - ------------------------------------------------------------    -
Myotis lucifugus ADIR               - ------------------------------------------------------------    -
Pteropus vampyrus ADIR              - ------------------------------------------------------------    -
Erinaceus europaeus ADIR            - ------------------------------------------------------------    -
Dasypus novemcinctus ADIR           - ------------------------------------------------------------    -
Choloepus hoffmanni ADIR            - ------------------------------------------------------------    -
Loxodonta africana ADIR             - ------------------------------------------------------------    -
```

```
Homo sapiens ADIA                32 ------------------TGP-PAIPGIP----------------------------GIP  44
                                              250       260       270       280       290       300

Homo sapiens ADIA                32 ------------------TGP-PAIPGIP----------------------------GIP  44
Papio hamadryas ADIA             32 ------------------TGP-PAIPGTP----------------------------GIP  44
Mus musculus Adia                59 ------------------TGP-PGIPGIP----------------------------GVP  71
Rattus norvegicus Adia           30 ------------------TGS-PGIPGVP----------------------------GIP  42
Cavia porcellus ADIA             30 ------------------VGH-PAIPGIP----------------------------GIP  42
Ochotona princeps ADIA           30 ------------------SGH-PAIPGTP----------------------------GIP  42
Bos taurus ADIA                  27 ------------------I-R-PSIPGIP----------------------------GIP  38
Equus caballus ADIA              31 ------------------PAS-QGIPGIP----------------------------GIP  43
Canis lupus familiaris ADIA      29 ------------------TGH-PAIPGIP----------------------------GIP  41
Felis catus ADIA                 33 ------------------TGH-PAIPGIP----------------------------GIP  45
Myotis lucifugus ADIA            30 ------------------TGP-AAIPGIP----------------------------GIP  42
Dasypus novemcinctus ADIA        27 ------------------TGP-ATIPGTP----------------------------GIP  39
Loxodonta africana ADIA          30 ------------------SGP-RTIPGIP----------------------------GIP  42
Homo sapiens ADIB                33 ------------------Y-------GIP----------------------------GMP  39
Pan troglodytes ADIB             33 ------------------Y-------GIP----------------------------GMP  39
Pongo abelii ADIB                33 ------------------Y-------GIP----------------------------GMP  39
Nomascus leucogenys ADIB         34 ------------------Y-------GIP----------------------------GMP  40
Callithrix jacchus ADIB          32 ------------------Y-------GIP----------------------------GMP  38
Otolemur garnettii ADIB          31 ------------------Y-------GIP----------------------------GMP  37
Mus musculus Adib                64 ------------------Y-------GIP----------------------------GMP  70
Rattus norvegicus Adib           56 ------------------Y-------GIP----------------------------GMP  62
Cavia porcellus ADIB             80 ------------------Y-------GIP----------------------------GMP  86
Oryctolagus cuniculus ADIB       86 ------------------Y-------GIP----------------------------GMP  92
Bos taurus ADIB                  31 ------------------Y-------GIP----------------------------GMP  37
Equus caballus ADIB              51 ------------------Y-------GIP----------------------------GMP  57
Canis lupus familiaris ADIB      33 ------------------Y-------GIP----------------------------GMP  39
Myotis lucifugus ADIB            33 ------------------Y-------GIP----------------------------GMP  39
Loxodonta africana ADIB          31 ------------------Y-------GIP----------------------------GMP  37
Homo sapiens ADIC                27 ------------------RAP-D---GKK----------------------------GEA  36
Macaca mulatta ADIC              27 ------------------RAP-D---GKN----------------------------GVA  36
Papio hamadryas ADIC             27 ------------------RAP-D---GKN----------------------------GVA  36
Callithrix jacchus ADIC          27 ------------------QAP-N---GKD----------------------------GVA  36
Mus musculus Adic                27 ------------------RAP-N---GKD----------------------------GAP  36
Rattus norvegicus Adic           39 ------------------RAP-N---GKD----------------------------GVA  48
Cavia porcellus ADIC             26 ------------------RAP-N---GKD----------------------------GAA  35
Oryctolagus cuniculus ADIC       68 ------------------RAP-D---GKD----------------------------GAA  77
Equus caballus ADIC              60 ------------------RAL-N---GKD----------------------------GAP  69
Canis lupus familiaris ADIC      46 ------------------RAL-D---GRD----------------------------GAA  55
Myotis lucifugus ADIC            27 ------------------RAP-D---GRD----------------------------GVA  36
Loxodonta africana ADIC          42 ------------------RAP-D---GTD----------------------------GTP  51
Homo sapiens ADID                68 ------------------TG---WMAGIP----------------------------GHP  78
Pan troglodytes ADID             68 ------------------TG---WMAGIP----------------------------GHP  78
Pongo abelii ADID                37 ------------------TG---WMAGIP----------------------------GHP  47
Nomascus leucogenys ADID         67 ------------------TG---WMAGIP----------------------------GHP  77
Macaca mulatta ADID              66 ------------------TG---WMAGIP----------------------------GHP  76
Otolemur garnettii ADID          37 ------------------MG---WMAGIP----------------------------GHP  47
Mus musculus Adid                51 ------------------AG---WMAGIP----------------------------GHP  61
Rattus norvegicus Adid           37 ------------------AG---WMAGIP----------------------------GYP  47
Cavia porcellus ADID             79 ------------------AG---WMAGIP----------------------------GHP  89
Oryctolagus cuniculus ADID       47 ------------------AG---WIAGIP----------------------------GHP  57
Bos taurus ADID                  32 ------------------AG---WMAGIP----------------------------GHP  42
Equus caballus ADID              94 ------------------AG---WMAGIP----------------------------GHP 104
Canis lupus familiaris ADID      46 ------------------PG---WMAGIP----------------------------GHP  56
Felis catus ADID                 37 ------------------TG---WMAGIP----------------------------GHP  47
Myotis lucifugus ADID            46 ------------------TG---WVAGIP----------------------------GHP  56
Dasypus novemcinctus ADID        38 ------------------V-----IAGIP----------------------------GLP  46
Loxodonta africana ADID          37 ------------------AG---WMAGIP----------------------------GHP  47
Homo sapiens ADIE                29 -------------------------PGHP----------------------------GLP  35
Pan troglodytes ADIE             29 -------------------------PGHP----------------------------GLP  35
Pongo abelii ADIE                29 -------------------------PGHP----------------------------GLP  35
Papio hamadryas ADIE             29 -------------------------PGHP----------------------------GLP  35
Callithrix jacchus ADIE          29 -------------------------PGHP----------------------------GLP  35
Otolemur garnettii ADIE          29 -------------------------PGHP----------------------------GLP  35
Mus musculus Adie                29 -------------------------PGQP----------------------------GLP  35
Rattus norvegicus Adie           29 -------------------------PGQP----------------------------GLP  35
Homo sapiens ADIF1               24 ------------------------RQGHP----------------------------GIP  31
Homo sapiens ADIF2               24 ------------------------RQGHP----------------------------GIP  31
Pan troglodytes ADIF1            24 ------------------------RQGHP----------------------------GIP  31
Pongo abelii ADIF1               24 ------------------------RQGHP----------------------------GIP  31
Nomascus leucogenys ADIF1        24 ------------------------RQGHP----------------------------GIP  31
Macaca mulatta ADIF1             24 ------------------------RQGHP----------------------------GIP  31
Papio hamadryas ADIF1            24 ------------------------RQGHP----------------------------GIP  31
```

```
Microcebus murinus ADIF1          24 ------------------------RQGHP----------------------------GVP  31
Otolemur garnettii ADIF1          24 ------------------------RQGPS----------------------------GIP  31
Mus musculus Adif1                53 ------------------------RQGHS----------------------------GIP  60
Rattus norvegicus Adif1           40 ------------------------RQGHS----------------------------GIP  47
Cavia porcellus ADIF1             24 ------------------------RQGHS----------------------------GIP  31
Oryctolagus cuniculus ADIF1       24 ------------------------RQGHP----------------------------GIP  31
Ochotona princeps ADIF1           24 ------------------------RQGHP----------------------------GMP  31
Bos taurus ADIF1                  24 ------------------------KQGHP----------------------------GIP  31
Equus caballus ADIF1              24 ------------------------RQGHP----------------------------GIP  31
Canis lupus familiaris ADIF1      24 ------------------------RQGHP----------------------------GIP  31
Felis catus ADIF1                 24 ------------------------RQGHP----------------------------GVP  31
Dasypus novemcinctus ADIF1        25 ------------------------RQGHP----------------------------GIP  32
Loxodonta africana ADIF1          24 ------------------------RQGHS----------------------------GIP  31
Homo sapiens ADIG                 89 ------------------S-----IPGLP----------------------------GPP  97
Pan troglodytes ADIG              89 ------------------S-----IPGLP----------------------------GPP  97
Pongo abelii ADIG                 89 ------------------S-----IPGLP----------------------------GPP  97
Callithrix jacchus ADIG           75 ------------------S-----IPGLP----------------------------GPP  83
Otolemur garnettii ADIG           35 ------------------S-----IPGLP----------------------------GPP  43
Mus musculus Adig                 77 ------------------S-----IPGLP----------------------------GPP  85
Rattus norvegicus Adig            78 ------------------S-----IPGLP----------------------------GPP  86
Cavia porcellus ADIG              35 ------------------S-----IPGLP----------------------------GPP  43
Oryctolagus cuniculus ADIG        35 ------------------S-----IPGLP----------------------------GPP  43
Tursiops truncatus ADIG           87 ------------------S-----IPGLP----------------------------GPP  95
Bos taurus ADIG                   35 ------------------S-----IPGLP----------------------------GPP  43
Equus caballus ADIG               76 ------------------S-----IPGLP----------------------------GPP  84
Canis lupus familiaris ADIG       76 ------------------S-----IPGLP----------------------------GPP  84
Myotis lucifugus ADIG             35 ------------------S-----IPGLP----------------------------GPP  43
Homo sapiens ADIH                 37 ------------------S-----LPGPQ----------------------------GPP  45
Pan troglodytes ADIH              37 ------------------S-----LPGPQ----------------------------GPP  45
Pongo abelii ADIH                 37 ------------------S-----LPGPQ----------------------------GPP  45
Nomascus leucogenys ADIH          37 ------------------S-----LPGPQ----------------------------GPP  45
Macaca mulatta ADIH               37 ------------------S-----LPGPQ----------------------------GPP  45
Papio hamadryas ADIH              37 ------------------S-----LPGPQ----------------------------GPP  45
Mus musculus Adih                 44 ------------------S-----LPGPQ----------------------------GPP  52
Rattus norvegicus Adih            81 ------------------S-----LPGPQ----------------------------GPP  89
Dipodomys ordii ADIH              37 ------------------S-----LPGPQ----------------------------GPP  45
Cavia porcellus ADIH              37 ------------------S-----LPGPQ----------------------------GPP  45
Bos taurus ADIH                   54 ------------------S-----MPGPQ----------------------------GPP  62
Equus caballus ADIH               37 ------------------S-----LPGPQ----------------------------GPP  45
Canis lupus familiaris ADIH       37 ------------------S-----LPGPQ----------------------------GPP  45
Pteropus vampyrus ADIH            37 ------------------S-----LPGPQ----------------------------GPP  45
Loxodonta africana ADIH           37 ------------------S-----MPGPQ----------------------------GPP  45
Homo sapiens ADII                 52 PGPSTAA-LEVMQDLSANPPP-PFIQGPK-------------------GDPGRGPKPGPR  90
Pongo abelii ADII                 52 PGPSTAA-LEVMQDLSANPPP-PFIQGPK-------------------GDPGRGPKPGPR  90
Macaca mulatta ADII               52 PGPSTAA-LEVMQDLSANPPP-PFIQGPK-------------------GDPGRGPKPGPR  90
Mus musculus Adii                 52 PGPSTAA-LEVMQDLSANPPP-PFIQGPK-------------------GDPGRGPKPGPR  90
Rattus norvegicus Adii            52 PGPSTAA-LEVMQDLSANPPP-PFIQGPK-------------------GDPGRGPKPGPR  90
Bos taurus ADII                   52 PGPSTAA-LEVMQDLSINPPP-PFIQGPK-------------------GDPGRGPKPGPR  90
Canis lupus familiaris ADII       52 PGPSTAA-LEVMQDLSANPPP-PFIQGPK-------------------GDPGRGPKPGPR  90
Dasypus novemcinctus ADII         52 PGPSTAA-LEVMQDLSANPPP-PFIQGPK-------------------GDPGRGPKPGPR  90
Homo sapiens ADIJ                 41 --PSTAATPDRGLMQS-L--P-TFIQGPK-------------------GEAGRPGKAGPR  75
Pan troglodytes ADIJ              41 --PSTAATPDRGLMQS-L--P-TFIQGPK-------------------GEAGRPGKAGPR  75
Papio hamadryas ADIJ              41 --PSTAATPDRGLMQS-L--P-TFIQGPK-------------------GEAGRPGKAGPR  75
Mus musculus Adij                 41 --PSTAATPDRGLMQS-L--P-TFIQGPK-------------------GEAGRPGKAGPR  75
Rattus norvegicus Adij            41 --PSTAATPDRGLMQS-L--P-TFIQGPK-------------------GEAGRPGKAGPR  75
Oryctolagus cuniculus ADIJ        41 --PSTAATPDRGLMQS-L--P-TFIQGPK-------------------GEAGRPGKAGPR  75
Bos taurus ADIJ                   41 --PSTAATPDRGLMQS-L--P-TFIQGPK-------------------GEAGRPGKAGPR  75
Canis lupus familiaris ADIJ       41 --PSTAATPDRGLMQS-L--P-TFIQGPK-------------------GEAGRPGKAGPR  75
Loxodonta africana ADIJ           41 --PSTAATPDRGLMQS-L--P-TFIQGPK-------------------GEAGRPGKAGPR  75
Homo sapiens ADIK                 36 --------------------------GPRGPGPDGAPASVPPFPPGAKGEVGRRGKAGLR  69
Pan troglodytes ADIK              36 --------------------------GPRGPGPDGAPASVPPFPPGAKGEVGRRGKAGLR  69
Gorilla gorilla ADIK              36 --------------------------GPRGPGPDGAPASVPPFPPGAKGEVGRRGKAGLR  69
Papio hamadryas ADIK              36 --------------------------GPRGPGPDGAPASVSPFPPGAKGEVGRRGKAGLR  69
Callithrix jacchus ADIK           36 --------------------------GPRGQGPDGAPASVPPFPPGAKGEVGRRGKAGLR  69
Rattus norvegicus Adik            36 --------------------------ASRGQGSDGAPSSVPPFPPGAKGEVGRRGKAGLR  69
Cavia porcellus ADIK              36 --------------------------GPRGPGPDGAPASVPPFPPGAKGEVGRRGKAGLR  69
Equus caballus ADIK               36 --------------------------GPRGPGTDGAPASVPPYPPGTKGEMGRRGKAGLR  69
Canis lupus familiaris ADIK       36 --------------------------GPRGPGPDGAPASVPPFPPGAKGEVGRRGKAGLR  69
Dasypus novemcinctus ADIK         36 --------------------------GPRGPDPEGAPASVPPFPPGAKGETGRRGKAGLR  69
Loxodonta africana ADIK           36 --------------------------GPRGPGPDGAPSSVPPFPPGAKGEVGRRGKAGLR  69
Mus musculus Adil                 48 GG-------DALSEQSGAPPPSTLVQGPQ-------------------GKPGRTGKPGPP  81
Rattus norvegicus Adil            48 GG-------DALSEQSGAPPPSTLVQGPQ-------------------GKPGRTGKPGPP  81
Homo sapiens ADIM                156 --------------------------GDY------------------------------- 158
Pan troglodytes ADIM             156 --------------------------GDY------------------------------- 158
```

```
Pongo abelii ADIM            112 --------------------------GDY------------------------------- 114
Nomascus leucogenys ADIM     156 --------------------------GDY------------------------------- 158
Papio hamadryas ADIM         156 --------------------------GDY------------------------------- 158
Callithrix jacchus ADIM      146 --------------------------GDY------------------------------- 148
Mus musculus Adim             45 --------------------------GDY-------------------------------  47
Rattus norvegicus Adim       119 --------------------------GDY------------------------------- 121
Cavia porcellus ADIM         112 --------------------------GDY------------------------------- 114
Bos taurus ADIM               45 --------------------------GDY-------------------------------  47
Equus caballus ADIM           45 --------------------------GDY-------------------------------  47
Canis lupus familiaris ADIM  106 --------------------------GEY------------------------------- 108
Homo sapiens ADIN              - ------------------------------------------------------------   -
Pan troglodytes ADIN           - ------------------------------------------------------------   -
Pongo abelii ADIN              - ------------------------------------------------------------   -
Nomascus leucogenys ADIN       - ------------------------------------------------------------   -
Macaca mulatta ADIN            - ------------------------------------------------------------   -
Callithrix jacchus ADIN        - ------------------------------------------------------------   -
Mus musculus Adin              - ------------------------------------------------------------   -
Cavia porcellus ADIN           - ------------------------------------------------------------   -
Tursiops truncatus ADIN        - ------------------------------------------------------------   -
Canis lupus familiaris ADIN    - ------------------------------------------------------------   -
Dasypus novemcinctus ADIN      - ------------------------------------------------------------   -
Loxodonta africana ADIN        - ------------------------------------------------------------   -
Homo sapiens ADIO             44 DRDLWRGDLWRGLPRVRPTIDIEILK----------------------------------  69
Pan troglodytes ADIO          44 DRDLWRGDLWRGLPRVRPTIDIEILK----------------------------------  69
Macaca mulatta ADIO           44 D-----GDPWRSLPRVRPTIDIEILK----------------------------------  64
Bos taurus ADIO               48 D-----GDAWAGLPRLRPTIDISILK----------------------------------  68
Myotis lucifugus ADIO         48 E-----GEKWVQLPHVRPTIDISILK----------------------------------  68
Homo sapiens ADIP            188 -----------------PQINITILK---------------------------------- 196
Otolemur garnettii ADIP       90 -----------------PQINITILK----------------------------------  98
Mus musculus Adip             90 -----------------PQINITILK----------------------------------  98
Cavia porcellus ADIP          90 -----------------PQINITILK----------------------------------  98
Bos taurus ADIP              113 -----------------PQINITILK---------------------------------- 121
Equus caballus ADIP           88 -----------------PQINITILK----------------------------------  96
Loxodonta africana ADIP      142 -----------------PQINITILK---------------------------------- 150
Homo sapiens ADIQ             73 SASS-SGRPHA-LPEIRPYINITILK----------------------------------  96
Pan troglodytes ADIQ          73 SASS-SGRPHA-LPEIRPYINITILK----------------------------------  96
Pongo abelii ADIQ             73 SASS-SGPPYA-LPEIRPYINITILK----------------------------------  96
Nomascus leucogenys ADIQ      73 SASS-SGPPHA-LPEIRPYINITILK----------------------------------  96
Macaca mulatta ADIQ           73 SASS-SGLPHA-LPEIRPYINITILK----------------------------------  96
Mus musculus Adiq             61 QAP---VSPYV-LPEVRPYINITILK----------------------------------  82
Rattus norvegicus Adiq        61 NA----VPPYV-LPEVRPYINITILK----------------------------------  81
Homo sapiens ADIR              - ------------------------------------------------------------   -
Pan troglodytes ADIR           - ------------------------------------------------------------   -
Pongo abelii ADIR              - ------------------------------------------------------------   -
Nomascus leucogenys ADIR       - ------------------------------------------------------------   -
Macaca mulatta ADIR            - ------------------------------------------------------------   -
Papio hamadryas ADIR           - ------------------------------------------------------------   -
Callithrix jacchus ADIR        - ------------------------------------------------------------   -
Tarsius syrichta ADIR          - ------------------------------------------------------------   -
Otolemur garnettii ADIR        - ------------------------------------------------------------   -
Tupaia belangeri ADIR          - ------------------------------------------------------------   -
Mus musculus Adir              - ------------------------------------------------------------   -
Rattus norvegicus Adir         - ------------------------------------------------------------   -
Cavia porcellus ADIR           - ------------------------------------------------------------   -
Oryctolagus cuniculus ADIR     - ------------------------------------------------------------   -
Tursiops truncatus ADIR        - ------------------------------------------------------------   -
Bos taurus ADIR                - ------------------------------------------------------------   -
Equus caballus ADIR            - ------------------------------------------------------------   -
Canis lupus familiaris ADIR    - ------------------------------------------------------------   -
Myotis lucifugus ADIR          - ------------------------------------------------------------   -
Pteropus vampyrus ADIR         - ------------------------------------------------------------   -
Erinaceus europaeus ADIR       - ------------------------------------------------------------   -
Dasypus novemcinctus ADIR      - ------------------------------------------------------------   -
Choloepus hoffmanni ADIR       - ------------------------------------------------------------   -
Loxodonta africana ADIR        - ------------------------------------------------------------   -
```

```
Homo sapiens ADIA               45 GTP------------GPDGQP---GTPGIKGEK---G-----------------------   63
                                           310       320       330       340       350       360

Homo sapiens ADIA               45 GTP------------GPDGQP---GTPGIKGEK---G-----------------------   63
Papio hamadryas ADIA            45 GTP------------GSDGQP---GTPGIKGEK---G-----------------------   63
Mus musculus Adia               72 GVP------------GSDGQP---GTPGIKGEK---G-----------------------   90
Rattus norvegicus Adia          43 GVP------------GSDGKP---GTPGIKGEK---G-----------------------   61
Cavia porcellus ADIA            43 GVP------------GSDGQP---GTPGTKGEK---G-----------------------   61
Ochotona princeps ADIA          43 GLP------------GSDGNP---GTPGVKGEK---G-----------------------   61
Bos taurus ADIA                 39 GKP------------GSDGKP---GTPGTKGEK---G-----------------------   57
Equus caballus ADIA             44 GSP------------GTDGKP---GTPGTKGEK---G-----------------------   62
Canis lupus familiaris ADIA     42 GAP------------GTDGTP---GTPGTKGEK---G-----------------------   60
Felis catus ADIA                46 GAP------------GSPGTP---GTPGIKGEK---G-----------------------   64
Myotis lucifugus ADIA           43 GIP------------GSDGTP---GTPGVKGAK---G-----------------------   61
Dasypus novemcinctus ADIA       40 GRP------------GSDGQP---GTPGIKGAK---G-----------------------   58
Loxodonta africana ADIA         43 GPP------------GTNGQP---GTPGIKGEK---G-----------------------   61
Homo sapiens ADIB               40 GLP------------GAPGKDGYDGLPGPKGEP---G-----------------------   61
Pan troglodytes ADIB            40 GLP------------GAPGKDGYDGLPGPKGEP---G-----------------------   61
Pongo abelii ADIB               40 GLP------------GAPGKDGYDGWPGPKGEP---G-----------------------   61
Nomascus leucogenys ADIB        41 GLP------------GAPGKDGYDGLPGPKGEP---G-----------------------   62
Callithrix jacchus ADIB         39 GLP------------GAPGKDGHDGLPGPKGEP---G-----------------------   60
Otolemur garnettii ADIB         38 GLP------------GAPGKDGHDGLPGPKGEP---G-----------------------   59
Mus musculus Adib               71 GMP------------GAPGKDGHDGLQGPKGEP---G-----------------------   92
Rattus norvegicus Adib          63 GLP------------GTPGKDGHDGLQGPKGEP---G-----------------------   84
Cavia porcellus ADIB            87 GLP------------GAPGKDGHDGLRGPKGEP---G-----------------------  108
Oryctolagus cuniculus ADIB      93 GLP------------GTPGKDGHDGLPGPKGEP---G-----------------------  114
Bos taurus ADIB                 38 GLP------------GAPGKDGYDGLPGPKGEP---G-----------------------   59
Equus caballus ADIB             58 GMP------------GTPGKDGHDGLPGPKGEP---G-----------------------   79
Canis lupus familiaris ADIB     40 GLP------------GAPGKDGHDGLPGPKGEP---G-----------------------   61
Myotis lucifugus ADIB           40 GMP------------GAPGKDGHDGLQGPKGEP---G-----------------------   61
Loxodonta africana ADIB         38 GLP------------GAPGKDGHDGLPGPKGEP---G-----------------------   59
Homo sapiens ADIC               37 GRP------------GRRGRP---GLKGEQGEP---G-----------------------   55
Macaca mulatta ADIC             37 GRP------------GRPGRP---GLKGERGEP---G-----------------------   55
Papio hamadryas ADIC            37 GRP------------GRPGRP---GLKGERGEP---G-----------------------   55
Callithrix jacchus ADIC         37 GRP------------GRPGRP---GLKGEQGEP---G-----------------------   55
Mus musculus Adic               37 GNP------------GRPGRP---GLKGERGEP---G-----------------------   55
Rattus norvegicus Adic          49 GIP------------GRPGRP---GLKGERGEP---G-----------------------   67
Cavia porcellus ADIC            36 GKP------------GRPGRP---GLKGERGEP---G-----------------------   54
Oryctolagus cuniculus ADIC      78 GKP------------GRPGRP---GLKGERGAP---G-----------------------   96
Equus caballus ADIC             70 GIP------------GRPGRP---GPKGERGEP---G-----------------------   88
Canis lupus familiaris ADIC     56 GTP------------GRPGRP---GLKGEQGEP---G-----------------------   74
Myotis lucifugus ADIC           37 GKP------------GRPGRP---GLKGEQGEP---G-----------------------   55
Loxodonta africana ADIC         52 GNP------------GRSGRP---GLKGERGEP---G-----------------------   70
Homo sapiens ADID               79 GHNGAP---------GRDGRDGTPGEKGEKGDP---G-----------------------  103
Pan troglodytes ADID            79 GHNGAP---------GRDGRDGTPGEKGEKGDP---G-----------------------  103
Pongo abelii ADID               48 GHNGAP---------GRDGRDGIPGEKGEKGDP---G-----------------------   72
Nomascus leucogenys ADID        78 GHNGAP---------GRDGRDGISGEKGEKGDP---G-----------------------  102
Macaca mulatta ADID             77 GHNGVP---------GRDGRDGTAGEKGEKGDP---G-----------------------  101
Otolemur garnettii ADID         48 GHNGTP---------GRDGRDGTPGEKGEKGDP---G-----------------------   72
Mus musculus Adid               62 GHNGTP---------GRDGRDGTPGEKGEKGDA---G-----------------------   86
Rattus norvegicus Adid          48 GHNGIP---------GRDGRDGTPGEKGEKGDA---G-----------------------   72
Cavia porcellus ADID            90 GHNGIP---------GRDGRDGAPGEKGEKGDA---G-----------------------  114
Oryctolagus cuniculus ADID      58 GHNGTP---------GRDGRDGTPGEKGEKGDA---G-----------------------   82
Bos taurus ADID                 43 GHNGTP---------GRDGRDGTPGEKGEKGDP---G-----------------------   67
Equus caballus ADID            105 GHNGTP---------GRDGRDGTPGEKGEKGDP---G-----------------------  129
Canis lupus familiaris ADID     57 GHNGTP---------GRDGRDGTPGEKGEKGDP---G-----------------------   81
Felis catus ADID                48 GHNGTP---------GRDGRDGTPGEKGEKGDP---G-----------------------   72
Myotis lucifugus ADID           57 GHNGTP---------GRDGRDGTPGEKGEKGDA---G-----------------------   81
Dasypus novemcinctus ADID       47 GHNGIP---------GRDGRDGVPGQKGEKGDT---G-----------------------   71
Loxodonta africana ADID         48 GHNGTP---------GRDGRDGTPGEKGEKGDP---G-----------------------   72
Homo sapiens ADIE               36 GTPGHHGSQGLPGRDGRDGRDGAPGAPGEKGEGGRPG-----------------------   72
Pan troglodytes ADIE            36 GTPGHHGSQGLPGRDGRDGRDGAPGAPGEKGEGGRPG-----------------------   72
Pongo abelii ADIE               36 GTPGHHGSQGLPGRDGRDGRDGAPGAPGEKGEGGRPG-----------------------   72
Papio hamadryas ADIE            36 GTPGHHGSQGLPGRDGRDGRDGAPGAPGEKGEGGRPG-----------------------   72
Callithrix jacchus ADIE         36 GTPGHHGSQGLPGRDGRDGRDGAPGAPGEKGEGGRPG-----------------------   72
Otolemur garnettii ADIE         36 GTPGHHGSQGLPGRDGRDGRDGAPGAPGEKGEGGQPG-----------------------   72
Mus musculus Adie               36 GTPGHHGSQGLPGRDGRDGRDGAPGAPGEKGEGGRPG-----------------------   72
Rattus norvegicus Adie          36 GTPGHHGSQGLPGRDGRDGRDGAPGAPGEKGEGGRPG-----------------------   72
Homo sapiens ADIF1              32 GNPGHN---------GLPGRDGRDGAKGDKGDA---GEPGRPGSPGKDGTSGEKGERGAD   79
Homo sapiens ADIF2              32 GNPGHN---------GLPGRDGRDGAKGDKGDA---GEPGCPGSPGKDGTSGEKGERGAD   79
Pan troglodytes ADIF1           32 GNPGHN---------GLPGRDGRDGAKGDKGDA---GEPGRPGSPGKDGTSGEKGERGAD   79
Pongo abelii ADIF1              32 GNPGHN---------GLPGRDGRDGAKGDKGDA---GEPGHPGSPGKDGTSGEKGERGAD   79
Nomascus leucogenys ADIF1       32 GNPGHN---------GLPGRDGRDGAKGDKGDA---GEPGRPGSPGKDGTSGEKGERGAD   79
Macaca mulatta ADIF1            32 GNPGHN---------GLPGRDGRDGVKGDKGDA---GEPGRPGSPGKDGTSGEKGERGAD   79
Papio hamadryas ADIF1           32 GNPGHN---------GLPGRDGRDGAKGDKGDA---GEPGRPGSPGKDGTSGEKGERGAD   79
```

***Microcebus murinus* ADIF1**	32	GNPGHN---------GLPGRDGREGAKGDKGDA---GEPGHPGSPGKDGASGDKGERGAD	79
***Otolemur garnettii* ADIF1**	32	GNPGHN---------GLPGRDGRDGAKGDKGDA---GEPGRPGSPGKDGMRGEKGQRGAD	79
***Mus musculus* Adif1**	61	GNPGHN---------GLPGRDGRDGAKGDKGDA---GEPGHPGGPGKDGIRGEKGEPGAD	108
***Rattus norvegicus* Adif1**	48	GTPGHN---------GLPGRDGRDGAKGDKGDT---GEPGHPGGPGKDGIRGEKGEPGAD	95
***Cavia porcellus* ADIF1**	32	GNPGHN---------GLPGRDGRDGAKGDKGDA---GEPGRPGGPGKDGMSGEKGERGAD	79
***Oryctolagus cuniculus* ADIF1**	32	GSPGHN---------GLPGRDGRDGAKGDKGDA---GEPGHPGSPGKEGRSGEKGDRGAD	79
***Ochotona princeps* ADIF1**	32	GSPGHN---------GLPGRDGRDGAKGDKGDT---GEPGHPGDPGKDGRSGEKGERGAD	79
***Bos taurus* ADIF1**	32	GNPGHN---------GLPGRDGRDGAKGDKGDA---GEPGHPGGPGKDGMTGKKGEPGAD	79
***Equus caballus* ADIF1**	32	GNPGHN---------GLPGRDGRDGAKGDKGDA---GEPGRPGGPGKDGVNGEKGERGAD	79
***Canis lupus familiaris* ADIF1**	32	GNPGHN---------GLPGRDGRDGAKGDKGEA---GEPGHPGGPGKDGMNGEKGERGAD	79
***Felis catus* ADIF1**	32	GNPGHN---------GLPGRDGRDGAKGDKGEA---GEPGHPGGPGKDGMNGEKGERGAD	79
***Dasypus novemcinctus* ADIF1**	33	GNPGHN---------GLPGRDGRDGAKGDKGDA---GEPGLPGGPGKDGMKGEKGERGAD	80
***Loxodonta africana* ADIF1**	32	GNPGHN---------GLPGRDGRDGAKGDKGDA---GEPGLPGGPGKDGINGEKGERGAD	79
***Homo sapiens* ADIG**	98	GPPGANGSPGPHGRIGLPGRDGRDGRKGEKGEKGTAG-----------------------	134
***Pan troglodytes* ADIG**	98	GPPGANGSPGPHGRIGLPGRDGRDGRKGEKGEKGTAG-----------------------	134
***Pongo abelii* ADIG**	98	GPPGANGSPGPHGRIGLPGRDGRDGRKGEKGEKGTAG-----------------------	134
***Callithrix jacchus* ADIG**	84	GPPGANGSPGPHGRIGLPGRDGRDGRKGEKGEKGTAG-----------------------	120
***Otolemur garnettii* ADIG**	44	GPPGATGSPGPHGRIGLPGRDGRDGRKGEKGEKGAAG-----------------------	80
***Mus musculus* Adig**	86	GPPGANGSPGPHGRIGLPGRDGRDGRKGEKGEKGTAG-----------------------	122
***Rattus norvegicus* Adig**	87	GPPGANGSPGPHGRIGLPGRDGRDGRKGEKGEKGTAG-----------------------	123
***Cavia porcellus* ADIG**	44	GPPGANGSPGHHGRIGLPGRDGRDGRKGEKGEKGTAG-----------------------	80
***Oryctolagus cuniculus* ADIG**	44	GPPGANGSPGHHGRIGLPGRDGRDGRKGEKGEKGSAG-----------------------	80
***Tursiops truncatus* ADIG**	96	GPPGANGSPGPHGRIGLPGRDGRDGRKGEKGEKGAAG-----------------------	132
***Bos taurus* ADIG**	44	GPPGANGSPGPHGRIGLPGRDGRDGRKGEKGEKGAAG-----------------------	80
***Equus caballus* ADIG**	85	GPPGANGSPGPHGRIGLPGRDGRDGRKGEKGEKGAAG-----------------------	121
***Canis lupus familiaris* ADIG**	85	GPPGANGSPGPHGRIGLPGRDGRDGRKGEKGEKGAAG-----------------------	121
***Myotis lucifugus* ADIG**	44	GPPGASGSPGPHGRIGLPGRDGRDGRKGEKGEKGAAG-----------------------	80
***Homo sapiens* ADIH**	46	GPPGAPGPSGMMGRMGFPGKDGQDGHDGDRGDSGEEG-----------------------	82
***Pan troglodytes* ADIH**	46	GPPGAPGPSGMMGRMGFPGKDGQDGHDGDQGDSGEEG-----------------------	82
***Pongo abelii* ADIH**	46	GPPGAPGPSGMMGRMGFPGKDGQDGHDGDQGDSGEEG-----------------------	82
***Nomascus leucogenys* ADIH**	46	GPPGAPGPSGIMGRMGFPGKDGQDGHDGDRGDSGEEG-----------------------	82
***Macaca mulatta* ADIH**	46	GPPGAPGPSGMMGRMGFPGKDGQDGQDGDRGDSGEEG-----------------------	82
***Papio hamadryas* ADIH**	46	GPPGAPGPSGMMGRMGFPGKDGQDGQDGDRGDSGEEG-----------------------	82
***Mus musculus* Adih**	53	GPPGAPGSSGVVGRMGFPGKDGQDGQDGDRGDSGEEG-----------------------	89
***Rattus norvegicus* Adih**	90	GPPGAPGSSGMVGRMGFPGKDGQDGQDGDRGDSGEEG-----------------------	126
***Dipodomys ordii* ADIH**	46	GPPGVPGPSGTVGRMGFPGKDGQDGQDGDRGDSGEEG-----------------------	82
***Cavia porcellus* ADIH**	46	GPPGAPGPSGTVGRMGFPGKDGQDGQDGDRGDNGEEG-----------------------	82
***Bos taurus* ADIH**	63	GPPGAPGPSGMVGRMGFPGRDGQDGQDGDRGETGEEG-----------------------	99
***Equus caballus* ADIH**	46	GPPGAPGPSGMVGRMGFPGKDGQDGQDGDRGDNGEEG-----------------------	82
***Canis lupus familiaris* ADIH**	46	GPPGAPGPSGMVGRMGFPGKDGQDGQDGDRGDSGEEG-----------------------	82
***Pteropus vampyrus* ADIH**	46	GPPGTPGLSGMVGRMGFPGKDGQDGQDGDRGDRGEEG-----------------------	82
***Loxodonta africana* ADIH**	46	GPPGAPGPSGMVGRMGFPGKDGQDGQDGDRGDNGEEG-----------------------	82
***Homo sapiens* ADII**	91	GPPGEPGPPGPRGPPGEKGDSGRPGLP------GLQ------------------------	120
***Pongo abelii* ADII**	91	GPPGEPGPPGPRGPPGEKGDSGRPGLP------GLQ------------------------	120
***Macaca mulatta* ADII**	91	GPPGEPGPPGPRGPPGEKGDSGRPGLP------GLQ------------------------	120
***Mus musculus* Adii**	91	GPPGEPGPPGPRGPPGEKGDSGRPGLP------GLQ------------------------	120
***Rattus norvegicus* Adii**	91	GPPGEPGPPGPRGPPGEKGDSGRPGLP------GLQ------------------------	120
***Bos taurus* ADII**	91	GPPGEPGPPGPRGPPGEKGDSGRPGLP------GLQ------------------------	120
***Canis lupus familiaris* ADII**	91	GPPGEPGPPGPRGPPGEKGDSGRPGLP------GLQ------------------------	120
***Dasypus novemcinctus* ADII**	91	GPPGEPGPPGPRGPPGEKGDSGRPGLP------GLQ------------------------	120
***Homo sapiens* ADIJ**	76	GPPGEPGPPGPMGPPGEKGEPGRQGLPGPPGAPGLN------------------------	111
***Pan troglodytes* ADIJ**	76	GPPGEPGPPGPMGPPGEKGEPGRQGLPGPPGAPGLN------------------------	111
***Papio hamadryas* ADIJ**	76	GPPGEPGPPGPMGPPGEKGEPGRQGLPGPPGAPGLN------------------------	111
***Mus musculus* Adij**	76	GPPGEPGPPGPVGPPGEKGEPGRQGLPGPPGAPGLN------------------------	111
***Rattus norvegicus* Adij**	76	GPPGEPGPPGPVGPPGEKGEPGRQGLPGPPGAPGLN------------------------	111
***Oryctolagus cuniculus* ADIJ**	76	GPPGEPGPPGPVGPPGEKGEPGRQGLPGPPGAPGLN------------------------	111
***Bos taurus* ADIJ**	76	GPPGEPGPPGPVGPPGEKGEPGRQGLPGPPGAPGLN------------------------	111
***Canis lupus familiaris* ADIJ**	76	GPPGEPGPPGPVGPPGEKGEPGRQGLPGPPGAPGLN------------------------	111
***Loxodonta africana* ADIJ**	76	GPPGEPGPPGPVGPPGEKGEPGRQGLPGPPGAPGLN------------------------	111
***Homo sapiens* ADIK**	70	GPP---GPPGPRGPP---GEPGRPGPPGPP---GPG------------------------	96
***Pan troglodytes* ADIK**	70	GPP---GPPGPRGPP---GEPGRPGPPGPP---GPG------------------------	96
***Gorilla gorilla* ADIK**	70	GPP---GPPGPRGPP---GEPGRPGPPGPP---GPG------------------------	96
***Papio hamadryas* ADIK**	70	GPP---GPPGPRGPP---GEPGRPGPPGPP---GPG------------------------	96
***Callithrix jacchus* ADIK**	70	GPP---GPPGPRGPP---GEPGRPGPPGPP---GPG------------------------	96
***Rattus norvegicus* Adik**	70	GPP---GPPGPRGPP---GEPGRPGPPGPP---GPG------------------------	96
***Cavia porcellus* ADIK**	70	GPP---GPPGPRGPP---GEPGRPGPPGPP---GPG------------------------	96
***Equus caballus* ADIK**	70	GPP---GPPGPRGPP---GEPGKPGPPGPP---GPG------------------------	96
***Canis lupus familiaris* ADIK**	70	GPP---GPPGPRGPP---GEPGRPGPPGPP---GPG------------------------	96
***Dasypus novemcinctus* ADIK**	70	GPP---GPPGPRGPP---GEPGRPGPPGPP---GPG------------------------	96
***Loxodonta africana* ADIK**	70	GPP---GPPGPRGPP---GEPGRPGPPGPP---GPG------------------------	96
***Mus musculus* Adil**	82	GPPGDRGPPGPVGPPGEKGEPGKPGPPGLPGSGGSG------------------------	117
***Rattus norvegicus* Adil**	82	GPPGDRGPPGPVGPPGEKGEPGKPGPPGLPGSGGSG------------------------	117
***Homo sapiens* ADIM**	159	------------SFRGYQGPPGPPGPPGIP------G-----------------------	177
***Pan troglodytes* ADIM**	159	------------SFRGYQGPPGPPGPPGIP------G-----------------------	177

***Pongo abelii* ADIM**	115	------------SFRGYQGPPGPPGPPGIP------G-----------------------	133
***Nomascus leucogenys* ADIM**	159	------------SFRGYQGPPGPPGPPGIP------G-----------------------	177
***Papio hamadryas* ADIM**	159	------------SFRGYQGPPGPPGPPGIP------G-----------------------	177
***Callithrix jacchus* ADIM**	149	------------SFRGYQGPPGPPGPPGIP------G-----------------------	167
***Mus musculus* Adim**	48	------------GFRGYQGPPGPPGPPGIP------G-----------------------	66
***Rattus norvegicus* Adim**	122	------------GFRGYQGPPGPPGPPGIP------G-----------------------	140
***Cavia porcellus* ADIM**	115	------------GFRGYQGPPGPPGPPGIP------G-----------------------	133
***Bos taurus* ADIM**	48	------------SFRGYQGPPGPPGPPGIP------G-----------------------	66
***Equus caballus* ADIM**	48	------------SFRGYQGPPGPPGPPGIP------G-----------------------	66
***Canis lupus familiaris* ADIM**	109	------------SFRGYQGPPGPPGPPGIP------G-----------------------	127
***Homo sapiens* ADIN**	-	--	-
***Pan troglodytes* ADIN**	-	--	-
***Pongo abelii* ADIN**	-	--	-
***Nomascus leucogenys* ADIN**	-	--	-
***Macaca mulatta* ADIN**	-	--	-
***Callithrix jacchus* ADIN**	-	--	-
***Mus musculus* Adin**	-	--	-
***Cavia porcellus* ADIN**	-	--	-
***Tursiops truncatus* ADIN**	-	--	-
***Canis lupus familiaris* ADIN**	-	--	-
***Dasypus novemcinctus* ADIN**	-	--	-
***Loxodonta africana* ADIN**	-	--	-
***Homo sapiens* ADIO**	70	------------------------------------G-----------------------	70
***Pan troglodytes* ADIO**	70	------------------------------------G-----------------------	70
***Macaca mulatta* ADIO**	65	------------------------------------G-----------------------	65
***Bos taurus* ADIO**	69	------------------------------------G-----------------------	69
***Myotis lucifugus* ADIO**	69	------------------------------------G-----------------------	69
***Homo sapiens* ADIP**	197	------------------------------------G-----------------------	197
***Otolemur garnettii* ADIP**	99	------------------------------------G-----------------------	99
***Mus musculus* Adip**	99	------------------------------------G-----------------------	99
***Cavia porcellus* ADIP**	99	------------------------------------G-----------------------	99
***Bos taurus* ADIP**	122	------------------------------------G-----------------------	122
***Equus caballus* ADIP**	97	------------------------------------G-----------------------	97
***Loxodonta africana* ADIP**	151	------------------------------------G-----------------------	151
***Homo sapiens* ADIQ**	97	------------------------------------G-----------------------	97
***Pan troglodytes* ADIQ**	97	------------------------------------G-----------------------	97
***Pongo abelii* ADIQ**	97	------------------------------------G-----------------------	97
***Nomascus leucogenys* ADIQ**	97	------------------------------------G-----------------------	97
***Macaca mulatta* ADIQ**	97	------------------------------------G-----------------------	97
***Mus musculus* Adiq**	83	------------------------------------G-----------------------	83
***Rattus norvegicus* Adiq**	82	------------------------------------G-----------------------	82
***Homo sapiens* ADIR**	-	--	-
***Pan troglodytes* ADIR**	-	--	-
***Pongo abelii* ADIR**	-	--	-
***Nomascus leucogenys* ADIR**	-	--	-
***Macaca mulatta* ADIR**	-	--	-
***Papio hamadryas* ADIR**	-	--	-
***Callithrix jacchus* ADIR**	-	--	-
***Tarsius syrichta* ADIR**	-	--	-
***Otolemur garnettii* ADIR**	-	--	-
***Tupaia belangeri* ADIR**	-	--	-
***Mus musculus* Adir**	-	--	-
***Rattus norvegicus* Adir**	-	--	-
***Cavia porcellus* ADIR**	-	--	-
***Oryctolagus cuniculus* ADIR**	-	--	-
***Tursiops truncatus* ADIR**	-	--	-
***Bos taurus* ADIR**	-	--	-
***Equus caballus* ADIR**	-	--	-
***Canis lupus familiaris* ADIR**	-	--	-
***Myotis lucifugus* ADIR**	-	--	-
***Pteropus vampyrus* ADIR**	-	--	-
***Erinaceus europaeus* ADIR**	-	--	-
***Dasypus novemcinctus* ADIR**	-	--	-
***Choloepus hoffmanni* ADIR**	-	--	-
***Loxodonta africana* ADIR**	-	--	-

```
Homo sapiens ADIA             - ------------------------------------------------------------   -
                                        370       380       390       400       410       420

Homo sapiens ADIA             - ------------------------------------------------------------   -
Papio hamadryas ADIA          - ------------------------------------------------------------   -
Mus musculus Adia             - ------------------------------------------------------------   -
Rattus norvegicus Adia        - ------------------------------------------------------------   -
Cavia porcellus ADIA          - ------------------------------------------------------------   -
Ochotona princeps ADIA        - ------------------------------------------------------------   -
Bos taurus ADIA               - ------------------------------------------------------------   -
Equus caballus ADIA           - ------------------------------------------------------------   -
Canis lupus familiaris ADIA   - ------------------------------------------------------------   -
Felis catus ADIA              - ------------------------------------------------------------   -
Myotis lucifugus ADIA         - ------------------------------------------------------------   -
Dasypus novemcinctus ADIA     - ------------------------------------------------------------   -
Loxodonta africana ADIA       - ------------------------------------------------------------   -
Homo sapiens ADIB             - ------------------------------------------------------------   -
Pan troglodytes ADIB          - ------------------------------------------------------------   -
Pongo abelii ADIB             - ------------------------------------------------------------   -
Nomascus leucogenys ADIB      - ------------------------------------------------------------   -
Callithrix jacchus ADIB       - ------------------------------------------------------------   -
Otolemur garnettii ADIB       - ------------------------------------------------------------   -
Mus musculus Adib             - ------------------------------------------------------------   -
Rattus norvegicus Adib        - ------------------------------------------------------------   -
Cavia porcellus ADIB          - ------------------------------------------------------------   -
Oryctolagus cuniculus ADIB    - ------------------------------------------------------------   -
Bos taurus ADIB               - ------------------------------------------------------------   -
Equus caballus ADIB           - ------------------------------------------------------------   -
Canis lupus familiaris ADIB   - ------------------------------------------------------------   -
Myotis lucifugus ADIB         - ------------------------------------------------------------   -
Loxodonta africana ADIB       - ------------------------------------------------------------   -
Homo sapiens ADIC             - ------------------------------------------------------------   -
Macaca mulatta ADIC           - ------------------------------------------------------------   -
Papio hamadryas ADIC          - ------------------------------------------------------------   -
Callithrix jacchus ADIC       - ------------------------------------------------------------   -
Mus musculus Adic             - ------------------------------------------------------------   -
Rattus norvegicus Adic        - ------------------------------------------------------------   -
Cavia porcellus ADIC          - ------------------------------------------------------------   -
Oryctolagus cuniculus ADIC    - ------------------------------------------------------------   -
Equus caballus ADIC           - ------------------------------------------------------------   -
Canis lupus familiaris ADIC   - ------------------------------------------------------------   -
Myotis lucifugus ADIC         - ------------------------------------------------------------   -
Loxodonta africana ADIC       - ------------------------------------------------------------   -
Homo sapiens ADID             - ------------------------------------------------------------   -
Pan troglodytes ADID          - ------------------------------------------------------------   -
Pongo abelii ADID             - ------------------------------------------------------------   -
Nomascus leucogenys ADID      - ------------------------------------------------------------   -
Macaca mulatta ADID           - ------------------------------------------------------------   -
Otolemur garnettii ADID       - ------------------------------------------------------------   -
Mus musculus Adid             - ------------------------------------------------------------   -
Rattus norvegicus Adid        - ------------------------------------------------------------   -
Cavia porcellus ADID          - ------------------------------------------------------------   -
Oryctolagus cuniculus ADID    - ------------------------------------------------------------   -
Bos taurus ADID               - ------------------------------------------------------------   -
Equus caballus ADID           - ------------------------------------------------------------   -
Canis lupus familiaris ADID   - ------------------------------------------------------------   -
Felis catus ADID              - ------------------------------------------------------------   -
Myotis lucifugus ADID         - ------------------------------------------------------------   -
Dasypus novemcinctus ADID     - ------------------------------------------------------------   -
Loxodonta africana ADID       - ------------------------------------------------------------   -
Homo sapiens ADIE             - ------------------------------------------------------------   -
Pan troglodytes ADIE          - ------------------------------------------------------------   -
Pongo abelii ADIE             - ------------------------------------------------------------   -
Papio hamadryas ADIE          - ------------------------------------------------------------   -
Callithrix jacchus ADIE       - ------------------------------------------------------------   -
Otolemur garnettii ADIE       - ------------------------------------------------------------   -
Mus musculus Adie             - ------------------------------------------------------------   -
Rattus norvegicus Adie        - ------------------------------------------------------------   -
Homo sapiens ADIF1           80 GKVEAKGIKGDQGSRGSPGKHGPKGLAGPMGEKGLRGETGPQGQKGNKGDVGPTGPEGPR 139
Homo sapiens ADIF2           80 GKVEAKGIKGDQGSRGSPGKHGPKGLAGPMGEKGLRGETGPQGQKGNKGDVGPTGPEGPR 139
Pan troglodytes ADIF1        80 GKVEAKGIKGDQGSRGSPGKHGPKGLAGPMGEKGLQGETGPQGQKGNKGDVGPTGPEGPR 139
Pongo abelii ADIF1           80 GKVEAKGIKGDQGSRGSPGKHGPKGLAGPMGEKGLRGETGPQGQKGNKGDVGPTGPEGPR 139
Nomascus leucogenys ADIF1    80 GKVEAKGIKGDQGSRGSPGKHGPKGLAGPMGEKGLRGETGPQGQKGNKGDVGPTGPEGPS 139
Macaca mulatta ADIF1         80 GKVEAKGIKGDQGSRGSPGKHGPKGLAGPMGEKGLRGETGPQGQKGNKGDVGPTGPEGPR 139
Papio hamadryas ADIF1        80 GKVEAKGIKGDQGSRGSPGKHGPKGLAGPMGEKGLRGETGPQGQKGNKGDVGPTGPEGPR 139
```

```
Microcebus murinus ADIF1          80 GNVEAKGIKGDQGSRGPPGKHGPKGLLGPMGEKGLQGETGPPGQKGEKGDVGPPGREGQR 139
Otolemur garnettii ADIF1          80 GNVEAKGIKGDQGSKGPPGKHGPKGLVGPMGEKGIQGEPGPPGEKGDKGDVGLPGPEGLR 139
Mus musculus Adif1               109 GRVEAKGIKGDPGSRGSPGKHGPKGSIGPTGEQGLPGETGPQGQKGDKGEVGPTGPEGLM 168
Rattus norvegicus Adif1           96 GRVEAKGIKGDPGSRGSPGKHGPKGSIGPTGEQGLPGETGPQGQKGDKGEVGPTGPEGLM 155
Cavia porcellus ADIF1             80 GKVEAKGIKGDPGSRGPPGKHGPKGSVGPTGERGLQGEMGPQGQKGDKGDVGSPGPRGLT 139
Oryctolagus cuniculus ADIF1       80 GKVEAKGIKGDQGSRGPPGKHGPKGSVGPMGEPGLQGETGLQGQKGDKGDVGPRGPEGLT 139
Ochotona princeps ADIF1           80 GKVEAKGMKGDPGSRGSPGKHGPKGSVGPVGEPGLQGETGPQGQKGEKGDVGPMGPEGQT 139
Bos taurus ADIF1                  80 GHVEAKGIKGDQGSRGPPGKHGPKGLVGPPGEKGLTGETGPQGQKGEKGDVGPVGPEGPE 139
Equus caballus ADIF1              80 GKVEAKGVKGDQGSRGSPGKHGPKGFVGPMGEKGLRGETGPQGQKGDKGDVGPTGPEGLK 139
Canis lupus familiaris ADIF1      80 GKVEAKGIKGDQGSRGPPGKHGPKGLVGPMGEKGLRGETGPQGQKGDKGGVGPSGPEGLK 139
Felis catus ADIF1                 80 GKVEAKGIKGDQGSQGPPGKHGPKGFVGPMGEKGLQGETGPRGPKGEKGDVGPTGPQGLQ 139
Dasypus novemcinctus ADIF1        81 GRVEAKGIKGEQGSIGPPGKHGPKGFVGPMGEKGLRGETGPQGQKGGKGEVGPTGPGGLQ 140
Loxodonta africana ADIF1          80 GKVEAKGIKGEPGSRGSPGKHGPKGFVGPMGEKGLKGETGPQGQKGSKGEVGPIGPEGLQ 139
Homo sapiens ADIG                135 ----------------------------------------------------------LR 136
Pan troglodytes ADIG             135 ----------------------------------------------------------LR 136
Pongo abelii ADIG                135 ----------------------------------------------------------LR 136
Callithrix jacchus ADIG          121 ----------------------------------------------------------LR 122
Otolemur garnettii ADIG           81 ----------------------------------------------------------LR  82
Mus musculus Adig                123 ----------------------------------------------------------LK 124
Rattus norvegicus Adig           124 ----------------------------------------------------------LK 125
Cavia porcellus ADIG              81 ----------------------------------------------------------LR  82
Oryctolagus cuniculus ADIG        81 ----------------------------------------------------------LR  82
Tursiops truncatus ADIG          133 ----------------------------------------------------------LR 134
Bos taurus ADIG                   81 ----------------------------------------------------------LR  82
Equus caballus ADIG              122 ----------------------------------------------------------LR 123
Canis lupus familiaris ADIG      122 ----------------------------------------------------------LR 123
Myotis lucifugus ADIG             81 ----------------------------------------------------------LR  82
Homo sapiens ADIH                 83 ----------------------------------------------------------PP  84
Pan troglodytes ADIH              83 ----------------------------------------------------------PP  84
Pongo abelii ADIH                 83 ----------------------------------------------------------PP  84
Nomascus leucogenys ADIH          83 ----------------------------------------------------------PP  84
Macaca mulatta ADIH               83 ----------------------------------------------------------PP  84
Papio hamadryas ADIH              83 ----------------------------------------------------------PP  84
Mus musculus Adih                 90 ----------------------------------------------------------PP  91
Rattus norvegicus Adih           127 ----------------------------------------------------------PP 128
Dipodomys ordii ADIH              83 ----------------------------------------------------------PP  84
Cavia porcellus ADIH              83 ----------------------------------------------------------PP  84
Bos taurus ADIH                  100 ----------------------------------------------------------SP 101
Equus caballus ADIH               83 ----------------------------------------------------------PP  84
Canis lupus familiaris ADIH       83 ----------------------------------------------------------PP  84
Pteropus vampyrus ADIH            83 ----------------------------------------------------------PP  84
Loxodonta africana ADIH           83 ----------------------------------------------------------AP  84
Homo sapiens ADII                  - ------------------------------------------------------------   -
Pongo abelii ADII                  - ------------------------------------------------------------   -
Macaca mulatta ADII                - ------------------------------------------------------------   -
Mus musculus Adii                  - ------------------------------------------------------------   -
Rattus norvegicus Adii             - ------------------------------------------------------------   -
Bos taurus ADII                    - ------------------------------------------------------------   -
Canis lupus familiaris ADII        - ------------------------------------------------------------   -
Dasypus novemcinctus ADII          - ------------------------------------------------------------   -
Homo sapiens ADIJ                  - ------------------------------------------------------------   -
Pan troglodytes ADIJ               - ------------------------------------------------------------   -
Papio hamadryas ADIJ               - ------------------------------------------------------------   -
Mus musculus Adij                  - ------------------------------------------------------------   -
Rattus norvegicus Adij             - ------------------------------------------------------------   -
Oryctolagus cuniculus ADIJ         - ------------------------------------------------------------   -
Bos taurus ADIJ                    - ------------------------------------------------------------   -
Canis lupus familiaris ADIJ        - ------------------------------------------------------------   -
Loxodonta africana ADIJ            - ------------------------------------------------------------   -
Homo sapiens ADIK                  - ------------------------------------------------------------   -
Pan troglodytes ADIK               - ------------------------------------------------------------   -
Gorilla gorilla ADIK               - ------------------------------------------------------------   -
Papio hamadryas ADIK               - ------------------------------------------------------------   -
Callithrix jacchus ADIK            - ------------------------------------------------------------   -
Rattus norvegicus Adik             - ------------------------------------------------------------   -
Cavia porcellus ADIK               - ------------------------------------------------------------   -
Equus caballus ADIK                - ------------------------------------------------------------   -
Canis lupus familiaris ADIK        - ------------------------------------------------------------   -
Dasypus novemcinctus ADIK          - ------------------------------------------------------------   -
Loxodonta africana ADIK            - ------------------------------------------------------------   -
Mus musculus Adil                  - ------------------------------------------------------------   -
Rattus norvegicus Adil             - ------------------------------------------------------------   -
Homo sapiens ADIM                  - ------------------------------------------------------------   -
Pan troglodytes ADIM               - ------------------------------------------------------------   -
```

Pongo abelii ADIM	-	--	-
Nomascus leucogenys ADIM	-	--	-
Papio hamadryas ADIM	-	--	-
Callithrix jacchus ADIM	-	--	-
Mus musculus Adim	-	--	-
Rattus norvegicus Adim	-	--	-
Cavia porcellus ADIM	-	--	-
Bos taurus ADIM	-	--	-
Equus caballus ADIM	-	--	-
Canis lupus familiaris ADIM	-	--	-
Homo sapiens ADIN	-	--	-
Pan troglodytes ADIN	-	--	-
Pongo abelii ADIN	-	--	-
Nomascus leucogenys ADIN	-	--	-
Macaca mulatta ADIN	-	--	-
Callithrix jacchus ADIN	-	--	-
Mus musculus Adin	-	--	-
Cavia porcellus ADIN	-	--	-
Tursiops truncatus ADIN	-	--	-
Canis lupus familiaris ADIN	-	--	-
Dasypus novemcinctus ADIN	-	--	-
Loxodonta africana ADIN	-	--	-
Homo sapiens ADIO	-	--	-
Pan troglodytes ADIO	-	--	-
Macaca mulatta ADIO	-	--	-
Bos taurus ADIO	-	--	-
Myotis lucifugus ADIO	-	--	-
Homo sapiens ADIP	-	--	-
Otolemur garnettii ADIP	-	--	-
Mus musculus Adip	-	--	-
Cavia porcellus ADIP	-	--	-
Bos taurus ADIP	-	--	-
Equus caballus ADIP	-	--	-
Loxodonta africana ADIP	-	--	-
Homo sapiens ADIQ	-	--	-
Pan troglodytes ADIQ	-	--	-
Pongo abelii ADIQ	-	--	-
Nomascus leucogenys ADIQ	-	--	-
Macaca mulatta ADIQ	-	--	-
Mus musculus Adiq	-	--	-
Rattus norvegicus Adiq	-	--	-
Homo sapiens ADIR	-	--	-
Pan troglodytes ADIR	-	--	-
Pongo abelii ADIR	-	--	-
Nomascus leucogenys ADIR	-	--	-
Macaca mulatta ADIR	-	--	-
Papio hamadryas ADIR	-	--	-
Callithrix jacchus ADIR	-	--	-
Tarsius syrichta ADIR	-	--	-
Otolemur garnettii ADIR	-	--	-
Tupaia belangeri ADIR	-	--	-
Mus musculus Adir	-	--	-
Rattus norvegicus Adir	-	--	-
Cavia porcellus ADIR	-	--	-
Oryctolagus cuniculus ADIR	-	--	-
Tursiops truncatus ADIR	-	--	-
Bos taurus ADIR	-	--	-
Equus caballus ADIR	-	--	-
Canis lupus familiaris ADIR	-	--	-
Myotis lucifugus ADIR	-	--	-
Pteropus vampyrus ADIR	-	--	-
Erinaceus europaeus ADIR	-	--	-
Dasypus novemcinctus ADIR	-	--	-
Choloepus hoffmanni ADIR	-	--	-
Loxodonta africana ADIR	-	--	-

***Homo sapiens* ADIA**	64	**-LPGL---AGDHGEFGEKGDPGIPGNPGKVGPKGPMGPKGGPGAPGAPGPKGESGDY---**	116
		430 440 450 460 470 480	
***Homo sapiens* ADIA**	64	-LPGL---AGDHGEFGEKGDPGIPGNPGKVGPKGPMGPKGGPGAPGAPGPKGESGDY---	116
***Papio hamadryas* ADIA**	64	-LPGL---AGDHGEFGEKGDPGIPGNPGKVGPKGPMGPKGGPGAPGAPGPKGESGDY---	116
***Mus musculus* Adia**	91	-LPGL---AGDLGEFGEKGDPGIPGTPGKVGPKGPVGPKGTPGPSGPRGPKGDSGDY---	143
***Rattus norvegicus* Adia**	62	-LPGL---AGDHGELGEKGDAGIPGIPGKVGPKGPVGPKGAPGPPGPRGPKGDSGDY---	114
***Cavia porcellus* ADIA**	62	-SPGL---AGDHGEFGEKGDPGIPGNPGKVGPKGPIGPKGAPGIPGGPGPKGESGDY---	114
***Ochotona princeps* ADIA**	62	-LPGL---AGDHGEFGEKGDPGSPGNPGKVGPKGPVGPKGVPGAPGAPGPKGESGDY---	114
***Bos taurus* ADIA**	58	-LPGL---VSHLNENGEKGDPGFPGMPGKVGPKGPIGPKGVPGPPGVRGPKGESGDY---	110
***Equus caballus* ADIA**	63	-LPGL---AAHRGNFGEKGEPGIPGNPGKVGPKGPAGPKGAPGPPGARGPKGESGDY---	115
***Canis lupus familiaris* ADIA**	61	-LPGL---AGDHGEFGEKGDPGIPGTPGKVGPKGPVGPKGSPGPPGARGAKGESGDY---	113
***Felis catus* ADIA**	65	-LPGL---AGDHGEFGEKGDPGIPGNPGKVGPKGPVGPKGSPGPPGARGPKGESGDY---	117
***Myotis lucifugus* ADIA**	62	-LPGL---AGDHGEFGEKGDPGTPGKPGKVGPKGPVGPKGAPGLPGLRGPKGESGDY---	114
***Dasypus novemcinctus* ADIA**	59	-LPGL---AGDHGEYGEKGDPGIPGNPGKVGPKGPVGPKGSPGPPGARGPKGESGDY---	111
***Loxodonta africana* ADIA**	62	-LPGL---AGDHGEFGEKGDPGTPGNPGKVGPKGPIGPKGSPGSPGARGPKGESGDY---	114
***Homo sapiens* ADIB**	62	-IPAI---PGIRGPKGQKGEPGLPGHPGKNGPMGPPGMPGVPGPMGIPGEPGEEGRY---	114
***Pan troglodytes* ADIB**	62	-IPAI---PGIRGPKGQKGEPGLPGHPGKNGPMGPPGMPGVPGPMGIPGEPGEEGRY---	114
***Pongo abelii* ADIB**	62	-IPAI---PGIRGPKGQKGEPGLPGHPGKNGPMGPPGMPGVPGPMGIPGEPGEEGRY---	114
***Nomascus leucogenys* ADIB**	63	-IPAI---PGIRGPKGQKGEPGLPGHPGKNGPMGPPGMPGVPGPMGIPGEPGEEGRY---	115
***Callithrix jacchus* ADIB**	61	-IPAI---PGTRGPKGQKGEPGTPGHPGKNGPMGPPGIPGVPGPIGIPGEPGEEGRY---	113
***Otolemur garnettii* ADIB**	60	-MPAI---PGTRGPKGHKGEPGIPGHPGKNGPMGPPGLPGVPGFMGPPGEPGEEGRY---	112
***Mus musculus* Adib**	93	-IPAV---PGTRGPKGQKGEPGMPGHRGKNGPRGTSGLPGDPGPRGPPGEPGVEGRY---	145
***Rattus norvegicus* Adib**	85	-IPAI---PGTQGPKGQKGEPGMPGHRGKNGPMGTSGSPGDPGPRGPPGEPGEEGRY---	137
***Cavia porcellus* ADIB**	109	-IPAI---PGTRGPKGHKGEPGTPGHPGKNGPMGAPGMPGNPGPRGPPGMPGEEGRY---	161
***Oryctolagus cuniculus* ADIB**	115	-IPAI---PGTRGPKGQKGEPGTPGHPGKNGPMGSSGPPGDPGHRGPPGEPGVEGRY---	167
***Bos taurus* ADIB**	60	-IPAA---PGTRGPKGQKGDPGTPGYPGKNGPMGTPGIPGAPGTMGPPGEPGVEGRY---	112
***Equus caballus* ADIB**	80	-IPAV---PGTQGPKGQKGEPGTPGHPGKNGPMGTSGMAGLPGLVGPRGEPGEEGRY---	132
***Canis lupus familiaris* ADIB**	62	-IPAI---PGTRGPKGQKGEPGTPGYPGKNGPMGTPGIPGVPGPVGPPGEPGEEGRY---	114
***Myotis lucifugus* ADIB**	62	-IPAI---PGTRGPKGQKGEAGTPGFPGKNGPMGAAGLPGTPGIMGPNGEPGEEGRY---	114
***Loxodonta africana* ADIB**	60	-IPAT---PRTRGPKGQKGEPGTPGHPGKNGPMGTSGIPGDPGIQGPPGEPGEEGRY---	112
***Homo sapiens* ADIC**	56	-APGIR--TGIQGLKGDQGEPGPSGNPGKVGYPGPSGPLGARGIPGIKGTKGSPGNI---	109
***Macaca mulatta* ADIC**	56	-APGIR--TGIQGLKGDQGEPGPSGNPGKVGYPGPSGPLGDRGIPGIKGIKGNPGNI---	109
***Papio hamadryas* ADIC**	56	-APGIR--TGIQGLKGDQGEPGPSGNPGKVGYPGPSGPLGDRGIPGIKGIKGNPGNI---	109
***Callithrix jacchus* ADIC**	56	-APGIR--TGIQGLKGDQGEPGPPGSPGKMGYPGPSGLPGIHGMPGIKGIKGNPGNI---	109
***Mus musculus* Adic**	56	-AAGIR--TGIRGFKGDPGESGPPGKPGNVGLPGPSGPLGDSGPQGLKGVKGNPGNI---	109
***Rattus norvegicus* Adic**	68	-AAGIR--TGIRGLKGDMGESGPPGKPGNVGFPGPTGPLGNSGPQGLKGVKGNPGNI---	121
***Cavia porcellus* ADIC**	55	-APGIR--TGIRGLKGDQGEPGPPGKPGNVGYPGPSGPPGEQGASGPRGMKGNPGNI---	108
***Oryctolagus cuniculus* ADIC**	97	-APGMR--TGIRGLKGDPGEPGPPGSPGKVGFPGPSGPPGDRGPQGSRGPRGNPGNI---	150
***Equus caballus* ADIC**	89	-APGIQ--TGIRGLKGDQGEPGVPGSPGNMGYPGPSGPPGVPGAPGLKGIKGNPGNL---	142
***Canis lupus familiaris* ADIC**	75	-APGMR--TGIRGLKGDQGDPGPPGNPGNMGFPGPSGLMGLPGIPGRRGPKGNPGNI---	128
***Myotis lucifugus* ADIC**	56	-APGIR--TGIRGLKGDQGEPGPPGIPGNMGYPGPSGPLGNPGAPGLKGIKGNPGNI---	109
***Loxodonta africana* ADIC**	71	-AAGIR--TGIQGLKGDQGDPGPPGKSGKMGFPGPSGPTGISGPPGLKGLKGNPGNI---	124
***Homo sapiens* ADID**	104	-------------------LIGPKGDIGETGVPGAEGPRGFPGIQGRKGEP---GEG---	138
***Pan troglodytes* ADID**	104	-------------------LIGPKGDIGETGVPGAEGPRGFPGIQGRKGEP---GEG---	138
***Pongo abelii* ADID**	73	-------------------LIGPKGDIGETGVPGAEGPRGFPGIQGRKGEP---GEG---	107
***Nomascus leucogenys* ADID**	103	-------------------LIGPKGDIGETGVPGAEGPRGFPGIQGRKGEP---GEG---	137
***Macaca mulatta* ADID**	102	-------------------LIGPKGDTGETGVTGAEGPRGFPGIQGRKGEP---GEG---	136
***Otolemur garnettii* ADID**	73	-------------------LVGPKGDTGETGVTGAEGPRGFPGTPGRKGEP---GEG---	107
***Mus musculus* Adid**	87	-------------------LLGPKGETGDVGMTGAEGPRGFPGTPGRKGEP---GEA---	121
***Rattus norvegicus* Adid**	73	-------------------VLGPKGDPGDAGMTGAEGPRGFPGTPGRKGEP---GEA---	107
***Cavia porcellus* ADID**	115	-------------------FVGPKGDTGETGVTGAEGPRGFPGVPGRKGER---GQD---	149
***Oryctolagus cuniculus* ADID**	83	-------------------LVGPKGDTGETGVTGAEGPRGFPGSPGRKGEP---GEG---	117
***Bos taurus* ADID**	68	-------------------LVGPKGDTGETGITGIEGPRGFPGTPGRKGEP---GES---	102
***Equus caballus* ADID**	130	-------------------LVGPKGDAGETGVPGVEGPRGFPGIPGRKGEP---GES---	164
***Canis lupus familiaris* ADID**	82	-------------------LVGPKGDTGETGVTGVEGPRGFPGTPGRKGEP---GES---	116
***Felis catus* ADID**	73	-------------------LVGPKGDTGETGVTGIEGPRGFPGIPGRKGEP---GES---	107
***Myotis lucifugus* ADID**	82	-------------------LVGPKGDTGETGVTGVEGPRGFPGVQGRKGEP---GES---	116
***Dasypus novemcinctus* ADID**	72	-------------------LLGPKGDSGEIGVSGVEGPRGFPGIPGRKGEP---GES---	106
***Loxodonta africana* ADID**	73	-------------------LIGPKGDTGETGVTGVEGPRGFPGTPGRKGEP---GEA---	107
***Homo sapiens* ADIE**	73	-------------------------------LPGPRGDPGPRGEAGPAGPTGPAGEC---	98
***Pan troglodytes* ADIE**	73	-------------------------------LPGPRGDPGPRGEAGPAGPTGPAGEC---	98
***Pongo abelii* ADIE**	73	-------------------------------LPGPRGEPGPRGEAGPAGPTGPAGEC---	98
***Papio hamadryas* ADIE**	73	-------------------------------LPGPRGEPGPRGEAGPAGPTGPAGEC---	98
***Callithrix jacchus* ADIE**	73	-------------------------------LPGPRGEPGPRGEAGPAGPTGPVGEC---	98
***Otolemur garnettii* ADIE**	73	-------------------------------LPGPRGEPGPRGEAGPAGATGPAGEC---	98
***Mus musculus* Adie**	73	-------------------------------LPGPRGEPGPRGEAGPMGAIGPAGEC---	98
***Rattus norvegicus* Adie**	73	-------------------------------LPGPRGEPGPRGEAGPVGAIGPAGEC---	98
***Homo sapiens* ADIF1**	140	GNIGPLGPTGLPGPMGPIGKPGPKGEAGPTGPQGEPGVRGIRGWKGDRGEKGKIGET---	196
***Homo sapiens* ADIF2**	140	GNIGPLGPTGLPGPMGPIGKPGPKGEAGPTGPQGEPGVRGIRGWKGDRGEKGKIGET---	196
***Pan troglodytes* ADIF1**	140	GNIGPLGPAGLPGPMGPIGKPGPKGEAGPTGPQGEPGVRGIRGWKGDRGEKGKIGET---	196
***Pongo abelii* ADIF1**	140	GNIGPLGPTGLPGPMGPIGKPGPKGEAGPMGPQGEPGVRGIRGWKGDRGEKGKIGET---	196
***Nomascus leucogenys* ADIF1**	140	GNIGPLGPTGLPGPMGPIGTPGPKGEAGPMGPQGEPGVRGIRGWKGDRGEKGKIGET---	196
***Macaca mulatta* ADIF1**	140	GDIGPLGPTGLPGSMGPIGKPGPKGEAGPMGPQGEPGVRGIRGWKGDRGEKGKIGET---	196
***Papio hamadryas* ADIF1**	140	GDIGPLGPTGLPGSMGPIGKPGPKGEAGPMGPQGEPGVRGIRGWKGDRGEKGKIGET---	196

```
Microcebus murinus ADIF1        140 GHTGPLGPTGLPGPVGPIGKPGPKGEAGPVGPQGEPGVRGIRGWKGDRGEKGKIGET--- 196
Otolemur garnettii ADIF1        140 GNTGPSGPTGVPGPVGPVGKPGLKGEAGPVGPQGEPGVRGLRGWKGDRGEKGKIGET--- 196
Mus musculus Adif1              169 GSTGPLGPKGLPGPMGPIGKPGPRGEAGPMGPQGEPGVRGMRGWKGDRGEKGKVGEA--- 225
Rattus norvegicus Adif1         156 GSTGPLGPKGLPGPVGPIGKPGPRGEAGPMGPQGEPGVRGMRGWKGDRGEKGKVGEA--- 212
Cavia porcellus ADIF1           140 GSTGPLGPTGLTGPMGPIGKPGPKGEAGPIGPQGEPGVRGIRGWKGDRGEKGKIGES--- 196
Oryctolagus cuniculus ADIF1     140 GDPGPLGPNGLPGPVGPIGKPGPKGEAGPVGPQGEPGVRGIRGWKGERGEKGKIGET--- 196
Ochotona princeps ADIF1         140 GDPGPAGPMGLRGPVGPIGKPGPKGDAGPVGPQGEPGVRGMRGWKGDRGEKGKIGET--- 196
Bos taurus ADIF1                140 GSTGPSGPTGLLGPTGPIGKPGPKGDAGPLGPQGEPGVRGPRGWKGERGEKGKIGEM--- 196
Equus caballus ADIF1            140 GNTGLSGPPGLPGPIGPIGKPGPKGDAGPLGPQGEPGVRGMRGWKGDRGEKGKIGET--- 196
Canis lupus familiaris ADIF1    140 GSTGPSGPMGLPGPMGPIGKPGPKGDAGPLGPQGEPGVRGMRGWKGDRGEKGKIGET--- 196
Felis catus ADIF1               140 GHTGPSGPTGLPGPMGPVGKPGPKGDAGPLGPQGEPGVRGMRGWKGDRGEKGKIGET--- 196
Dasypus novemcinctus ADIF1      141 GNAGPSGPMGPPGPVGPIGKPGPKGEAGPLGPQGEPGGRGMRGWKGDRGEKGKMGET--- 197
Loxodonta africana ADIF1        140 GNPGPLGTTGLRGPVGPIGKPGPKGDAGPIGPQGEPGDRGMRGWKGDRGEKGKIGET--- 196
Homo sapiens ADIG               137 GKTGPLGLAGEKGDQGETGKKGPIGPEGEKGEVGPIGPPGPKGDRGEQGDPGLPGVC--- 193
Pan troglodytes ADIG            137 GKTGPLGLAGEKGDQGETGKKGPIGPEGEKGEVGPIGPPGPKGDRGEQGDPGLPGVC--- 193
Pongo abelii ADIG               137 GKTGPLGLAGEKGDQGETGKKGPMGPEGEKGEVGPVGPPGPKGDRGEQGDPGLPGVC--- 193
Callithrix jacchus ADIG         123 GKTGPLGLTGEKGDQGETGKKGPMGPEGDKGELGPAGPPGPKGDRGEQGDPGLPGVC--- 179
Otolemur garnettii ADIG          83 GKTGPVGLAGEKGDQGETGKKGPMGPEGEKGEVGPVGPPGPKGDRGEQGEPGLPGVC--- 139
Mus musculus Adig               125 GKTGPLGLAGEKGDQGETGKKGPIGPEGEKGEVGPAGPPGPKGDRGDQGDPGLPGVC--- 181
Rattus norvegicus Adig          126 GKTGPLGLAGEKGDQGETGKKGPIGPEGEKGEVGPAGPPGPKGDRGDQGDPGLPGVC--- 182
Cavia porcellus ADIG             83 GKTGPLGLAGEKGDQGERGKKGPIGPEGQKGEVGPAGPPGPKGDRGDQGDPGLPGVC--- 139
Oryctolagus cuniculus ADIG       83 GKTGPLGLAGEKGDQGETGKKGPMGPEGEKGEVGPAGPPGPKGDRGDQGDPGLPGVC--- 139
Tursiops truncatus ADIG         135 GKTGPLGLAGEKGDQGETGKKGPMGPEGEKGEVGPVGPPGPKGDRGEQGGPGLPGVC--- 191
Bos taurus ADIG                  83 GKAGPLGLAGEKGDQGETGKKGPMGPEGEKGEVGPPGPPGPKGDRGEQGDPGLPGVC--- 139
Equus caballus ADIG             124 GKTGPLGLAGEKGDQGETGKKGPMGPEGEKGEIGPVGPPGPKGDRGEQGDPGLPGVC--- 180
Canis lupus familiaris ADIG     124 GKTGPLGLAGEKGDQGETGKKGPVGPEGEKGEVGPVGPPGAKGDRGEQGDPGPPGIC--- 180
Myotis lucifugus ADIG            83 GKTGPLGLAGEKGDQGETGKKGPMGPEGEKGEVGPAGPAGPKGDRGEQGDPGLPGVC--- 139
Homo sapiens ADIH                85 GRTGNRGKPGPKGKAGAIGRAGPRGPKGVNGTPGKHGTPGKKGPKGKKGEPGLPGPC--- 141
Pan troglodytes ADIH             85 GRTGNRGKPGPKGKAGAIGRAGPRGPKGVNGTPGKHGTPGKKGPKGKKGEPGLPGPC--- 141
Pongo abelii ADIH                85 GRTGNRGKPGPKGKAGATGRAGPRGPKGVNGTPGKHGTPGKKGPKGKKGEPGLPGPC--- 141
Nomascus leucogenys ADIH         85 GRTGNRGKPGPKGKAGAIGRAGPRGPKGVNGTPGKHGTPGKKGPKGKKGEPGLPGPC--- 141
Macaca mulatta ADIH              85 GRTGNRGKPGPKGKAGAIGRAGPRGPKGVNGTPGKHGTPGKKGPKGKKGEPGLPGPC--- 141
Papio hamadryas ADIH             85 GRTGNRGKPGPKGKAGAIGRAGPRGPKGVNGTPGKHGTPGKKGPKGKKGEPGLPGPC--- 141
Mus musculus Adih                92 GRTGNRGKQGPKGKAGAIGRAGPRGPKGVSGTPGKHGTPGKKGPKGKKGEPGLPGPC--- 148
Rattus norvegicus Adih          129 GRTGNRGKQGPKGKAGAIGRAGPRGPKGVSGTPGKHGIPGKKGPKGKKGEPGLPGPC--- 185
Dipodomys ordii ADIH             85 GRTGNRGKQGPKGKAGAIGRAGPRGPKGLPGAPGKHGTPGKKGPKGRKGEPGLPGPC--- 141
Cavia porcellus ADIH             85 GRTGNRGKQGPKGKAGAIGLAGPRGPKGVSGTPGKHGTPGKKGPKGKKGEPGLPGPC--- 141
Bos taurus ADIH                 102 GRTGNRGKPGPKGKAGAIGRAGPRGPKGVSGAPGKHGTPGKKGPKGKKGEPGLPGPC--- 158
Equus caballus ADIH              85 GRTGNRGKPGPKGKAGAIGRAGPRGPKGVSGAPGKHGTPGKKGPKGRKGEPGLPGPC--- 141
Canis lupus familiaris ADIH      85 GRTGNRGKPGPKGKAGAIGRAGPRGPKGVSGAPGKHGTPGKKGPKGRKGEPGLPGPC--- 141
Pteropus vampyrus ADIH           85 GRTGNRGKPGPKGKAGAIGQAGPRGPKGVSGTPGKHGTPGKKGPKGKKGEPGLPGPC--- 141
Loxodonta africana ADIH          85 GRAGNRGKPGPKGKAGAIGRAGPRGPKGVSGAPGKNGTPGKKGPKGKKGDPGLPGPC--- 141
Homo sapiens ADII                 - ------------------------------------------------------------   -
Pongo abelii ADII                 - ------------------------------------------------------------   -
Macaca mulatta ADII               - ------------------------------------------------------------   -
Mus musculus Adii                 - ------------------------------------------------------------   -
Rattus norvegicus Adii            - ------------------------------------------------------------   -
Bos taurus ADII                   - ------------------------------------------------------------   -
Canis lupus familiaris ADII       - ------------------------------------------------------------   -
Dasypus novemcinctus ADII         - ------------------------------------------------------------   -
Homo sapiens ADIJ                 - ------------------------------------------------------------   -
Pan troglodytes ADIJ              - ------------------------------------------------------------   -
Papio hamadryas ADIJ              - ------------------------------------------------------------   -
Mus musculus Adij                 - ------------------------------------------------------------   -
Rattus norvegicus Adij            - ------------------------------------------------------------   -
Oryctolagus cuniculus ADIJ        - ------------------------------------------------------------   -
Bos taurus ADIJ                   - ------------------------------------------------------------   -
Canis lupus familiaris ADIJ       - ------------------------------------------------------------   -
Loxodonta africana ADIJ           - ------------------------------------------------------------   -
Homo sapiens ADIK                 - ------------------------------------------------------------   -
Pan troglodytes ADIK              - ------------------------------------------------------------   -
Gorilla gorilla ADIK              - ------------------------------------------------------------   -
Papio hamadryas ADIK              - ------------------------------------------------------------   -
Callithrix jacchus ADIK           - ------------------------------------------------------------   -
Rattus norvegicus Adik            - ------------------------------------------------------------   -
Cavia porcellus ADIK              - ------------------------------------------------------------   -
Equus caballus ADIK               - ------------------------------------------------------------   -
Canis lupus familiaris ADIK       - ------------------------------------------------------------   -
Dasypus novemcinctus ADIK         - ------------------------------------------------------------   -
Loxodonta africana ADIK           - ------------------------------------------------------------   -
Mus musculus Adil                 - ------------------------------------------------------------   -
Rattus norvegicus Adil            - ------------------------------------------------------------   -
Homo sapiens ADIM               178 ----------NHGNNGNNGATGHEGAKGEKGDKGDLGPRGERGQHGPKGEKGYPGIP--- 224
Pan troglodytes ADIM            178 ----------NHGNNGNNGATGHEGAKGEKGDKGDLGPRGERGQHGPKGEKGYPGIP--- 224
```

```
Pongo abelii ADIM              134 ----------NHGNNGNNGATGHEGAKGEKGDKGDLGPRGERGQHGPKGEKGYPGIP--- 180
Nomascus leucogenys ADIM       178 ----------NHGNNGNNGATGHEGAKGEKGDKGDLGPRGERGQHGPKGEKGYPGIP--- 224
Papio hamadryas ADIM           178 ----------NHGNNGNNGATGHEGAKGEKGDKGDLGPRGERGQHGPKGEKGYPGIP--- 224
Callithrix jacchus ADIM        168 ----------NHGNNGNNGATGHEGAKGEKGDKGDLGPRGERGQHGPKGEKGYPGIP--- 214
Mus musculus Adim               67 ----------NHGNNGNNGATGHEGAKGEKGDKGDLGPRGERGQHGPKGEKGYPGVP--- 113
Rattus norvegicus Adim         141 ----------NHGNNGNNGATGHEGAKGEKGDKGDLGPRGERGQHGPKGEKGYPGVP--- 187
Cavia porcellus ADIM           134 ----------NHGNNGNNGATGHEGAKGEKGDKGDLGPRGERGQHGPKGEKGSPGIP--- 180
Bos taurus ADIM                 67 ----------NHGNNGNNGATGHEGAKGEKGDKGDLGPRGERGQHGPKGEKGYPGIP--- 113
Equus caballus ADIM             67 ----------NHGNNGNNGATGHEGAKGEKGDKGDLGPRGERGQHGPKGEKGHPGIP--- 113
Canis lupus familiaris ADIM    128 ----------NHGNNGNNGATGHEGAKGEKGDKGDLGPRGERGQHGPKGEKGYPGIP--- 174
Homo sapiens ADIN               17 ---------------------------------------------------------LGP  19
Pan troglodytes ADIN            17 ---------------------------------------------------------LGP  19
Pongo abelii ADIN               17 ---------------------------------------------------------LGP  19
Nomascus leucogenys ADIN        17 ---------------------------------------------------------LGP  19
Macaca mulatta ADIN             17 ---------------------------------------------------------LGP  19
Callithrix jacchus ADIN         17 ---------------------------------------------------------LGP  19
Mus musculus Adin               17 ---------------------------------------------------------LGP  19
Cavia porcellus ADIN            28 ---------------------------------------------------------LGP  30
Tursiops truncatus ADIN         17 ---------------------------------------------------------LGP  19
Canis lupus familiaris ADIN     17 ---------------------------------------------------------LGP  19
Dasypus novemcinctus ADIN       17 ---------------------------------------------------------LGA  19
Loxodonta africana ADIN         17 ---------------------------------------------------------LGP  19
Homo sapiens ADIO               71 ----------------EKGEAGVRGRAGRSGKEGPPGARGLQGRRGQKGQVGPPGAA--- 111
Pan troglodytes ADIO            71 ----------------EKGEAGVRGRAGRSGKEGLPGARGLQGRKGQKGQVGPPGAA--- 111
Macaca mulatta ADIO             66 ----------------EKGEAGVRGRAGRSGKEGPPGARGLQGRKGQKGQVGPPGAP--- 106
Bos taurus ADIO                 70 ----------------EKGEAGVRGRSGRSGKEGPPGSRGLRGRKGQKGQAGLPGAQ--- 110
Myotis lucifugus ADIO           70 ----------------EKGETGVRGRSGRSGKEGPPGSPGLRGHKGQKGQVGLPGTP--- 110
Homo sapiens ADIP              198 ----------------EKGDRGDRGLQGKYGKTGSAGARGHTGPKGQKGSMGAPGER--- 238
Otolemur garnettii ADIP        100 ----------------EKGDRGDRGLQGKYGKTGSAGARGHVGPKGQKGSMGAPGDR--- 140
Mus musculus Adip              100 ----------------EKGDRGDRGLQGKYGKIGSTGPRGHVGPKGQKGSIGAPGNH--- 140
Cavia porcellus ADIP           100 ----------------EKGDRGDRGLQGKYGKTGSVGARGHIGPKGQKGSMGAPGDR--- 140
Bos taurus ADIP                123 ----------------EKGDRGDRGLQGKYGKTGSVGARGHTGPKGQKGSMGAPGDR--- 163
Equus caballus ADIP             98 ----------------EKGDRGDRGLQGKYGKPGSVGARGHVGPKGQKGSMGAPGDR--- 138
Loxodonta africana ADIP        152 ----------------EKGDRGDRGLQGKYGKTGSVGARGHMGPKGQKGSVGAPGDR--- 192
Homo sapiens ADIQ               98 ----------------DKGDPGPMGLPGYMGREGPQGEPGPQGSKGDKGEMGSPGAP--- 138
Pan troglodytes ADIQ            98 ----------------DKGDPGPMGLPGYMGREGPQGEPGPQGSKGDKGEMGSPGAP--- 138
Pongo abelii ADIQ               98 ----------------DKGDPGPMGLPGYMGREGPQGEPGPQGSKGDKGEMGSPGAP--- 138
Nomascus leucogenys ADIQ        98 ----------------DKGDPGPMGLPGYMGREGPQGEPGPQGSKGDKGEMGSPGAP--- 138
Macaca mulatta ADIQ             98 ----------------DKGDPGAMGLPGHMGREGPQGEPGPQGSKGDKGEMGSPSAP--- 138
Mus musculus Adiq               84 ----------------DKGDRGPTGTPGKPGKNGTRGDRGSQGVKGDKGQAGSPGSS--- 124
Rattus norvegicus Adiq          83 ----------------DKGDRGPSGTPGKPGKNGTRGDRGSQGIKGDKGQAGSPGSS--- 123
Homo sapiens ADIR                - ------------------------------------------------------------   -
Pan troglodytes ADIR             - ------------------------------------------------------------   -
Pongo abelii ADIR                - ------------------------------------------------------------   -
Nomascus leucogenys ADIR         - ------------------------------------------------------------   -
Macaca mulatta ADIR              - ------------------------------------------------------------   -
Papio hamadryas ADIR             - ------------------------------------------------------------   -
Callithrix jacchus ADIR          - ------------------------------------------------------------   -
Tarsius syrichta ADIR            - ------------------------------------------------------------   -
Otolemur garnettii ADIR          - ------------------------------------------------------------   -
Tupaia belangeri ADIR            - ------------------------------------------------------------   -
Mus musculus Adir                - ------------------------------------------------------------   -
Rattus norvegicus Adir           - ------------------------------------------------------------   -
Cavia porcellus ADIR             - ------------------------------------------------------------   -
Oryctolagus cuniculus ADIR       - ------------------------------------------------------------   -
Tursiops truncatus ADIR          - ------------------------------------------------------------   -
Bos taurus ADIR                  - ------------------------------------------------------------   -
Equus caballus ADIR              - ------------------------------------------------------------   -
Canis lupus familiaris ADIR      - ------------------------------------------------------------   -
Myotis lucifugus ADIR            - ------------------------------------------------------------   -
Pteropus vampyrus ADIR           - ------------------------------------------------------------   -
Erinaceus europaeus ADIR         - ------------------------------------------------------------   -
Dasypus novemcinctus ADIR        - ------------------------------------------------------------   -
Choloepus hoffmanni ADIR         - ------------------------------------------------------------   -
Loxodonta africana ADIR          - ------------------------------------------------------------   -
```

```
Homo sapiens ADIA              - ------------------------------------------------------------ -
                                         490       500       510       520       530       540

Homo sapiens ADIA              - ------------------------------------------------------------ -
Papio hamadryas ADIA           - ------------------------------------------------------------ -
Mus musculus Adia              - ------------------------------------------------------------ -
Rattus norvegicus Adia         - ------------------------------------------------------------ -
Cavia porcellus ADIA           - ------------------------------------------------------------ -
Ochotona princeps ADIA         - ------------------------------------------------------------ -
Bos taurus ADIA                - ------------------------------------------------------------ -
Equus caballus ADIA            - ------------------------------------------------------------ -
Canis lupus familiaris ADIA    - ------------------------------------------------------------ -
Felis catus ADIA               - ------------------------------------------------------------ -
Myotis lucifugus ADIA          - ------------------------------------------------------------ -
Dasypus novemcinctus ADIA      - ------------------------------------------------------------ -
Loxodonta africana ADIA        - ------------------------------------------------------------ -
Homo sapiens ADIB              - ------------------------------------------------------------ -
Pan troglodytes ADIB           - ------------------------------------------------------------ -
Pongo abelii ADIB              - ------------------------------------------------------------ -
Nomascus leucogenys ADIB       - ------------------------------------------------------------ -
Callithrix jacchus ADIB        - ------------------------------------------------------------ -
Otolemur garnettii ADIB        - ------------------------------------------------------------ -
Mus musculus Adib              - ------------------------------------------------------------ -
Rattus norvegicus Adib         - ------------------------------------------------------------ -
Cavia porcellus ADIB           - ------------------------------------------------------------ -
Oryctolagus cuniculus ADIB     - ------------------------------------------------------------ -
Bos taurus ADIB                - ------------------------------------------------------------ -
Equus caballus ADIB            - ------------------------------------------------------------ -
Canis lupus familiaris ADIB    - ------------------------------------------------------------ -
Myotis lucifugus ADIB          - ------------------------------------------------------------ -
Loxodonta africana ADIB        - ------------------------------------------------------------ -
Homo sapiens ADIC              - ------------------------------------------------------------ -
Macaca mulatta ADIC            - ------------------------------------------------------------ -
Papio hamadryas ADIC           - ------------------------------------------------------------ -
Callithrix jacchus ADIC        - ------------------------------------------------------------ -
Mus musculus Adic              - ------------------------------------------------------------ -
Rattus norvegicus Adic         - ------------------------------------------------------------ -
Cavia porcellus ADIC           - ------------------------------------------------------------ -
Oryctolagus cuniculus ADIC     - ------------------------------------------------------------ -
Equus caballus ADIC            - ------------------------------------------------------------ -
Canis lupus familiaris ADIC    - ------------------------------------------------------------ -
Myotis lucifugus ADIC          - ------------------------------------------------------------ -
Loxodonta africana ADIC        - ------------------------------------------------------------ -
Homo sapiens ADID              - ------------------------------------------------------------ -
Pan troglodytes ADID           - ------------------------------------------------------------ -
Pongo abelii ADID              - ------------------------------------------------------------ -
Nomascus leucogenys ADID       - ------------------------------------------------------------ -
Macaca mulatta ADID            - ------------------------------------------------------------ -
Otolemur garnettii ADID        - ------------------------------------------------------------ -
Mus musculus Adid              - ------------------------------------------------------------ -
Rattus norvegicus Adid         - ------------------------------------------------------------ -
Cavia porcellus ADID           - ------------------------------------------------------------ -
Oryctolagus cuniculus ADID     - ------------------------------------------------------------ -
Bos taurus ADID                - ------------------------------------------------------------ -
Equus caballus ADID            - ------------------------------------------------------------ -
Canis lupus familiaris ADID    - ------------------------------------------------------------ -
Felis catus ADID               - ------------------------------------------------------------ -
Myotis lucifugus ADID          - ------------------------------------------------------------ -
Dasypus novemcinctus ADID      - ------------------------------------------------------------ -
Loxodonta africana ADID        - ------------------------------------------------------------ -
Homo sapiens ADIE              - ------------------------------------------------------------ -
Pan troglodytes ADIE           - ------------------------------------------------------------ -
Pongo abelii ADIE              - ------------------------------------------------------------ -
Papio hamadryas ADIE           - ------------------------------------------------------------ -
Callithrix jacchus ADIE        - ------------------------------------------------------------ -
Otolemur garnettii ADIE        - ------------------------------------------------------------ -
Mus musculus Adie              - ------------------------------------------------------------ -
Rattus norvegicus Adie         - ------------------------------------------------------------ -
Homo sapiens ADIF1             - ------------------------------------------------------------ -
Homo sapiens ADIF2             - ------------------------------------------------------------ -
Pan troglodytes ADIF1          - ------------------------------------------------------------ -
Pongo abelii ADIF1             - ------------------------------------------------------------ -
Nomascus leucogenys ADIF1      - ------------------------------------------------------------ -
Macaca mulatta ADIF1           - ------------------------------------------------------------ -
Papio hamadryas ADIF1          - ------------------------------------------------------------ -
```

```
Microcebus murinus ADIF1        - ------------------------------------------------------------   -
Otolemur garnettii ADIF1        - ------------------------------------------------------------   -
Mus musculus Adif1              - ------------------------------------------------------------   -
Rattus norvegicus Adif1         - ------------------------------------------------------------   -
Cavia porcellus ADIF1           - ------------------------------------------------------------   -
Oryctolagus cuniculus ADIF1     - ------------------------------------------------------------   -
Ochotona princeps ADIF1         - ------------------------------------------------------------   -
Bos taurus ADIF1                - ------------------------------------------------------------   -
Equus caballus ADIF1            - ------------------------------------------------------------   -
Canis lupus familiaris ADIF1    - ------------------------------------------------------------   -
Felis catus ADIF1               - ------------------------------------------------------------   -
Dasypus novemcinctus ADIF1      - ------------------------------------------------------------   -
Loxodonta africana ADIF1        - ------------------------------------------------------------   -
Homo sapiens ADIG               - ------------------------------------------------------------   -
Pan troglodytes ADIG            - ------------------------------------------------------------   -
Pongo abelii ADIG               - ------------------------------------------------------------   -
Callithrix jacchus ADIG         - ------------------------------------------------------------   -
Otolemur garnettii ADIG         - ------------------------------------------------------------   -
Mus musculus Adig               - ------------------------------------------------------------   -
Rattus norvegicus Adig          - ------------------------------------------------------------   -
Cavia porcellus ADIG            - ------------------------------------------------------------   -
Oryctolagus cuniculus ADIG      - ------------------------------------------------------------   -
Tursiops truncatus ADIG         - ------------------------------------------------------------   -
Bos taurus ADIG                 - ------------------------------------------------------------   -
Equus caballus ADIG             - ------------------------------------------------------------   -
Canis lupus familiaris ADIG     - ------------------------------------------------------------   -
Myotis lucifugus ADIG           - ------------------------------------------------------------   -
Homo sapiens ADIH               - ------------------------------------------------------------   -
Pan troglodytes ADIH            - ------------------------------------------------------------   -
Pongo abelii ADIH               - ------------------------------------------------------------   -
Nomascus leucogenys ADIH        - ------------------------------------------------------------   -
Macaca mulatta ADIH             - ------------------------------------------------------------   -
Papio hamadryas ADIH            - ------------------------------------------------------------   -
Mus musculus Adih               - ------------------------------------------------------------   -
Rattus norvegicus Adih          - ------------------------------------------------------------   -
Dipodomys ordii ADIH            - ------------------------------------------------------------   -
Cavia porcellus ADIH            - ------------------------------------------------------------   -
Bos taurus ADIH                 - ------------------------------------------------------------   -
Equus caballus ADIH             - ------------------------------------------------------------   -
Canis lupus familiaris ADIH     - ------------------------------------------------------------   -
Pteropus vampyrus ADIH          - ------------------------------------------------------------   -
Loxodonta africana ADIH         - ------------------------------------------------------------   -
Homo sapiens ADII               - ------------------------------------------------------------   -
Pongo abelii ADII               - ------------------------------------------------------------   -
Macaca mulatta ADII             - ------------------------------------------------------------   -
Mus musculus Adii               - ------------------------------------------------------------   -
Rattus norvegicus Adii          - ------------------------------------------------------------   -
Bos taurus ADII                 - ------------------------------------------------------------   -
Canis lupus familiaris ADII     - ------------------------------------------------------------   -
Dasypus novemcinctus ADII       - ------------------------------------------------------------   -
Homo sapiens ADIJ               - ------------------------------------------------------------   -
Pan troglodytes ADIJ            - ------------------------------------------------------------   -
Papio hamadryas ADIJ            - ------------------------------------------------------------   -
Mus musculus Adij               - ------------------------------------------------------------   -
Rattus norvegicus Adij          - ------------------------------------------------------------   -
Oryctolagus cuniculus ADIJ      - ------------------------------------------------------------   -
Bos taurus ADIJ                 - ------------------------------------------------------------   -
Canis lupus familiaris ADIJ     - ------------------------------------------------------------   -
Loxodonta africana ADIJ         - ------------------------------------------------------------   -
Homo sapiens ADIK               - ------------------------------------------------------------   -
Pan troglodytes ADIK            - ------------------------------------------------------------   -
Gorilla gorilla ADIK            - ------------------------------------------------------------   -
Papio hamadryas ADIK            - ------------------------------------------------------------   -
Callithrix jacchus ADIK         - ------------------------------------------------------------   -
Rattus norvegicus Adik          - ------------------------------------------------------------   -
Cavia porcellus ADIK            - ------------------------------------------------------------   -
Equus caballus ADIK             - ------------------------------------------------------------   -
Canis lupus familiaris ADIK     - ------------------------------------------------------------   -
Dasypus novemcinctus ADIK       - ------------------------------------------------------------   -
Loxodonta africana ADIK         - ------------------------------------------------------------   -
Mus musculus Adil               - ------------------------------------------------------------   -
Rattus norvegicus Adil          - ------------------------------------------------------------   -
Homo sapiens ADIM               - ------------------------------------------------------------   -
Pan troglodytes ADIM            - ------------------------------------------------------------   -
```

Pongo abelii ADIM	-	--	-
Nomascus leucogenys ADIM	-	--	-
Papio hamadryas ADIM	-	--	-
Callithrix jacchus ADIM	-	--	-
Mus musculus Adim	-	--	-
Rattus norvegicus Adim	-	--	-
Cavia porcellus ADIM	-	--	-
Bos taurus ADIM	-	--	-
Equus caballus ADIM	-	--	-
Canis lupus familiaris ADIM	-	--	-
Homo sapiens ADIN	20	TPGPGSSELRSAFSAARTTPLEGTSEMAVTFDKVYVNIGGDFDVATGQFRCRVPGAYFFS	79
Pan troglodytes ADIN	20	TPGPGSSELRSAFSAARTTPLEGTSEMAVTFDKVYVNIGGDFDVATGQFRCRVPGAYYFS	79
Pongo abelii ADIN	20	APGPGSSELRSAFSAARTTPLEGTSEMAVTFDKVYVNIGGDFDAATGQFRCRVPGAYFFS	79
Nomascus leucogenys ADIN	20	ALGPGSSELRSAFSAARTTPLEGTSEMAVTFDKVYVNIGGDFDAATGQFRCRVPGAYFFS	79
Macaca mulatta ADIN	20	APGPGSSELRSAFSAARTTPLEGASEMAVTFDKVYVNIGGDFDAATGQFRCRVPGAYFFS	79
Callithrix jacchus ADIN	20	APGPGSSELRSAFSAARTTPLEGTSEMAVTFDKVYVNIGGDFDAATGQFRCRVPGAYFFS	79
Mus musculus Adin	20	A-GPGSSELRSAFSAARTTPLEGTSEMAVTFDKVYVNIGGDFDAATGRFRCRVPGAYFFS	78
Cavia porcellus ADIN	31	APGPDSTGLRSAFSAVRTTPLEGTSEMAVTFDKVLVNIGSDFDAATGHFRCRVPGAYFFS	90
Tursiops truncatus ADIN	20	ALG--STELRSAFSAARITPLEGTSEMAVTFDKVYVNIGGDFDAATGQFRCRVPGAYFFS	77
Canis lupus familiaris ADIN	20	APG--SAELRSAFSAARTTPLEGTSEMAVTFDKVYVNIGGDFDAATGQFRCRVPGAYFFS	77
Dasypus novemcinctus ADIN	20	APG--SSELRSAFSAARTSSLEGTSEMAVTFDKVFVNIGGDFDAATGQFRCRVPGAYFFS	77
Loxodonta africana ADIN	20	APG--SSELRSAFSAARTSPLEGTSEMAVTFDKVYVNIGGDFDAATGQFRCRVPGAYFFS	77
Homo sapiens ADIO	-	--	-
Pan troglodytes ADIO	-	--	-
Macaca mulatta ADIO	-	--	-
Bos taurus ADIO	-	--	-
Myotis lucifugus ADIO	-	--	-
Homo sapiens ADIP	-	--	-
Otolemur garnettii ADIP	-	--	-
Mus musculus Adip	-	--	-
Cavia porcellus ADIP	-	--	-
Bos taurus ADIP	-	--	-
Equus caballus ADIP	-	--	-
Loxodonta africana ADIP	-	--	-
Homo sapiens ADIQ	-	--	-
Pan troglodytes ADIQ	-	--	-
Pongo abelii ADIQ	-	--	-
Nomascus leucogenys ADIQ	-	--	-
Macaca mulatta ADIQ	-	--	-
Mus musculus Adiq	-	--	-
Rattus norvegicus Adiq	-	--	-
Homo sapiens ADIR	-	--	-
Pan troglodytes ADIR	-	--	-
Pongo abelii ADIR	-	--	-
Nomascus leucogenys ADIR	-	--	-
Macaca mulatta ADIR	-	--	-
Papio hamadryas ADIR	-	--	-
Callithrix jacchus ADIR	-	--	-
Tarsius syrichta ADIR	-	--	-
Otolemur garnettii ADIR	-	--	-
Tupaia belangeri ADIR	-	--	-
Mus musculus Adir	-	--	-
Rattus norvegicus Adir	-	--	-
Cavia porcellus ADIR	-	--	-
Oryctolagus cuniculus ADIR	-	--	-
Tursiops truncatus ADIR	-	--	-
Bos taurus ADIR	-	--	-
Equus caballus ADIR	-	--	-
Canis lupus familiaris ADIR	-	--	-
Myotis lucifugus ADIR	-	--	-
Pteropus vampyrus ADIR	-	--	-
Erinaceus europaeus ADIR	-	--	-
Dasypus novemcinctus ADIR	-	--	-
Choloepus hoffmanni ADIR	-	--	-
Loxodonta africana ADIR	-	--	-

```
Homo sapiens ADIA                - ------------------------------------------------------------   -
                                            550       560       570       580       590       600
Homo sapiens ADIA                - ------------------------------------------------------------   -
Papio hamadryas ADIA             - ------------------------------------------------------------   -
Mus musculus Adia                - ------------------------------------------------------------   -
Rattus norvegicus Adia           - ------------------------------------------------------------   -
Cavia porcellus ADIA             - ------------------------------------------------------------   -
Ochotona princeps ADIA           - ------------------------------------------------------------   -
Bos taurus ADIA                  - ------------------------------------------------------------   -
Equus caballus ADIA              - ------------------------------------------------------------   -
Canis lupus familiaris ADIA      - ------------------------------------------------------------   -
Felis catus ADIA                 - ------------------------------------------------------------   -
Myotis lucifugus ADIA            - ------------------------------------------------------------   -
Dasypus novemcinctus ADIA        - ------------------------------------------------------------   -
Loxodonta africana ADIA          - ------------------------------------------------------------   -
Homo sapiens ADIB                - ------------------------------------------------------------   -
Pan troglodytes ADIB             - ------------------------------------------------------------   -
Pongo abelii ADIB                - ------------------------------------------------------------   -
Nomascus leucogenys ADIB         - ------------------------------------------------------------   -
Callithrix jacchus ADIB          - ------------------------------------------------------------   -
Otolemur garnettii ADIB          - ------------------------------------------------------------   -
Mus musculus Adib                - ------------------------------------------------------------   -
Rattus norvegicus Adib           - ------------------------------------------------------------   -
Cavia porcellus ADIB             - ------------------------------------------------------------   -
Oryctolagus cuniculus ADIB       - ------------------------------------------------------------   -
Bos taurus ADIB                  - ------------------------------------------------------------   -
Equus caballus ADIB              - ------------------------------------------------------------   -
Canis lupus familiaris ADIB      - ------------------------------------------------------------   -
Myotis lucifugus ADIB            - ------------------------------------------------------------   -
Loxodonta africana ADIB          - ------------------------------------------------------------   -
Homo sapiens ADIC                - ------------------------------------------------------------   -
Macaca mulatta ADIC              - ------------------------------------------------------------   -
Papio hamadryas ADIC             - ------------------------------------------------------------   -
Callithrix jacchus ADIC          - ------------------------------------------------------------   -
Mus musculus Adic                - ------------------------------------------------------------   -
Rattus norvegicus Adic           - ------------------------------------------------------------   -
Cavia porcellus ADIC             - ------------------------------------------------------------   -
Oryctolagus cuniculus ADIC       - ------------------------------------------------------------   -
Equus caballus ADIC              - ------------------------------------------------------------   -
Canis lupus familiaris ADIC      - ------------------------------------------------------------   -
Myotis lucifugus ADIC            - ------------------------------------------------------------   -
Loxodonta africana ADIC          - ------------------------------------------------------------   -
Homo sapiens ADID                - ------------------------------------------------------------   -
Pan troglodytes ADID             - ------------------------------------------------------------   -
Pongo abelii ADID                - ------------------------------------------------------------   -
Nomascus leucogenys ADID         - ------------------------------------------------------------   -
Macaca mulatta ADID              - ------------------------------------------------------------   -
Otolemur garnettii ADID          - ------------------------------------------------------------   -
Mus musculus Adid                - ------------------------------------------------------------   -
Rattus norvegicus Adid           - ------------------------------------------------------------   -
Cavia porcellus ADID             - ------------------------------------------------------------   -
Oryctolagus cuniculus ADID       - ------------------------------------------------------------   -
Bos taurus ADID                  - ------------------------------------------------------------   -
Equus caballus ADID              - ------------------------------------------------------------   -
Canis lupus familiaris ADID      - ------------------------------------------------------------   -
Felis catus ADID                 - ------------------------------------------------------------   -
Myotis lucifugus ADID            - ------------------------------------------------------------   -
Dasypus novemcinctus ADID        - ------------------------------------------------------------   -
Loxodonta africana ADID          - ------------------------------------------------------------   -
Homo sapiens ADIE                - ------------------------------------------------------------   -
Pan troglodytes ADIE             - ------------------------------------------------------------   -
Pongo abelii ADIE                - ------------------------------------------------------------   -
Papio hamadryas ADIE             - ------------------------------------------------------------   -
Callithrix jacchus ADIE          - ------------------------------------------------------------   -
Otolemur garnettii ADIE          - ------------------------------------------------------------   -
Mus musculus Adie                - ------------------------------------------------------------   -
Rattus norvegicus Adie           - ------------------------------------------------------------   -
Homo sapiens ADIF1               - ------------------------------------------------------------   -
Homo sapiens ADIF2               - ------------------------------------------------------------   -
Pan troglodytes ADIF1            - ------------------------------------------------------------   -
Pongo abelii ADIF1               - ------------------------------------------------------------   -
Nomascus leucogenys ADIF1        - ------------------------------------------------------------   -
Macaca mulatta ADIF1             - ------------------------------------------------------------   -
Papio hamadryas ADIF1            - ------------------------------------------------------------   -
```

```
Microcebus murinus ADIF1        - ------------------------------------------------------------   -
Otolemur garnettii ADIF1        - ------------------------------------------------------------   -
Mus musculus Adif1              - ------------------------------------------------------------   -
Rattus norvegicus Adif1         - ------------------------------------------------------------   -
Cavia porcellus ADIF1           - ------------------------------------------------------------   -
Oryctolagus cuniculus ADIF1     - ------------------------------------------------------------   -
Ochotona princeps ADIF1         - ------------------------------------------------------------   -
Bos taurus ADIF1                - ------------------------------------------------------------   -
Equus caballus ADIF1            - ------------------------------------------------------------   -
Canis lupus familiaris ADIF1    - ------------------------------------------------------------   -
Felis catus ADIF1               - ------------------------------------------------------------   -
Dasypus novemcinctus ADIF1      - ------------------------------------------------------------   -
Loxodonta africana ADIF1        - ------------------------------------------------------------   -
Homo sapiens ADIG               - ------------------------------------------------------------   -
Pan troglodytes ADIG            - ------------------------------------------------------------   -
Pongo abelii ADIG               - ------------------------------------------------------------   -
Callithrix jacchus ADIG         - ------------------------------------------------------------   -
Otolemur garnettii ADIG         - ------------------------------------------------------------   -
Mus musculus Adig               - ------------------------------------------------------------   -
Rattus norvegicus Adig          - ------------------------------------------------------------   -
Cavia porcellus ADIG            - ------------------------------------------------------------   -
Oryctolagus cuniculus ADIG      - ------------------------------------------------------------   -
Tursiops truncatus ADIG         - ------------------------------------------------------------   -
Bos taurus ADIG                 - ------------------------------------------------------------   -
Equus caballus ADIG             - ------------------------------------------------------------   -
Canis lupus familiaris ADIG     - ------------------------------------------------------------   -
Myotis lucifugus ADIG           - ------------------------------------------------------------   -
Homo sapiens ADIH               - ------------------------------------------------------------   -
Pan troglodytes ADIH            - ------------------------------------------------------------   -
Pongo abelii ADIH               - ------------------------------------------------------------   -
Nomascus leucogenys ADIH        - ------------------------------------------------------------   -
Macaca mulatta ADIH             - ------------------------------------------------------------   -
Papio hamadryas ADIH            - ------------------------------------------------------------   -
Mus musculus Adih               - ------------------------------------------------------------   -
Rattus norvegicus Adih          - ------------------------------------------------------------   -
Dipodomys ordii ADIH            - ------------------------------------------------------------   -
Cavia porcellus ADIH            - ------------------------------------------------------------   -
Bos taurus ADIH                 - ------------------------------------------------------------   -
Equus caballus ADIH             - ------------------------------------------------------------   -
Canis lupus familiaris ADIH     - ------------------------------------------------------------   -
Pteropus vampyrus ADIH          - ------------------------------------------------------------   -
Loxodonta africana ADIH         - ------------------------------------------------------------   -
Homo sapiens ADII               - ------------------------------------------------------------   -
Pongo abelii ADII               - ------------------------------------------------------------   -
Macaca mulatta ADII             - ------------------------------------------------------------   -
Mus musculus Adii               - ------------------------------------------------------------   -
Rattus norvegicus Adii          - ------------------------------------------------------------   -
Bos taurus ADII                 - ------------------------------------------------------------   -
Canis lupus familiaris ADII     - ------------------------------------------------------------   -
Dasypus novemcinctus ADII       - ------------------------------------------------------------   -
Homo sapiens ADIJ               - ------------------------------------------------------------   -
Pan troglodytes ADIJ            - ------------------------------------------------------------   -
Papio hamadryas ADIJ            - ------------------------------------------------------------   -
Mus musculus Adij               - ------------------------------------------------------------   -
Rattus norvegicus Adij          - ------------------------------------------------------------   -
Oryctolagus cuniculus ADIJ      - ------------------------------------------------------------   -
Bos taurus ADIJ                 - ------------------------------------------------------------   -
Canis lupus familiaris ADIJ     - ------------------------------------------------------------   -
Loxodonta africana ADIJ         - ------------------------------------------------------------   -
Homo sapiens ADIK               - ------------------------------------------------------------   -
Pan troglodytes ADIK            - ------------------------------------------------------------   -
Gorilla gorilla ADIK            - ------------------------------------------------------------   -
Papio hamadryas ADIK            - ------------------------------------------------------------   -
Callithrix jacchus ADIK         - ------------------------------------------------------------   -
Rattus norvegicus Adik          - ------------------------------------------------------------   -
Cavia porcellus ADIK            - ------------------------------------------------------------   -
Equus caballus ADIK             - ------------------------------------------------------------   -
Canis lupus familiaris ADIK     - ------------------------------------------------------------   -
Dasypus novemcinctus ADIK       - ------------------------------------------------------------   -
Loxodonta africana ADIK         - ------------------------------------------------------------   -
Mus musculus Adil               - ------------------------------------------------------------   -
Rattus norvegicus Adil          - ------------------------------------------------------------   -
Homo sapiens ADIM               - ------------------------------------------------------------   -
Pan troglodytes ADIM            - ------------------------------------------------------------   -
```

Pongo abelii ADIM	-	--	-
Nomascus leucogenys ADIM	-	--	-
Papio hamadryas ADIM	-	--	-
Callithrix jacchus ADIM	-	--	-
Mus musculus Adim	-	--	-
Rattus norvegicus Adim	-	--	-
Cavia porcellus ADIM	-	--	-
Bos taurus ADIM	-	--	-
Equus caballus ADIM	-	--	-
Canis lupus familiaris ADIM	-	--	-
Homo sapiens ADIN	80	FTAGKAPHKSLSVMLVRNRDEVQALAFDEQRRPGARRAASQSAMLQLDYGDTVWLRLHGA	139
Pan troglodytes ADIN	80	FTAGKAPHKSLSVMLVRNRDEVQALAFDEQRRPGARRAASQSAMLQLDYGDTVWLRLHGA	139
Pongo abelii ADIN	80	FTAGKAPHKSLSVMLVRNRDEVQALAFDEQRRPGARRAASQSAMLQLDYGDTVWLRLHGA	139
Nomascus leucogenys ADIN	80	FTAGKAPHKSLSVMLVRNRDEVQALAFDEQRRPGARRAASQSAMLQLDYGDTVWLRLHGA	139
Macaca mulatta ADIN	80	FTVGKAPHKSLSVMLVRNHDEVQALAFDEQRRPSARRAASQSAMLQLDYGDTVWLRLHGS	139
Callithrix jacchus ADIN	80	FTAGKAPHKSLSVMLVRNRDEVQALAFDEQRRPSARRAASQSAMLQLDYGDTVWLRLHGA	139
Mus musculus Adin	79	FTAGKAPHKSLSVMLVRNRDEVQALAFDEQRRPGARRAASQSAMLQLDYGDTVWLRLHGA	138
Cavia porcellus ADIN	91	FTAGKAPHKSLSVMLVRNRDEVQALAFDEQRRPGTRRAASQSAMLQLDYGDTVWLRLHGA	150
Tursiops truncatus ADIN	78	FTAGKAPHKSLSVMLVRNRDEVQALAFDEQRRPATRHAASQSAMLQLDYGDTVWLRLHGA	137
Canis lupus familiaris ADIN	78	FTAGKAPHKSLSVMLVRNRDEVQALAFDEQRRPGARRAASQSAMLQLDYGDTVWLRLHGA	137
Dasypus novemcinctus ADIN	78	FTAGKAPHKSLSVMLVRNSDEVQTLAFDEQRRPGARRAASQSAMLQLDYGDTVWLRLHGA	137
Loxodonta africana ADIN	78	FTAGKAPHKSLSVMLVRNRNEVQALAFDEQRRPGARRAASQSAMLQLDYGDTVWLRLHGA	137
Homo sapiens ADIO	-	--	-
Pan troglodytes ADIO	-	--	-
Macaca mulatta ADIO	-	--	-
Bos taurus ADIO	-	--	-
Myotis lucifugus ADIO	-	--	-
Homo sapiens ADIP	-	--	-
Otolemur garnettii ADIP	-	--	-
Mus musculus Adip	-	--	-
Cavia porcellus ADIP	-	--	-
Bos taurus ADIP	-	--	-
Equus caballus ADIP	-	--	-
Loxodonta africana ADIP	-	--	-
Homo sapiens ADIQ	-	--	-
Pan troglodytes ADIQ	-	--	-
Pongo abelii ADIQ	-	--	-
Nomascus leucogenys ADIQ	-	--	-
Macaca mulatta ADIQ	-	--	-
Mus musculus Adiq	-	--	-
Rattus norvegicus Adiq	-	--	-
Homo sapiens ADIR	-	--	-
Pan troglodytes ADIR	-	--	-
Pongo abelii ADIR	-	--	-
Nomascus leucogenys ADIR	-	--	-
Macaca mulatta ADIR	-	--	-
Papio hamadryas ADIR	-	--	-
Callithrix jacchus ADIR	-	--	-
Tarsius syrichta ADIR	-	--	-
Otolemur garnettii ADIR	-	--	-
Tupaia belangeri ADIR	-	--	-
Mus musculus Adir	-	--	-
Rattus norvegicus Adir	-	--	-
Cavia porcellus ADIR	-	--	-
Oryctolagus cuniculus ADIR	-	--	-
Tursiops truncatus ADIR	-	--	-
Bos taurus ADIR	-	--	-
Equus caballus ADIR	-	--	-
Canis lupus familiaris ADIR	-	--	-
Myotis lucifugus ADIR	-	--	-
Pteropus vampyrus ADIR	-	--	-
Erinaceus europaeus ADIR	-	--	-
Dasypus novemcinctus ADIR	-	--	-
Choloepus hoffmanni ADIR	-	--	-
Loxodonta africana ADIR	-	--	-

```
Homo sapiens ADIA                -  ------------------------------------------------------------  -
                                             610       620       630       640       650       660

Homo sapiens ADIA                -  ------------------------------------------------------------  -
Papio hamadryas ADIA             -  ------------------------------------------------------------  -
Mus musculus Adia                -  ------------------------------------------------------------  -
Rattus norvegicus Adia           -  ------------------------------------------------------------  -
Cavia porcellus ADIA             -  ------------------------------------------------------------  -
Ochotona princeps ADIA           -  ------------------------------------------------------------  -
Bos taurus ADIA                  -  ------------------------------------------------------------  -
Equus caballus ADIA              -  ------------------------------------------------------------  -
Canis lupus familiaris ADIA      -  ------------------------------------------------------------  -
Felis catus ADIA                 -  ------------------------------------------------------------  -
Myotis lucifugus ADIA            -  ------------------------------------------------------------  -
Dasypus novemcinctus ADIA        -  ------------------------------------------------------------  -
Loxodonta africana ADIA          -  ------------------------------------------------------------  -
Homo sapiens ADIB                -  ------------------------------------------------------------  -
Pan troglodytes ADIB             -  ------------------------------------------------------------  -
Pongo abelii ADIB                -  ------------------------------------------------------------  -
Nomascus leucogenys ADIB         -  ------------------------------------------------------------  -
Callithrix jacchus ADIB          -  ------------------------------------------------------------  -
Otolemur garnettii ADIB          -  ------------------------------------------------------------  -
Mus musculus Adib                -  ------------------------------------------------------------  -
Rattus norvegicus Adib           -  ------------------------------------------------------------  -
Cavia porcellus ADIB             -  ------------------------------------------------------------  -
Oryctolagus cuniculus ADIB       -  ------------------------------------------------------------  -
Bos taurus ADIB                  -  ------------------------------------------------------------  -
Equus caballus ADIB              -  ------------------------------------------------------------  -
Canis lupus familiaris ADIB      -  ------------------------------------------------------------  -
Myotis lucifugus ADIB            -  ------------------------------------------------------------  -
Loxodonta africana ADIB          -  ------------------------------------------------------------  -
Homo sapiens ADIC                -  ------------------------------------------------------------  -
Macaca mulatta ADIC              -  ------------------------------------------------------------  -
Papio hamadryas ADIC             -  ------------------------------------------------------------  -
Callithrix jacchus ADIC          -  ------------------------------------------------------------  -
Mus musculus Adic                -  ------------------------------------------------------------  -
Rattus norvegicus Adic           -  ------------------------------------------------------------  -
Cavia porcellus ADIC             -  ------------------------------------------------------------  -
Oryctolagus cuniculus ADIC       -  ------------------------------------------------------------  -
Equus caballus ADIC              -  ------------------------------------------------------------  -
Canis lupus familiaris ADIC      -  ------------------------------------------------------------  -
Myotis lucifugus ADIC            -  ------------------------------------------------------------  -
Loxodonta africana ADIC          -  ------------------------------------------------------------  -
Homo sapiens ADID                -  ------------------------------------------------------------  -
Pan troglodytes ADID             -  ------------------------------------------------------------  -
Pongo abelii ADID                -  ------------------------------------------------------------  -
Nomascus leucogenys ADID         -  ------------------------------------------------------------  -
Macaca mulatta ADID              -  ------------------------------------------------------------  -
Otolemur garnettii ADID          -  ------------------------------------------------------------  -
Mus musculus Adid                -  ------------------------------------------------------------  -
Rattus norvegicus Adid           -  ------------------------------------------------------------  -
Cavia porcellus ADID             -  ------------------------------------------------------------  -
Oryctolagus cuniculus ADID       -  ------------------------------------------------------------  -
Bos taurus ADID                  -  ------------------------------------------------------------  -
Equus caballus ADID              -  ------------------------------------------------------------  -
Canis lupus familiaris ADID      -  ------------------------------------------------------------  -
Felis catus ADID                 -  ------------------------------------------------------------  -
Myotis lucifugus ADID            -  ------------------------------------------------------------  -
Dasypus novemcinctus ADID        -  ------------------------------------------------------------  -
Loxodonta africana ADID          -  ------------------------------------------------------------  -
Homo sapiens ADIE                -  ------------------------------------------------------------  -
Pan troglodytes ADIE             -  ------------------------------------------------------------  -
Pongo abelii ADIE                -  ------------------------------------------------------------  -
Papio hamadryas ADIE             -  ------------------------------------------------------------  -
Callithrix jacchus ADIE          -  ------------------------------------------------------------  -
Otolemur garnettii ADIE          -  ------------------------------------------------------------  -
Mus musculus Adie                -  ------------------------------------------------------------  -
Rattus norvegicus Adie           -  ------------------------------------------------------------  -
Homo sapiens ADIF1               -  ------------------------------------------------------------  -
Homo sapiens ADIF2               -  ------------------------------------------------------------  -
Pan troglodytes ADIF1            -  ------------------------------------------------------------  -
Pongo abelii ADIF1               -  ------------------------------------------------------------  -
Nomascus leucogenys ADIF1        -  ------------------------------------------------------------  -
Macaca mulatta ADIF1             -  ------------------------------------------------------------  -
Papio hamadryas ADIF1            -  ------------------------------------------------------------  -
```

Microcebus murinus ADIF1	-	--	-
Otolemur garnettii ADIF1	-	--	-
Mus musculus Adif1	-	--	-
Rattus norvegicus Adif1	-	--	-
Cavia porcellus ADIF1	-	--	-
Oryctolagus cuniculus ADIF1	-	--	-
Ochotona princeps ADIF1	-	--	-
Bos taurus ADIF1	-	--	-
Equus caballus ADIF1	-	--	-
Canis lupus familiaris ADIF1	-	--	-
Felis catus ADIF1	-	--	-
Dasypus novemcinctus ADIF1	-	--	-
Loxodonta africana ADIF1	-	--	-
Homo sapiens ADIG	-	--	-
Pan troglodytes ADIG	-	--	-
Pongo abelii ADIG	-	--	-
Callithrix jacchus ADIG	-	--	-
Otolemur garnettii ADIG	-	--	-
Mus musculus Adig	-	--	-
Rattus norvegicus Adig	-	--	-
Cavia porcellus ADIG	-	--	-
Oryctolagus cuniculus ADIG	-	--	-
Tursiops truncatus ADIG	-	--	-
Bos taurus ADIG	-	--	-
Equus caballus ADIG	-	--	-
Canis lupus familiaris ADIG	-	--	-
Myotis lucifugus ADIG	-	--	-
Homo sapiens ADIH	-	--	-
Pan troglodytes ADIH	-	--	-
Pongo abelii ADIH	-	--	-
Nomascus leucogenys ADIH	-	--	-
Macaca mulatta ADIH	-	--	-
Papio hamadryas ADIH	-	--	-
Mus musculus Adih	-	--	-
Rattus norvegicus Adih	-	--	-
Dipodomys ordii ADIH	-	--	-
Cavia porcellus ADIH	-	--	-
Bos taurus ADIH	-	--	-
Equus caballus ADIH	-	--	-
Canis lupus familiaris ADIH	-	--	-
Pteropus vampyrus ADIH	-	--	-
Loxodonta africana ADIH	-	--	-
Homo sapiens ADII	121	-----------------------------LTAGTASGVGVVGGGAGVGGDSEGEVTSALS	151
Pongo abelii ADII	121	-----------------------------LTAGTASGVGVVGGGAGVGGDSEGEVTSALS	151
Macaca mulatta ADII	121	-----------------------------LTAGTAGGVGVVGGGAGGAGDSEGEVTSALS	151
Mus musculus Adii	121	-----------------------------LTTSAAGGVGVVSGGTGGGGDTEGEVTSALS	151
Rattus norvegicus Adii	121	-----------------------------LTTSAAGGVGVVGGGTGGGGDTEGEVTSALS	151
Bos taurus ADII	121	-----------------------------LTAGAAGGVGVVGGGTGGGGDSEGEVTGALS	151
Canis lupus familiaris ADII	121	-----------------------------LAAGAAGGVGVAGGGAGGGGDSEGEVTGALS	151
Dasypus novemcinctus ADII	121	-----------------------------LTAGAAGGVGVAGGGAPGGGDSEGEVTGALG	151
Homo sapiens ADIJ	112	--AAGAIS	117
Pan troglodytes ADIJ	112	--AAGAIS	117
Papio hamadryas ADIJ	112	--AAGAIS	117
Mus musculus Adij	112	--AAGAIS	117
Rattus norvegicus Adij	112	--AAGAIS	117
Oryctolagus cuniculus ADIJ	112	--AAGAIS	117
Bos taurus ADIJ	112	--AAGAIS	117
Canis lupus familiaris ADIJ	112	--AAGAIS	117
Loxodonta africana ADIJ	112	--AAGAIS	117
Homo sapiens ADIK	97	---PGGVAP-	102
Pan troglodytes ADIK	97	---PGGVAP-	102
Gorilla gorilla ADIK	97	---PGGVAP-	102
Papio hamadryas ADIK	97	---PGGVAP-	102
Callithrix jacchus ADIK	97	---PGGVAP-	102
Rattus norvegicus Adik	97	---PGGAAP-	102
Cavia porcellus ADIK	97	---PGGAAP-	102
Equus caballus ADIK	97	---PGGVAP-	102
Canis lupus familiaris ADIK	97	---PGGVAP-	102
Dasypus novemcinctus ADIK	97	---PGGAAP-	102
Loxodonta africana ADIK	97	---PGGVAP-	102
Mus musculus Adil	118	---AIS	120
Rattus norvegicus Adil	118	---AIS	120
Homo sapiens ADIM	-	--	-
Pan troglodytes ADIM	-	--	-

Pongo abelii ADIM	-	--	-
Nomascus leucogenys ADIM	-	--	-
Papio hamadryas ADIM	-	--	-
Callithrix jacchus ADIM	-	--	-
Mus musculus Adim	-	--	-
Rattus norvegicus Adim	-	--	-
Cavia porcellus ADIM	-	--	-
Bos taurus ADIM	-	--	-
Equus caballus ADIM	-	--	-
Canis lupus familiaris ADIM	-	--	-
Homo sapiens ADIN	140	PQYALGAPGATFSGYLVYADADA--DAPA--------------------------RGPPA	171
Pan troglodytes ADIN	140	PQYALGAPGATFSGYLVYADADA--DAPA--------------------------RGPPA	171
Pongo abelii ADIN	140	PQYALGAPGATFSGYLVYADADA--DAPA--------------------------RGPPA	171
Nomascus leucogenys ADIN	140	PQYALGAPGATFSGYLVYADADA--DAPA-------------------------------	166
Macaca mulatta ADIN	140	PQYALGAPGATFSGYLVYADADADADAPA--------------------------RGPPA	173
Callithrix jacchus ADIN	140	PQYALGAPGATFSGYLVYADADA--DAPA--------------------------RGPPA	171
Mus musculus Adin	139	PQYALGAPGATFSGYLVYADADA--DAPA--------------------------RG-PA	169
Cavia porcellus ADIN	151	PQYALGSPGATFSGYLVYADADA--PAPA--------------------------PG-PA	181
Tursiops truncatus ADIN	138	QQYALGAPGATFSGYLVYADAD----APA--------------------------RGPPA	167
Canis lupus familiaris ADIN	138	PQYALGAPGATFSGYLVYADAD----APA--------------------------RGPPA	167
Dasypus novemcinctus ADIN	138	PQYALGAPGATFSGYLVYADAD----APA--------------------------RGPPA	167
Loxodonta africana ADIN	138	PQYALGAPGATFSGYLVYADAD----APA--------------------------RSPQG	167
Homo sapiens ADIO	-	--	-
Pan troglodytes ADIO	-	--	-
Macaca mulatta ADIO	-	--	-
Bos taurus ADIO	-	--	-
Myotis lucifugus ADIO	-	--	-
Homo sapiens ADIP	-	--	-
Otolemur garnettii ADIP	-	--	-
Mus musculus Adip	-	--	-
Cavia porcellus ADIP	-	--	-
Bos taurus ADIP	-	--	-
Equus caballus ADIP	-	--	-
Loxodonta africana ADIP	-	--	-
Homo sapiens ADIQ	-	--	-
Pan troglodytes ADIQ	-	--	-
Pongo abelii ADIQ	-	--	-
Nomascus leucogenys ADIQ	-	--	-
Macaca mulatta ADIQ	-	--	-
Mus musculus Adiq	-	--	-
Rattus norvegicus Adiq	-	--	-
Homo sapiens ADIR	16	--LPVHVYPL	23
Pan troglodytes ADIR	16	--LPVHVYPL	23
Pongo abelii ADIR	16	--LPVHVYPL	23
Nomascus leucogenys ADIR	16	--LPVHVYPL	23
Macaca mulatta ADIR	16	--LPVHVYPL	23
Papio hamadryas ADIR	16	--LPVHVYPL	23
Callithrix jacchus ADIR	16	--LPVHVYPL	23
Tarsius syrichta ADIR	16	--LPVHVYPL	23
Otolemur garnettii ADIR	16	--LPVHVYPL	23
Tupaia belangeri ADIR	16	--LPVHVYPL	23
Mus musculus Adir	16	--LPVHIYPL	23
Rattus norvegicus Adir	16	--LPVHVYPL	23
Cavia porcellus ADIR	16	--LPVHVYPL	23
Oryctolagus cuniculus ADIR	16	--LPVHVYPL	23
Tursiops truncatus ADIR	16	--LPVHVYPL	23
Bos taurus ADIR	16	--LPVHVYPL	23
Equus caballus ADIR	16	--LPVHVYPL	23
Canis lupus familiaris ADIR	16	--LPVHVYPL	23
Myotis lucifugus ADIR	16	--LPVHVYPL	23
Pteropus vampyrus ADIR	16	--LPVHVYPL	23
Erinaceus europaeus ADIR	-	--	-
Dasypus novemcinctus ADIR	16	--LPVHVYPL	23
Choloepus hoffmanni ADIR	16	--LPVHVYPL	23
Loxodonta africana ADIR	16	--LPVHVFPL	23

```
Homo sapiens ADIA               117 ----K---ATQKIAFSATRTINVP-----L-RRDQT-IRFDHVITN-MN-NNYEPRSGKF 160
                                                670       680       690       700       710       720
Homo sapiens ADIA               117 ----K---ATQKIAFSATRTINVP-----L-RRDQT-IRFDHVITN-MN-NNYEPRSGKF 160
Papio hamadryas ADIA            117 ----K---ATQKIAFSATRTINTP-----L-RRDQT-IRFDHVITN-MN-NNYEPRSGKF 160
Mus musculus Adia               144 ----G---ATQKVAFSALRTINSP-----L-RPNQV-IRFEKVITN-AN-ENYEPRNGKF 187
Rattus norvegicus Adia          115 ----K---ATQKVAFSALRTVNSA-----L-RPNQA-IRFEKVITN-VN-DNYEPRSGKF 158
Cavia porcellus ADIA            115 ----K---ATQKVAFSALKTLSAP-----V-RAKQA-IRFDSVITN-VN-KNYEPRSGKF 158
Ochotona princeps ADIA          115 ----K---ATQKIVFSVLKTTATP-----V-RRDLP-VRFEHTITN-EN-NNYYSRSGKF 158
Bos taurus ADIA                 111 ----K---ATQKIAFSASRTINHH-----Q-RQGQP-IRFDHVITN-AN-ENYQARSSKF 154
Equus caballus ADIA             116 ----K---TTQKIGFSATRTVNIP-----L-RKDQV-IRFDHLITN-VN-DNYQPRSGKF 159
Canis lupus familiaris ADIA     114 ----K---ATQKIAFSAMRTINIP-----L-RRDQT-IRFDHIVTN-EN-RNYEPRSGKF 157
Felis catus ADIA                118 ----K---ATQKIAFCAKRTINSA-----L-RRDQA-IRFDQVITN-LN-NNYESRSGKF 161
Myotis lucifugus ADIA           115 ----K---ATQKVAFSALRKINTP-----L-RKDQV-IRFDHVITN-VN-NNYESRLGKF 158
Dasypus novemcinctus ADIA       112 ----K---ATQKIAFSATRTVTTP-----L-RRDQT-IRFDHVITN-EN-NNYEPRSGKF 155
Loxodonta africana ADIA         115 ----K---ATQKIAFSALRNINVP-----L-RRDQA-IRFDHVITN-EN-NNYEPRSGKF 158
Homo sapiens ADIB               115 ----K---QKFQSVFTVTRQTHQP-----P-APNSL-IRFNAVLTN-PQ-GDYDTSTGKF 158
Pan troglodytes ADIB            115 ----K---QKFQSVFTVARQTHQP-----P-APNSL-IRFNAVLTN-PQ-GDYDTSTGKF 158
Pongo abelii ADIB               115 ----K---QKFQSVFTVTRQTHQP-----P-APNSL-IRFNAVLTN-PQ-GDYDTSTGKF 158
Nomascus leucogenys ADIB        116 ----K---QKFQSVFTVTRQTHQP-----P-APNSL-IRFNAVLTN-PQ-GDYDTSTGKF 159
Callithrix jacchus ADIB         114 ----K---QKYQSVFTVTRQTSQF-----P-AANGL-IIFNTAVTN-PQ-GDYDTSTGKF 157
Otolemur garnettii ADIB         113 ----K---QKHQSVFTVTRQTAQY-----P-EPNSL-VKFNTVITN-PQ-GDYNTGTGKF 156
Mus musculus Adib               146 ----K---QKHQSVFTVTRQTTQY-----P-EANAL-VRFNSVVTN-PQ-GHYNPSTGKF 189
Rattus norvegicus Adib          138 ----K---QKHQSVFTVTRQTAQY-----P-AANGL-VKFNSAITN-PQ-GDYNTNTGKF 181
Cavia porcellus ADIB            162 ----K---QKHQSVFTVARQTEQH-----P-TANGL-VRFNAAITN-PQ-GDYDRDSGRF 205
Oryctolagus cuniculus ADIB      168 ----K---QKHQSVFTVTRQTAQF-----P-ESNSL-VRFNTAITN-PQ-GDYDTSTGKF 211
Bos taurus ADIB                 113 ----K---QKHQSVFSVTRQTVQF-----P-EANSL-VKFNRVITN-PQ-GHYDKDSGKF 156
Equus caballus ADIB             133 ----K---QKHQSVFTVTRQTDQY-----P-AANSL-VKFNTVVTN-PQ-GHYDTSTGKF 176
Canis lupus familiaris ADIB     115 ----K---QKHQSVFTVTRQTAQY-----P-LANNL-VKFNTVITN-PQ-GDYDTSTGKF 158
Myotis lucifugus ADIB           115 ----K---QKHQSVFTVTRQTAEH-----P-TSNSL-VKFNTVITN-PQ-GDYNTSTGKF 158
Loxodonta africana ADIB         113 ----K---QKHQSVFTVTRQTVQY-----P-EPNGL-VKFNGVITN-PQ-GHYDTSTGKF 156
Homo sapiens ADIC               110 ----K---DQPRPAFSAIRRNP-------P-MGGNV-VIFDTVITN-QE-EPYQNHSGRF 151
Macaca mulatta ADIC             110 ----K---DQPRPAFSAIRRNP-------P-MGGNV-VIFDMVITN-QE-EPYQNHSGRF 151
Papio hamadryas ADIC            110 ----K---DQPRPAFSAIRRNP-------P-MGGNV-VIFDTVITN-QE-EPYQNHSGRF 151
Callithrix jacchus ADIC         110 ----R---DQPRPAFSAIRRNP-------P-MGGNV-VIFDTVLTN-QE-GPYQNNSGRF 151
Mus musculus Adic               110 ----R---DQPRPAFSAIRQNP-------M-TLGNV-VIFDKVLTN-QE-SPYQNHTGRF 151
Rattus norvegicus Adic          122 ----R---DQPRPAFSAIRQNP-------P-TYGNV-VVFDKVLTN-QE-NPYQNRTGHF 163
Cavia porcellus ADIC            109 ----R---DQPRPAFSAVRRNPQ------P-KRDKV-VVFDTVIIN-QE-GPYRNDTGHF 151
Oryctolagus cuniculus ADIC      151 ----R---DQPRPAFSAVRRNP-------P-SGGNV-VIFDTVITN-QE-EPYQNHTGHF 192
Equus caballus ADIC            143 ----K---DQPRPAFSAVRRNA-------P-LDSNM-VIFDEVITN-QE-GSYDSRTSHF 184
Canis lupus familiaris ADIC     129 ----R---DQPRPAFSAIRRNP-------P-TGGNV-VIFDTVITN-QE-GPYQNHSGRF 170
Myotis lucifugus ADIC           110 ----N---DQPRPAFSAVRRNP-------P-TGGNV-VIFDTVITN-EE-GRYQSHSGRF 151
Loxodonta africana ADIC         125 ----K---DQPRPAFSAVRRNP-------P-MGGNV-VIFDTVLTN-QE-GPYQNHSGLF 166
Homo sapiens ADID               139 ----A---YVYRSAFSVGLE--TY-----V-TIPNMPIRFTKIFYN-QQ-NHYDGSTGKF 181
Pan troglodytes ADID            139 ----A---YVYRSAFSVGLE--TY-----I-TVPNMPIRFTKIFYN-QQ-NHYDGSTGKF 181
Pongo abelii ADID               108 ----A---YVYRSAFSVGLE--TY-----V-TVPNMPIRFTKIFYN-QQ-NHYDGSTGKF 150
Nomascus leucogenys ADID        138 ----A---YVYRSAFSVGLE--TY-----V-TVPNMPIRFTKIFYN-QQ-NHYDGSTGKF 180
Macaca mulatta ADID             137 ----A---YVYRSAFSVGLE--TY-----V-TVPNMPIRFTKIFYN-QQ-NHYDGSTGKF 179
Otolemur garnettii ADID         108 ----A---YVYRSAFSVGLE--NR-----V-TVPNVPIRFTKIFYN-QQ-NHYDGTTGKF 150
Mus musculus Adid               122 ----A---YVYRSAFSVGLE--TR-----V-TVPNVPIRFTKIFYN-QQ-NHYDGSTGKF 164
Rattus norvegicus Adid          108 ----A---YMYHSAFSVGLE--TR-----V-TVPNVPIRFTKIFYN-QQ-NHYDGSTGKF 150
Cavia porcellus ADID            150 ----A---FVYRSAFSVGLE--AY-----V-TVPNVPIRFTKIFYN-QQ-NHYDGTTGKF 192
Oryctolagus cuniculus ADID      118 ----A---YVYRSAFSVGLE--GR-----V-TIPNVPIRFTKIFYN-QQ-NHYDSTTGKF 160
Bos taurus ADID                 103 ----A---YVYRSAFSVGLE--RQ-----V-TVPNVPIRFTKIFYN-QQ-NHYDGTTGKF 145
Equus caballus ADID             165 ----S---YVYRSAFSVGLE--TR-----V-TVPNVPIRFTKIFYN-QQ-NHYDGSTGKF 207
Canis lupus familiaris ADID     117 ----A---YVHRSAFSVGLE--SR-----I-TVPNVPIRFTKIFYN-LQ-NHYDGTTGKF 159
Felis catus ADID                108 ----A---YVYRSAFSVGLE--SR-----V-TVPNVPIRFTKIFYN-QQ-NHYDVTTGKF 150
Myotis lucifugus ADID           117 ----A---YVYRSAFSVGLE--TR-----V-TVPNVPIRFTKIFYN-QQ-NHYDDTTGKF 159
Dasypus novemcinctus ADID       107 ----A---YVYRSAFSVGLE--AR-----V-TVPNVPIRFTKIFYN-QQ-SHYDDTTGKF 149
Loxodonta africana ADID         108 ----A---YSYRSAFSVGLE--AR-----V-TVPNVPIRFTKIFYN-QQ-NHYDGSTGKF 150
Homo sapiens ADIE                99 ----S---VPPRSAFSAKRSESRV-----P-PPSDAPLPFDRVLVN-EQ-GHYDAVTGKF 143
Pan troglodytes ADIE             99 ----S---VPPRSAFSAKRSESRV-----P-PPSDAPLPFDRVLVN-EQ-GHYDAVTGKF 143
Pongo abelii ADIE                99 ----S---VPPRSAFSAKRSESRV-----P-PPSDAPLPFDRVLVN-EQ-GHYDAVTGKF 143
Papio hamadryas ADIE             99 ----S---VPPRSAFSAKRSESRL-----P-PPSDAPLPFDRVLVN-EQ-GHYDAVTGKF 143
Callithrix jacchus ADIE          99 ----S---VPPRSAFSAKRSESRV-----P-PPSDAPLPFDRVLVN-EQ-GHYDAVTGKF 143
Otolemur garnettii ADIE          99 ----S---VPPRSAFSAKRSESRV-----P-PLSDAPLPFDRVLVN-EQ-GHYDAATGKF 143
Mus musculus Adie                99 ----S---VPPRSAFSAKRSESRV-----P-PPADTPLPFDRVLLN-EQ-GHYDPTTGKF 143
Rattus norvegicus Adie           99 ----S---VPPRSAFSAKRSESRV-----P-PPADTPLPFDRVLLN-EQ-GHYDATTGKF 143
Homo sapiens ADIF1              197 ----L---VLPKSAFTVGLTVLSK-----F-PSSDMPIKFDKILYN-EF-NHYDTAAGKF 241
Homo sapiens ADIF2              197 ----L---VLPKSAFTVGLTVLSK-----F-PSSDVPIKFDKILYN-EF-NHYDTAAGKF 241
Pan troglodytes ADIF1           197 ----L---VLPKSAFTVGLTVLSK-----F-PSSDVPIKFDKILYN-EF-NHYDTAAGKF 241
Pongo abelii ADIF1              197 ----L---VLPKSAFTVGLTVLSK-----F-PSSDVPIKFDKILYN-EF-NHYDIATGKF 241
Nomascus leucogenys ADIF1       197 ----L---VLPKSAFTVGLTVLSK-----F-PSSDVPIKFDKILYN-EF-NHYDIATGKF 241
Macaca mulatta ADIF1            197 ----L---VLPKSAFTVGLTVLSK-----F-PSSDVPIKFDKILYN-EF-NHYDIATGKF 241
Papio hamadryas ADIF1           197 ----L---VLPKSAFTVGLTVLSK-----F-PSSDVPIKFDKILYN-EF-NHYDIATGKF 241
```

***Microcebus murinus* ADIF1**	197	----P---ALPKSAFTVGLTVLSK-----F-PSSDVPIKFDKILYN-EF-NHYDVATGKF	241
***Otolemur garnettii* ADIF1**	197	----P---VLPRSAFTVGLTVLSK-----F-PSSDVPIKFDKILYN-DF-NHYDIAKGKF	241
***Mus musculus* Adif1**	226	----P---LVPKSAFTVGLTVISK-----F-PPPDAPIKFDKILYN-EL-NHYNVATGKF	270
***Rattus norvegicus* Adif1**	213	----P---MVPKSAFTVGLTVISK-----F-PPPDVPIKFDKILYN-EQ-NHYNVATGKF	257
***Cavia porcellus* ADIF1**	197	----P---VLPKSAFTVGLTVLNK-----F-PASDAPIKFDKILYN-EF-NHYDVATGKF	241
***Oryctolagus cuniculus* ADIF1**	197	----P---VLPRSAFTVGLTVLSK-----F-PASDVPIKFDRILYN-EL-NHYNVATGKF	241
***Ochotona princeps* ADIF1**	197	----P---VTPKSAFTVGLTVLSK-----F-PDADVPIKFDKILYN-DL-NHYNVATGKF	241
***Bos taurus* ADIF1**	197	----P---ALPKSAFTVGLTVLSK-----F-PPSDTPIKFDRILYN-EF-GHYDVATGKF	241
***Equus caballus* ADIF1**	197	----P---VLPKSAFTVGLTVLSK-----F-PSSDVPIKFDKILYN-EF-NHFDIATGKF	241
***Canis lupus familiaris* ADIF1**	197	----P---VLPKSAFTVGLTVLSK-----F-PTSDVPIKFDKILYN-EF-NHYDVATGKF	241
***Felis catus* ADIF1**	197	----P---VLPKSAFTVGLTVLSK-----F-PASDAPIKFDKILYN-EF-NHYDVATGKF	241
***Dasypus novemcinctus* ADIF1**	198	----P---VPPKSAFTVGLTVLSK-----F-PSSDVPIKFDRILFN-EF-NHFDIATGKF	242
***Loxodonta africana* ADIF1**	197	----P---VLPKSAFTVGLTVLSK-----F-PSSDLPIKFDRILYN-EF-HHYNVATGKF	241
***Homo sapiens* ADIG**	194	----RCGSIVLKSAFSVGIT-TS------Y-PEERLPIIFNKVLFN-EG-EHYNPATGKF	239
***Pan troglodytes* ADIG**	194	----RCGSIVLKSAFSVGIT-TS------Y-PEERLPIIFNKVLFN-EG-EHYNPATGKF	239
***Pongo abelii* ADIG**	194	----RCGSIVLKSAFSVGIT-TS------Y-PEERLPIIFNKVLFN-EG-EHYNPATGKF	239
***Callithrix jacchus* ADIG**	180	----RCGSIVLKSAFSVGIT-TS------Y-PEERLPIIFNKVLFN-EG-EHYNPATGKF	225
***Otolemur garnettii* ADIG**	140	----RCGSIVLKSAFSVGIT-TS------Y-PEERLPIIFNKVLFN-EG-EHYNPATGKF	185
***Mus musculus* Adig**	182	----RCGSIVLKSAFSVGIT-TS------Y-PEERLPIIFNKVLFN-EG-EHYNPATGKF	227
***Rattus norvegicus* Adig**	183	----RCGSIVLKSAFSVGIT-TS------Y-PEERLPIIFNKVLFN-EG-EHYNPATGKF	228
***Cavia porcellus* ADIG**	140	----RCGSIVLKSAFSVGIT-TS------Y-PEERLPIIFNKVLFN-EG-EHYNPVTGKF	185
***Oryctolagus cuniculus* ADIG**	140	----RCGSIVLKSAFSVGIT-TS------Y-PEERLPIIFNKVLFN-EG-EHYNPATGKF	185
***Tursiops truncatus* ADIG**	192	----RCGSIVLKSAFSVGIT-TS------Y-PEERLPIIFNKVLFN-EG-EHYNPATGKF	237
***Bos taurus* ADIG**	140	----RCGSIVLKSAFSVGIT-TS------Y-PEERLPIIFNKVLFN-EG-EHYNPATGKF	185
***Equus caballus* ADIG**	181	----KCGSIVLKSAFSVGIT-TS------Y-PEERLPIIFNKVLFN-EG-EHYNPATGKF	226
***Canis lupus familiaris* ADIG**	181	----RCGSIMLKSAFSVGIT-TS------Y-PEERLPIIFNKVLFN-EG-EHYNPATGKF	226
***Myotis lucifugus* ADIG**	140	----RCGSIVLKSAFSVGIT-TS------Y-PEERLPIIFNKVLFN-EG-EHYNPATGKF	185
***Homo sapiens* ADIH**	142	----SCGSGHTKSAFSVAVT-KS------Y-PRERLPIKFDKILMN-EG-GHYNASSGKF	187
***Pan troglodytes* ADIH**	142	----SCGSGHTKSAFSVAVT-KS------Y-PRERLPIKFDKILMN-EG-GHYNASSGKF	187
***Pongo abelii* ADIH**	142	----SCGSGHTKSAFSVAVT-KS------Y-PRERLPIKFDKILMN-EG-GHYNASSGKF	187
***Nomascus leucogenys* ADIH**	142	----SCGSGHTKSAFSVAVT-KS------Y-PRERLPIKFDKILMN-EG-GHYNASSGKF	187
***Macaca mulatta* ADIH**	142	----SCGSGHTKSAFSVAVT-KS------Y-PRERLPIKFDKILMN-EG-GHYNASSGKF	187
***Papio hamadryas* ADIH**	142	----SCGSGHTKSAFSVAVT-KS------Y-PRERLPIKFDKILMN-EG-GHYNASSGKF	187
***Mus musculus* Adih**	149	----SCGSSRAKSAFSVAVT-KS------Y-PRERLPIKFDKILMN-EG-GHYNASSGKF	194
***Rattus norvegicus* Adih**	186	----SCGSSRAKSAFSVAVT-KS------Y-PRERLPIKFDKILMN-EG-GHYNASSGKF	231
***Dipodomys ordii* ADIH**	142	----SCGSGRAKSAFSVAVT-KS------Y-PRERLPIKFDKILMN-EG-GHYNASSGKF	187
***Cavia porcellus* ADIH**	142	----SCGSGRPKSAFSVAVT-KS------Y-PRERLPIKFDKILMN-EG-GHYNSSSGKF	187
***Bos taurus* ADIH**	159	----SCGSSHAKSAFSVAVT-KS------Y-PRERLPIKFDKILMN-EG-GHYNASSGKF	204
***Equus caballus* ADIH**	142	----SCGSGRAKSAFSVAVT-KS------Y-PRERLPIKFDKILMN-EG-GHYNASSGKF	187
***Canis lupus familiaris* ADIH**	142	----SCGSGHAKSAFSVAVT-KS------Y-PRERLPIKFDKILMN-EG-GHYNASSGKF	187
***Pteropus vampyrus* ADIH**	142	----SCSSGHAKSAFSVAVT-KS------Y-PRERLPIKFDKILMN-EG-SHYNASSGKF	187
***Loxodonta africana* ADIH**	142	----SCGSARAKSAFSVAVT-KS------Y-PRERLPIKFDKILMN-EG-GHYNASSGKF	187
***Homo sapiens* ADII**	152	AT-FS----GPKIAFYVGLKSP-H--E-----GYEV-LKFDDVVTNL-G-NHYDPTTGKF	195
***Pongo abelii* ADII**	152	AT-FS----GPKIAFYVGLKSP-H--E-----GYEV-LKFDDVVTNL-G-NHYDPTTGKF	195
***Macaca mulatta* ADII**	152	AT-FS----GPKIAFYVGLKSP-H--E-----GYEV-LKFDDVVTNL-G-NHYDPTTGKF	195
***Mus musculus* Adii**	152	AA-FS----GPKIAFYVGLKSP-H--E-----GYEV-LKFDDVVTNL-G-NHYDPTTGKF	195
***Rattus norvegicus* Adii**	152	AA-FS----GPKIAFYVGLKSP-H--E-----GYEV-LKFDDVVTNL-G-NHYDPTTGKF	195
***Bos taurus* ADII**	152	AA-FS----GPKIAFYVGLKSP-H--E-----GYEV-LKFDDVVTNL-G-NHYDPTTGKF	195
***Canis lupus familiaris* ADII**	152	AA-FS----GPKIAFYVGLKSP-H--E-----GYEV-LKFDDVVTNL-G-NHYDPTTGKF	195
***Dasypus novemcinctus* ADII**	152	AA-FS----GPKIAFYVGLKSP-H--E-----GYEV-LKFDDVVTNL-G-NHYDPTTGKF	195
***Homo sapiens* ADIJ**	118	AATYS---TVPKIAFYAGLKRQ-H--E-----GYEV-LKFDDVVTNL-G-NHYDPTTGKF	163
***Pan troglodytes* ADIJ**	118	AATYS---TVPKIAFYAGLKRQ-H--E-----GYEV-LKFDDVVTNL-G-NHYDPTTGKF	163
***Papio hamadryas* ADIJ**	118	AATYS---TVPKIAFYAGLKRQ-H--E-----GYEV-LKFDDVVTNL-G-NHYDPTTGKF	163
***Mus musculus* Adij**	118	AATYS---TVPKIAFYAGLKRQ-H--E-----GYEV-LKFDDVVTNL-G-NHYDPTTGKF	163
***Rattus norvegicus* Adij**	118	AATYS---TVPKIAFYAGLKRQ-H--E-----GYEV-LKFDDVVTNL-G-NHYDPTTGKF	163
***Oryctolagus cuniculus* ADIJ**	118	AATYS---TVPKIAFYAGLKRQ-H--E-----GYEV-LKFDDVVTNL-G-NHYDPTTGKF	163
***Bos taurus* ADIJ**	118	AATYS---TVPKIAFYAGLKRQ-H--E-----GYEV-LKFDDVVTNL-G-NHYDPTTGKF	163
***Canis lupus familiaris* ADIJ**	118	AATYS---TVPKIAFYAGLKRQ-H--E-----GYEV-LKFDDVVTNL-G-NHYDPTTGKF	163
***Loxodonta africana* ADIJ**	118	AATYS---TVPKIAFYAGLKRQ-H--E-----GYEV-LKFDDVVTNL-G-NHYDPTTGKF	163
***Homo sapiens* ADIK**	103	AAGY-----VPRIAFYAGLRRP-H--E-----GYEV-LRFDDVVTNV-G-NAYEAASGKF	146
***Pan troglodytes* ADIK**	103	AAGY-----VPRIAFYAGLRRP-H--E-----GYEV-LRFDDVVTNV-G-NAYEAASGKF	146
***Gorilla gorilla* ADIK**	103	AARY-----VPRIAFYAGLRRP-H--E-----GYEV-LRFDDVVTNV-G-NAYEAASGKF	146
***Papio hamadryas* ADIK**	103	AAGY-----VPRIAFYAGLRRP-H--E-----GYEV-LRFDDVVTNV-G-NAYEAASGKF	146
***Callithrix jacchus* ADIK**	103	PAGY-----VPRIAFYAGLRRP-H--E-----GYEV-LRFDDVVTNV-G-NAYEAASGKF	146
***Rattus norvegicus* Adik**	103	PAGY-----VPRIAFYAGLRRP-H--E-----GYEV-LRFDDVVTNV-G-NAYEAASGKF	146
***Cavia porcellus* ADIK**	103	PAGY-----LPRIAFYAGLRRP-H--E-----GYEV-LRFDDVVTNV-G-NAYEAASGKF	146
***Equus caballus* ADIK**	103	PAGY-----VPRIAFYAGLRRP-H--E-----GYEV-LRFDDVVTNV-G-NAYEAASGKF	146
***Canis lupus familiaris* ADIK**	103	PAGY-----VPRIAFYAGLRRP-H--E-----GYEV-LRFDDVVTNV-G-NAYEAASGKF	146
***Dasypus novemcinctus* ADIK**	103	PAGY-----MPRIAFYAGLRRP-H--E-----GYEV-LRFDDVVTNV-G-NAYEAASGKF	146
***Loxodonta africana* ADIK**	103	PAGY-----VPRIAFYAGLRRP-H--E-----GYEV-LRFDDVVTNV-G-NAYEAASGKF	146
***Mus musculus* Adil**	121	TATYT---TVPRVAFYAGLKNP-H--E-----GYEV-LKFDDVVTNL-G-NNYDAASGKF	166
***Rattus norvegicus* Adil**	121	TATYT---TVPRVAFYAGLKNP-H--E-----GYEV-LKFDDVVTNL-G-NNYDAASGKF	166
***Homo sapiens* ADIM**	225	--------PELQIAFMASLAT--HFSN-----QNSG-IIFSSVETNI-G-NFFDVMTGRF	266
***Pan troglodytes* ADIM**	225	--------PELQIAFMASLAT--HFSN-----QNSG-IIFSSVETNI-G-NFFDVMTGRF	266

***Pongo abelii* ADIM**	181	--------PELQIAFMASLAT--HFSN-----QNSG-IIFSSVETNI-G-NFFDVMTGRF	222
***Nomascus leucogenys* ADIM**	225	--------PELQIAFMASLAT--HFSN-----QNSG-IIFSSVETNI-G-NFFDVMTGRF	266
***Papio hamadryas* ADIM**	225	--------PELQIAFMASLAT--HFSN-----QNSG-IIFSSVETNI-G-NFFDVMTGRF	266
***Callithrix jacchus* ADIM**	215	--------PELQIAFMASLAT--HFSN-----QNSG-IIFSSVETNI-G-NFFDVMTGRF	256
***Mus musculus* Adim**	114	--------PELQIAFMASLAT--HFSN-----QNSG-IIFSSVETNI-G-NFFDVMTGRF	155
***Rattus norvegicus* Adim**	188	--------PELQIAFMASLAT--HFSN-----QNSG-IIFSSVETNI-G-NFFDVMTGRF	229
***Cavia porcellus* ADIM**	181	--------PELQIAFMASLAT--HFTN-----QNSG-IIFSSVETNI-G-NFFDVMTGRF	222
***Bos taurus* ADIM**	114	--------PELQIAFMASLAT--HFTN-----QNSG-IIFSSVETNI-G-NFFDVMTGRF	155
***Equus caballus* ADIM**	114	--------PELQIAFMASLAT--HFTN-----QNSG-IIFSSVETNI-G-NFFDVMTGRF	155
***Canis lupus familiaris* ADIM**	175	--------PELQIAFMASLAT--HFSN-----QNSG-IIFSSVETNI-G-NFFDVMTGRF	216
***Homo sapiens* ADIN**	172	P-------PEPRSAFSAARTRSLVGSDAGPGPRHQ-PLAFDTEFVNIGG--DFDAAAGVF	221
***Pan troglodytes* ADIN**	172	P-------PEPRSAFSAARTRSLVGSDAGPGPRHR-PLAFDTEFVNIGG--DFDAAAGVF	221
***Pongo abelii* ADIN**	172	P-------PEPRSAFSAARTRSLVGSDAGPGPRHR-PLAFDTEFVNIGG--DFDAAAGVF	221
***Nomascus leucogenys* ADIN**	167	P-------PEPRSAFSAARTRSLVGSDAGPGPRHR-PLAFDTEFVNIGG--DFDAAAGVF	216
***Macaca mulatta* ADIN**	174	P-------PEPRSAFSAARTRSLVGSDAGSGPRHR-PLSFDTELVNIGG--DFDAAAGVF	223
***Callithrix jacchus* ADIN**	172	P-------PEPRSAFSAARTHSLVGSDASPGPRHQ-PLAFDTELVNIGG--DFDAAAGVF	221
***Mus musculus* Adin**	170	A-------PEPRSAFSAARTRSLVGSDAAPGPRHR-PLAFDTELVNIGG--DFDAAAGVF	219
***Cavia porcellus* ADIN**	182	P-------SQPRSAFSAARTRSLVGADAGPGPRHR-PLAFDTELVNIGG--DFDAAAGVF	231
***Tursiops truncatus* ADIN**	168	P-------PEPRSAFSAARTRSLVGSDAGPGPRHR-PLAFDTELVNIGG--DFDAAAGVF	217
***Canis lupus familiaris* ADIN**	168	P-------PEPRSAFSAARTRSLVGSDAGPGPRHR-PLAFDTELVNIGG--DFDAAAGVF	217
***Dasypus novemcinctus* ADIN**	168	P-------PEPRSAFSAARTRSLVGSDAGPGPRHR-PLAFDTELVNIGG--DFDAAAGVF	217
***Loxodonta africana* ADIN**	168	P-------PEPRSAFSAARTRSLVGSDSGPGPRHR-PLAFDTELVNIGG--DFDAAAGVF	217
***Homo sapiens* ADIO**	112	-----C--RRAYAAFSVGRREGLHSSD-----HFQA-VPFDTELVNLDG--AFDLAAGRF	156
***Pan troglodytes* ADIO**	112	-----C--QRAYAAFSVGRREGLHSSD-----HFQA-VPFDTELVNLDG--AFDLAGGRF	156
***Macaca mulatta* ADIO**	107	-----C--QRAYAAFSVGRREGLHSSD-----DFQA-VPFDTELVNLDG--AFDLAAGRF	151
***Bos taurus* ADIO**	111	-----C--PRAYAAFSVGRREGLHSAD-----ALQA-VTFDTELVNLDG--AFDLASGRF	155
***Myotis lucifugus* ADIO**	111	-----C--QRAYAAFSVGRREGLHSTD-----AFQA-VPFDTELVNLDG--AFDLASGRF	155
***Homo sapiens* ADIP**	239	-----C--KSHYAAFSVGRKKPMHSNH-----YYQT-VIFDTEFVNLYD--HFNMFTGKF	283
***Otolemur garnettii* ADIP**	141	-----C--KNHYAAFSVGRKKPLHSND-----YYQT-VVFDTEFVNLYG--HFNMFTGRF	185
***Mus musculus* Adip**	141	-----C--KSQYAAFSVGRKKALHSND-----YFQP-VVFDTEFVNLYK--HFNMFTGKF	185
***Cavia porcellus* ADIP**	141	-----C--KNYYAAFSVGRKKPLHSND-----YYQT-VVFDTEFVNLYG--HFNMFTGRF	185
***Bos taurus* ADIP**	164	-----C--KNHYAAFSVGRKKPLHSND-----YYQT-VIFDTEFVNLYS--HFNMFTGKF	208
***Equus caballus* ADIP**	139	-----C--KNHYAAFSVGRKKPLHSND-----YYQT-VIFDTEFVNLYS--HFNMFTGKF	183
***Loxodonta africana* ADIP**	193	-----C--KNHYAAFSVGRKKPLHSND-----YYQT-VVFDTEFVNLYG--HFNMFTGKF	237
***Homo sapiens* ADIQ**	139	-----C--QKRFFAFSVGRKTALHSGE-----DFQT-LLFERVFVNLDGC--FDMATGQF	183
***Pan troglodytes* ADIQ**	139	-----C--QKRFFAFSVGRKTALHSGE-----DFQT-LLFERVFVNLDGC--FDMATGQF	183
***Pongo abelii* ADIQ**	139	-----C--QKRFFAFSVGRKTALHSGD-----DFQT-LLFERVFVNLDGC--FDMATGQF	183
***Nomascus leucogenys* ADIQ**	139	-----C--QKRFFAFSVGRKTALHSGE-----DFQT-LLFERVFVNLDGC--FDMVTGQF	183
***Macaca mulatta* ADIQ**	139	-----C--QKRFFAFSVGRKTALHSGE-----DFQR-LLFERVFVNLDGC--FDMAAGHF	183
***Mus musculus* Adiq**	125	-----C--QTHYSAFSVGRKTGLHSSE-----NFLS-LLFDRVFVNTDGH--FDMATGSF	169
***Rattus norvegicus* Adiq**	124	-----C--QTHYSAFSVGRKTGLHSSE-----NFLS-LLFDRVFVNTDGH--FDMATGRF	168
***Homo sapiens* ADIR**	24	P-------QQMRVAFSAARTSNL----A-PGTLDQ-PIVFDLLLNNLGE--TFDLQLGRF	68
***Pan troglodytes* ADIR**	24	P-------QQMRVAFSAARTSNL----A-PGTLDQ-PIVFDLLLNNLGE--TFDLQLGRF	68
***Pongo abelii* ADIR**	24	P-------QQMRVAFSAARTSNL----A-PGTLDQ-PIVFDLLLNNLGE--TFDLQLGRF	68
***Nomascus leucogenys* ADIR**	24	P-------QQMRVAFSAARTSNL----A-PGTLDQ-PIVFDLLLNNLGE--TFDLQLGRF	68
***Macaca mulatta* ADIR**	24	P-------QQMRVAFSAARTSNL----A-PGTLDQ-PIVFDLLLNNLGE--TFDLQLGRF	68
***Papio hamadryas* ADIR**	24	P-------QQMRVAFSAARTSNL----A-PGTLDQ-PIVFDLLLNNLGE--TFDLQLGRF	68
***Callithrix jacchus* ADIR**	24	P-------QQMRVAFSAARTSNL----A-PGTLDQ-PIVFDLLLNNLGE--TFDLQLGRF	68
***Tarsius syrichta* ADIR**	24	P-------QQMRVAFSAARTSNL----A-PGTLDQ-PIVFDLLLNNLGE--TFDLQLGRF	68
***Otolemur garnettii* ADIR**	24	P-------QQMRVAFSAARTSNL----A-PGTLDQ-PIVFDLLLNNLGE--TFDLQLGRF	68
***Tupaia belangeri* ADIR**	24	P-------QQMRVAFSAARTSNL----A-PGTLDQ-PIVFDLLLNNLGE--TFDLQLGRF	68
***Mus musculus* Adir**	24	P-------QQMRVAFSAARTSNL----A-PGTLDQ-PIVFDLLLNNLGE--TFDLQLGRF	68
***Rattus norvegicus* Adir**	24	P-------QQMRVAFSAARTSNL----A-PGTLDQ-PIVFDLLLNNLGE--TFDLQLGRF	68
***Cavia porcellus* ADIR**	24	P-------QQMRVAFSAARTSNL----A-PGTLDQ-PIVFDLLLNNLGE--TFDLQLGRF	68
***Oryctolagus cuniculus* ADIR**	24	P-------QQMRVAFSAARTSNL----A-PGTLDQ-PIVFDLLLNNLGE--TFDLQLGRF	68
***Tursiops truncatus* ADIR**	24	P-------QQMRVAFSAARTSNL----A-PGTLDQ-PIVFDLLLNNLGE--TFDLQLGRF	68
***Bos taurus* ADIR**	24	P-------QQMRVAFSAARTSNL----A-PGTLDQ-PIVFDLLLNNLGE--TFDLQLGRF	68
***Equus caballus* ADIR**	24	P-------QQMRVAFSAARTSNL----A-PGTLDQ-PIVFDLLLNNLGE--TFDLQLGRF	68
***Canis lupus familiaris* ADIR**	24	P-------QQMRVAFSAARTSNL----A-PGTLDQ-PIVFDLLLNNLGE--TFDLQLGRF	68
***Myotis lucifugus* ADIR**	24	P-------QQMRVAFSAARTSNL----A-PGTLDQ-PIVFDLLLNNLGE--TFDLQLGRF	68
***Pteropus vampyrus* ADIR**	24	P-------QQMRVAFSAARTSNL----A-PGTLDQ-PIVFDLLLNNLGE--TFDLQLGRF	68
***Erinaceus europaeus* ADIR**	1	----------MRVAFSAARTSNL----T-PGTLDQ-PIVFDLLINNLGE--TFDLQLGRF	42
***Dasypus novemcinctus* ADIR**	24	P-------QQMRVAFSAARTSNL----A-PGTLDQ-PIVFDLLLNNLGE--TFDLQLGRF	68
***Choloepus hoffmanni* ADIR**	24	P-------QQMRVAFSAARTSNL----A-PGTLDQ-PIVFDLLLNNLGE--TFDLQLGRF	68
***Loxodonta africana* ADIR**	24	P-------QQMRVAFSAARTSNL----A-PGTLDQ-PIVFDLLLNNLGE--TFDLQLGRF	68

```
Homo sapiens ADIA               161 -TCKVPGLYYFTYH--------AS--SRGNLCVNLMRG--RERAQKVVTFC--DYAY--- 202
                                         730        740        750        760        770        780
Homo sapiens ADIA               161 -TCKVPGLYYFTYH--------AS--SRGNLCVNLMRG--RERAQKVVTFC--DYAY--- 202
Papio hamadryas ADIA            161 -TCRVPGLYYFTYH--------AS--SRGNLCVKLMRG--RERPQKVVTFC--DYAY--- 202
Mus musculus Adia               188 -TCKVPGLYYFTYH--------AS--SRGNLCVNLVRGRDRDSMQKVVTFC--DYAQ--- 231
Rattus norvegicus Adia          159 -TCKVPGLYYFTYH--------AS--SRGNLCVNIVRGRDRDRMQKVLTFC--DYAQ--- 202
Cavia porcellus ADIA            159 -TCKVPGLYYFTYH--------AS--SRGNLCVNLMQG--REHLKKVVTFC--DYVR--- 200
Ochotona princeps ADIA          159 -TCTVPGLYYFTYH--------AS--SRGNLCVGIFRG--REKPQKVLTFC--DYSI--- 200
Bos taurus ADIA                 155 -TCKVPGLYFFTYH--------AS--SRGQLCVDLMRG--RAEPQKVVTFC--DYVQ--- 196
Equus caballus ADIA             160 -TCKVPGLYYFAYH--------AS--SRGNLCVNLMRG--RERIQKVVTFC--DFVH--- 201
Canis lupus familiaris ADIA     158 -TCNVPGIYYFAYH--------AS--SRGNLCVNVMRG--RERMQKVVTFC--DYVQ--- 199
Felis catus ADIA                162 -TCRVPGIYYFTYH--------AS--SRGNLCVNLMRG--REQMQKVVTFC--DYVH--- 203
Myotis lucifugus ADIA           159 -TCKVPGLYYFTYH--------AS--SRGNLCVNLMRG--LERMEKVVTFC--DYVQ--- 200
Dasypus novemcinctus ADIA       156 -TCKVPGLYYFTFH--------AS--SRGNLCVNLVRG--RERPQRVVAFC--DFVH--- 197
Loxodonta africana ADIA         159 -TCRVPGLYYFTYH--------AS--SRGNLCVNVVRG--RERVQKVVTFC--DYVQ--- 200
Homo sapiens ADIB               159 -TCKVPGLYYFVYH--------AS--HTANLCVLLYR-----SGVKVVTFC--GHTS--- 197
Pan troglodytes ADIB            159 -TCKVPGLYYFVYH--------AS--HTANLCVLLYR-----SGVKVVTFC--GHTS--- 197
Pongo abelii ADIB               159 -TCKVPGLYYFVYH--------AS--HTANLCVLLYR-----SGVKVVTFC--GHTS--- 197
Nomascus leucogenys ADIB        160 -TCKVPGLYYFVYH--------AS--HTANLCVLLYR-----SGVKVVTFC--GHTS--- 198
Callithrix jacchus ADIB         158 -TCKVPGLYYFVYH--------TS--QTANLCVLLYR-----NGVKVVTFC--DHMS--- 196
Otolemur garnettii ADIB         157 -TCKVPGLYYFVYH--------TS--QTANLCVHLYH-----NNVRVTTFC--DHMS--- 195
Mus musculus Adib               190 -TCEVPGLYYFVYY--------TS--HTANLCVHLNL-----NLARVASFC--DHMF--- 228
Rattus norvegicus Adib          182 -TCKVPGLYYFVHH--------TS--QTANLCVQLLL-----NNAKVTSFC--DHMS--- 220
Cavia porcellus ADIB            206 -TCQVPGLYYFIYH--------TS--LTENLCVQLYH-----DNVKVTTFC--EHVLQGN 247
Oryctolagus cuniculus ADIB      212 -TCKVPGLYYFVHH--------TS--HTANLCVQLYH-----NGARVTAFC--DHMA--- 250
Bos taurus ADIB                 157 -TCKVPGLYYFVFH--------TS--HTSNLCVLLYR-----SGFKVATFC--DHMT--- 195
Equus caballus ADIB             177 -TCRVPGIYYFAHH--------TS--QTANLCVQLYR-----NGAKVTAFC--DHMS--- 215
Canis lupus familiaris ADIB     159 -TCKVPGLYYFVYH--------TS--LTSNLCVHLYR-----SGTRVTTFC--DHMS--- 197
Myotis lucifugus ADIB           159 -TCKVPGLYYFVHH--------TS--QTANLCVQLYR-----NEIQMTSFC--DHMS--- 197
Loxodonta africana ADIB         157 -TCKVPGLYYFVYH--------TS--QTANLCVQLYR-----SGVKVTTFC--DHMS--- 195
Homo sapiens ADIC               152 -VCTVPGYYYFTFQ--------VL--SQWEICLSIVSSS-RGQVRRSLGFC--DTTN--- 194
Macaca mulatta ADIC             152 -VCTVPGYYYFTFQ--------VV--SEREICLSIVSSS-RGQVRRSLGFC--DTTN--- 194
Papio hamadryas ADIC            152 -VCTVPGYYYFTFQ--------VV--SEREICLSIVSSS-RGQVRRSLGFC--DTTN--- 194
Callithrix jacchus ADIC         152 -VCAVPGYYYFTFQ--------VV--SKWDICLFIASSS-RGQVRRSLGFC--DANS--- 194
Mus musculus Adic               152 -ICAVPGFYYFNFQ--------VI--SKWDLCLFIKSSS-GGQPRDSLSFS--NTNN--- 194
Rattus norvegicus Adic          164 -ICAVPGFYYFTFQ--------VI--SKWDLCLSIVSSS-RGQPRNSLGFC--DTNS--- 206
Cavia porcellus ADIC            152 -VCAVPGFYYFSFY--------VV--ATGSICLTIVSSS-QGQLRHSPGFC--DTNN--- 194
Oryctolagus cuniculus ADIC      193 -VCAVPGFYYFTFQ--------VV--SKWDLCLFIVSAS-RGQTRRSVGFC--DTNS--- 235
Equus caballus ADIC             185 -VCSVPGYYYFTFQ--------VV--SKWDVCLSIVSLR-RDQVRHSLGFC--DTNS--- 227
Canis lupus familiaris ADIC     171 -ICAVPGYYYFTFQ--------VV--SKWDICLSIVSSG-RAQIRRSLGFC--DTNS--- 213
Myotis lucifugus ADIC           152 -VCAVPGYYYFTFQ--------VV--SKWDICLSIMSSR-RGLTQRSVGFC--DANS--- 194
Loxodonta africana ADIC         167 -VCAVPGYYYFTFQ--------VV--SKRDICLSIVSSG-RNHPRRSLGFC--DTNS--- 209
Homo sapiens ADID               182 -HCNIPGLYYFAYH--------ITVYMKDVK-VSLFK---KDK-AMLFTY---DQYQE-- 222
Pan troglodytes ADID            182 -HCNIPGLYYFAYH--------ITVYMKDVK-VSLFK---KDK-AMLFTY---DQYQE-- 222
Pongo abelii ADID               151 -HCNIPGLYYFAYH--------ITVYMKDVK-VSLFK---KDK-AMLFTY---DQYQE-- 191
Nomascus leucogenys ADID        181 -HCNIPGLYYFAYH--------ITVYMKDVK-VSLFK---KDK-AMLFTY---DQYQE-- 221
Macaca mulatta ADID             180 -HCNIPGLYYFAYH--------ITVYMKDVK-VSLFK---KDK-AMLFTY---DQYQE-- 220
Otolemur garnettii ADID         151 -LCNIPGLYYFSYH--------ITVYMKDVK-VSLFK---KDK-AVIVTY---DQYQD-- 191
Mus musculus Adid               165 -YCNIPGLYYFSYH--------ITVYMKDVK-VSLFK---KDK-AVLFTY---DQYQE-- 205
Rattus norvegicus Adid          151 -HCNIPGLYYFSYH--------ITVYMKDVK-VSLFK---KDK-AVLFTY---DQYQE-- 191
Cavia porcellus ADID            193 -HCNIPGLYYFSYH--------VTVYLKDVK-VSLFR---NDK-AELFTY---DQYQD-- 233
Oryctolagus cuniculus ADID      161 -RCNIPGLYYFSYH--------ITVYMKDVK-VSLFK---KDK-AMLFTY---DQYQD-- 201
Bos taurus ADID                 146 -LCNIPGLYYFSYH--------ITVYLKDVK-VSLYK---NDK-ALLFTH---DQFQD-- 186
Equus caballus ADID             208 -HCNIPGLYYFSYH--------ITVYLKDVK-VSLYK---KDK-AVLFTY---DQYQD-- 248
Canis lupus familiaris ADID     160 -HCNIPGLYYFSYH--------ITVYLKDVK-VSLYK---KDK-AMLFTY---DQYQE-- 200
Felis catus ADID                151 -HCNIPGLYYFSYH--------ITVYLKDVK-VSLYK---RDK-AMLFTY---DQYQE-- 191
Myotis lucifugus ADID           160 -HCNIPGLYYFSYH--------ITVYHKDVK-VSLYK---KDK-AVLFTF---DKYQD-- 200
Dasypus novemcinctus ADID       150 -HCNIPGLYYFSYH--------ITVYMKDVK-VSLFK---KDK-AVLFTY---DQYQE-- 190
Loxodonta africana ADID         151 -HCNIPGVYFFSYH--------ITVYMKDVK-VSLFK---KDK-AVLFTY---DQYQE-- 191
Homo sapiens ADIE               144 -TCQVPGVYYFAVH--------ATVYRASLQ-FDLVK---NGE-SIASFF---QFFGGW- 185
Pan troglodytes ADIE            144 -TCQVPGIYYFAVH--------ATVYRASLQ-FDLVK---NGE-SIASFF---QFFGGW- 185
Pongo abelii ADIE               144 -TCQVPGVYYFAVH--------ATVYRASLQ-FDLVK---NGE-SIASFF---QFFGGW- 185
Papio hamadryas ADIE            144 -TCQVPGVYYFAVH--------ATVYRASLQ-FDLVK---NGE-SIASFF---QFFGGW- 185
Callithrix jacchus ADIE         144 -TCQVPGVYYFAVH--------ATVYRASLQ-FDLMK---NGE-SIASFF---QFFGGW- 185
Otolemur garnettii ADIE         144 -TCQVPGVYYFAVH--------ATVYRASLQ-FDLVK---NGV-SIASFF---QFFGGW- 185
Mus musculus Adie               144 -TCQVPGVYYFAVH--------ATVYRASLQ-FDLVK---NGQ-SIASFF---QYFGGW- 185
Rattus norvegicus Adie          144 -TCQVPGVYYFAVH--------ATVYRASLQ-FDLVK---NGQ-SIASFF---QFFGGW- 185
Homo sapiens ADIF1              242 -TCHIAGVYYFTYH--------ITVFSRNVQ-VSLVK---NGV-KILHTK---DAYM--- 281
Homo sapiens ADIF2              242 -TCHIAGVYYFTYH--------ITVFSRNVQ-VSLVK---NGV-KILHTR---DAYV--- 281
Pan troglodytes ADIF1           242 -TCHIAGVYYFTYH--------ITVFSRNVQ-VSLVK---NRV-KILHTK---DAYM--- 281
Pongo abelii ADIF1              242 -TCHIAGVYYFTYH--------ITVFSRNVQ-VSLVK---NGV-KILHTK---DAYM--- 281
Nomascus leucogenys ADIF1       242 -TCHIAGVYYFTYH--------ITVFSRNVQ-VSLVK---NGV-KILHTK---DAYM--- 281
Macaca mulatta ADIF1            242 -TCHIAGVYYFTYH--------ITVFSRNVQ-VSLVK---NGV-KILHTK---DAYM--- 281
Papio hamadryas ADIF1           242 -TCHIAGVYYFTYH--------ITVFSRNVQ-VSLVK---NGV-KILHTK---DAYM--- 281
```

```
Microcebus murinus ADIF1        242 -TCHVAGVYYFTYH--------ITVFSRNVQ-VSLVK---NGA-KVLHTK---DGYM--- 281
Otolemur garnettii ADIF1        242 -VCHVAGVYYFTYH--------ITVFSRNVQ-VSLVK---NGV-KILHTK---DGYM--- 281
Mus musculus Adif1              271 -TCHVAGVYYFTYH--------ITVFSRNVQ-VSLVK---NGV-KVLHTK---DSYM--- 310
Rattus norvegicus Adif1         258 -TCHVAGVYYFTYH--------ITVFSRNVQ-VSLVK---NGV-KVLHTK---DGYM--- 297
Cavia porcellus ADIF1           242 -TCHIAGTYYFTYH--------VTVFSRNVQ-VALVK---NGV-KVLHTK---DGYM--- 281
Oryctolagus cuniculus ADIF1     242 -TCHVAGVYYFTYH--------ITVFSRNVQ-VSLVK---NGV-KVLHTK---DGYM--- 281
Ochotona princeps ADIF1         242 -ICYVAGVYYFTYH--------ITVFSRNVQ-VSLVK---NGM-KVLHTK---DAYM--- 281
Bos taurus ADIF1                242 -ICHVAGVYYFTYH--------ITVFSRNIQ-VSLVK---NGA-KVLHTK---DGYT--- 281
Equus caballus ADIF1            242 -TCHLAGVYYFTYH--------VTVFSRNVQ-VSLVK---NGV-KILHTK---DGYT--- 281
Canis lupus familiaris ADIF1    242 -TCHVAGVYYFTYH--------ITVFSRNVQ-VSLVK---NGV-KILHTK---DSYM--- 281
Felis catus ADIF1               242 -TCHVAGVYYFTYH--------ITVFSRNVQ-VALVK---NGV-KILHTK---DGYM--- 281
Dasypus novemcinctus ADIF1      243 -TCHIAGVYYFTYH--------ITVFSRNVQ-VSLVK---NGV-KVLHTK---DSYM--- 282
Loxodonta africana ADIF1        242 -TCHIAGVYYFTYH--------ITVFSRNVQ-VSLVK---NGV-KVLHTK---DGYL--- 281
Homo sapiens ADIG               240 -ICAFPGIYYFSYD--------ITLANKHLA-IGLVH---NGQ-YRIKTF---DANTG-- 280
Pan troglodytes ADIG            240 -ICAFPGIYYFSYD--------ITLANKHLA-IGLVH---NGQ-YRIKTF---DANTG-- 280
Pongo abelii ADIG               240 -ICAFPGIYYFSYD--------ITLANKHLA-IGLVH---NGQ-YRIKTF---DANTG-- 280
Callithrix jacchus ADIG         226 -ICAFPGIYYFSYD--------ITLANKHLA-IGLVH---NGQ-YRIKTF---DANTG-- 266
Otolemur garnettii ADIG         186 -ICAFPGIYYFSYD--------ITLANKHLA-IGLVH---NGQ-YRIKTF---DANTG-- 226
Mus musculus Adig               228 -ICAFPGIYYFSYD--------ITLANKHLA-IGLVH---NGQ-YRIRTF---DANTG-- 268
Rattus norvegicus Adig          229 -ICAFPGIYYFSYD--------ITLANKHLA-IGLVH---NGQ-YRIRTF---DANTG-- 269
Cavia porcellus ADIG            186 -ICAFPGIYYFSYD--------ITLANKHLA-IGLVH---NGQ-YKIKTF---DANTG-- 226
Oryctolagus cuniculus ADIG      186 -ICAFPGIYYFSYD--------ITLANKHLA-IGLVH---NGQ-YRIRTF---DANTG-- 226
Tursiops truncatus ADIG         238 -ICAFPGIYYFSYD--------ITLANKHLA-IGLVH---NGQ-YRIKTF---DANTG-- 278
Bos taurus ADIG                 186 -ICAFPGIYYFSYD--------ITLANKHLA-IGLVH---NGQ-YRIKTF---DANTG-- 226
Equus caballus ADIG             227 -ICAFPGIYYFSYD--------ITLANKHLA-IGLVH---NGQ-YRIKTF---DANTG-- 267
Canis lupus familiaris ADIG     227 -ICAFPGIYYFSYD--------ITLANKHLA-IGLVH---NGQ-YRIKTF---DANTG-- 267
Myotis lucifugus ADIG           186 -ICAFPGIYYFSYD--------ITLANKHLA-IGLVH---NGQ-YRIKTF---DANTG-- 226
Homo sapiens ADIH               188 -VCGVPGIYYFTYD--------ITLANKHLA-IGLVH---NGQ-YRIRTF---DANTG-- 228
Pan troglodytes ADIH            188 -VCGVPGIYYFTYD--------ITLANKHLA-IGLVH---NGQ-YRIRTF---DANTG-- 228
Pongo abelii ADIH               188 -VCGVPGIYYFTYD--------ITLANKHLA-IGLVH---NGQ-YRIRTF---DANTG-- 228
Nomascus leucogenys ADIH        188 -VCGVPGIYYFTYD--------ITLANKHLA-IGLVH---NGQ-YRIRTF---DANTG-- 228
Macaca mulatta ADIH             188 -VCGVPGIYYFTYD--------ITLANKHLA-IGLVH---NGQ-YRIRTF---DANTG-- 228
Papio hamadryas ADIH            188 -VCGVPGIYYFTYD--------ITLANKHLA-IGLVH---NGQ-YRIRTF---DANTG-- 228
Mus musculus Adih               195 -VCSVPGIYYFTYD--------ITLANKHLA-IGLVH---NGQ-YRIRTF---DANTG-- 235
Rattus norvegicus Adih          232 -VCSVPGIYYFTYD--------ITLANKHLA-IGLVH---NGQ-YRIRTF---DANTG-- 272
Dipodomys ordii ADIH            188 -VCGVPGVYYFTYD--------ITLANKHLA-IGLVH---NGQ-YRIRTF---DANTG-- 228
Cavia porcellus ADIH            188 -ICGVPGIYYFTYD--------ITLANKHLA-IGLVH---NGQ-YRIRTF---DANTG-- 228
Bos taurus ADIH                 205 -VCGVPGIYYFTYD--------ITLANKHLA-IGLVH---NGQ-YRIRTF---DANTG-- 245
Equus caballus ADIH             188 -VCGVPGIYYFSYD--------ITLANKHLA-IGLVH---NGQ-YRIRTF---DANTG-- 228
Canis lupus familiaris ADIH     188 -VCGVPGIYYFTYD--------ITLANKHLA-IGLVH---NGQ-YRIRTF---DANTG-- 228
Pteropus vampyrus ADIH          188 -VCSVPGIYYFTYD--------ITLANKHLA-IGLVH---NGQ-YRIRTF---DANTG-- 228
Loxodonta africana ADIH         188 -VCGVPGIYYFTYD--------ITLANKHLA-IGLVH---NGQ-YRIRTF---DANTG-- 228
Homo sapiens ADII               196 -SCQVRGIYFFTYHILMRGGDGTSMW-ADL-C----K---NGQ-VRASAI-AQDADQ--- 240
Pongo abelii ADII               196 -SCQVRGIYFFTYHILMRGGDGTSMW-ADL-C----K---NGQ-VRASAI-AQDADQ--- 240
Macaca mulatta ADII             196 -SCQVRGIYFFTYHILMRGGDGTSMW-ADL-C----K---NGQ-VRASAI-AQDADQ--- 240
Mus musculus Adii               196 -SCQVRGIYFFTYHILMRGGDGTSMW-ADL-C----K---NGQ-VRASAI-AQDADQ--- 240
Rattus norvegicus Adii          196 -SCQVRGIYFFTYHILMRGGDGTSMW-ADL-C----K---NGQ-VRASAI-AQDADQ--- 240
Bos taurus ADII                 196 -SCQVRGIYFFTYHILMRGGDGTSMW-ADL-C----K---NGQ-VRASAI-AQDADQ--- 240
Canis lupus familiaris ADII     196 -SCQVRGIYFFTYHILMRGGDGTSMW-ADL-C----K---NGQ-VRASAI-AQDADQ--- 240
Dasypus novemcinctus ADII       196 -SCQVRGIYFFTYHILMRGGDGTSMW-ADL-C----K---NGQ-VRASAI-AQDADQ--- 240
Homo sapiens ADIJ               164 -TCSIPGIYFFTYHVLMRGGDGTSMW-ADL-C----K---NNQ-VRASAI-AQDADQ--- 208
Pan troglodytes ADIJ            164 -TCSIPGIYFFTYHVLMRGGDGTSMW-ADL-C----K---NNQ-VRASAI-AQDADQ--- 208
Papio hamadryas ADIJ            164 -TCSIPGIYFFTYHVLMRGGDGTSMW-ADL-C----K---NNQ-VRASAI-AQDADQ--- 208
Mus musculus Adij               164 -TCSIPGIYFFTYHVLMRGGDGTSMW-ADL-C----K---NNQ-VRASAI-AQDADQ--- 208
Rattus norvegicus Adij          164 -TCSIPGIYFFTYHVLMRGGDGTSMW-ADL-C----K---NNQ-VRASAI-AQDADQ--- 208
Oryctolagus cuniculus ADIJ      164 -TCSIPGIYFFTYHVLMRGGDGTSMW-ADL-C----K---NNQ-VRASAI-AQDADQ--- 208
Bos taurus ADIJ                 164 -TCSIPGIYFFTYHVLMRGGDGTSMW-ADL-C----K---NNQ-VRASAI-AQDADQ--- 208
Canis lupus familiaris ADIJ     164 -TCSIPGIYFFTYHVLMRGGDGTSMW-ADL-C----K---NNQ-VRASAI-AQDADQ--- 208
Loxodonta africana ADIJ         164 -TCSIPGIYFFTYHVLMRGGDGTSMW-ADL-C----K---NNQ-VRASAI-AQDADQ--- 208
Homo sapiens ADIK               147 -TCPMPGVYFFAYHVLMRGGDGTSMW-ADL-----MK---NGQ-VRASAI-AQDADQ--- 191
Pan troglodytes ADIK            147 -TCPMPGVYFFAYHVLMRGGDGTSMW-ADL-----MK---NGQ-VRASAI-AQDADQ--- 191
Gorilla gorilla ADIK            147 -TCPMPGVYFFAYHVLMRGGDGTSMW-ADL-----MK---NGQ-VRASAI-AQDADQ--- 191
Papio hamadryas ADIK            147 -TCPMPGVYFFAYHVLMRGGDGTSMW-ADL-----MK---NGQ-VRASAI-AQDADQ--- 191
Callithrix jacchus ADIK         147 -TCPMPGVYFFAYHVLMRGGDGTSMW-ADL-----MK---NGQ-VRASAI-AQDADQ--- 191
Rattus norvegicus Adik          147 -TCPMPGVYFFAYHVLMRGGDGTSMW-ADL-----MK---NGQ-VRASAI-AQDADQ--- 191
Cavia porcellus ADIK            147 -TCPMPGVYFFAYHVLMRGGDGTSMW-ADL-----MK---NGQ-VRASAI-AQDADQ--- 191
Equus caballus ADIK             147 -TCPMPGVYFFAYHVLMRGGDGTSMW-ADL-----MK---NGQ-VRASAI-AQDADQ--- 191
Canis lupus familiaris ADIK     147 -TCPMPGVYFFAYHVLMRGGDGTSMW-ADL-----MK---NGQ-VRASAI-AQDADQ--- 191
Dasypus novemcinctus ADIK       147 -TCPMPGVYFFAYHVLMRGGDGTSMW-ADL-----MK---NGQ-VRASAI-AQDADQ--- 191
Loxodonta africana ADIK         147 -TCPMPGVYFFAYHVLMRGGDGTSMW-ADL-----MK---NGQ-VRASAI-AQDADQ--- 191
Mus musculus Adil               167 -TCNIPGTYFFTYHVLMRGGDGTSMW-ADL-C----K---NGQ-VRASAI-AQDADQ--- 211
Rattus norvegicus Adil          167 -TCNIPGTYFFTYHVLMRGGDGTSMW-ADL-C----K---NGQ-VRASAI-AQDADQ--- 211
Homo sapiens ADIM               267 GA-PVSGVYFFTFSM-MKHEDVEEVY------VYLMH---NGN-TVFSMY--SYEM---- 308
Pan troglodytes ADIM            267 GA-PVSGVYFFTFSM-MKHEDVEEVY------VYLMH---NGN-TVFSMY--SYEM---- 308
```

```
Pongo abelii ADIM                 223 GA-PVSGVYFFTFSM-MKHEDVEEVY------VYLMH---NGN-TVFSMY--SYEM---- 264
Nomascus leucogenys ADIM          267 GA-PVSGVYFFTFSM-MKHEDVEEVY------VYLMH---NGN-TVFSMY--SYEM---- 308
Papio hamadryas ADIM              267 GA-PVSGVYFFTFSM-MKHEDVEEVY------VYLMH---NGN-TVFSMY--SYEM---- 308
Callithrix jacchus ADIM           257 GA-PVSGVYFFTFSM-MKHEDVEEVY------VYLMH---NGN-TVFSMY--SYET---- 298
Mus musculus Adim                 156 GA-PVSGVYFFTFSM-MKHEDVEEVY------VYLMH---NGN-TVFSMY--SYET---- 197
Rattus norvegicus Adim            230 GA-PVSGVYFFTFSM-MKHEDVEEVY------VYLMH---NGN-TVFSMY--SYET---- 271
Cavia porcellus ADIM              223 GA-PVSGVYFFTFSM-MKHEDVDEVY------VYLMH---NGN-TVFSMY--SYET---- 264
Bos taurus ADIM                   156 GA-PVSGVYFFTFSM-MKHEDVEEVY------VYLMH---NGN-TVFSMY--SYET---- 197
Equus caballus ADIM               156 GA-PVSGVYFFTFSM-MKHEDVEEVY------VYLMH---NGN-TVFSMY--SYET---- 197
Canis lupus familiaris ADIM       217 GA-PVSGVYFFTFSM-MKHEDVEEVY------VYLMH---NGN-TVFSMY--SYET---- 258
Homo sapiens ADIN                 222 -RCRLPGAYFFSFTLG-KLPRK-TLS------VKLMK---NRDEVQAMIY--DDGAS--- 264
Pan troglodytes ADIN              222 -RCRLPGAYFFSFTLG-KMPRK-TLS------VKLMK---NRDEVQAMIY--DDGAS--- 264
Pongo abelii ADIN                 222 -RCRLPGAYFFSFTLG-KLPRK-TLS------VKLMK---NRDEVQAMIY--DDGAS--- 264
Nomascus leucogenys ADIN          217 -RCRLPGAYFFSFTLG-KLPRK-TLS------VKLMK---NRDEVQAMIY--DDGAS--- 259
Macaca mulatta ADIN               224 -RCRLPGAYFFSFTLG-KLPRK-TLS------VKLMK---NRDEVQAMIY--DDGAS--- 266
Callithrix jacchus ADIN           222 -RCRLPGAYFFSFTLG-KLPRK-TLS------VKLMK---NRDEVQAMIY--DDGAS--- 264
Mus musculus Adin                 220 -RCRLPGAYFFSFTLG-KLPRK-TLS------VKLMK---NRDEVQAMIY--DDGAS--- 262
Cavia porcellus ADIN              232 -RCRLPGAYFFSFTLG-KMPRK-TLS------VKLMK---NRDEVQAMIY--DDGAS--- 274
Tursiops truncatus ADIN           218 -RCRLPGAYFFSFTLG-KLPRK-TLS------VKLMK---NRDEVQAMIY--DDGTS--- 260
Canis lupus familiaris ADIN       218 -RCRLPGAYFFSFTLG-KLPRK-TLS------VKLMK---NRDEVQAMIY--DDGAS--- 260
Dasypus novemcinctus ADIN         218 -RCRLPGAYFFSFTLG-KLPRK-TLS------VKLMK---NRDEVQAMIY--DDGAS--- 260
Loxodonta africana ADIN           218 -RCRLPGAYFFSFTLG-KLPRK-TLS------VKLMK---NRDEVQAMIY--DDGGS--- 260
Homo sapiens ADIO                 157 -LCTVPGVYFLSLNVH-TWNYKET-Y------LHIML---NRRPA-AVLY-AQPSE---- 198
Pan troglodytes ADIO              157 -LCTVPGVYFLSLNVH-TWNYKET-Y------LHVML---NRRPA-AVLY-AQPSE---- 198
Macaca mulatta ADIO               152 -LCTVPGVYFLSLNVH-TWNYKET-Y------LHIML---NRRPA-AVLY-AQPSE---- 193
Bos taurus ADIO                   156 -FCTAPGVYFLSLNVH-TWNYKET-Y------LHIMR---NARAA-AVLY-AQPSE---- 197
Myotis lucifugus ADIO             156 -LCIVPGVYFLSLTVH-TWNYKET-Y------LHIMC---NQQAT-AVLY-AQPSE---- 197
Homo sapiens ADIP                 284 -YCYVPGLYFFSLNVH-TWNQKET-Y------LHIMK---NEEEV-VILF-AQVGD---- 325
Otolemur garnettii ADIP           186 -YCYVPGIYFFSLNVH-TWNQKET-Y------LHIMR---NAEEV-VILY-AQVSD---- 227
Mus musculus Adip                 186 -YCYVPGIYFFSLNVH-TWNQKET-Y------LHIMK---NEEEV-VILY-AQVSD---- 227
Cavia porcellus ADIP              186 -YCYVPGIYFFSLNVH-TWNQKET-Y------LHIMR---NAEEV-VILY-AQVSD---- 227
Bos taurus ADIP                   209 -YCYVPGIYFFSLNVH-TWNQKET-Y------LHIMK---NAEEV-VILY-AQVSD---- 250
Equus caballus ADIP               184 -YCYVPGIYFFSLNVH-TWNQKET-Y------LHIMK---NGEEV-VILY-AQVSD---- 225
Loxodonta africana ADIP           238 -YCYVPGIYFFSLNVH-TWNQKET-Y------LHIMR---NAEEA-VILY-AQVSD---- 279
Homo sapiens ADIQ                 184 AA-PLRGIYFFSLNVH-SWNYKET-Y------VHIMH---NQKEA-VILY-AQPSE---- 225
Pan troglodytes ADIQ              184 AA-PLRGIYFFSLNVH-SWNYKET-Y------VHIMH---NQKEA-VILY-AQPSE---- 225
Pongo abelii ADIQ                 184 AA-PLRGIYFFSLNVH-SWNYKET-Y------VHIMH---NQKEA-VILY-AQPSE---- 225
Nomascus leucogenys ADIQ          184 AA-PLRGIYFFSLNVH-SWNYKET-Y------VHIMH---NQKEA-VILY-AQPSE---- 225
Macaca mulatta ADIQ               184 AA-PLRGIYFFSLNVH-SWNYKET-Y------VHIMH---NQKEA-VILY-AQPSE---- 225
Mus musculus Adiq                 170 VA-PLRGLYFFSLNVH-SWNYKET-Y------VHIVH---NEQAV-VILY-AQPSE---- 211
Rattus norvegicus Adiq            169 VA-PLRGLYFFSLNVH-SWNYKET-Y------VHIVH---NEQAV-VILY-AQPSE---- 210
Homo sapiens ADIR                  69 -NCPVNGTYVFIFHML-KLAVNVPLY------VNLMK---N-EEVLVSAY-ANDGAP---  112
Pan troglodytes ADIR               69 -NCPVNGTYVFIFHML-KLAVNVPLY------VNLMK---N-EEVLVSAY-ANDGAP---  112
Pongo abelii ADIR                  69 -NCPVNGTYVFIFHML-KLAVNVPLY------VNLMK---N-EEVLVSAY-ANDGAP---  112
Nomascus leucogenys ADIR           69 -NCPVNGTYVFIFHML-KLAVNVPLY------VNLMK---N-EEVLVSAY-ANDGAP---  112
Macaca mulatta ADIR                69 -NCPVNGTYVFIFHML-KLAVNVPLY------VNLMK---N-EEVLVSAY-ANDGAP---  112
Papio hamadryas ADIR               69 -NCPVNGTYVFIFHML-KLAVNVPLY------VNLMK---N-EEVLVSAY-ANDGAP---  112
Callithrix jacchus ADIR            69 -NCPVNGTYVFIFHML-KLAVNVPLY------VNLMK---N-EEVLVSAY-ANDGAP---  112
Tarsius syrichta ADIR              69 -NCPVNGTYVFIFHML-KLAVNVPLY------VNLMK---N-EEVLVSAY-ANDGAP---  112
Otolemur garnettii ADIR            69 -NCPVNGTYVFIFHML-KLAVNVPLY------VNLMK---N-EEVLVSAY-ANDGAP---  112
Tupaia belangeri ADIR              69 -NCPVNGTYVFIFHML-KLAVNVPLY------VNLMK---N-EEVLVSAY-ANDGAP---  112
Mus musculus Adir                  69 -NCPVNGTYVFIFHML-KLAVNVPLY------VNLMK---N-EEVLVSAY-ANDGAP---  112
Rattus norvegicus Adir             69 -NCPVNGTYVFIFHML-KLAVNVPLY------VNLMK---N-EEVLVSAY-ANDGAP---  112
Cavia porcellus ADIR               69 -SCPVNGTYVFIFHML-KLAVNVPLY------VNLMK---N-EEVLVSAY-ANDGAP---  112
Oryctolagus cuniculus ADIR         69 -NCPVNGTYVFIFHML-KLAVNVPLY------VNLMK---N-EEVLVSAY-ANDGAP---  112
Tursiops truncatus ADIR            69 -NCPVNGTYVFIFHML-KLAVNVPLY------VNLMK---N-EEVLVSAY-ANDGAP---  112
Bos taurus ADIR                    69 -NCPVNGTYVFIFHML-KLAVNVPLY------VNLMK---N-EEVLVSAY-ANDGAP---  112
Equus caballus ADIR                69 -NCPVNGTYVFIFHML-KLAVNVPLY------VNLMK---N-EEVLVSAY-ANDGAP---  112
Canis lupus familiaris ADIR        69 -NCPVNGTYVFIFHML-KLAVNVPLY------VNLMK---N-EEVLVSAY-ANDGAP---  112
Myotis lucifugus ADIR              69 -NCPVNGTYVFIFHML-KLAVNVPLY------VNLMK---N-EEVLVSAY-ANDGAP---  112
Pteropus vampyrus ADIR             69 -NCPVNGTYVFIFHML-KLAVNVPLY------VNLMK---N-EEVLVSAY-ANDGAP---  112
Erinaceus europaeus ADIR           43 -ACPVNGTYVFIFHML-KLAVNVPLY------VNLMR---N-EEVLVSAY-ANDGAP---   86
Dasypus novemcinctus ADIR          69 -NCPVNGTYVFIFHML-KLAVNVPLY------VNLMK---N-EEVLVSAY-ANDGAP---  112
Choloepus hoffmanni ADIR           69 -NCPVNGTYVFIFHML-KLAVNVPLY------VNLMK---N-EEVLVSAY-ANDGAP---  112
Loxodonta africana ADIR            69 -NCPVNGTYVFIFHML-KLAVNVPLY------VNLMK---N-EEVLVSAY-ANDGAP---  112
```

```
Homo sapiens ADIA                 203 --NTFQVTTGGMVLKLEQGENVFLQA--TD--KNSLL--GMEGANSIFSGFLLFPD-MEA 253
                                             790       800       810       820       830       840
Homo sapiens ADIA                 203 --NTFQVTTGGMVLKLEQGENVFLQA--TD--KNSLL--GMEGANSIFSGFLLFPD-MEA 253
Papio hamadryas ADIA              203 --NTFQVTTGGMVLKLEQGENVFLQA--TD--KNSLL--GMEGANSIFSGFLLFPD-VEA 253
Mus musculus Adia                 232 --NTFQVTTGGVVLKLEQEEVVHLQA--TD--KNSLL--GIEGANSIFTGFLLFPD-MDA 282
Rattus norvegicus Adia            203 --NTFQVTTGGVVLKLEQEEVVHLQA--TD--KNSLL--GVEGANSIFTGFLLFPD-MDV 253
Cavia porcellus ADIA              201 --NSFQVTTGGVVLKLEREEMVYLEA--TN--QNALL--GIEGANSIFSGFLLFPD-VEL 251
Ochotona princeps ADIA            201 --NTFQVTTGGVVLKLEMGESVHLQT--ND--KNSLL--AMEGANSIFSGFLLFPD-ADV 251
Bos taurus ADIA                   197 --NTFQVTTGSIVLKLEKDETVFLQA--TE--KNALV--GIEGANSIFSGFMLFPD-TEA 247
Equus caballus ADIA               202 --NTFQVTTGGIVLKLELEETVFLQA--TD--RNSLL--GMEGANSIFSGFLLFPD-REA 252
Canis lupus familiaris ADIA       200 --NTFQVTTGSVVLKLSQGENVYLQA--TD--KNSLL--GMEGANSIFSGFLLFPD-AEA 250
Felis catus ADIA                  204 --NTFQVSTGSMVLKLKQGENVFLQA--TD--KNSLV--GMEGANSIFSGFLLFPD-VEA 254
Myotis lucifugus ADIA             201 --STFQVTTGGIVLKLGLGESVFLQA--TD--RNSLL--GIEGANSIFSGFLLFPN-PEV 251
Dasypus novemcinctus ADIA         198 --SSFQVTTGGVVLKLGLGENVFLQA--TD--RNSLL--GLDGANSIFSGFLLFPD-AEA 248
Loxodonta africana ADIA           201 --STFQVTMGSVVLKLAQGENVFLQA--TD--KNSLL--GMEGANSIFSGFLLFPD-AEA 251
Homo sapiens ADIB                 198 --KTNQVNSGGVLLRLQVGEEVWLAV--ND--YYDMV--GIQGSDSVFSGFLLFPD---- 245
Pan troglodytes ADIB              198 --KTNQVNSGGVLLRLQVGEEVWLAV--ND--YYEMV--GIQGSDSVFSGFLLFPD---- 245
Pongo abelii ADIB                 198 --KTNQVNSGGVLLRLQVGEEVWLAV--ND--YYDMV--GIQGSDSVFSGFLLFPD---- 245
Nomascus leucogenys ADIB          199 --KTNQVNSGGVLLRLQVGEEVWLAV--ND--YYEMV--GIQGSDSVFSGFLLFPD---- 246
Callithrix jacchus ADIB           197 --NTKQVSSGGVLLRLQVGEEVWLAV--ND--YNGMV--GIEGSDSVFSGFLLFPD---- 244
Otolemur garnettii ADIB           196 --NSKQVSSGGVLLRVQVGDEVWLAV--ND--YNGMV--GTEGSDSVFSGFLLFPD---- 243
Mus musculus Adib                 229 --NSKQVSSGGVLLRLQRGDEVWLSV--ND--YNGMV--GIEGSNSVFSGFLLFPD---- 276
Rattus norvegicus Adib            221 --NSKQVSSGGVLLRLQRGDEVWLAV--ND--YNGMV--GTEGSDSVFSGFLLFPD---- 268
Cavia porcellus ADIB              248 SQDSKQVTSGGVVLHLQQGAQVWLGV--ND--YNGMV--GTQGSDSVFSGFLLFPD---- 297
Oryctolagus cuniculus ADIB        251 --NTKQVSSGGVLLRLQVGDEVWLGV--ND--YNGMV--GTEGSDSVFSGFLLFPD---- 298
Bos taurus ADIB                   196 --SAKQVSSGGVLLRLQEGQQVWLAV--ND--YNGMV--GMEGSDSVFSGFLLFPD---- 243
Equus caballus ADIB               216 --NSKQVSSGGVLLQLQVGEEVWLAV--ND--YNGMV--GTRGSDSVFSGFLLFPD---- 263
Canis lupus familiaris ADIB       198 --NSKQVSSGGVLLRLQMGEQVWLAV--ND--YNGMV--GTEGSDSVFSGFLLFPD---- 245
Myotis lucifugus ADIB             198 --NSKQVSSGGVLLRLQEGAQVWLRV--ND--YNGMV--GTEGSDSVFSGFLLFPD---- 245
Loxodonta africana ADIB           196 --NKKQVSSGGVLLRLQLGDEVWLAV--ND--YNGMV--GTEGSDSVFSGFLLFPD---- 243
Homo sapiens ADIC                 195 -KGLFQVVSGGMVLQLQQGDQVWVE---KDP-KKGHIYQGSE-ADSVFSGFLIFP--S-A 245
Macaca mulatta ADIC               195 -KGLFQVVSGGMVLQLQRGDQVWVE---KDP-RKGNIYQGLE-ADSVFSGFLIFP--S-T 245
Papio hamadryas ADIC              195 -KGLFQVVSGGMVLQLQRGDQVWVE---KDP-RKGNIYHGLE-ADSVFSGFLIFP--S-T 245
Callithrix jacchus ADIC           195 -KGIFQVVSGGTVLHLQRGDQVWIE---KDP-SKGRIYQGSE-ADSVFSGFLIFP--S-A 245
Mus musculus Adic                 195 -KGLFQVLAGGTVLQLRRGDEVWIE---KDP-AKGRIYQGTE-ADSIFSGFLIFP--S-A 245
Rattus norvegicus Adic            207 -KGLFQVLAGGTVLQLQRGDEVWIE---KDP-AKGRIYQGTE-ADSIFSGFLIFP--S-A 257
Cavia porcellus ADIC              195 -KGLFQ-ISGGTSLQLQQGDKVWIE---RDL-SKGNIFQGSE-ADSVFSGFLIFP--S-V 244
Oryctolagus cuniculus ADIC        236 -KGIFQVVSGGTALQLQRGDQVWIE---KDP-ARGRIYQGTE-ADSVFSGFLIFP--S-A 286
Equus caballus ADIC               228 -RGIFQVVSGGTVLQLDRGDKVRIE---KE---RGRIYQGSE-ADSVFSGFLIFP--S-T 276
Canis lupus familiaris ADIC       214 -KGIFQVVSGGMALQLQQGDQVWIE---KDP-IKGRIYQGPE-ADSIFSGFLIFP--S-L 264
Myotis lucifugus ADIC             195 -RGTFQVVSGGTVLQLQDGDQVWIE---KDP-VKGRIYQGPE-ADSVFSGFLIFP--S-A 245
Loxodonta africana ADIC           210 -RGLFQVVSGGTVLQLQHGDQVWIE---KDP-VKGHIYQGTD-ADSIFSGFLIFP--S-T 260
Homo sapiens ADID                 223 --NNVDQASGSVLLHLEVGDQVWLQVYGEGE-RNG-LYAD-NDNDSTFTGFLLYHD-TN- 275
Pan troglodytes ADID              223 --NNVDQASGSVLLHLEVGDQVWLQVYGEGE-RNG-LYAD-NDNDSTFTGFLLYHD-TN- 275
Pongo abelii ADID                 192 --NNVDQASGSVLLHLEVGDQVWLQVYGEGE-RNG-LYAD-NDNDSTFTGFLLYHD-TN- 244
Nomascus leucogenys ADID          222 --NNVDQASGSVLLHLEVGDQVWLQVYGEGE-RNG-LYAD-NDNDSTFTGFLLYHD-TN- 274
Macaca mulatta ADID               221 --NNVDQASGSVLLHLEVGDQVWLQVYGEGE-RNG-LYAD-NDNDSTFTGFLLYHD-TN- 273
Otolemur garnettii ADID           192 --KNVDQASGSVLLHLEVGDQVWLQLYGDGE-HNG-LYAD-NVNDSTFTGFLLYHD-TN- 244
Mus musculus Adid                 206 --KNVDQASGSVLLHLEVGDQVWLQVYGDGD-HNG-LYAD-NVNDSTFTGFLLYHD-TN- 258
Rattus norvegicus Adid            192 --KNVDQASGSMLLHLEVGDQVWLQVYGEGD-NNG-LYAD-NVNDSTFTGFLLYHD-TN- 244
Cavia porcellus ADID              234 --KNVDQASGSVLLHLSVGDQVWLQVYGNGE-QAG-VYAD-NVNDSTFTGFLLYHDLTDP 288
Oryctolagus cuniculus ADID        202 --KNVDQASGSVLLHLQVDDQVWLQVYGDGD-HNG-LYAD-NVNDSIFTGFLLYHD-TE- 254
Bos taurus ADID                   187 --KNVDQASGSVLLYLEKGDQVWLQVYEGEN-HNG-VYAD-NVNDSTFTGFLLYHNIVE- 240
Equus caballus ADID               249 --KNLDQASGSVLLYLEKGDQVWLQVYGDGD-HNG-LYAD-NVNDSTFTGFLLYHD-TN- 301
Canis lupus familiaris ADID       201 --KNVDQASGSVLLHLEVGDQVWLQVYGDGD-SYG-IYAD-NVNDSTFTGFLLYHD-TN- 253
Felis catus ADID                  192 --KNVDQASGSVLLHLETGDEVWLQVYGDGD-YNG-LYAD-NVNDSTFTGFLLYYD-TV- 244
Myotis lucifugus ADID             201 --QDVDQASGSVLLHLEMGDQVWLQVYGEGE-NNG-LYAD-NINDSTFTGFLLYHD-TN- 253
Dasypus novemcinctus ADID         191 --KNVDQASGSVLLHLEMGDQVWLQVYESGE-QMG-LYAD-NVNDSTFTGFLLYHD-IE- 243
Loxodonta africana ADID           192 --KNVDQASGSVLLHLEVGDQVWLQVFADGEQNNG-LYAD-NVNDSTFMGFLLYHD-TN- 245
Homo sapiens ADIE                 186 --PKPASLSGGAMVRLEPEDQVWVQVGVGD--YIG-IYAS-IKTDSTFSGFLVYSD-WHS 238
Pan troglodytes ADIE              186 --PKPASLSGGAMVRLEPEDQVWVQVGVGD--YIG-IYAS-IKTDSTFSGFLVYSD-WHS 238
Pongo abelii ADIE                 186 --PKPASLSGGAMVRLEPEDQVWVQVGVGD--YIG-IYAS-IKTDSTFSGFLVYSD-WHS 238
Papio hamadryas ADIE              186 --PKPASLSGGAMVRLEPEDQVWVQVGVGD--YIG-IYAS-IKTDSTFSGFLVYSD-WHS 238
Callithrix jacchus ADIE           186 --PKPASLSGGAMVRLEPEDQVWVQVGVGD--YIG-IYAS-IKTDSTFSGFLVYSD-WHS 238
Otolemur garnettii ADIE           186 --PKPASLSGGAMVRLEPEDQVWVQVGVGD--YIG-IYAS-IKTDSTFSGFLVYSD-WHS 238
Mus musculus Adie                 186 --PKPASLSGGAMVRLEPEDQVWVQVGVGD--YIG-IYAS-IKTDSTFSGFLVYSD-WHS 238
Rattus norvegicus Adie            186 --PKPASLSGGAMVRLEPEDQVWVQVGVGD--YIG-IYAS-IKTDSTFSGFLVYSD-WHS 238
Homo sapiens ADIF1                282 --SSEDQASGGIVLQLKLGDEVWLQVTGGER-FNG-LFAD-EDDDTTFTGFLLF---SSP 333
Homo sapiens ADIF2                282 --SSEDQASGSIVLQLKLGDEMWLQVTGGER-FNG-LFAD-EDDDTTFTGFLLF---SSQ 333
Pan troglodytes ADIF1             282 --SSEDQASGGIVLQLKLGDEVWLQVTGGER-FNG-LFAD-EDDDTTFTGFLLF---SSP 333
Pongo abelii ADIF1                282 --SSEDQASGGIVLQLKLGDEVWLQVTGGER-FNG-LFAD-EDDDTTFTGFLLF---SSP 333
Nomascus leucogenys ADIF1         282 --SSEDQASGGIVLQLKLGDEVWLQVTGGER-FNG-LFAD-EDDDTTFTGFLLF---SSP 333
Macaca mulatta ADIF1              282 --SSEDQASGGLVLQLKLGDEVWLQVTGGDR-FNG-LFAD-EDDDTTFTGFLLF---GSP 333
Papio hamadryas ADIF1             282 --SSEDQASGGIVLQLKLGDEVWLQVTGGER-FNG-LFAD-EDDDTTFTGFLLF---SSP 333
```

```
Microcebus murinus ADIF1          282 --SSEDQASGGIVLQLKLGDEVWMQVTGGER-FNG-LFAD-EDDDTTFTGFLLF---SS- 332
Otolemur garnettii ADIF1          282 --GSEDQASGGIVLKLKLGDEVWMQVTGGER-FNG-LFAD-EDDDTTFTGFLLF---SS- 332
Mus musculus Adif1                311 --SSEDQASGGIVQELKLGDEVWMQVTGGER-FNG-LFAD-EDDDTTFTGFLLF---SSS 362
Rattus norvegicus Adif1           298 --SSEDQASGGIVLELKLGDEVWMQVTGGER-FNG-LFAD-EDDDTTFTGFLLF---SSS 349
Cavia porcellus ADIF1             282 --SSEDQASGGTVLPLKLGDEVWLQVTGGER-FNG-LFAD-EDDDTTFTGFLLF---SSP 333
Oryctolagus cuniculus ADIF1       282 --SSEDQASGGIVLELKLGDEVWMQVTGGER-FNG-LFAD-EDDDTTFTGFLLF---SS- 332
Ochotona princeps ADIF1           282 --SSEDQASGGVVLELKLADEVWLQVAGGER-FNG-LFAD-EDDDTTFTGFLLF---ST- 332
Bos taurus ADIF1                  282 --GSEDQASGGIVLPLKLGDEVWLQVTGGER-FNG-LFAD-KDDDTTFTGFLLF---SSS 333
Equus caballus ADIF1              282 --SSEDQASGGTILQLKLGDEVWVQVTGGER-FNG-LFAD-EDDDTTFTGFLLF---SSS 333
Canis lupus familiaris ADIF1      282 --SSEDQASGGIVLPLKLGDEVWMQVTGGER-FNG-LFAD-EDDDTTFTGFLLF---SSQ 333
Felis catus ADIF1                 282 --SSEDQASGGIVLPLKLGDEVWMQVAGGER-FNG-LFAD-EDDDTTFTGFLLF---S-Q 332
Dasypus novemcinctus ADIF1        283 --SSEDQASGGMVLELKLGDEVWLQASGGER-FNG-LFAD-EDDDTTFTGFLLF---SS- 333
Loxodonta africana ADIF1          282 --SSEDQASGGTVLELKLGDEVWMQVTGGER-FNG-LFAD-EDDDTTFTGFLLF---TSS 333
Homo sapiens ADIG                 281 ---NHDVASGSTVIYLQPEDEVWLEIFFTD--QNG-LFSDPGWADSLFSGFLLYVD-TDY 333
Pan troglodytes ADIG              281 ---NHDVASGSTVIYLQPEDEVWLEIFFTD--QNG-LFSDPGWADSLFSGFLLYVD-TDY 333
Pongo abelii ADIG                 281 ---NHDVASGSTVIYLQPEDEVWLEIFFTD--QNG-LFSDPGWADSLFSGFLLYVD-TDY 333
Callithrix jacchus ADIG           267 ---NHDVASGSTVIYLQPEDEVWLEIFFTD--QNG-LFSDPGWADSLFSGFLLYVD-TDY 319
Otolemur garnettii ADIG           227 ---NHDVASGSTVIYLQPEDEVWLEIFFTD--QNG-LFSDPGWADSLFSGFLLYVD-TDY 279
Mus musculus Adig                 269 ---NHDVASGSTVIYLQPEDEVWLEIFFND--QNG-LFSDPGWADSLFSGFLLYVD-TDY 321
Rattus norvegicus Adig            270 ---NHDVASGSTVIYLQPEDEVWLEIFFND--QNG-LFSDPGWADSLFSGFLLYVD-TDY 322
Cavia porcellus ADIG              227 ---NHDVASGSTVIYLQPEDEVWLEIFFTD--QNG-LFSDPGWADSLFSGFLLYVD-TDY 279
Oryctolagus cuniculus ADIG        227 ---NHDVASGSTVIYLQPEDEVWLEIFFTD--QNG-LFSDPGWADSLFSGFLLYVD-TDY 279
Tursiops truncatus ADIG           279 ---NHDVASGSTVIYLQPEDEVWLEIFFTD--QNG-LFSDPGWADSLFSGFLLYVD-TDY 331
Bos taurus ADIG                   227 ---NHDVASGSTVIYLQPEDEVWLEIFFTD--QNG-LFSDPGWADSLFSGFLLYVD-TDY 279
Equus caballus ADIG               268 ---NHDVASGSTVIYLQPEDEVWLEIFFTD--QNG-LFSDPGWADSLFSGFLLYVD-TDY 320
Canis lupus familiaris ADIG       268 ---NHDVASGSTVIYLQPEDEVWLEIFFTD--QNG-LFSDPGWADSLFSGFLLYVD-TDY 320
Myotis lucifugus ADIG             227 ---NHDVASGSTVIYLQPEDEVWLEIFFND--QNG-LFSDPGWADSLFSGFLLYVD-TDY 279
Homo sapiens ADIH                 229 ---NHDVASGSTILALKQGDEVWLQIFYSE--QNG-LFYDPYWTDSLFTGFLIYAD-QD- 280
Pan troglodytes ADIH              229 ---NHDVASGSTILALKQGDEVWLQIFYSE--QNG-LFYDPYWTDSLFTGFLIYAD-QD- 280
Pongo abelii ADIH                 229 ---NHDVASGSTILALKQGDEVWLQIFYSE--QNG-LFYDPYWTDSLFTGFLIYAD-QD- 280
Nomascus leucogenys ADIH          229 ---NHDVASGSTILALKQGDEVWLQIFYSE--QNG-LFYDPYWTDSLFTGFLIYAD-QD- 280
Macaca mulatta ADIH               229 ---NHDVASGSTILALKQGDEVWLQIFYSE--QNG-LFYDPYWTDSLFTGFLIYAD-QD- 280
Papio hamadryas ADIH              229 ---NHDVASGSTILALKQGDEVWLQIFYSE--QNG-LFYDPYWTDSLFTGFLIYAD-QD- 280
Mus musculus Adih                 236 ---NHDVASGSTILALKEGDEVWLQIFYSE--QNG-LFYDPYWTDSLFTGFLIYAD-QG- 287
Rattus norvegicus Adih            273 ---NHDVASGSTILALKEGDEVWLQIFYSE--QNG-LFYDPYWTDSLFTGFLIYAD-QG- 324
Dipodomys ordii ADIH              229 ---NHDIASGSTILALKQDDEVWLQIFYSE--QNG-LFYDPYWTDSLFTGFLIYAD-QG- 280
Cavia porcellus ADIH              229 ---NHDVASGSTILALKQGDEVWLQIFYSE--QNG-LFYDPYWTDSLFTGFLIYAD-QD- 280
Bos taurus ADIH                   246 ---NHDVASGSTILALRQGDEVWLQIFYSE--QNG-LFYDPYWTDSLFTGFLIYAD-QD- 297
Equus caballus ADIH               229 ---NHDVASGSTILALKQGDEVWLQIFYSE--QNG-LFYDPYWTDSLFTGFLIYAD-QD- 280
Canis lupus familiaris ADIH       229 ---NHDVASGSTILALKQGDEVWLQIFYSE--QNG-LFYDPYWTDSLFTGFLIYAD-QD- 280
Pteropus vampyrus ADIH            229 ---NHDVASGSTILALKQGDEVWLQIFYSE--QNG-LFYDPYWTDSLFTGFLIYAD-QD- 280
Loxodonta africana ADIH           229 ---NHDVASGSTILALKQGDEVWLQIFYSE--QNG-LFYDPYWTDSLFTGFLIYAD-QD- 280
Homo sapiens ADII                 241 ---NYDYASNSVVLHLDSGDEVYVKL---D---GGKAHGGNNNKYSTFSGFLLYPD---- 287
Pongo abelii ADII                 241 ---NYDYASNSVVLHLDSGDEVYVKL---D---GGKAHGGNNNKYSTFSGFLLYPD---- 287
Macaca mulatta ADII               241 ---NYDYASNSVVLHLDSGDEVYVKL---D---GGKAHGGNNNKYSTFSGFLLYPD---- 287
Mus musculus Adii                 241 ---NYDYASNSVVLHLDSGDEVYVKL---D---GGKAHGGNNNKYSTFSGFLLYPD---- 287
Rattus norvegicus Adii            241 ---NYDYASNSVVLHLDSGDEVYVKL---D---GGKAHGGNNNKYSTFSGFLLYPD---- 287
Bos taurus ADII                   241 ---NYDYASNSVVLHLDSGDEVYVKL---D---GGKAHGGNNNKYSTFSGFLLYPD---- 287
Canis lupus familiaris ADII       241 ---NYDYASNSVVLHLDSGDEVYVKL---D---GGKAHGGNNNKYSTFSGFLLYPD---- 287
Dasypus novemcinctus ADII         241 ---NYDYASNSVVLHLDSGDEVYVKL---D---GGKAHGGNNNKYSTFSGFLLYPD---- 287
Homo sapiens ADIJ                 209 ---NYDYASNSVVLHLEPGDEVYIKL---D---GGKAHGGNNNKYSTFSGFIIYAD---- 255
Pan troglodytes ADIJ              209 ---NYDYASNSVVLHLEPGDEVYIKL---D---GGKAHGGNNNKYSTFSGFIIYAD---- 255
Papio hamadryas ADIJ              209 ---NYDYASNSVVLHLEPGDEVYIKL---D---GGKAHGGNNNKYSTFSGFIIYAD---- 255
Mus musculus Adij                 209 ---NYDYASNSVVLHLEPGDEVYIKL---D---GGKAHGGNNNKYSTFSGFIIYAD---- 255
Rattus norvegicus Adij            209 ---NYDYASNSVVLHLEPGDEVYIKL---D---GGKAHGGNNNKYSTFSGFIIYAD---- 255
Oryctolagus cuniculus ADIJ        209 ---NYDYASNSVVLHLEPGDEVYIKL---D---GGKAHGGNNNKYSTFSGFIIYAD---- 255
Bos taurus ADIJ                   209 ---NYDYASNSVVLHLEPGDEVYIKL---D---GGKAHGGNNNKYSTFSGFIIYAD---- 255
Canis lupus familiaris ADIJ       209 ---NYDYASNSVVLHLEPGDEVYIKL---D---GGKAHGGNNNKYSTFSGFIIYAD---- 255
Loxodonta africana ADIJ           209 ---NYDYASNSVVLHLEPGDEVYIKL---D---GGKAHGGNNNKYSTFSGFIIYAD---- 255
Homo sapiens ADIK                 192 ---NYDYASNSVILHLDVGDEVFIKL---D---GGKVHGGNTNKYSTFSGFIIYPD---- 238
Pan troglodytes ADIK              192 ---NYDYASNSVILHLDVGDEVFIKL---D---GGKVHGGNTNKYSTFSGFIIYPD---- 238
Gorilla gorilla ADIK              192 ---NYDYASNSVILHLDVGDEVFIKL---D---GGKVHGGNTNKYSTFSGFIIYPD---- 238
Papio hamadryas ADIK              192 ---NYDYASNSVILHLDVGDEVFIKL---D---GGKVHGGNTNKYSTFSGFIIYPD---- 238
Callithrix jacchus ADIK           192 ---NYDYASNSVILHLDVGDEVFIKL---D---GGKVHGGNTNKYSTFSGFIIYPD---- 238
Rattus norvegicus Adik            192 ---NYDYASNSVILHLDVGDEVFIKL---D---GGKVHGGNTNKYSTFSGFIIYPD---- 238
Cavia porcellus ADIK              192 ---NYDYASNSVILHLDVGDEVFIKL---D---GGKVHGGNTNKYSTFSGFIIYPD---- 238
Equus caballus ADIK               192 ---NYDYASNSVILHLDVGDEVFIKL---D---GGKVHGGNTNKYSTFSGFIIYPD---- 238
Canis lupus familiaris ADIK       192 ---NYDYASNSVILHLDVGDEVFIKL---D---GGKVHGGNTNKYSTFSGFIIYPD---- 238
Dasypus novemcinctus ADIK         192 ---NYDYASNSVILHLDVGDEVFIKL---D---GGKVHGGNTNKYSTFSGFIIYPD---- 238
Loxodonta africana ADIK           192 ---NYDYASNSVILHLDVGDEVFIKL---D---GGKVHGGNTNKYSTFSGFIIYPD---- 238
Mus musculus Adil                 212 ---NYDYASNSVILHLDAGDEVFIKL---D---GGKAHGGNSNKYSTFSGFIIYSD---- 258
Rattus norvegicus Adil            212 ---NYDYASNSVILHLDAGDEVFIKL---D---GGKAHGGNSNKYSTFSGFIIYSD---- 258
Homo sapiens ADIM                 309 -KGKSDTSSNHAVLKLAKGDEVWLRM---G---NGALH-GDHQRFSTFAGFLLF-E-TK- 357
Pan troglodytes ADIM              309 -KGKSDTSSNHAVLKLAKGDEVWLRM---G---NGALH-GDHQRFSTFAGFLLF-E-TK- 357
```

```
Pongo abelii ADIM                265 -KGKSDTSSNHAVLKLAKGDEVWLRM---G---NGALH-GDHQRFSTFAGFLLF-E-TK- 313
Nomascus leucogenys ADIM         309 -KGKSDTSSNHAVLKLAKGDEVWLRM---G---NGALH-GDHQRFSTFAGFLLF-E-TK- 357
Papio hamadryas ADIM             309 -KGKSDTSSNHAVLKLAKGDEVWLRM---G---NGALH-GDHQRFSTFAGFLLF-E-TK- 357
Callithrix jacchus ADIM          299 -KGKSDTSSNHAVLKLAKGDEVWLRM---G---NGALH-GDHQRFSTFAGFLLF-E-TK- 347
Mus musculus Adim                198 -KGKSDTSSNHAVLKLAKGDEVWLRM---G---NGALH-GDHQRFSTFAGFLLF-E-TK- 246
Rattus norvegicus Adim           272 -KGKSDTSSNHAVLKLAKGDEVWLRM---G---NGALH-GDHQRFSTFAGFLLF-E-TK- 320
Cavia porcellus ADIM             265 -KGKSDTSSNHAVLKLAKGDEVWLRM---G---NGALH-GDHQRFSTFAGFLLF-E-TK- 313
Bos taurus ADIM                  198 -KGKSDTSSNHAVLKLAKGDEVWLRM---G---NGALH-GDHQRFSTFAGFLLF-E-TK- 246
Equus caballus ADIM              198 -KGKSDTSSNHAVLKLAKGDEVWLRM---G---NGALH-GDHQRFSTFAGFLLF-E-TK- 246
Canis lupus familiaris ADIM      259 -KGKSDTSSNHAVLKLAKGDEVWLRM---G---NGALH-GDHQRFSTFAGFLLF-E-TK- 307
Homo sapiens ADIN                265 --RRREMQSQSVMLALRRGDAVWLLSHDHD---GYGAYSN-HGKYITFSGFLVYPDLAPA 318
Pan troglodytes ADIN             265 --RRREMQSQSVMLALRRGDAVWLLSHDHD---GYGAYSN-HGKYITFSGFLVYPDLAPA 318
Pongo abelii ADIN                265 --RRREMQSQSVMLALRRGDAVWLLSHDHD---GYGAYSN-HGKYITFSGFLVYPDLAPA 318
Nomascus leucogenys ADIN         260 --RRREMQSQSVMLALRRGDAVWLLSHDHD---GYGAYSN-HGKYITFSGFLVYPDLAPA 313
Macaca mulatta ADIN              267 --RRREMQSQSVMLALRRGDAVWLLSHDHD---GYGAYSN-HGKYITFSGFLVYPDLAPA 320
Callithrix jacchus ADIN          265 --RRREMQSQSVMLALRRGDAVWLLSHDHD---GYGAYSN-HGKYITFSGFLVYPDLEST 318
Mus musculus Adin                263 --RRREMQSQSVMLPLRRGDAVWLLSHDHD---GYGAYSN-HGKYITFSGFLVYPDLAAA 316
Cavia porcellus ADIN             275 --RRREMQSQSVMLSLRRGDAVWLLSHDHD---GYGAYSN-HGKYITFSGFLVYPDLTPD 328
Tursiops truncatus ADIN          261 --RRREMQSQSVMLALRRGDAVWLLSHDHD---GYGAYSN-HGKYITFSGFLVYPDLIPA 314
Canis lupus familiaris ADIN      261 --RRREMQSQSVMLALRRGDAVWLLSHDHD---GYGAYSN-HGKYITFSGFLVYPDLAAT 314
Dasypus novemcinctus ADIN        261 --RRREMQSQSVMLALRRGDAVWLLSHDHD---GYGAYSN-HGKYITFSGFLVYPDLAPA 314
Loxodonta africana ADIN          261 --RRREMQSQSIMLALQRGDTVWLLSHDHD---GYGAYSN-HGKYITFSGFLVYPDPA-A 313
Homo sapiens ADIO                199 ---RSVMQAQSLMLLLAAGDAVWVRMFQRD--RDNAIYGEHGDLYITFSGHLVKP----A 249
Pan troglodytes ADIO             199 ---RSVMQAQSLMLLLAAGDAVWVRMFQRD--RDNAIYGEHGDLYITFSGHLVKP----A 249
Macaca mulatta ADIO              194 ---RSVMQAQSLMLLLAAGDAVWVRMFQRE--QDNAVYGERGDLYITFSGHLVKP----A 244
Bos taurus ADIO                  198 ---RSVMQAQSLLLPLAAGDAVWVRMFQRD--RDNAIFGESGDLYITFSGHLVKP----D 248
Myotis lucifugus ADIO            198 ---RSVMQTQSLLLPLAAGDTAWVRMFKRD--QDNAIYGEHGDLYITFSGHLVKP----A 248
Homo sapiens ADIP                326 ---RSIMQSQSLMLELREQDQVWVRLYKGE--RENAIFSEELDTYITFSGYLVKH----A 376
Otolemur garnettii ADIP          228 ---RSIMQSQSLLLQLREQDEVWVRLFRGE--RENAIFSDEFDTYITFSGYLVKH----A 278
Mus musculus Adip                228 ---RSIMQSQSLMMELREEDEVWVRLFKGE--RENAIFSDEFDTYITFSGYLVKP----A 278
Cavia porcellus ADIP             228 ---RSIMQSQSLMLELQEQDEVWVRLFKGE--RENAIFSDEFDTYITFSGYLVKH----A 278
Bos taurus ADIP                  251 ---RSIMQSQSLMLELRDQDEVWVRLFKGE--RENAIFSDEFDTYITFSGYLVKP----A 301
Equus caballus ADIP              226 ---RSIMQSQSLMLELQEQDEVWVRLFKGE--RENAIFSDEFDTYITFSGYLVKH----A 276
Loxodonta africana ADIP          280 ---RSIMQSQSLMLELREQDEVWVRLFKGE--RENAIFSDEFDTYITFSGYLVKH----A 330
Homo sapiens ADIQ                226 ---RSIMQSQSVMLDLAYGDRVWVRLFKRQ--RENAIYSNDFDTYITFSGHLIK-----A 275
Pan troglodytes ADIQ             226 ---RSIMQSQSVMLDLAYGDRVWVRLFKRQ--RENAIYSNDFDTYITFSGHLIK-----A 275
Pongo abelii ADIQ                226 ---RSIMQSQSVMLDLAYGDRVWVRLFKRQ--RENAIYSNDFDTYITFSGHLIK-----A 275
Nomascus leucogenys ADIQ         226 ---RSIMQSQSVMLDLAYGDRVWVRLFKRQ--RENAIYSNDFDTYITFSGHLIK-----A 275
Macaca mulatta ADIQ              226 ---RSIMQSQSVMLDLAYGDRVWVRLFKRQ--RENAIYSNDFDTYITFSGHLIK-----A 275
Mus musculus Adiq                212 ---RSIMQSQSVMLPLVPGDRVWVRLFKRE--RENGIYSDDVDTYITFSGHLIK-----A 261
Rattus norvegicus Adiq           211 ---RSIMQSQSVMLPLVPGDYVWVRLFKRE--RENGIYSDDMDTYITFSGHLIK-----A 260
Homo sapiens ADIR                113 ---DHETASNHAILQLFQGDQIWLRLHRGA---IYG--SS-W-KYSTFSGYLLYQD---- 158
Pan troglodytes ADIR             113 ---DHETASNHAILQLFQGDQIWLRLHRGA---IYG--SS-W-KYSTFSGYLLYQD---- 158
Pongo abelii ADIR                113 ---DHETASNHAILQLFQGDQIWLRLHRGA---IYG--SS-W-KYSTFSGYLLYQD---- 158
Nomascus leucogenys ADIR         113 ---DHETASNHAILQLFQGDQIWLRLHRGA---IYG--SS-W-KYSTFSGYLLYQD---- 158
Macaca mulatta ADIR              113 ---DHETASNHAILQLFQGDQIWLRLHRGA---IYG--SS-W-KYSTFSGYLLYQD---- 158
Papio hamadryas ADIR             113 ---DHETASNHAILQLFQGDQIWLRLHRGA---IYG--SS-W-KYSTFSGYLLYQD---- 158
Callithrix jacchus ADIR          113 ---DHETASNHAILQLFQGDQIWLRLHRGA---IYG--SS-W-KYSTFSGYLLYQD---- 158
Tarsius syrichta ADIR            113 ---DHETASNHAILQLFQGDQIWLRLHRGA---IYG--SS-W-KYSTFSGYLLYQD---- 158
Otolemur garnettii ADIR          113 ---DHETASNHAILQLFQGDQIWLRLHRGA---IYG--SS-W-KYSTFSGYLLYQD---- 158
Tupaia belangeri ADIR            113 ---DHETASNHAILQLFQGDQIWLRLHRGA---IYG--SS-W-KYSTFSGYLLYQD---- 158
Mus musculus Adir                113 ---DHETASNHAVLQLLQGDQIWLRLHRGA---IYG--SS-W-KYSTFSGYLLYQD---- 158
Rattus norvegicus Adir           113 ---DHETASNHAILQLLQGDQIWLRLHRGA---IYG--SS-W-KYSTFSGYLLYQD---- 158
Cavia porcellus ADIR             113 ---DHETASNHAVLQLFQGDQIWLRLHRGA---IYG--SS-W-KYSTFSGYLLYQD---- 158
Oryctolagus cuniculus ADIR       113 ---DHETASNHAILQLFQGDQIWLRLHRGA---IYG--SS-W-KYSTFSGYLLYQD---- 158
Tursiops truncatus ADIR          113 ---DHETASNHAILQLFQGDQIWLRLHRGA---IYG--SS-W-KYSTFSGYLLYQD---- 158
Bos taurus ADIR                  113 ---DHETASNHAILQLFQGDQIWLRLHRGA---IYG--SS-W-KYSTFSGYLLYQD---- 158
Equus caballus ADIR              113 ---DHETASNHAILQLFQGDQIWLRLHRGA---IYG--SS-W-KYSTFSGYLLYQD---- 158
Canis lupus familiaris ADIR      113 ---DHETASNHAILQLFQGDQIWLRLHRGA---IYG--SS-W-KYSTFSGYLLYQD---- 158
Myotis lucifugus ADIR            113 ---DHETASNHAILQLFQGDQIWLRLHRGA---IYG--SS-W-KYSTFSGYLLYQD---- 158
Pteropus vampyrus ADIR           113 ---DHETASNHAILQLFQGDQIWLRLHRGA---IYG--SS-W-KYSTFSGYLLYQD---- 158
Erinaceus europaeus ADIR          87 ---DHETASNHAVLQLCRGDQIWLRLHRGA---IYG--SS-W-KYSTFSGYLLYQD---- 132
Dasypus novemcinctus ADIR        113 ---DHETASNHAILQLFQGDQIWLRLHRGA---IYG--SS-W-KYSTFSGYLLYQD---- 158
Choloepus hoffmanni ADIR         113 ---DHETASNHAILQLFQGDQIWLRLHRGA---IYG--SS-W-KYSTFSGYLLYQD---- 158
Loxodonta africana ADIR          113 ---DHETASNHAILQLFQGDQIWLRLHRGA---IYG--SS-W-KYSTFSGYLLYQD---- 158
```

***Homo sapiens* ADIA**	-	**-----------&**	-
		850	
***Homo sapiens* ADIA**	-	-----------&	-
***Papio hamadryas* ADIA**	-	-----------&	-
***Mus musculus* Adia**	-	-----------&	-
***Rattus norvegicus* Adia**	-	-----------&	-
***Cavia porcellus* ADIA**	-	-----------&	-
***Ochotona princeps* ADIA**	-	-----------&	-
***Bos taurus* ADIA**	-	-----------&	-
***Equus caballus* ADIA**	-	-----------&	-
***Canis lupus familiaris* ADIA**	-	-----------&	-
***Felis catus* ADIA**	-	-----------&	-
***Myotis lucifugus* ADIA**	-	-----------&	-
***Dasypus novemcinctus* ADIA**	-	-----------&	-
***Loxodonta africana* ADIA**	-	-----------&	-
***Homo sapiens* ADIB**	-	-----------&	-
***Pan troglodytes* ADIB**	-	-----------&	-
***Pongo abelii* ADIB**	-	-----------&	-
***Nomascus leucogenys* ADIB**	-	-----------&	-
***Callithrix jacchus* ADIB**	-	-----------&	-
***Otolemur garnettii* ADIB**	-	-----------&	-
***Mus musculus* Adib**	-	-----------&	-
***Rattus norvegicus* Adib**	-	-----------&	-
***Cavia porcellus* ADIB**	-	-----------&	-
***Oryctolagus cuniculus* ADIB**	-	-----------&	-
***Bos taurus* ADIB**	-	-----------&	-
***Equus caballus* ADIB**	-	-----------&	-
***Canis lupus familiaris* ADIB**	-	-----------&	-
***Myotis lucifugus* ADIB**	-	-----------&	-
***Loxodonta africana* ADIB**	-	-----------&	-
***Homo sapiens* ADIC**	-	-----------&	-
***Macaca mulatta* ADIC**	-	-----------&	-
***Papio hamadryas* ADIC**	-	-----------&	-
***Callithrix jacchus* ADIC**	-	-----------&	-
***Mus musculus* Adic**	-	-----------&	-
***Rattus norvegicus* Adic**	-	-----------&	-
***Cavia porcellus* ADIC**	-	-----------&	-
***Oryctolagus cuniculus* ADIC**	-	-----------&	-
***Equus caballus* ADIC**	-	-----------&	-
***Canis lupus familiaris* ADIC**	-	-----------&	-
***Myotis lucifugus* ADIC**	-	-----------&	-
***Loxodonta africana* ADIC**	-	-----------&	-
***Homo sapiens* ADID**	-	-----------&	-
***Pan troglodytes* ADID**	-	-----------&	-
***Pongo abelii* ADID**	-	-----------&	-
***Nomascus leucogenys* ADID**	-	-----------&	-
***Macaca mulatta* ADID**	-	-----------&	-
***Otolemur garnettii* ADID**	-	-----------&	-
***Mus musculus* Adid**	-	-----------&	-
***Rattus norvegicus* Adid**	-	-----------&	-
***Cavia porcellus* ADID**	289	S----------&	289
***Oryctolagus cuniculus* ADID**	-	-----------&	-
***Bos taurus* ADID**	-	-----------&	-
***Equus caballus* ADID**	-	-----------&	-
***Canis lupus familiaris* ADID**	-	-----------&	-
***Felis catus* ADID**	-	-----------&	-
***Myotis lucifugus* ADID**	-	-----------&	-
***Dasypus novemcinctus* ADID**	-	-----------&	-
***Loxodonta africana* ADID**	-	-----------&	-
***Homo sapiens* ADIE**	239	-SPVF----A-&	243
***Pan troglodytes* ADIE**	239	-SPVF----A-&	243
***Pongo abelii* ADIE**	239	-SPVF----A-&	243
***Papio hamadryas* ADIE**	239	-SPVF----A-&	243
***Callithrix jacchus* ADIE**	239	-SPVF----A-&	243
***Otolemur garnettii* ADIE**	239	-SPVF----A-&	243
***Mus musculus* Adie**	239	-SPVF----A-&	243
***Rattus norvegicus* Adie**	239	-SPVF----A-&	243
***Homo sapiens* ADIF1**	-	-----------&	-
***Homo sapiens* ADIF2**	-	-----------&	-
***Pan troglodytes* ADIF1**	-	-----------&	-
***Pongo abelii* ADIF1**	-	-----------&	-
***Nomascus leucogenys* ADIF1**	-	-----------&	-
***Macaca mulatta* ADIF1**	-	-----------&	-
***Papio hamadryas* ADIF1**	-	-----------&	-

Microcebus murinus ADIF1	-	-----------&	-
Otolemur garnettii ADIF1	-	-----------&	-
Mus musculus Adif1	-	-----------&	-
Rattus norvegicus Adif1	-	-----------&	-
Cavia porcellus ADIF1	-	-----------&	-
Oryctolagus cuniculus ADIF1	-	-----------&	-
Ochotona princeps ADIF1	-	-----------&	-
Bos taurus ADIF1	-	-----------&	-
Equus caballus ADIF1	-	-----------&	-
Canis lupus familiaris ADIF1	-	-----------&	-
Felis catus ADIF1	-	-----------&	-
Dasypus novemcinctus ADIF1	-	-----------&	-
Loxodonta africana ADIF1	-	-----------&	-
Homo sapiens ADIG	334	LDSISEDDEL-&	343
Pan troglodytes ADIG	334	LDSISEDDEL-&	343
Pongo abelii ADIG	334	LDSISEDDEL-&	343
Callithrix jacchus ADIG	320	LDSISEDDEL-&	329
Otolemur garnettii ADIG	280	LDSISEDDEL-&	289
Mus musculus Adig	322	LDSISEDDEL-&	331
Rattus norvegicus Adig	323	LDSISEDDEL-&	332
Cavia porcellus ADIG	280	LDAISEDDEL-&	289
Oryctolagus cuniculus ADIG	280	LDSISEDDEL-&	289
Tursiops truncatus ADIG	332	LDSISEDDEL-&	341
Bos taurus ADIG	280	LDSISEDDEL-&	289
Equus caballus ADIG	321	LDSISEDDEL-&	330
Canis lupus familiaris ADIG	321	LDSISEDDEL-&	330
Myotis lucifugus ADIG	280	LDSISEDDEL-&	289
Homo sapiens ADIH	281	-DP-N---EV-&	285
Pan troglodytes ADIH	281	-DP-N---EV-&	285
Pongo abelii ADIH	281	-DP-N---EI-&	285
Nomascus leucogenys ADIH	281	-DP-N---EV-&	285
Macaca mulatta ADIH	281	-DP-N---EV-&	285
Papio hamadryas ADIH	281	-DP-N---EV-&	285
Mus musculus Adih	288	-DP-N---EV-&	292
Rattus norvegicus Adih	325	-DP-N---EV-&	329
Dipodomys ordii ADIH	281	-GP-N---EV-&	285
Cavia porcellus ADIH	281	-NP-N---EL-&	285
Bos taurus ADIH	298	-NP-S---EV-&	302
Equus caballus ADIH	281	-NP-N---EL-&	285
Canis lupus familiaris ADIH	281	-NP-N---EV-&	285
Pteropus vampyrus ADIH	281	-DP-N---EV-&	285
Loxodonta africana ADIH	281	-DP-N---EV-&	285
Homo sapiens ADII	-	-----------&	-
Pongo abelii ADII	-	-----------&	-
Macaca mulatta ADII	-	-----------&	-
Mus musculus Adii	-	-----------&	-
Rattus norvegicus Adii	-	-----------&	-
Bos taurus ADII	-	-----------&	-
Canis lupus familiaris ADII	-	-----------&	-
Dasypus novemcinctus ADII	-	-----------&	-
Homo sapiens ADIJ	-	-----------&	-
Pan troglodytes ADIJ	-	-----------&	-
Papio hamadryas ADIJ	-	-----------&	-
Mus musculus Adij	-	-----------&	-
Rattus norvegicus Adij	-	-----------&	-
Oryctolagus cuniculus ADIJ	-	-----------&	-
Bos taurus ADIJ	-	-----------&	-
Canis lupus familiaris ADIJ	-	-----------&	-
Loxodonta africana ADIJ	-	-----------&	-
Homo sapiens ADIK	-	-----------&	-
Pan troglodytes ADIK	-	-----------&	-
Gorilla gorilla ADIK	-	-----------&	-
Papio hamadryas ADIK	-	-----------&	-
Callithrix jacchus ADIK	-	-----------&	-
Rattus norvegicus Adik	-	-----------&	-
Cavia porcellus ADIK	-	-----------&	-
Equus caballus ADIK	-	-----------&	-
Canis lupus familiaris ADIK	-	-----------&	-
Dasypus novemcinctus ADIK	-	-----------&	-
Loxodonta africana ADIK	-	-----------&	-
Mus musculus Adil	-	-----------&	-
Rattus norvegicus Adil	-	-----------&	-
Homo sapiens ADIM	-	-----------&	-
Pan troglodytes ADIM	-	-----------&	-

Pongo abelii ADIM	-	-----------&	-
Nomascus leucogenys ADIM	-	-----------&	-
Papio hamadryas ADIM	-	-----------&	-
Callithrix jacchus ADIM	-	-----------&	-
Mus musculus Adim	-	-----------&	-
Rattus norvegicus Adim	-	-----------&	-
Cavia porcellus ADIM	-	-----------&	-
Bos taurus ADIM	-	-----------&	-
Equus caballus ADIM	-	-----------&	-
Canis lupus familiaris ADIM	-	-----------&	-
Homo sapiens ADIN	319	APPGLGASELL&	329
Pan troglodytes ADIN	319	APPGLGASELL&	329
Pongo abelii ADIN	319	APPGLGASELL&	329
Nomascus leucogenys ADIN	314	APPGLEASELL&	324
Macaca mulatta ADIN	321	APPGLGAPELL&	331
Callithrix jacchus ADIN	319	GPPGLGARELL&	329
Mus musculus Adin	317	GPPALKPPE-L&	326
Cavia porcellus ADIN	329	GDP-----E-L&	333
Tursiops truncatus ADIN	315	GPPGLGPPE-L&	324
Canis lupus familiaris ADIN	315	GPPGLGPPE-L&	324
Dasypus novemcinctus ADIN	315	GLPGLGPAE-L&	324
Loxodonta africana ADIN	314	HPPGLGPPE-L&	323
Homo sapiens ADIO	250	A-------EL-&	252
Pan troglodytes ADIO	250	A-------EL-&	252
Macaca mulatta ADIO	245	A-------EL-&	247
Bos taurus ADIO	249	A-------EL-&	251
Myotis lucifugus ADIO	249	A-------EL-&	251
Homo sapiens ADIP	377	T-------EP-&	379
Otolemur garnettii ADIP	279	A-------EP-&	281
Mus musculus Adip	279	S-------EP-&	281
Cavia porcellus ADIP	279	A-------EP-&	281
Bos taurus ADIP	302	N-------EP-&	304
Equus caballus ADIP	277	A-------EP-&	279
Loxodonta africana ADIP	331	A-------EP-&	333
Homo sapiens ADIQ	276	E-------DD-&	278
Pan troglodytes ADIQ	276	E-------DD-&	278
Pongo abelii ADIQ	276	E-------DD-&	278
Nomascus leucogenys ADIQ	276	E-------DD-&	278
Macaca mulatta ADIQ	276	E-------DD-&	278
Mus musculus Adiq	262	E-------DN-&	264
Rattus norvegicus Adiq	261	E-------DN-&	263
Homo sapiens ADIR	-	-----------&	-
Pan troglodytes ADIR	-	-----------&	-
Pongo abelii ADIR	-	-----------&	-
Nomascus leucogenys ADIR	-	-----------&	-
Macaca mulatta ADIR	-	-----------&	-
Papio hamadryas ADIR	-	-----------&	-
Callithrix jacchus ADIR	-	-----------&	-
Tarsius syrichta ADIR	-	-----------&	-
Otolemur garnettii ADIR	-	-----------&	-
Tupaia belangeri ADIR	-	-----------&	-
Mus musculus Adir	-	-----------&	-
Rattus norvegicus Adir	-	-----------&	-
Cavia porcellus ADIR	-	-----------&	-
Oryctolagus cuniculus ADIR	-	-----------&	-
Tursiops truncatus ADIR	-	-----------&	-
Bos taurus ADIR	-	-----------&	-
Equus caballus ADIR	-	-----------&	-
Canis lupus familiaris ADIR	-	-----------&	-
Myotis lucifugus ADIR	-	-----------&	-
Pteropus vampyrus ADIR	-	-----------&	-
Erinaceus europaeus ADIR	-	-----------&	-
Dasypus novemcinctus ADIR	-	-----------&	-
Choloepus hoffmanni ADIR	-	-----------&	-
Loxodonta africana ADIR	-	-----------&	-
