# Supplementary material for: Comparative genomic analysis of eutherian adiponectin genes
Source: Heliyon. 2018 Jun 6;4(6):e00647. doi: 10.1016/j.heliyon.2018.e00647 (PMC6040601; doi:10.1016/j.heliyon.2018.e00647)
Supplement: Supplementary data file 6 — Reference human ADIA amino acid sequence. The 33 invariant amino acid sites were shown using white letters on violet backgrounds and 1 forward amino acid site was shown using white letter on red background. The rectangle displayed low complexity region including 26 tripeptide repeats (G-x(2)). The arrows indicated 6 invariant amino acid sites F124, F142, F160, G166, Y168, F242 and G244 corresponding to invariant amino acid sites H98, W119, L139, G145, Y147, F246 and G247 in human TNLG1B (Premzl, 2016). The secondary structure elements A, A′, B′, B, C, D, E, F, G and H (Gaboriaud et al., 2003) were labelled below reference protein amino acid sequence using grey letters. The black triangle showed predicted N-terminal signal peptide cleavage site. [file mmc6.pdf]

```
                                 10        20        30        40        50        60        70        80
Homo sapiens ADIA   1 MMKIPWGSIPVLMLLLLLGLIDISQAQLSCTGPPAIPGIPGIPGTPGPDGQPGTPGIKGEKGLPGLAGDHGEFGEKGDP 80

                                 90       100       110       120       130       140       150
Homo sapiens ADIA  81 GIPGNPGKVGPKGPMGPKGGPGAPGAPGPKGESGDYKATQKIAFSATRTINVPLRRDQTIRFDHVITNMNNNYEPRSGKF 160
                                                                AAAAAA                A'A'A'  B'B   BB

                                          180       190       200       210       220       230       240
Homo sapiens ADIA 161 TCKVPGLYYFTYHASSRGNLCVNLMRGRERAQKVVTFCDYAYNTFQVTTGGMVLKLEQGENVFLQATDKNSLLGMEGANS 240
                      BB   CCCCCCCCCCC  DDDDDDDDD  EEEEEEEEEE       FFFFFFFFFFF  GGGGGGGG                H

                               250
Homo sapiens ADIA 241 IFSGFLLFPDMEA 253
                      HHHHHHHH
```
